# A C-to-B Atom Swap on Coumarins and Dibenzolactones

**DOI:** 10.1002/anie.202509674

**Published:** 2025-07-21

**Authors:** Tian You, Quang H. Luu, Junqi Li

**Affiliations:** Department of Chemistry, Iowa State University, Ames, IA 50011, USA

**Keywords:** Atom swapping, Boronic ester, Coumarin, Decarbonylation, Nickel catalysis

## Abstract

We report a carbon-to-boron “C-to-B” atom swap reaction to transform readily available coumarins into their isosteric benzoxaborins via a net replacement of the C=O group with a B–OH moiety. These conditions were applied to coumarin natural products and other 6–7-membered lactones (25 examples, 29%–93%). We leverage this methodology to transform a flat polyaromatic hydrocarbon into three-dimensional tribenzo[b.d.f]oxepines through a series of atom-swapping reactions followed by ring expansion via the oxaborin intermediate.

## Graphical Abstract

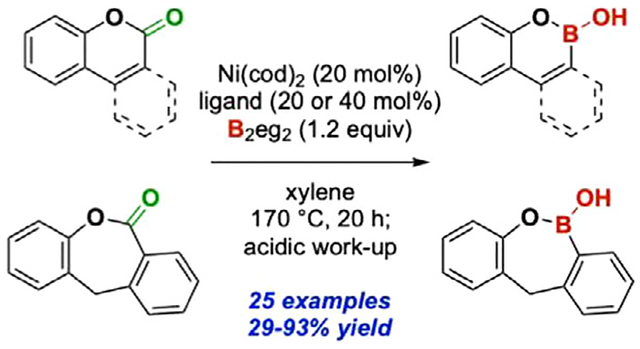

Cyclic boronic hemiesters, boron isosteres of lactones, display unique and valuable chemical properties. Herein, we report a novel method for their synthesis via nickel-catalyzed decarbonylative borylation, enabled by electron-rich phosphine ligands to achieve a formal “C-to-B” atom swap. This strategy efficiently transforms a variety of coumarin derivatives and seven-membered dibenzolactones into the corresponding benzoxaborines, showcasing broad functional group tolerance and synthetic versatility.

Cyclic boronic hemiesters are boron isosteres of lactones. In medicinal chemistry, the isosteric relationship between the boronic acids and esters has been utilized to great effect in the design of Lewis acidic transition state analog inhibitors that bind to critical nucleophilic residues in the active site of enzymes.^[1]^ Furthermore, the variety of intra- and intermolecular interactions^[2]^ by which cyclic boronic esters such as QPX7728 and Tavaborole engage their biological targets have made them increasingly valuable in drug discovery efforts (Figure 1a).^[3–6]^ As synthetic intermediates, cyclic boronic hemiesters are known to be competent coupling partners in Suzuki–Miyaura cross-coupling reactions,^[7–9]^ as precursors to (benzo)coumarins,^[9–11]^ and as boron enolates.^[12]^ The development of new approaches to rapidly access these compounds thus stands to advance their applications in medicinal chemistry and downstream methodology development.

A few synthetic approaches already exist for the synthesis of oxaborins (6-membered cyclic boronic hemiesters),^[7–23]^ the most common method being the borylation of an aryl or vinyl halide, followed by cyclization of the pendant phenol onto the newly-installed boron center.^[7,8]^ In recent patent literature,^[24]^ the boron-Wittig reaction was utilized to access oxaborin structures (Figure 1b). Yorimitsu and coworkers undertook an “aromatic metamorphosis” approach by converting benzofurans into benzoxaborins by Ni-catalyzed boron insertion.^[10,11]^ In this work, we report a conceptually distinct carbon-to-boron “atom swap” strategy to access benzoxaborins from their more abundant^[25,26]^ carbon isosteres (Figure 1c). During the course of our manuscript revision, a report on a carbonyl-to-boranyl exchange on aromatic lactones was recently published.^[27]^

We previously demonstrated that lactones are viable precursors to phosphine-ligated Ni(aryl)(aryloxo) metallacycles (Figure 2a) in our development of a carbon-to-oxygen (“C-to-O”) atom swapping reaction sequence.^[28]^ C–O reductive elimination from these and related intermediates^[28,29]^ can be challenging. We thus reasoned that intercepting these Ni(aryl)(aryloxo) intermediates by transmetalation with a bisboron reagent would form the required C–B bond. The proposed catalytic cycle would proceed via the oxidative addition of Ni(0) into the acyl C–O bond in coumarin **1a**, CO deinsertion, followed by transmetallation with a B_2_(OR)_4_ reagent with the pendant aryloxo ligand acting as an internal base, and reductive elimination to furnish a bisboron intermediate **2a-B** (Figure 2b). Acidic work-up would then generate the desired oxaborin. Accomplishing this transformation would also establish coumarins as viable electrophiles by C(acyl)–O bond activation by Ni catalysts.

We started the investigation by evaluating ligands for the proposed decarbonylative borylation on coumarin **1a**. After a set of ligand screening, P(^*n*^Bu)_3_ turned out to be the optimal ligand for the desired transformation. Other monodentate phosphine ligands that were bulkier or less electron-rich gave no to low yields (see Supporting Information for full optimization details). It is worth noting that the IPr ligand could also give a competitive yield, showing the potential of the *N*-heterocyclic carbene (NHC) ligands in promoting such transformations. We also evaluated different bisboron reagents, which had a significant impact on the borylation reaction. The commonly used B_2_nep_2_ and B_2_pin_2_ were less effective than B_2_eg_2_ (Figure 3, entries 7 and 10, 35% and 36%, respectively). Other bisboron reagents that are bulkier and/or with lower Lewis acidity than B_2_eg_2_ gave lower yields (Figure 3, entries 8 and 9), presumably due to their lower reactivity in transmetallation.^[30,31]^ However, the more electron-deficient B_2_cat_2_ was also an unsuitable borylating reagent.

The Ni(cod)_2_/P(^*n*^Bu)_3_ catalyst derived from our optimization studies is consistent with the use of nickel complexes bearing strong *σ*-donating ligands in Ni-catalyzed intermolecular decarbonylative borylation of esters by the Shi and Rueping groups.^[32,33]^ However, directly applying the reported reaction conditions to **1a** gave no detectable yields of **2a**. Furthermore, we found that the addition of base, which is often used to promote transmetalation, led to a lower yield of **2a**, highlighting the nuances of developing seemingly similar decarbonylative transformations on different substrates.

The optimal conditions were then applied to transform a collection of coumarins, including natural products and fluorophores, into their boron isosteres (Figure 4). Common substituents on the benzene ring of coumarin natural products were tolerated. For instance, a C-to-B swap on toncarine, a food additive and fragrance,^[34]^ resulted in a 93% yield of **2b**. Derivatives of umbeliferone gave 68% and 84% yields of the corresponding benzoxaborins **2c** and **2d**, respectively. A C-to-B swap on the pharmaceutical NSC31868 resulted in a 41% yield of the product **2e**. 5,7-dimethoxy substituted coumarin **2f** gave a modest 38% yield, but a higher 47% yield was obtained when the ligand was switched to dcype.

The borylation of 4-substituted coumarins **2g**–**2l** could not be effectively promoted by the Ni/P(^*n*^Bu)_3_ catalytic system. However, switching the ligand from P(^*n*^Bu)_3_ to dcype significantly improved the reaction yield to 61% for **2 g** (see Supporting Information for optimization details). Other 4-methyl substituted coumarins followed the same trend: dcype outperformed P(^*n*^Bu)_3_. An EOM-protected hymecromone gave 58% of **2i**, the structure of which was further confirmed by X-ray crystallography.^[35]^ Protected 4-methylesculetin also gave 71% of the boron isostere **2j**. Similarly, another non-natural coumarin **2k** can be transformed into boron-containing products. It is worth mentioning that 3-methyl substituted coumarin also gave a better yield of **2l** with dcype than with P(^*n*^Bu)_3_. The decarbonylative borylation on 4-aryl substituted similarly favored dcype as the preferred ligand. Both electron-rich and electron-deficient aryl substituents on C4 could be tolerated (**2m–2p**). Benzocoumarins are viable substrates for this protocol as well. The preference of ligands is in accordance with that of 4-substituted coumarins, in which dcype is more suitable for the reaction. A benzocoumarin with a *π*-extended system also gave a 55% yield of the product **2r**. An effect of protecting groups on yields was also observed. In particular, the methyl-protected urolithin B gave a 44% yield of the dibenzoxaborin **2 s**, while the EOM-protected one gave a 49% yield of **2t**. In contrast, the methyl-protected umbeliferone gave a better yield than the EOM-protected derivative. In addition, the C-to-B swap method was successfully applied to access the 7-membered dibenzoxaborepins, which, to the best of our knowledge, have not been reported in the literature. Without modifying the reaction conditions, 7-membered dibenzolactones with electron-rich and electron-deficient substituents were transformed into the corresponding cyclic boronic hemiesters **2u–2y** in 31%–79% yields. The ligand preference in such substrates was not as prominent as in the cases of coumarin. An X-ray crystal structure of **2w** was obtained, which confirms that it exists as a cyclic boronic hemiester.^[35]^

We next sought to engage oxaborins as synthetic intermediates in new transformations. The Yorimitsu group demonstrated that oxaborins can undergo Suzuki–Miyaura cross-coupling reactions with iodobenzene to give predominantly Z-alkenes.^[10]^ We saw an opportunity to combine this C–C bond formation with a C–O bond-forming reaction in one pot. This tandem reaction successfully transformed oxaborin **2a** to oxazepin **4** in > 99% yield using a Pd/BrettPhos catalyst, indicating retention of the Z-alkene configuration under these reaction conditions (Figure 5a).

Finally, inspired by strong interest in accessing boron-doped polyaromatic hydrocarbons for their optical properties,^[16,18,36–38]^ we questioned whether these molecules can be accessed from their all carbon isosteres through a series of atom-swapping reactions. In this vein, we accomplished a “C-to-O” swap on phenanthrene **5** by oxidation to the lactone **1q** by oxone in one step in 62% yield (Figure 5b).^[39]^ Using the decarbonylative borylation reaction we developed, a “C-to-B” swap on 2.0 mmol scale then converted **1q** to **2q** in 47% yield without modification of our standard conditions. Further molecular editing of the two-dimensional polyaromatic hydrocarbon core using the arene insertion conditions we developed for **2a** then gave the heterocycles **6** and **7** containing the tribenzo[b.d.f]oxepine framework for which few synthetic methods exist.^[40,41]^ Collectively, the transformation of **1a** to oxepins and **5** to tribenzoxepines further advances the synthetic utility of cyclic boronic hemiesters.

A mechanistic question that we wish to answer is why P(^*n*^Bu)_3_ outperforms dcype for 4-H coumarin substrates **2a–2e** while the opposite is true for 4-methyl and 4-aryl substituted derivatives **2g–2l**. We first explored an alternative mechanism, which involves the formation of benzofuran, followed by boron insertion into the benzofuran reported by Yorimitsu and coworkers.^[10]^ A later report also demonstrated that this chemistry is viable with several electron-rich phosphines.^[21]^ Competition studies between a benzofuran and a coumarin with a similar backbone (**3** vs. **2b**, and **4** vs. **2h**, Figure 6a) showed a large conversion to the coumarin-related benzoxaborin but very little conversion to the benzofuran-related product. The inferior performance of the benzofuran substrates shows that the mechanism involving the borylation of benzofuran is unlikely to be the major reaction pathway.

A series of stoichiometric reactions without the bisboron reagent revealed the formation of a nickelacycle species **dcype-Ni-I** arising from strong back-donation of the Ni complex to the C3=C4 bond of the coumarin.^[42]^ We observed that nickelacycle formation occurred more readily with coumarin **1a** compared to coumarins with 4-substituents (Figure 6b, entry 1 vs. entries 2 and 3). This observation aligns with the trend in yields of **2a**<<**2g** < **2m** (Figure 4) with the Ni/dcype system, suggesting that substrate **1a** contributes to catalyst deactivation when dcype ligand is used. In contrast, the Ni–P(^*n*^Bu)_3_ catalyst exhibited minimal substrate dependence in nickelacycle formation—**1a**, **1g**, and **1m** underwent quantitative conversion to the corresponding nickelacycle species. Furthermore, the rate of nickelacycle formation is significantly different for dcype and P(^*n*^Bu)_3_ (2 days vs. 30 min, see Section S4.1). Based on these results, we speculate that the mechanism is more complicated than what is initially depicted in Figure 2b and may involve the nickelacycle as an off-cycle species. Consequently, the catalyst resting state and therefore the detailed mechanism of the reaction may be different for each ligand and substrate.

In conclusion, we have achieved a C-to-B atom-swapping reaction via Ni-catalyzed decarbonylative borylation to transform a variety of coumarins and benzocoumarins into their boron isosteres in one step. The same catalytic reaction can be applied to dibenzolactones to generate rare 7-membered cyclic boronic hemiesters. We further utilized the new cyclic boronic hemiesters to access medium-ring oxygen heterocycles for which few synthetic methods exist. Finally, our preliminary investigations into the molecular editing of polyaromatic hydrocarbons demonstrate that C-to-O and C-to-B atom swapping reactions can be utilized to transform these hydrocarbons into valuable boron-doped heterocycles and new three-dimensional architectures.

## Supplementary Material

SI file 1

cif files

Additional supporting information can be found online in the Supporting Information section

## Figures and Tables

**Figure 1.**
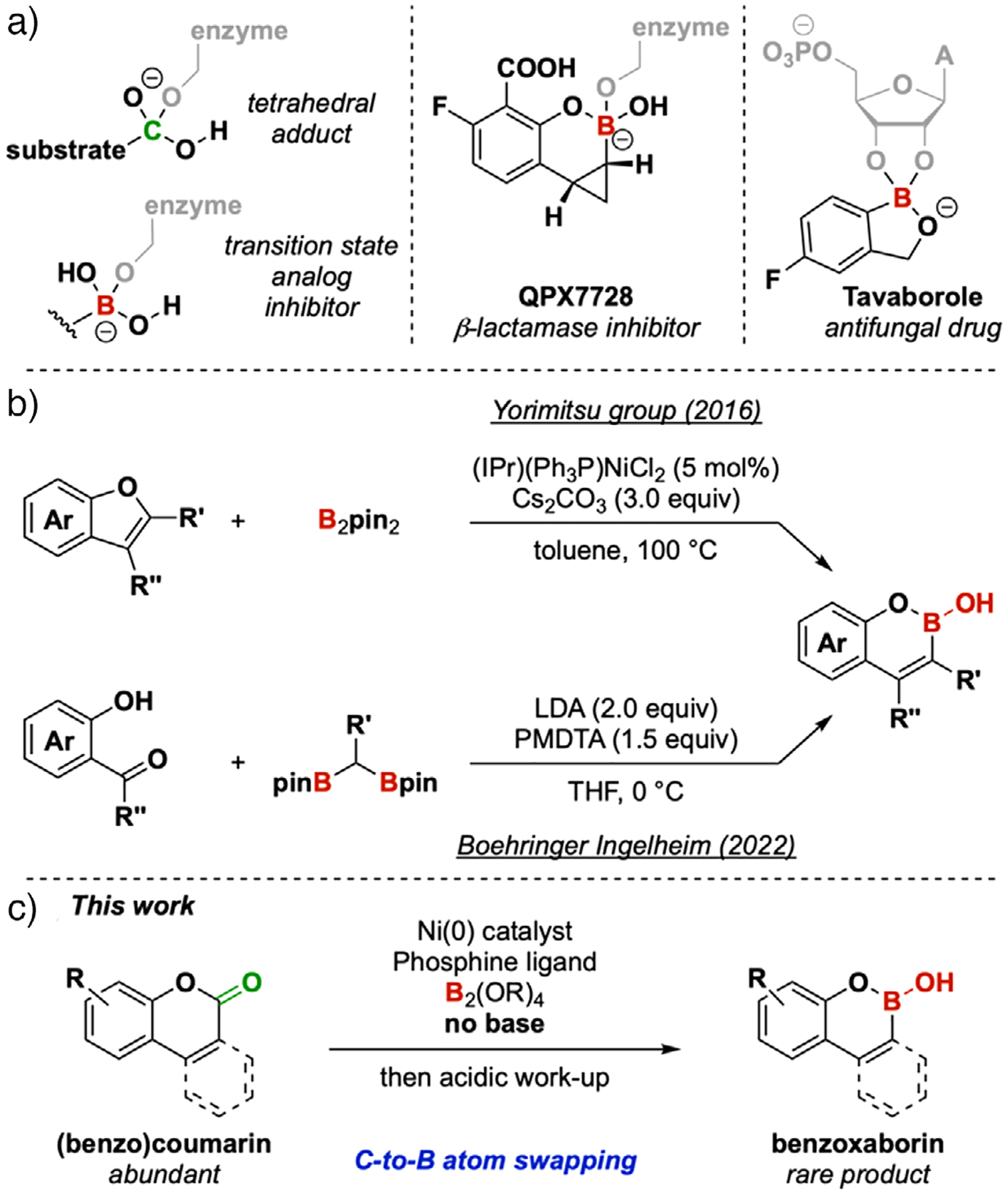
a) The medicinal chemistry of boronic acids and esters arises from their ability to bind to nucleophilic residues. b) Recent strategies for benzoxaborin synthesis. c) This work.

**Figure 2.**
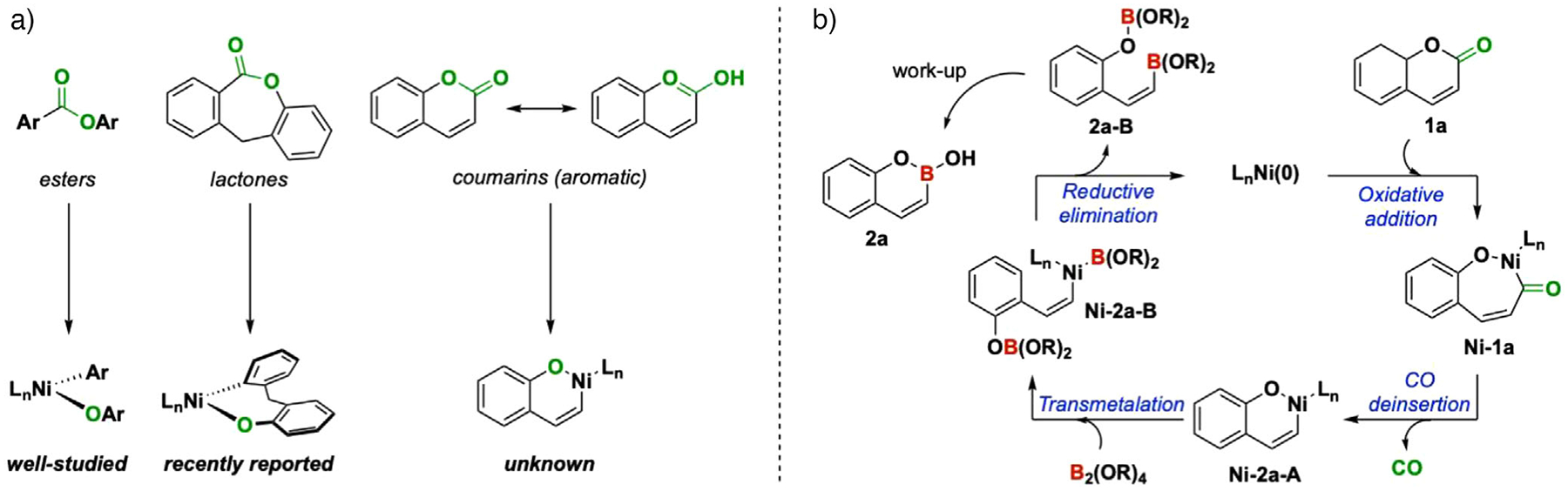
a) Esters as precursors to Ni(aryl)(aryloxo) intermediates. b) Proposed catalytic cycle for a nickel-catalyzed decarbonylative–borylation reaction.

**Figure 3.**
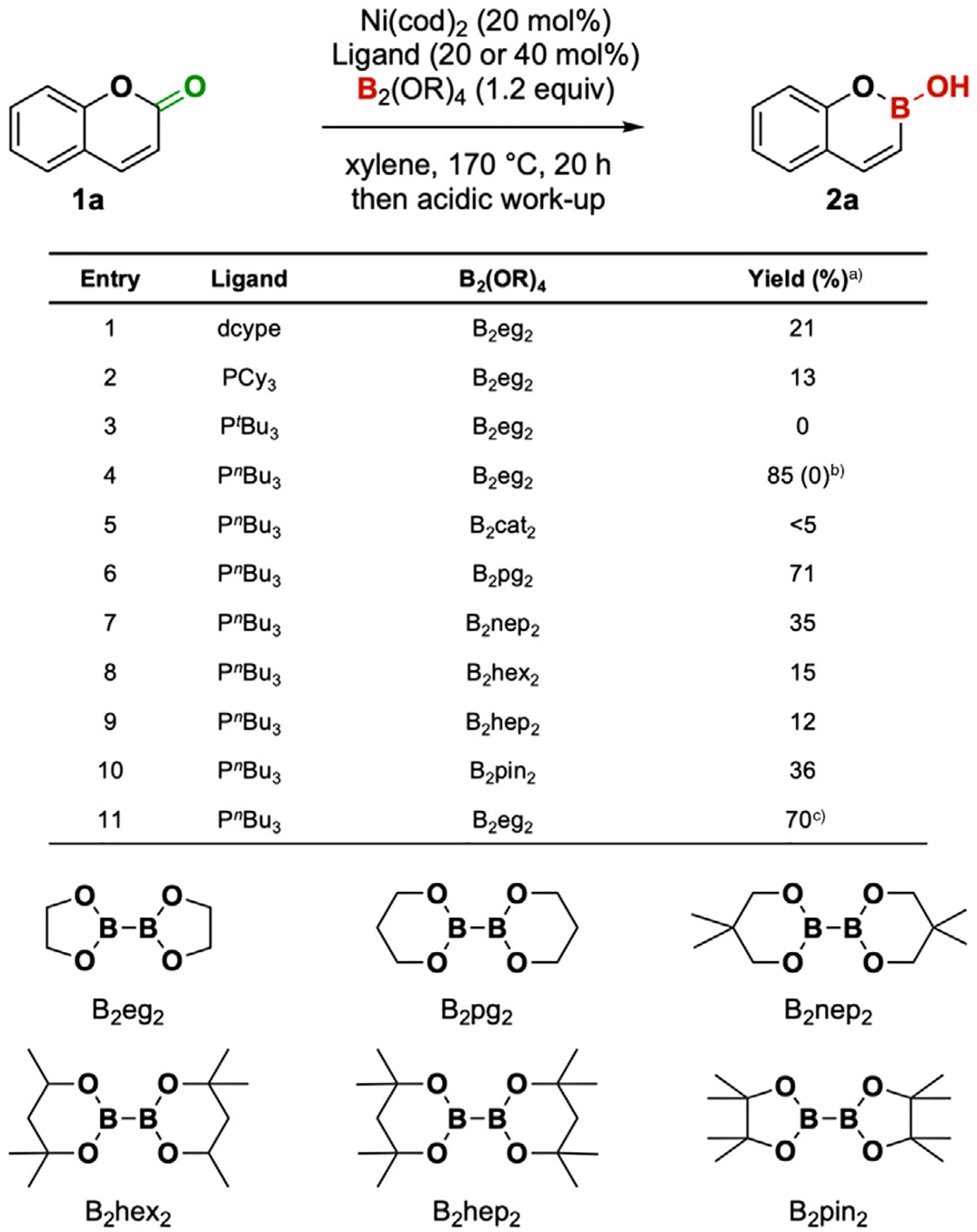
Reaction optimization. Reaction conditions: **1a** (0.200 mmol), Ni(cod)_2_ (0.040 mmol, 20 mol%), ligands (40 mol% for monodentate, 20 mol% for bidentate), B_2_(OR)_4_ (0.240 mmol, 1.2 equiv), xylene (1.0 mL, 0.20 M). ^a)^Isolated yield was obtained as described in the Supporting Information. ^b)^Ni(cod)_2_ was replaced by Pd_2_(dba)_3_. ^c)^1.5 equiv. Na_2_CO_3_ was added.

**Figure 4.**
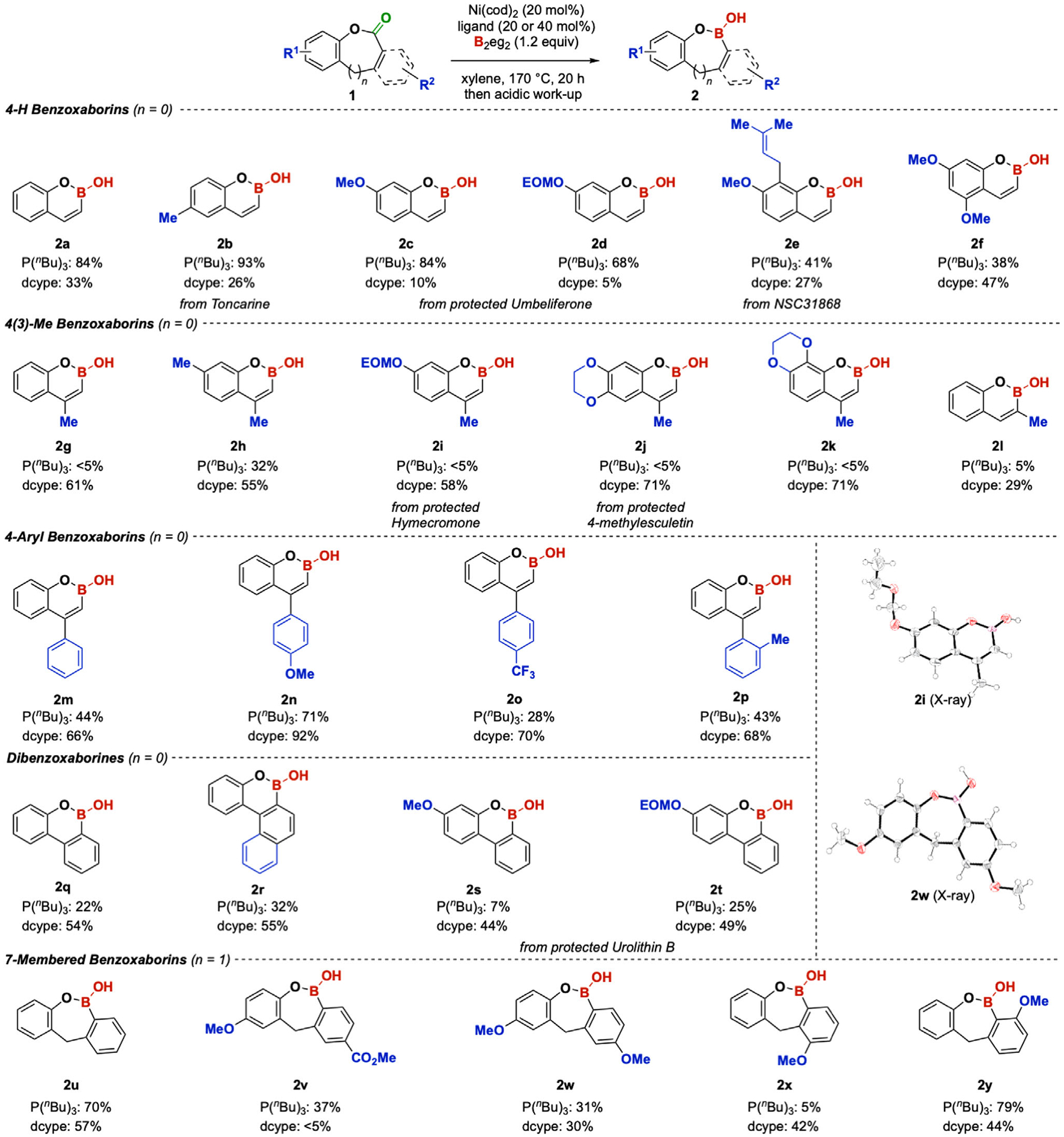
Scope of the C-to-B swap. Reaction condition: 1 (0.200 mmol), Ni(cod)_2_ (0.040 mmol, 20 mol%), ligand (40 mol% for P(^*n*^Bu)_3_, 20 mol% for dcype), B_2_eg_2_ (0.240 mmol, 1.2 equiv.), xylene (1.0 mL, 0.20 M). Isolated yields were obtained as described in the Supporting Information.

**Figure 5.**
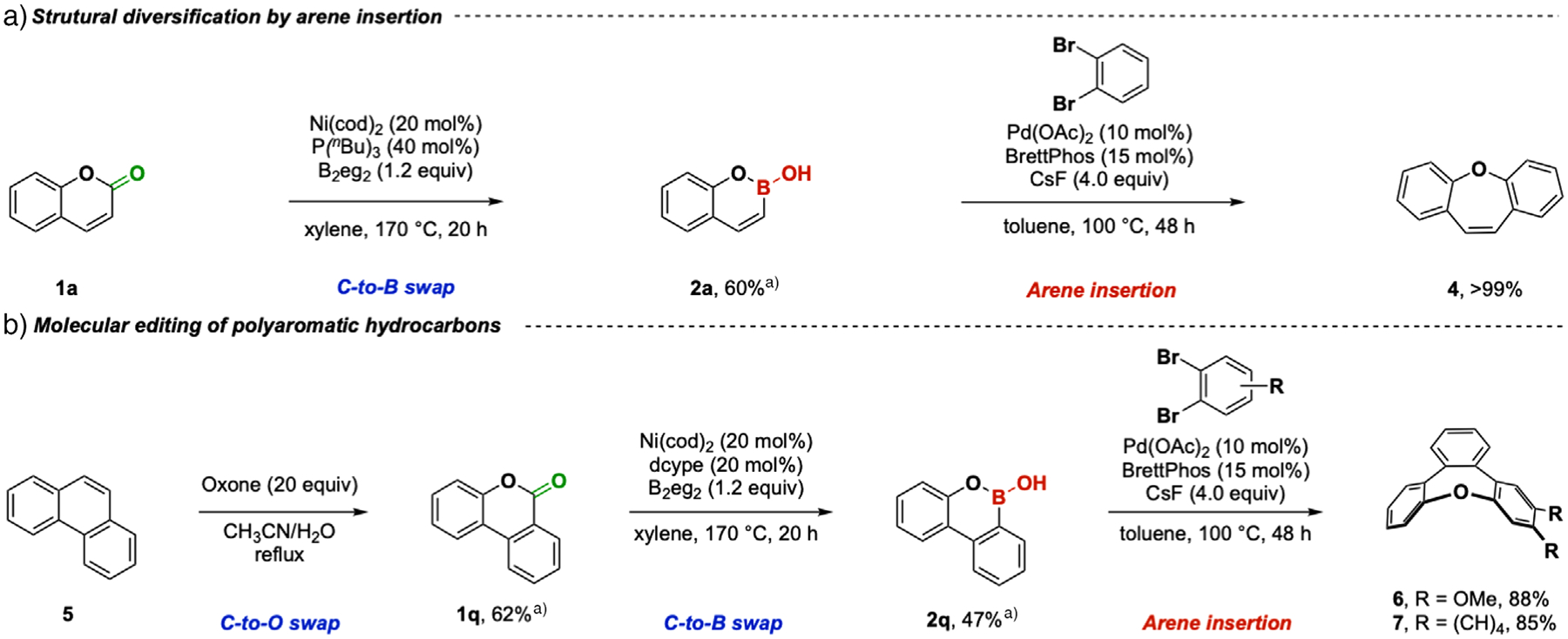
Applications of C-to-B swap. a) Arene insertion into the oxaborin intermediate; and b) editing polyaromatic hydrocarbons. ^a)^Yield of a 2.00 mmol scale reaction.

**Figure 6.**
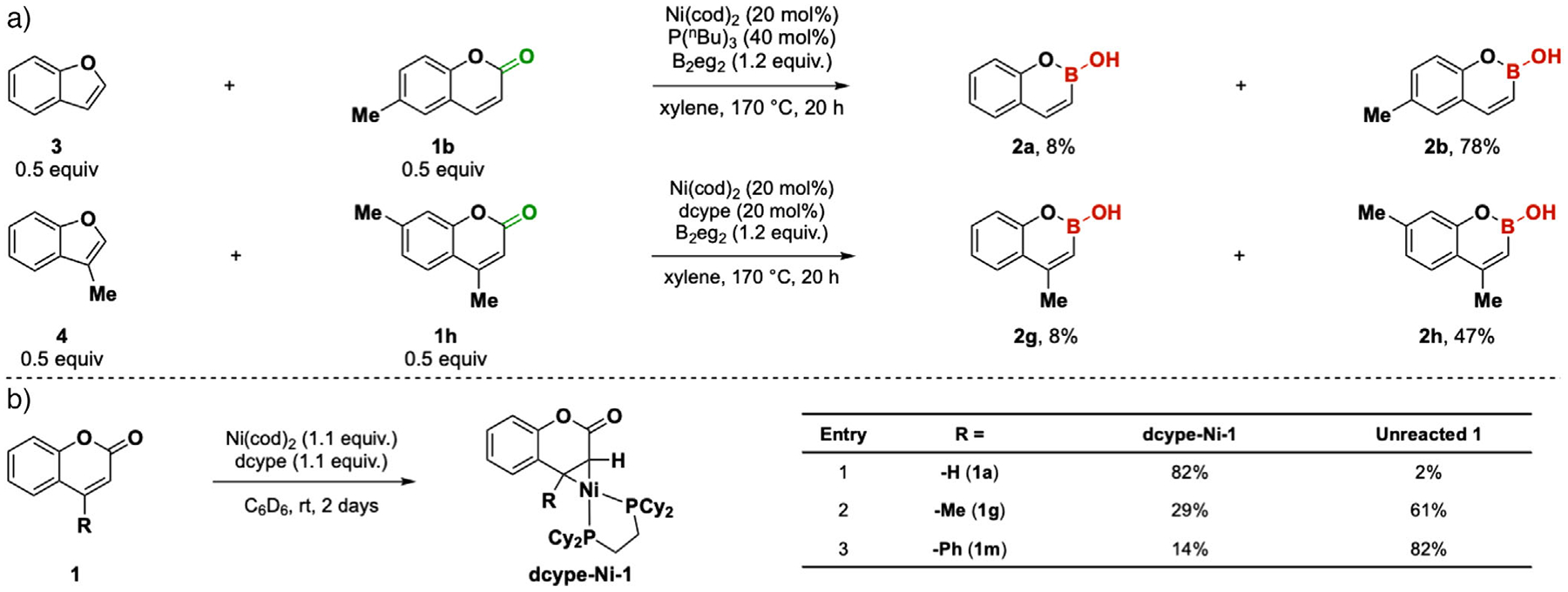
Mechanistic study: a) Competition experiments between coumarin and benzofuran substrate. b) Formation of nickelacycles between Ni-dcype and different coumarin substrates.

## Data Availability

The data that support the findings of this study are available in the Supporting Information of this article.
