# Supplementary material for: A C-to-B Atom Swap on Coumarins and Dibenzolactones
Source: Angew Chem Int Ed Engl. Author manuscript; Available in PMC 2025 Jul 29. (PMC12306449; doi:10.1002/anie.202509674)
Supplement: SI file 1 [file NIHMS2099008-supplement-SI_file_1.pdf]

Supporting Information for:

A C-to-B Atom Swap on Coumarins and Dibenzolactones

Tian You,ǂ[a] Quang H. Luu,ǂ[a] and Junqi Li[a]

[a] Department of Chemistry, Iowa State University

ǂT. You and Q. H. Luu contributed equally to this work.

correspondence to:

Junqi Li, junqili@iastate.edu

Table of Contents

1. Materials and Methods 2

2. Abbreviations 3

3. Experimental procedures for additional optimization studies 4

3.1. Ligand screen (Table S1) 4

3.2. Catalyst loading screen (Table S2) 4

3.3. Ligand loading screen (Table S3) 5

3.4. Bisboron reagent screen (Table S4) 5

3.5. Solvent screen (Table S5) 6

3.6. Concentration screen (Table S6) 7

4. Experimental procedures for Mechanistic Study 8

4.1. Stoichiometric experiments with coumarins and nickel catalyst 8

4.2. Additional experiments with benzofuran **3** 15

5. Experimental procedures and characterization data for compounds in Figure 4 17

5.1. Synthesis of substrates and characterization data for substrates 17

5.1.1. Synthesis of substrate **1d** 17

5.1.2. Synthesis of substrate **1i** 17

5.1.3. Synthesis of substrate **1t** 18

5.1.4. Synthesis of B_2eg_2 19

5.2. General procedure and characterization data for decarbonylative borylation products 19

5.3. Unsuccessful substrates 35

6. Experimental procedures for applications of C-to-B swap 35

6.1. Synthesis of dibenzo[b,f]oxepine (**4**) from coumarin **1a** 35

6.2. Synthesis of heteroarenes **6** and **7** from phenanthrene **5** 36

7. References 38

8. NMR spectra of new compounds 39

1. Materials and Methods

Room temperature is defined as 23 °C. All reagents were purchased from commercial suppliers and used without further purification, unless otherwise noted. *N*,*N*-dimethylformamide, dichloromethane, diethyl ether and toluene were obtained from a 800L Solvent Purification System by Pure Process Technology, in which the solvent was dried over alumina and dispensed under an atmosphere of Ar. Where indicated, experiments were carried out in a nitrogen-filled mBraun glovebox. All other solvents were purchased from commercial suppliers and used without further purification, unless otherwise noted. Rotary evaporation was carried out at 40 °C.

Routine ^{1}H NMR spectra were recorded on Bruker 400 or 600 MHz spectrometers at ambient temperature unless otherwise stated. All NMR solvents were purchased from Cambridge Isotope Laboratories and used without further purification. Acetone-d_6, chloroform-d_3, and dichloromethane-d_2, were stored at ambient temperature. Benzene-d_6 was stored in the glovebox over 4Å molecular sieves. Spectra were processed using MestReNova 14.0.1 using the automatic phasing and polynomial baseline correction capabilities. Splitting was determined using the automatic multiplet analysis function with manual intervention as necessary. Spectral data are reported as follows: chemical shift (multiplicity [singlet (s), broad singlet (br s), doublet (d), triplet (t), quartet (q), pentet (p), multiplet (m), doublet of doublets (dd), doublet of doublet of doublets (ddd), doublet of triplet of doublets (dtd), doublet of doublet of doublet of doublets (dddd), doublet of triplets (dt), triplet of doublets (td), etc.], coupling constant, integration). Chemical shifts are reported in ppm (δ), and coupling constants are reported in Hz. ^{1}H Resonances are referenced to solvent residual peaks for $(CD_3)_2CO$ (2.05 ppm), $CDCl_3$ (7.26 ppm), C_6D_6 (7.16 ppm), and CD_2Cl_2 (5.32 ppm). ^{13}C Resonances are referenced to solvent residual peaks for $(CD_3)_2CO$ (29.84 and 206.06 ppm), $CDCl_3$ (77.16 ppm), C_6D_6 (128.06 ppm), and CD_2Cl_2 (53.84 ppm). Note: Small deviations in chemical shifts may be observed depending on the concentration of NMR samples. ^{11}B NMR was run with background suppression.

Analytical thin-layer chromatography was performed using 60 Å Silica Gel F_{254} pre-coated plates (0.25 mm thickness). TLC plates were visualized by irradiation with a UV lamp. Normal-phase column chromatography was performed using 60 Å Silica Gel (32–62 micron) with an appropriate mobile phase composition and gradient. Automated column chromatography was performed using a CombiFlash NextGen 300+ System by Teledyne ISCO on RediSep Rf Gold silica gel columns or RediSep Rf disposable flash columns. Positive (and/or negative) ion mode mass spectra were obtained using the Agilent (Santa Clara, CA) mass spectrometer. Agilent LC 6470 system, equipped with Agilent autosampler was used. Infrared spectra were recorded on a Bruker Tensor 37 ATR/FT-IR spectrometer, and ν_{max} are reported in cm^{-1}.

2. Abbreviations

cod	Cyclooctadiene
CV	Column volumes
Cy	Cyclohexyl
DCM	Dichloromethane
dcype	1,2-Bis(dicyclohexylphosphino)ethane
DMF	*N*,*N*-Dimethylformamide
EOMCl	Chloromethyl ethyl ether
Et	Ethyl
EtOAc	Ethyl acetate
HRMS	High-resolution mass spectrometry
IR	Infrared
^{n}Bu	*n*-Butyl
NMR	Nuclear magnetic resonance
rt	Room temperature
^{t}Bu	*tert*-Butyl
TLC	Thin-layer chromatography

3. Experimental procedures for additional optimization studies

3.1. Ligand screen (Table S1)

A 16 × 150 mm reaction tube was charged with a magnetic stir bar and **1a** (29.2 mg, 0.200 mmol). The tube was brought into a glovebox, then Ni(cod)$_2$ (11 mg, 0.040 mmol, 20 mol%), a ligand (20 mol% for bidentate and 40 mol% for monodentate), B_2eg_2 (34.0 mg, 0.240 mmol, 1.2 equiv), and xylene (1.00 mL) were added. The tube was sealed, brought outside, and placed in an oil bath preheated at 170 °C. The mixture was stirred (700 rpm) for 20 h. After cooling to room temperature, the mixture was directly loaded onto a Combiflash sample cartridge packed with silica gel (5 × 10 cm). The sample was chromatographed by Combiflash using a 12-g Gold column and ethyl acetate/hexanes mixture as the eluent (0% ethyl acetate for 5 CV, 10% ethyl acetate for 10 CV, 20% ethyl acetate for 10 CV, 25% ethyl acetate for 10 CV, then 30% ethyl acetate for 5 CV). The product **2a** started to elute at 25 CV. The combined product-containing fractions were concentrated by a rotary evaporator. The residue was dried to give **2a** as a white solid. Characterization data are reported in section 5.2.

Table S1. Ligand screen.

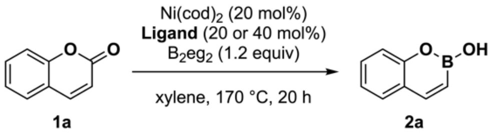

Entry	Ligands	Isolated yields (%)	Entry	Ligands	Isolated yields (%)
1	IMes	21	8	PCy_3	13
2	IMes^{Me}	25	9	P^nBu_3	85
3	IPr	60	10	PEt_3	76
4	I^{t}Bu	0	11	Ph_2PMe	12
5	sIMes	15	12	dppe	7
6	P^tBu_3	0	13	dcype	21
7	P^iPr_3	0	14	dcypf	0

3.2. Catalyst loading screen (Table S2)

A 16 × 150 mm reaction tube was charged with a magnetic stir bar and **1a** (29.2 mg, 0.200 mmol). The tube was brought into a glovebox, then Ni(cod)$_2$ (5-20 mol%), P^nBu_3 (10-40 mol%), B_2eg_2 (34.0 mg, 0.240 mmol, 1.2 equiv), and xylene (1.00 mL) were added. The vial was sealed, brought outside, and placed in an aluminum heating block preheated at 170 °C. The mixture was stirred (700 rpm) for the indicated time in Table S2. After cooling to room temperature, the mixture was directly loaded onto a Combiflash sample cartridge packed with silica gel (5 × 10 cm). The sample was chromatographed by Combiflash using a 12-g Gold column and ethyl acetate/hexanes mixture as the eluent (0% ethyl acetate for 5 CV, 10% ethyl acetate for 10 CV, 20% ethyl acetate for 10 CV, 25% ethyl acetate for 10 CV, then 30% ethyl acetate for 5 CV). The product **2a** started to elute at 25 CV. The combined product-containing fractions were concentrated by rotary evaporator. The residue was dried to give **2a** as a white solid. Characterization data are reported in section 5.2.

Table S2. Catalyst loading screen.

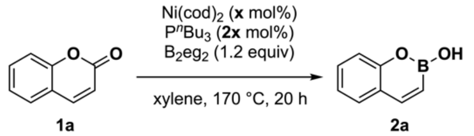

Entry	$Ni(cod)_2$ (mol%)	isolated yield (%)
1	20	85
2	15	88
3	10	88
4	5.0	54

3.3. Ligand loading screen (Table S3)

A 16 × 150 mm reaction tube was charged with a magnetic stir bar and **1a** (29.2 mg, 0.200 mmol). The tube was brought into a glovebox, then $Ni(cod)_2$ (11.0 mg, 0.0400 mmol, 20 mol%), P^nBu_3 (40-100 mol%), B_2eg_2 (34.0 mg, 0.240 mmol, 1.2 equiv), and xylene (1.00 mL) were added. The tube was sealed, brought outside, and placed in an oil bath preheated at 170 °C. The mixture was stirred (700 rpm) for 20 h. After cooling to room temperature, the mixture was directly loaded onto a Combiflash sample cartridge packed with silica gel (5 × 10 cm). The sample was chromatographed by Combiflash using a 12-g Gold column and ethyl acetate/hexanes mixture as the eluent (0% ethyl acetate for 5 CV, 10% ethyl acetate for 10 CV, 20% ethyl acetate for 10 CV, 25% ethyl acetate for 10 CV, then 30% ethyl acetate for 5 CV) .The product **2a** started to elute at 25 CV. The combined product-containing fractions were concentrated by rotary evaporator. The residue was dried to give **2a** as a white solid. Characterization data are reported in section 5.2.

Table S3. Ligand loading screen.

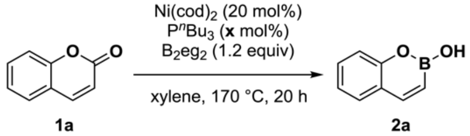

Entry	P^nBu_3 (mol%)	$Ni(cod)_2/P^nBu_3$ ratio	isolated yield (%)
1	40	1/2	85
2	60	1/3	78
3	80	1/4	74
4	100	1/5	76

3.4. Bisboron reagent screen (Table S4)

A 16 × 150 mm reaction tube was charged with a magnetic stir bar and **1a** (29.2 mg, 0.200 mmol). The tube was brought into a glovebox, then $Ni(cod)_2$ (11.0 mg, 0.0400 mmol, 20 mol%), P^nBu_3 (16.2 mg, 0.0800 mmol, 40 mol%), $B_2(OR)_4$ (0.120 mmol, 1.2 equiv), and xylene (1.00 mL) were added. The tube was sealed, brought outside, and placed in an oil bath preheated at 170 °C. The

mixture was stirred (700 rpm) for 20 h. After cooling to room temperature, the mixture was directly loaded onto a Combiflash sample cartridge packed with silica gel (5 × 10 cm). The sample was chromatographed by Combiflash using a 12-g Gold column and ethyl acetate/hexanes mixture as the eluent (0% ethyl acetate for 5 CV, 10% ethyl acetate for 10 CV, 20% ethyl acetate for 10 CV, 25% ethyl acetate for 10 CV, then 30% ethyl acetate for 5 CV). The product **2a** started to elute at 25 CV. The combined product-containing fractions were concentrated by rotary evaporator. The residue was dried to give **2a** as a white solid. Characterization data are reported in section 5.2.

Table S4. Reagent screen.

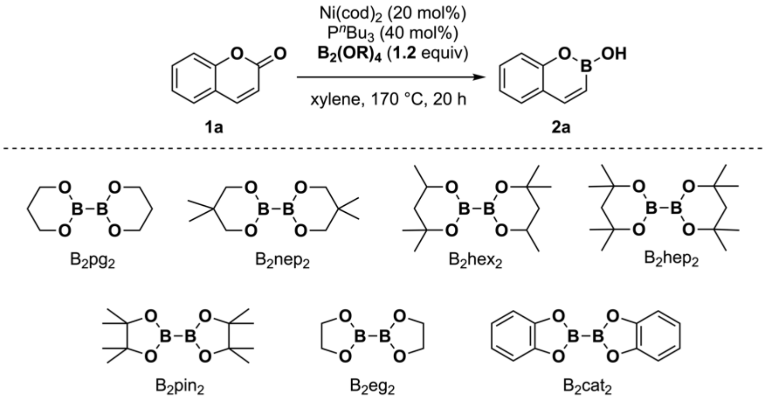

Entry	$B_2(OR)_4$	isolated yield (%)
1	B_2eg_2	85
2	B_2pg_2	71
3	B_2hex_2	15
4	B_2hep_2	12
5	B_2pin_2	36
6	B_2nep_2	35
7	B_2cat_2	<5

3.5. Solvent screen (Table S5)

A 16 × 150 mm reaction tube was charged with a magnetic stir bar and **1a** (29.2 mg, 0.200 mmol). The tube was brought into a glovebox, then $Ni(cod)_2$ (11.0 mg, 0.0400 mmol, 20 mol%), P^nBu_3 (16.2 mg, 0.0800 mmol, 40 mol%), B_2eg_2 (34.0 mg, 0.240 mmol, 1.2 equiv), and a solvent (1.00 mL) were added. The tube was sealed, brought outside, and placed in an oil bath preheated at 170 °C. The mixture was stirred (700 rpm) for 20 h. After cooling to room temperature, the mixture was directly loaded onto a Combiflash sample cartridge packed with silica gel (5 × 10 cm). The sample was chromatographed by Combiflash using a 12-g Gold column and ethyl acetate/hexanes mixture as the eluent (0% ethyl acetate for 5 CV, 10% ethyl acetate for 10 CV, 20% ethyl acetate for 10 CV, 25% ethyl acetate for 10 CV, then 30% ethyl acetate for 5 CV). The product **2a** started to elute at 25 CV. The combined product-containing fractions were concentrated by rotary evaporator. The residue was dried to give **2a** as a white solid. Characterization data are reported in section 5.2.

Table S5. Solvent screen.

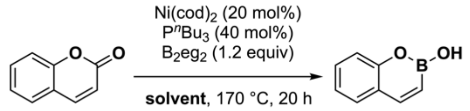

Entry	Solvent	isolated yield (%)
1	toluene	85
2	xylene	85
3	mesitylene	41
4	dioxane	54

3.6. Concentration screen (Table S6)

A 16 × 150 mm reaction tube was charged with a magnetic stir bar and **1a** (29.2 mg, 0.200 mmol). The tube was brought into a glovebox, then $Ni(cod)_2$ (11.0 mg, 0.0400 mmol, 20 mol%), P^nBu_3 (16.2 mg, 0.0800 mmol, 40 mol%), B_2eg_2 (34.0 mg, 0.240 mmol, 1.2 equiv), and xylene (1.00-3.00 mL) were added. The tube was sealed, brought outside, and placed in an oil bath preheated at 170 °C. The mixture was stirred (700 rpm) for 20 h. After cooling to room temperature, the mixture was directly loaded onto a Combiflash sample cartridge packed with silica gel (5 × 10 cm). The sample was chromatographed by Combiflash using a 12-g Gold column and ethyl acetate/hexanes mixture as the eluent (0% ethyl acetate for 5 CV, 10% ethyl acetate for 10 CV, 20% ethyl acetate for 10 CV, 25% ethyl acetate for 10 CV, then 30% ethyl acetate for 5 CV). The product **2a** started to elute at 25 CV. The combined product-containing fractions were concentrated by rotary evaporator. The residue was dried to give **2a** as a white solid. Characterization data are reported in section 5.2.

Table S6. Concentration screen.

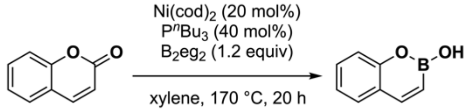

1a 2a

Entry	Xylene (mL)	Concentration (M)	isolated yield (%)
1	3.00	0.067	73
2	2.00	0.10	71
3	1.00	0.20	85

4. Experimental procedures for Mechanistic Study

4.1. Stoichiometric experiments with coumarins and nickel catalyst

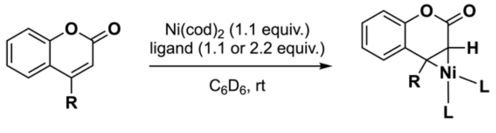

General procedure: In a glovebox, a 2-dram vial was charged with a magnetic stir bar, coumarin **1a**, **1g**, or **1m** (0.0400 mmol), Ni(cod)$_2$ (12.1 mg, 0.0440 mmol), dcype (0.0440 mmol) or P(^{n}Bu)$_3$ (0.0880 mmol), 2,6-dimethylnaphthalene (0.0200 mmol, 3.1 mg) and benzene-d6 (1.00 mL) were added into the vial. The mixture was stirred for 2 minutes and then transferred to an NMR tube. The sample was subjected to quantitative ^{1}H NMR analysis multiple times until the ratio of all species became constant.

The reactions in which P(^{n}Bu)$_3$ was used took less than 30 minutes to complete the conversion to the related nicklacyclopropane, while the reactions in which dcype were used took longer to reach an equilibrium. The spectra below were collected two days after the samples were prepared. No decomposition of the nickel complex was observed at that time.

Entry	Coumarin	Ligand	Metallocyclopropane	Time	Yield of M*	Yield of unreacted C*	M: C
1	**1a**	P(^{n}Bu)$_3$	**P(^{n}Bu)$_3$-Ni-1a**	< 30 min	100%	< 5%	> 20: 1
2	**1g**	P(^{n}Bu)$_3$	**P(^{n}Bu)$_3$-Ni-1g**	< 30 min	100%	< 5%	> 20: 1
3	**1m**	P(^{n}Bu)$_3$	**P(^{n}Bu)$_3$-Ni-1m**	< 30 min	100%	< 5%	> 20: 1
4	**1a**	dcype	**dcype-Ni-1a**	2 days	82%	2%	> 20: 1
5	**1g**	dcype	**dcype-Ni-1g**	2 days	29%	61%	1: 2.1
6	**1m**	dcype	**dcype-Ni-1m**	2 days	14%	83%	1: 5.9

*: "**M**" is the abbreviation of "metallocyclopropane"; "**C**" is the abbreviation of "coumarin."

From those experiments, we can conclude that: **1)** P(^{n}Bu)$_3$ ligand is more prone to form the off-cycle nickelacyclopropane than dcype; **2)** 4-H coumarin is easier to undergo olefin oxidative addition than 4-Me and 4-Ph substituted coumarins, while the latter two substrates showed similar reactivities on such reaction.

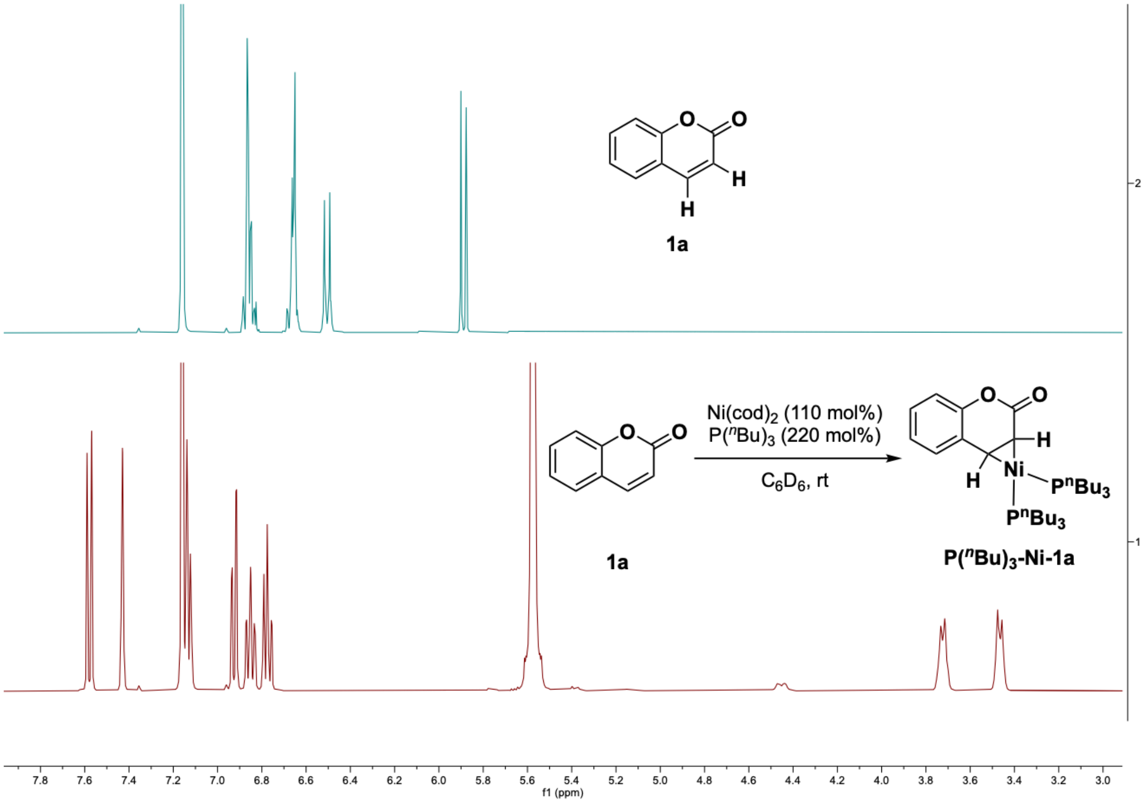
1a
Ni(cod)2 (110 mol%)
P(nBu)3 (220 mol%)
C6D6, rt
P(nBu)3-Ni-1a
f1 (ppm)

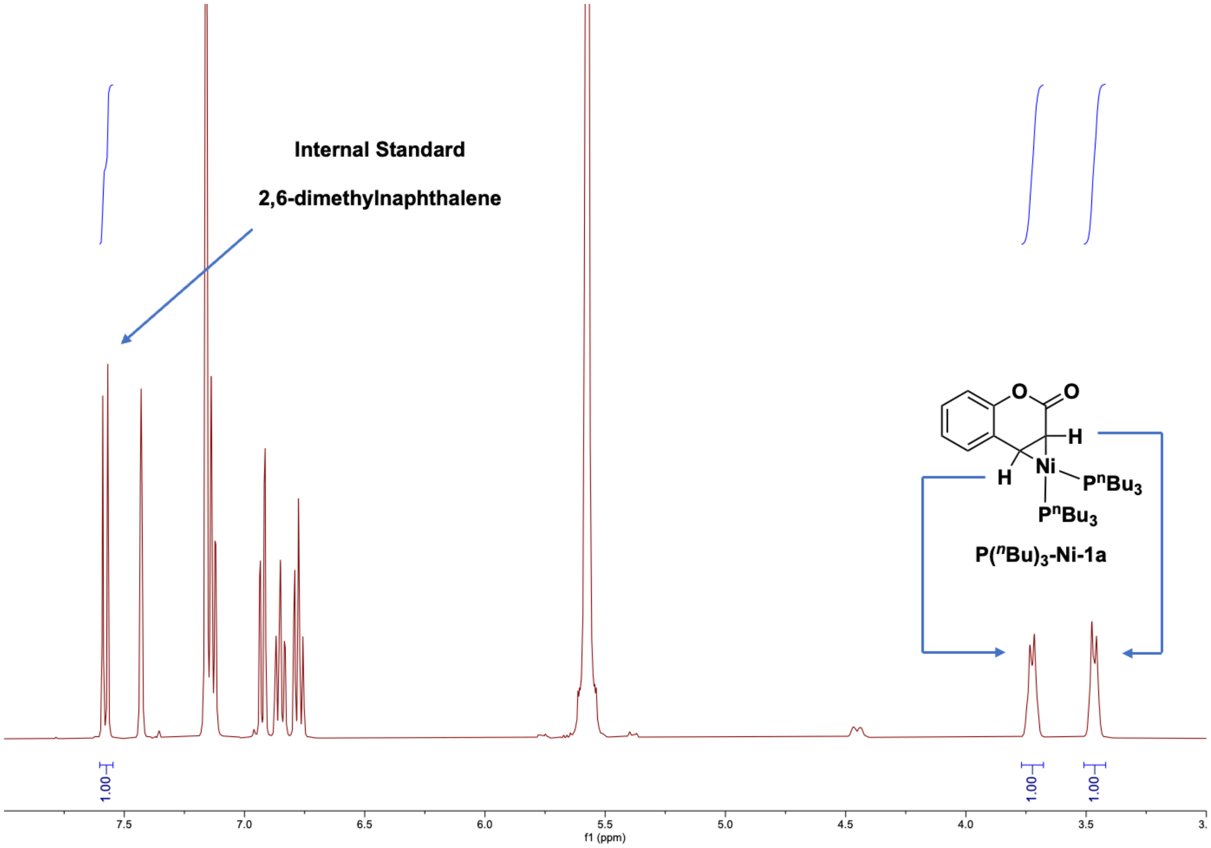
Internal Standard
2,6-dimethylnaphthalene
P(nBu)3-Ni-1a
1.00
1.00
1.00
f1 (ppm)

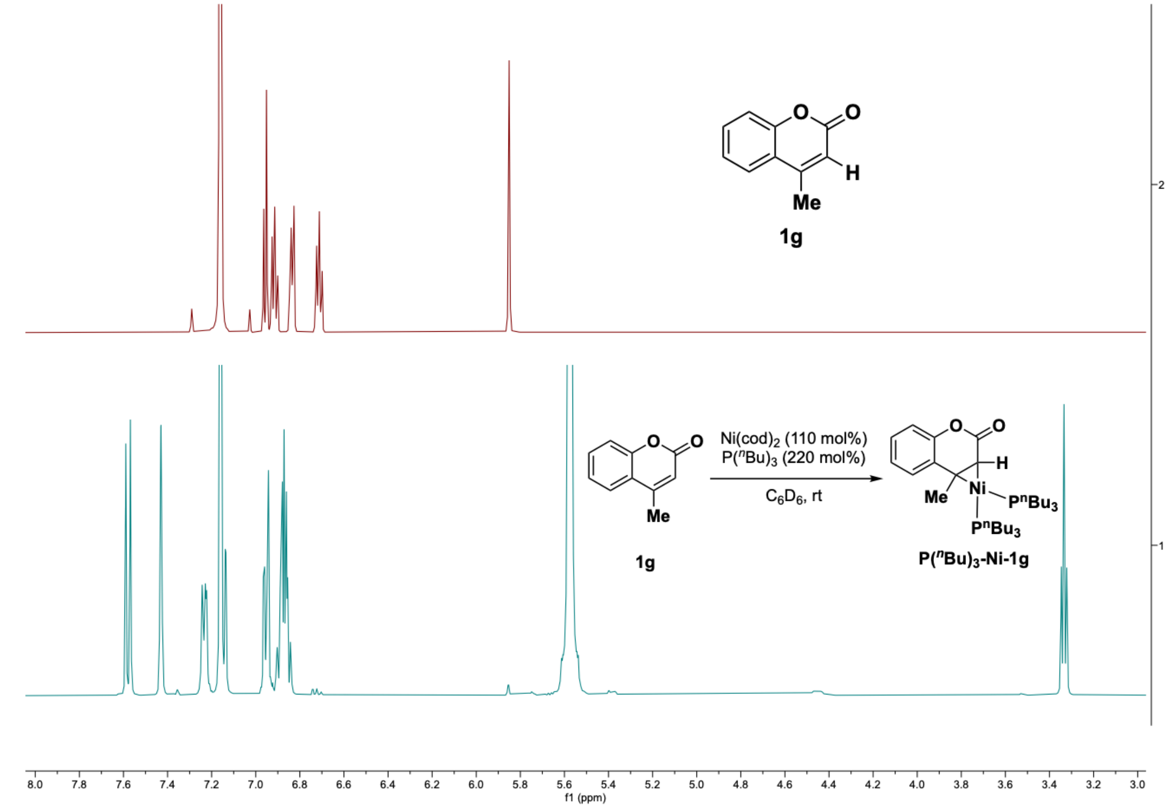
O
O
H
Me
1g
Ni(cod)2 (110 mol%)
P(nBu)3 (220 mol%)
C6D6, rt
Ni
PnBu3
PnBu3
P(nBu)3-Ni-1g
f1 (ppm)

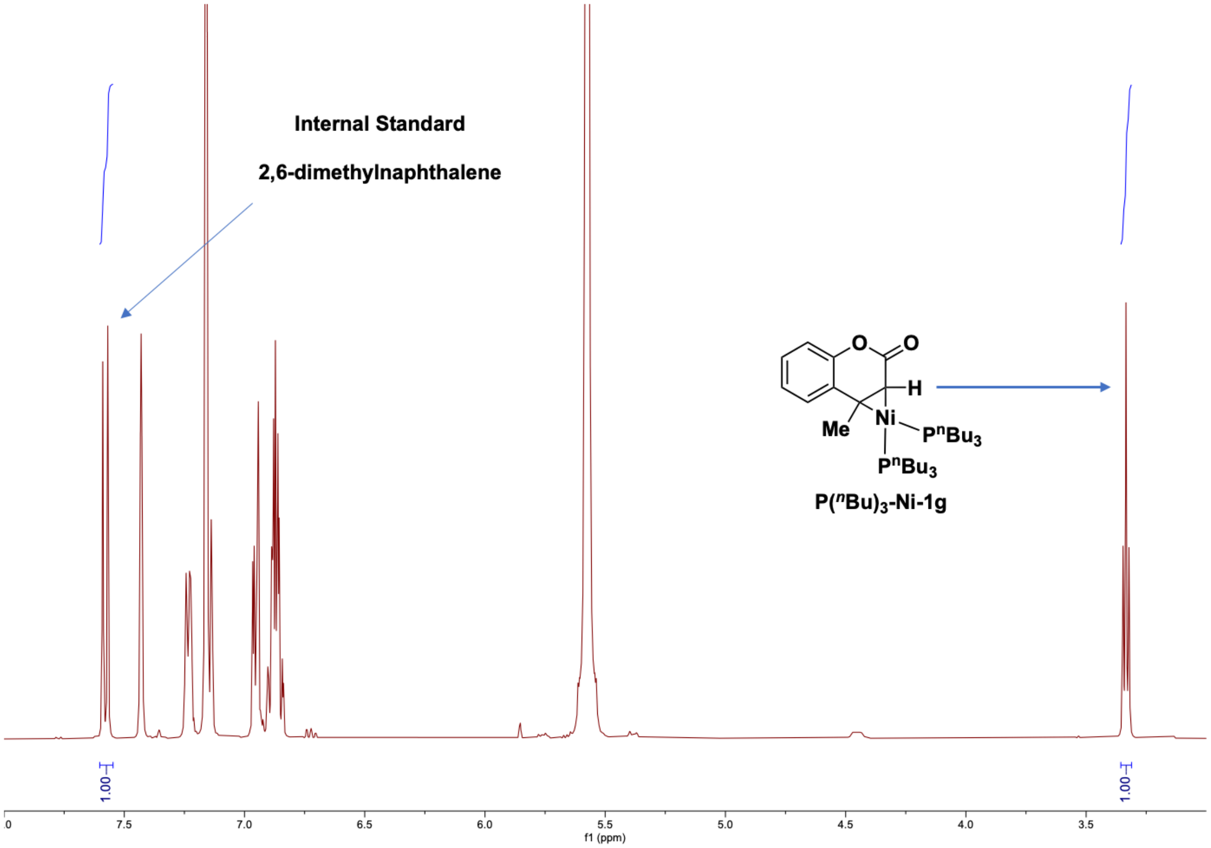
Internal Standard
2,6-dimethylnaphthalene
O
O
H
Me
Ni
PnBu3
PnBu3
P(nBu)3-Ni-1g
1.00
1.00
f1 (ppm)

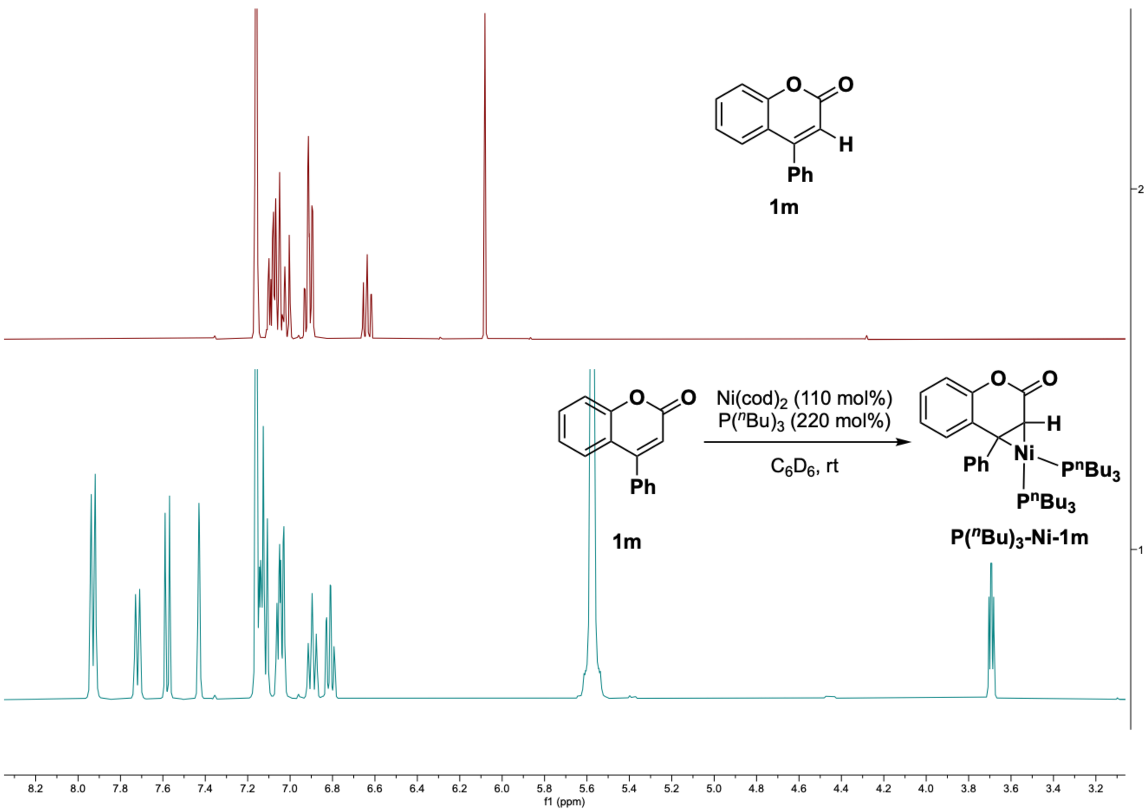

1m
Ni(cod)2 (110 mol%)
P(nBu)3 (220 mol%)
C6D6, rt
P(nBu)3-Ni-1m
f1 (ppm)

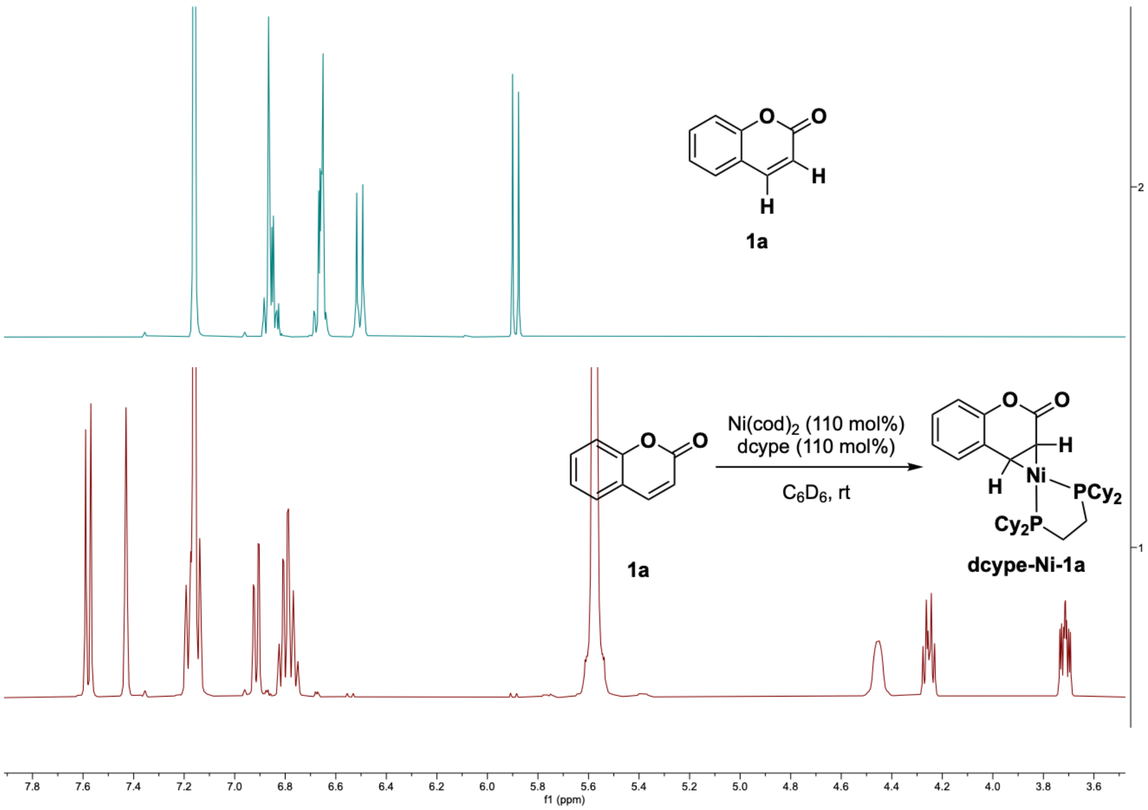

1a
Ni(cod)2 (110 mol%)
dcype (110 mol%)
C6D6, rt
dcype-Ni-1a
f1 (ppm)

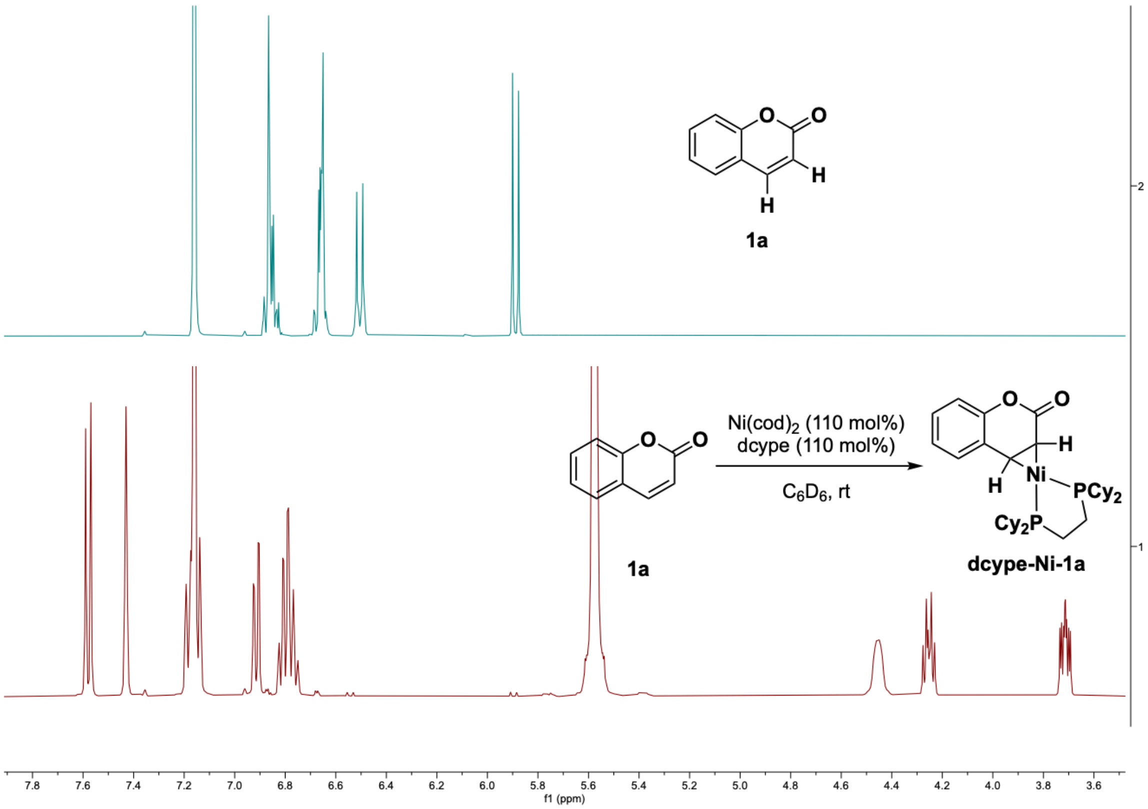
1a
Ni(cod)2 (110 mol%)
dcype (110 mol%)
C6D6, rt
1a
dcype-Ni-1a
f1 (ppm)

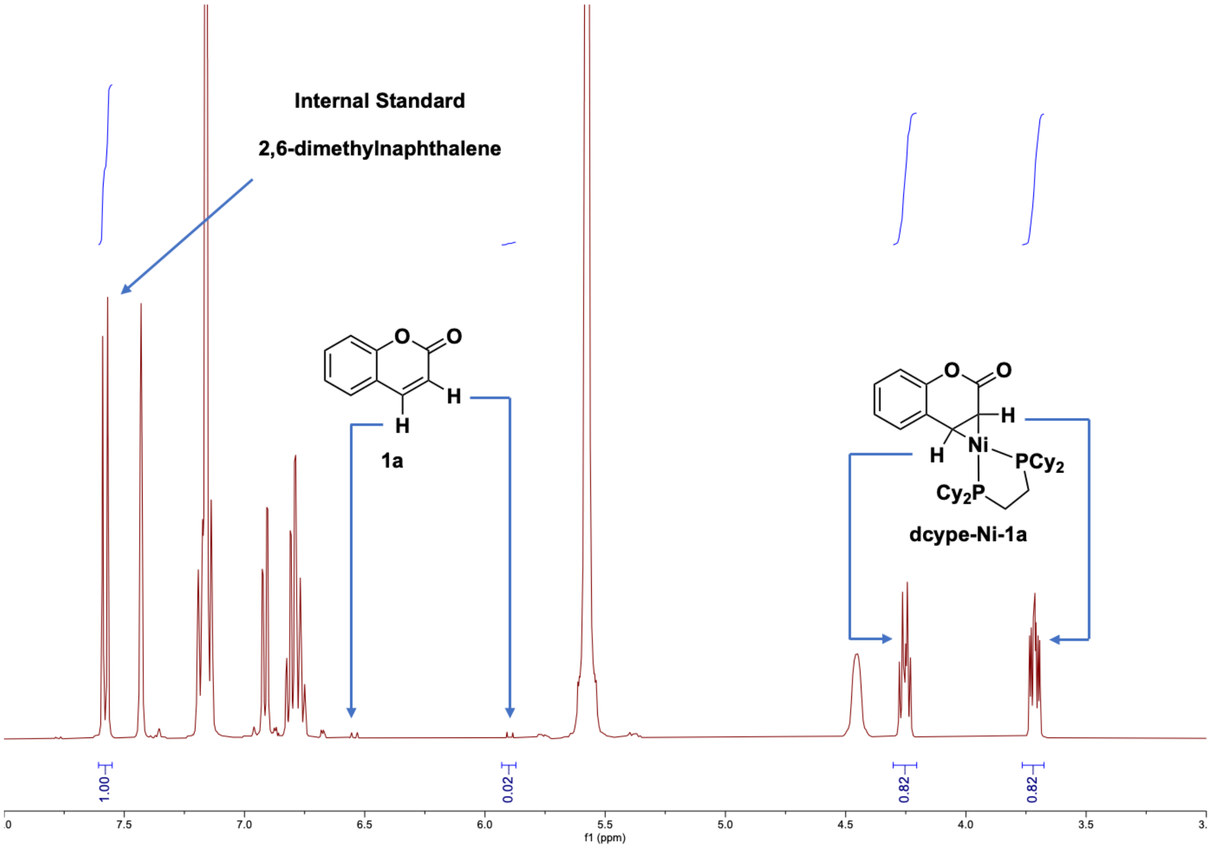
Internal Standard
2,6-dimethylnaphthalene
1a
dcype-Ni-1a
1.00
0.02
0.82
0.82
f1 (ppm)

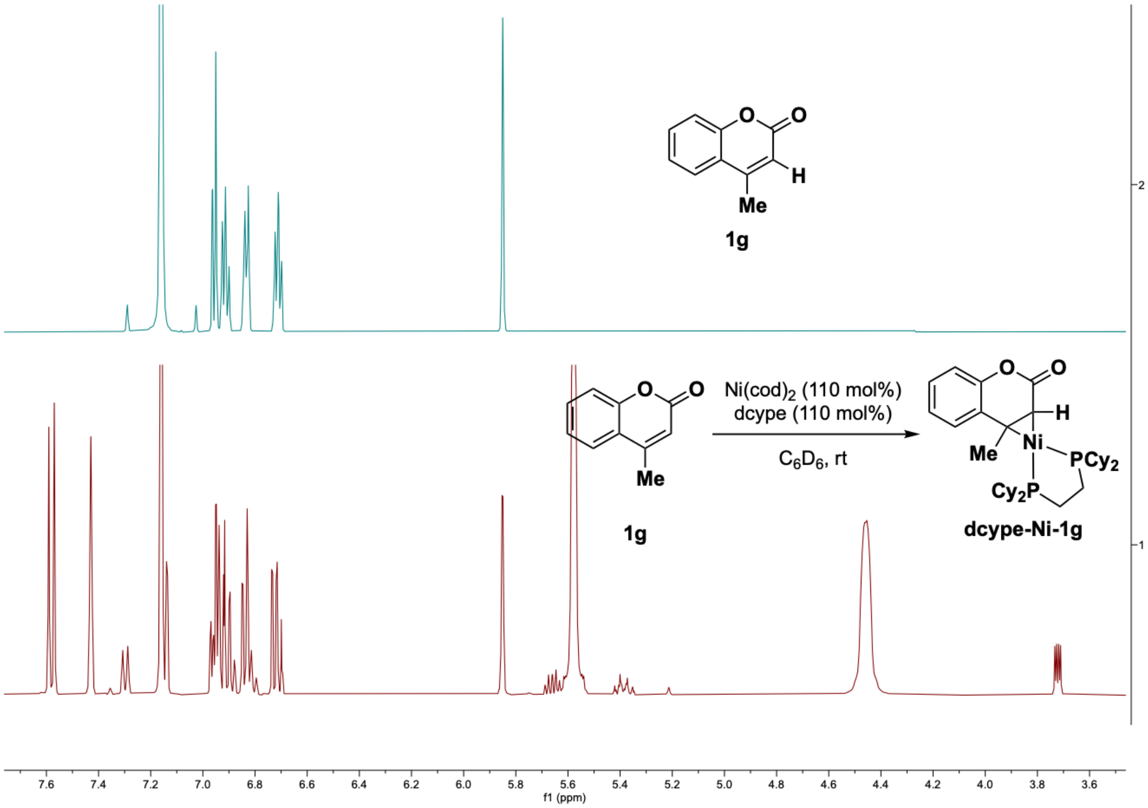

1g
Me
Ni(cod)$_2$ (110 mol%)
dcype (110 mol%)
C_6D_6, rt
dcype-Ni-1g
f1 (ppm)

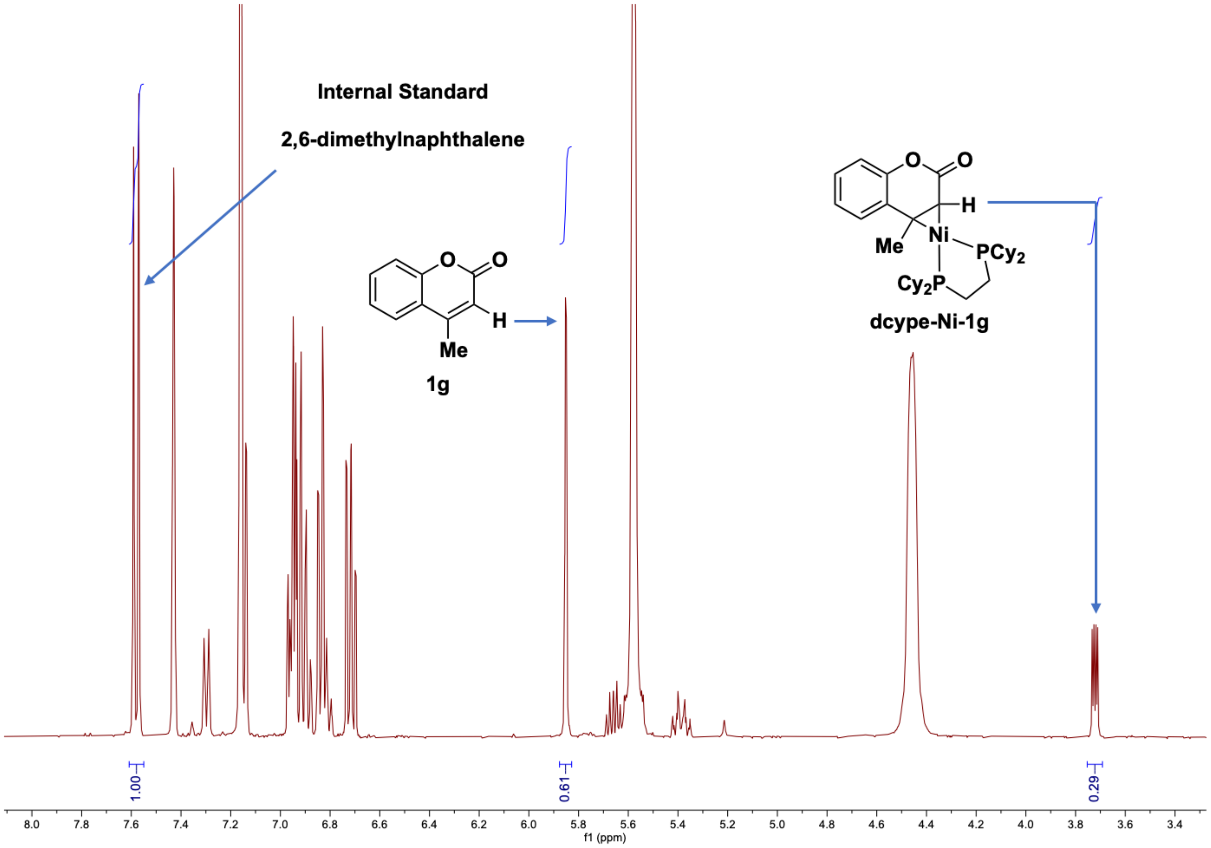

Internal Standard
2,6-dimethylnaphthalene
1g
dcype-Ni-1g
1.00
0.61
0.29
f1 (ppm)

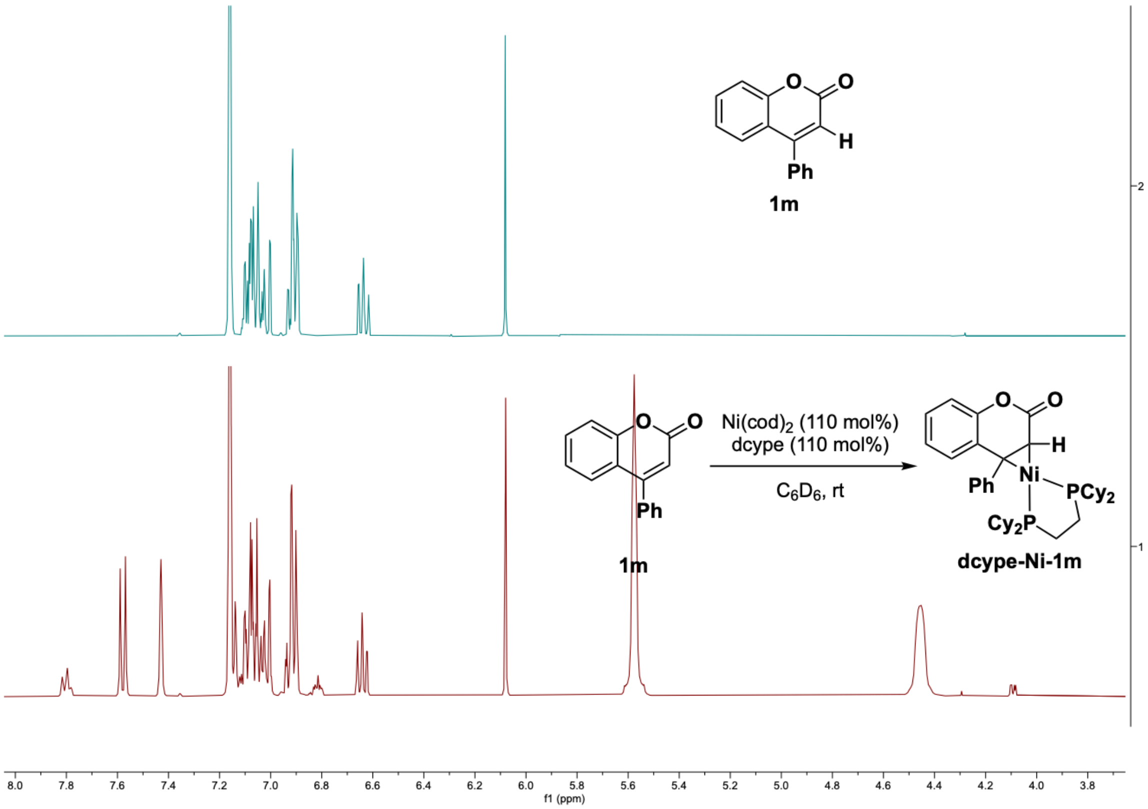
1m
Ni(cod)$_2$ (110 mol%)
dcype (110 mol%)
C_6D_6, rt
dcype-Ni-1m
f1 (ppm)

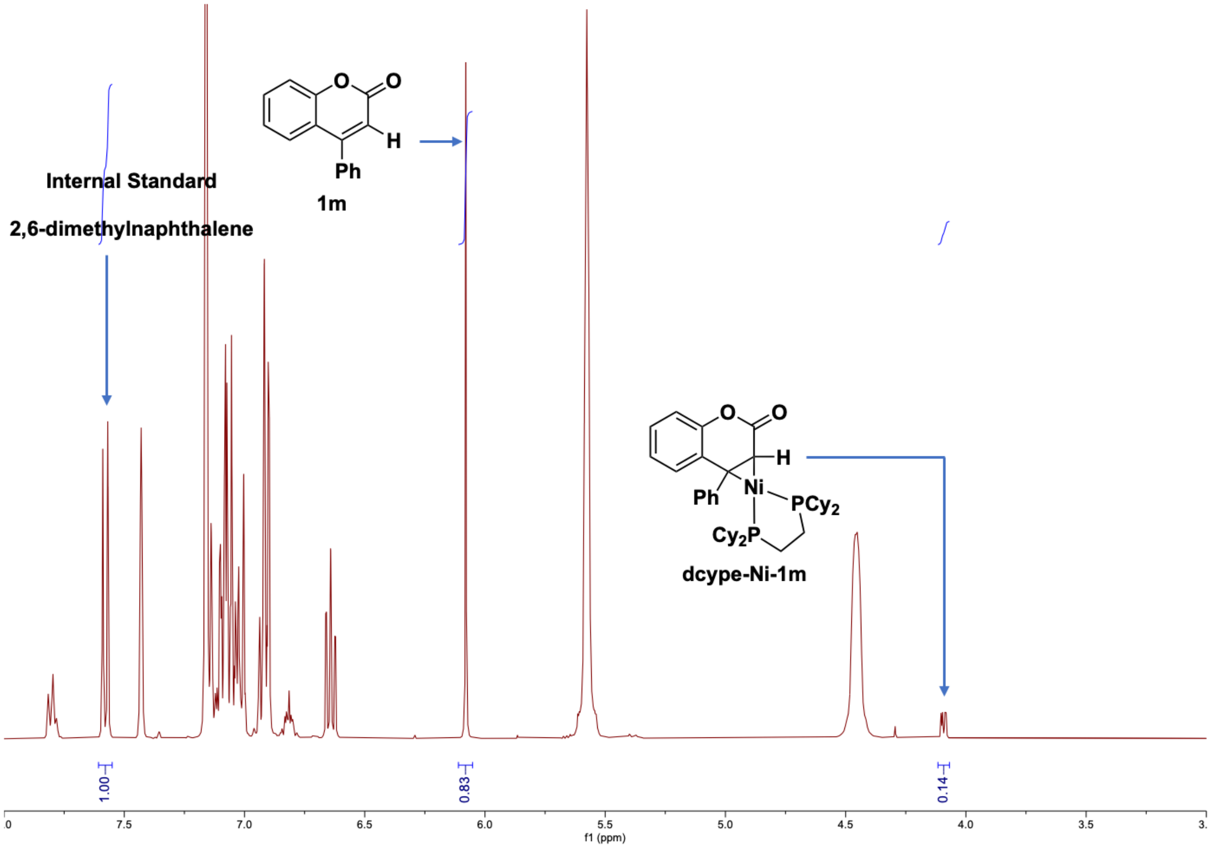
Internal Standard
2,6-dimethylnaphthalene
1m
dcype-Ni-1m
1.00
0.83
0.14
f1 (ppm)

4.2. Additional experiments with benzofuran 3

We performed additional experiments to probe if benzofuran acted as an intermediate in the catalytic decarbonylative borylation. We found that boron insertion into benzofuran or 3-methyl benzofuran using either dcype or $P(^nBu)_3$ as the ligand could occur, but all those reactions gave low yields. In the competition experiments, the benzofuran also exhibited lower reactivity than coumarin. Therefore, we conclude that although benzofuran can undergo borylation under the standard reaction condition, it is unlikely to serve as a major intermediate in the main reaction pathway due to the low reactivity it showed.

Boron insertion into benzofuran: In a glovebox, a 16 × 150 mm reaction tube was charged with a magnetic stir bar, benzofuran **3** (23.6 mg, 22.0 µL, 0.200 mmol) or **4** (26.4 mg, 25.0 µL, 0.200 mmol), $Ni(cod)_2$ (11.0 mg, 0.0400 mmol, 20 mol%), $P(^nBu)_3$ (16.2 mg, 19.8 µL, 0.0800 mmol, 40 mol%) or dcype (16.9 mg, 0.0400 mmol, 20 mol%), B_2eg_2 (34.0 mg, 0.240 mmol, 1.2 equiv), and xylene (1.00 mL) were added. The tube was sealed, brought outside, and placed in an oil bath preheated at 170 °C. The mixture was stirred (700 rpm) for 20 h at 170 °C. After cooling to room temperature, the mixture was directly loaded onto a Combiflash sample cartridge packed with silica gel (5 cm in diameter, 10 cm in length) using a glass pipette. The reaction tube was washed with DCM (0.5 mL × 5) to ensure a complete transfer. The sample was chromatographed by Combiflash using a 12-g RediSep Gold column and ethyl acetate/hexanes mixture as the eluent (0% ethyl acetate for 5 CV, 10% ethyl acetate for another 10 CV, 20% ethyl acetate for 10 CV, 30% ethyl acetate for 10 CV, then 40% ethyl acetate for 10 CV). The product-containing fractions were combined and concentrated. The residue was stored overnight in a well-ventilated fume hood to evaporate the remaining solvents. Drying the product under high vacuum was avoided as some products could sublime. Further purification, if necessary, is described for individual compounds below (Table S7).

Table S7. Boron insertion into benzofuran.

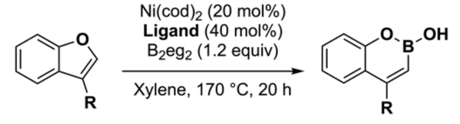

3 (R = H)
4 (R = Me)

2a (R = H)
2g (R = Me)

Entry	substrate	ligand	isolated yield (%)
1	**3**	$P(^nBu)_3$	28
2	**3**	dcype	23
3	**4**	$P(^nBu)_3$	23
4	**4**	dcype	25

Competition experiment: To detect whether there is any preference between coumarin and benzofuran in going through oxidative addition with Ni(0), a competition experiment was carried out as below.

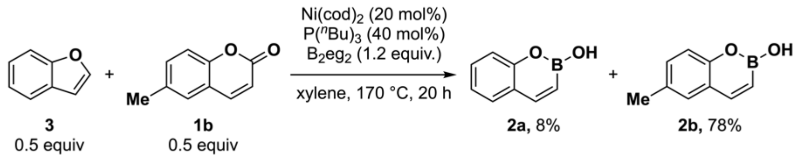

In a glovebox, a 16 × 150 mm reaction tube was charged with a magnetic stir bar, **3** (11.8 mg, 0.100 mmol, 0.5 equiv), **1b** (16.0 mg, 0.100 mmol, 0.5 equiv), Ni(cod)$_2$ (11.0 mg, 0.0400 mmol, 20 mol%), P(^{n}Bu)$_3$ (16.2 mg, 19.8 µL, 0.0800 mmol, 40 mol%), B_2eg_2 (34.0 mg, 0.240 mmol, 1.2 equiv), and xylene (1.00 mL) were added. The tube was sealed, brought outside, and placed in an oil bath preheated at 170 °C. The mixture was stirred (700 rpm) for 20 h at 170 °C. After cooling to room temperature, the mixture was directly loaded onto a Combiflash sample cartridge packed with silica gel (5 cm in diameter, 10 cm in length) using a glass pipette. The reaction tube was washed with DCM (0.5 mL × 5) to ensure a complete transfer. The sample was chromatographed by Combiflash using a 12-g RediSep Gold column and ethyl acetate/hexanes mixture as the eluent (0% ethyl acetate for 5 CV, 10% ethyl acetate for another 10 CV, 15% ethyl acetate for 10 CV, then 20% ethyl acetate for 10 CV). The products started to elute at 15 CV. The products **2a** and **2b** could not be cleanly separated, so the fractions containing those products were combined. The residue was stored overnight in a well-ventilated fume hood to evaporate the remaining solvents. Drying the product under high vacuum was avoided as some products could sublime. An internal standard (dimethyl terephthalate, 9.71 mg, 0.0500 mmol, 0.25 equiv) was added to the product mixture followed byacetone-d6 (0.6 mL). The resulting sample was subjected to quantitative NMR analysis to determine the yield of **2a** and **2b**.

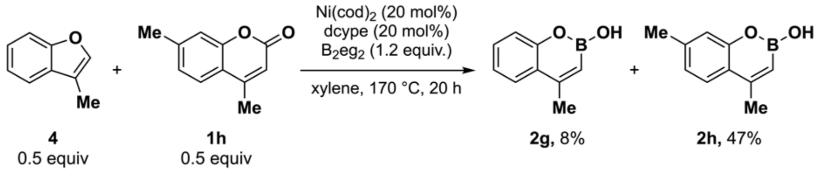

In a glovebox, a 16 × 150 mm reaction tube was charged with a magnetic stir bar, **4** (13.2 mg, 0.100 mmol, 0.5 equiv), **1b** (17.4 mg, 0.100 mmol, 0.5 equiv), Ni(cod)$_2$ (11.0 mg, 0.0400 mmol, 20 mol%), dcype (16.9 mg, 0.0400 mmol, 20 mol%), B_2eg_2 (34.0 mg, 0.240 mmol, 1.2 equiv), and xylene (1.00 mL) were added. The tube was sealed, brought outside, and placed in an oil bath preheated at 170 °C. The mixture was stirred (700 rpm) for 20 h at 170 °C. After cooling to room temperature, the mixture was directly loaded onto a Combiflash sample cartridge packed with silica gel (5 cm in diameter, 10 cm in length) using a glass pipette. The reaction tube was washed with DCM (0.5 mL × 5) to ensure a complete transfer. The sample was chromatographed by Combiflash using a 12-g RediSep Gold column and ethyl acetate/hexanes mixture as the eluent (0% ethyl acetate for 5 CV, 10% ethyl acetate for another 10 CV, 15% ethyl acetate for 10 CV, then 20% ethyl acetate for 10 CV). The product started to elude at 15 CV. The products **2g** and **2h** could not be cleanly separated, so the fractions containing those products were combined. The residue was stored overnight in a well-ventilated fume hood to evaporate the remaining solvents. Drying the product under high vacuum was avoided as some products could sublime. An internal standard (dimethyl terephthalate, 9.71 mg, 0.0500 mmol, 0.25 equiv) was added to the product mixture followed byacetone-d6 (0.6 mL). The resulting samplewas subjected to quantitative NMR analysis to determine the yield of **2g** and **2h**.

The results showed a preference for coumarin in oxidative addition with Ni(0), although the yields of **2b** and **2g** were both slightly lower than their independent substrate screening results. (**Figure 4** in the main text). Unreacted **3** and **4** were seen in these competition experiments.

5. Experimental procedures and characterization data for compounds in Figure 4

5.1. Synthesis of substrates and characterization data for substrates

Substrates **1a**, **1b**, **1c**, **1e**, **1f**, **1g**, and **1h** are commercially available. Literature procedures were used for the syntheses of **1s**[2], **1j** and **1k**,[3] **1l**,[4] **1m-1p**[5], **1q**[6], **1r**[7], and **1u-1y**.[6]

5.1.1. Synthesis of substrate 1d

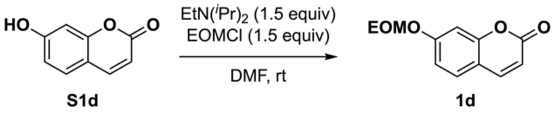

7-(ethoxymethoxy)-2H-chromen-2-one (1d): A round bottom flask was charged with a stir bar, **S1d** (0.324 g, 2.00 mmol), and DMF (3.0 mL). Diisopropylethylamine (0.52 mL, 3.00 mmol, 1.50 equiv) was then added with stirring via syringe followed by chloromethyl ethyl ether (EOMCl, 0.28 mL, 3.00 mmol, 1.50 equiv) at room temperature. The resulting solution was stirred at room temperature for 12 h and was diluted with 100 mL of water. The mixture was transferred to a separatory funnel and was extracted with diethyl ether (5 × 10 mL). The combined organic fractions were washed with water (5 × 30 mL) followed by brine (10 mL) and were dried with Na_2SO_4. The solvent was removed by rotary evaporator to give an off-white solid. The solid was dissolved in DCM (3.0 mL) and loaded onto a Combiflash sample cartridge packed with silica gel (5 × 10 cm). The sample was chromatographed by Combiflash using a 12-g Gold column and hexanes/ethyl acetate mixture as the eluent (0% EtOAc for 7 CV then 20% EtOAc for 30 CV). The product started to elute at 17 CV. The product containing fractions were combined and concentrated by rotary evaporator. The residue was dried under vacuum to give **1d** (0.391 g, 1.77 mmol, 89%) as a white solid.

TLC (80/20 hexanes/ethyl acetate): R_f = 0.20 (visualized by UV)
^{1}H NMR (600 MHz, $CDCl_3$) δ 7.63 (d, *J* = 9.5 Hz, 1H), 7.37 (d, *J* = 8.7 Hz, 1H), 7.01 (d, *J* = 2.5 Hz, 1H), 6.95 (dd, *J* = 8.6, 2.4 Hz, 1H), 6.26 (d, *J* = 9.4 Hz, 1H), 5.27 (s, 2H), 3.73 (q, *J* = 7.1 Hz, 2H), 1.22 (t, *J* = 7.0 Hz, 3H).
^{13}C{^{1}H} NMR (101 MHz, $CDCl_3$) δ 161.2, 160.6, 155.7, 143.4, 128.9, 113.8, 113.7, 113.4, 103.9, 93.3, 64.9, 15.2.
IR (FT-ATR, cm^{-1}, neat) ν_{max} 2984 (m), 2915 (m), 2866 (m), 1711 (vs), 1615 (vs), 1506 (s).
HRMS (ESI) calcd. for $[C_{12}H_{12}O_4+H]^+$: 221.0809, found 221.0900.

5.1.2. Synthesis of substrate 1i

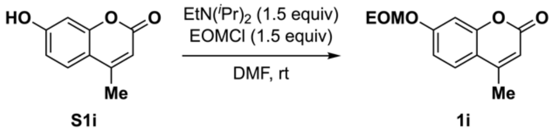

7-(ethoxymethoxy)-4-methyl-2H-chromen-2-one (1i): A round bottom flask was charged with a stir bar, **S1i** (0.352 g, 2.00 mmol), and DMF (3.0 mL). Diisopropylethylamine (0.52 mL, 3.00 mmol, 1.50 equiv) was then added with stirring via syringe followed by EOMCl (0.28 mL, 3.00 mmol, 1.50 equiv) at room temperature. The resulting solution was stirred at room temperature for 12 h and was diluted with 100 mL of water. The mixture was transferred to a separatory funnel and was extracted with diethyl ether (5 × 10 mL). The combined organic fractions were washed with water (5 × 30 mL) followed by brine (10 mL) and were dried with Na_2SO_4. The solvent was removed by rotary evaporator. The residue was dried under vacuum to give **1i** (0.427 g, 1.82 mmol, 91%) as a white solid.

TLC (80/20 hexanes/ethyl acetate): R_f = 0.20 (visualized by UV)

^{1}H NMR (400 MHz, $CDCl_3$) δ 7.50 (d, J = 8.8 Hz, 1H), 7.03 (d, J = 2.5 Hz, 1H), 6.98 (dd, J = 8.8, 2.5 Hz, 1H), 6.16 (s, 1H), 5.28 (s, 2H), 3.73 (q, J = 7.1 Hz, 2H), 2.40 (s, 3H), 1.22 (t, J = 7.1 Hz, 3H).

^{13}C{^{1}H} NMR (101 MHz, $CDCl_3$) δ 161.4, 160.5, 155.2, 152.5, 125.6, 114.5, 113.4, 112.6, 104.0, 93.3, 64.9, 18.8, 15.2

IR (FT-ATR, cm^{-1}, neat) ν_{max} 2976 (w), 2930 (w), 2884 (w), 1719 (s), 1612 (s), 1427 (s), 1385 (s).

HRMS (ESI) calcd. for $C_{13}H_{14}O_4$: 235.0965, found 235.1113.

5.1.3. Synthesis of substrate 1t

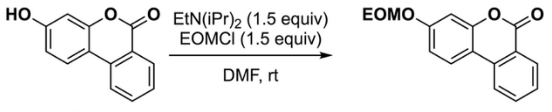

3-(methoxymethoxy)-6H-benzo[c]chromen-6-one (1t): A round bottom flask was charged with a stir bar, **S1t** (0.212 g, 1.00 mmol), and DMF (2.0 mL). Diisopropylethylamine (0.26 mL, 1.50 mmol, 1.50 equiv) was then added with stirring via syringe followed by EOMCl (0.28 mL, 1.50 mmol, 1.50 equiv) at room temperature. The resulting solution was stirred at room temperature for 12 h and was diluted with 100 mL of water. The mixture was transferred to a separatory funnel and was extracted with diethyl ether (5 × 10 mL). The combined organic fractions were washed with water (5 × 20 mL) followed by brine (10 mL) and were dried with Na_2SO_4. The solvent was removed by rotary evaporator to give an off-white solid. The solid was dissolved in DCM (5.0 mL) and loaded onto a Combiflash sample cartridge packed with silica gel (5 ×10 cm). The sample was chromatographed using Combiflash 12-g Gold column and hexanes/ethyl acetate mixture as the eluent (0% EtOAc for 7 CV then 20% EtOAc for 30 CV). The product started to elute at 13 CV. The product containing fractions were combined and concentrated by rotary evaporator. The residue was dried under vacuum to give **1t** (0.231 g, 0.902 mmol, 90%) as a white solid.

TLC (80/20 hexanes/ethyl acetate): R_f = 20 (visualized by UV)

^{1}H NMR (400 MHz, $CDCl_3$) δ 8.34 (dt, J = 8.0, 1.6 Hz, 1H), 7.98 (d, J = 7.1 Hz, 1H), 7.93 (dd, J = 8.8, 1.9 Hz, 1H), 7.77 (ddt, J = 8.4, 7.2, 1.3 Hz, 1H), 7.49 (tt, J = 7.3, 1.2 Hz, 1H), 7.05 (d, J = 2.4 Hz, 1H), 7.01 (dd, J = 8.7, 2.5 Hz, 1H), 5.28 (s, 2H), 3.75 (q, J = 7.1 Hz, 2H), 1.23 (t, J = 7.1 Hz, 3H).

^{13}C{^{1}H} NMR (101 MHz, $CDCl_3$) δ 161.5, 159.3, 152.5, 135.1, 135.0, 130.6, 128.0, 123.8, 121.3, 120.2, 113.6, 112.2, 104.8, 93.3, 64.8, 15.2.

IR (FT-ATR, cm^{-1}, neat) 3295 (w), 3052 (w), 2977 (m), 2918 (w), 2881 (w), 1732 (s), 1697 (w), 1607 (s), 1517 (w), 1479 (m), 1458 (s), 1422 (w), 1342 (w), 1311 (m), 1277 (w), 1258 (s), 1212 (w).
HRMS (ESI) calcd. for $[C_{16}H_{14}O_4+H]^+$: 271.0965, found 271.0970.

5.1.4. Synthesis of B_2eg_2

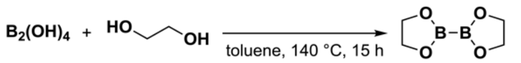

A 100 mL flask was charged with tetrahydroxydiboron (2.24 g, 25.0 mmol) and a magnetic stir bar. Toluene (36 mL) and ethylene glycol (3.03 mg, 2.72 mL, 48.8 mmol) were added to the flask. The flask was connected to a Dean-Stark apparatus and was placed into a 140 °C oil bath. The mixture was heated at 140 °C for 15 h while stirring under ambient atmosphere. The mixture was cooled to room temperature. The solvent was then removed by a rotary evaporator. The solid residue was purified by sublimation under high vacuum (50 mTorr) to give B_2eg_2 (2.89 g, 20.4 mmol, 82%). The product was collected into an oven-dried 20 mL vial and immediately transferred into the glovebox for future storage. NMR data match the literature-reported values.[8]

^{1}H NMR (400 MHz, DMSO-d_6) δ 4.09 (s, 6H).
^{13}C NMR (101 MHz, DMSO-d_6) δ 64.9.
^{11}B NMR (128 MHz, DMSO-d_6) δ 30.4.

A sample of the product (7.1 mg) was mixed with an internal standard (dimethyl terephthalate, 13.0 mg, 0.0667 mmol) in a 2-dram vial with a stir bar in the glovebox. Dry DMSO-d_6 (0.7 mL) was added to the vial, and the vial was sealed, brought outside the glovebox, and placed on a 60 °C heating block for 3-5 minutes until the dissolution was complete. The mixture was moved back to the glovebox, transferred into an NMR tube and then subjected to quantitative NMR analysis to show >98% purity.

Note: All experiments involving B_2eg_2 in this report were carried out with material having >98% purity (determined by quantitative NMR as described above). Lower purity leads to inconsistent results. .

5.2. General procedure and characterization data for decarbonylative borylation products
General procedure: An oven-dried 16 x 150 mm reaction tube was charged with a magnetic stir bar, a coumarin or a lactone **1** (0.200 mmol). The tube was brought into a glovebox, then $Ni(cod)_2$ (11.0 mg, 0.040 mmol, 20 mol%), P^nBu_3 (16.2 mg, 0.080 mmol, 40 mol%) or dcype (16.9 mg, 0.040 mmol, 20 mol%), B_2eg_2 (34.0 mg, 0.240 mmol, 1.2 equiv), and xylene (1.00 mL) were added. The vial was sealed and brought outside and placed in an oil bath preheated at 170 °C. The mixture was stirred (700 rpm) for 20 h at 170 °C. After cooling to room temperature, the mixture was directly loaded onto a Combiflash sample cartridge packed with silica gel (5 × 10 cm). The sample was chromatographed by Combiflash using a 12-g RediSep Gold column and ethyl acetate/hexanes mixture as the eluent. The product-containing fractions were combined and concentrated. Further purification, if necessary, is described for the individual compound below.

Notes about the procedure:

- The amount of silica gel in the sample loading cartridge stated in **general procedure A** is essential to get the pure product. Lower amounts of silica gel gave lower yields and purities.
- The UV absorbance of some 7-membered dibenzoxaborepin is significantly lower than the starting material.
- The ^{1}H NMR (spectra) of most products taken in CD_2Cl_2 showed an unidentified species, which are likely boroxines arising from the dimerization or trimerization of the oxaborines.

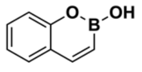

2a

2H-benzo[e][1,2]oxaborinin-2-ol (2a): Compound **2a** was obtained as a white solid from **1a** (29.2 mg, 0.200 mmol). General Procedure A: 24.7 mg of **2a**, 0.169 mmol, 85%; General Procedure B: 9.64 mg of **2a**, 0.0661 mmol, 33%. NMR data match previously reported values.[1]

Column condition: 0% ethyl acetate for 5 CV, 10% ethyl acetate for 10 CV, 20% ethyl acetate for 10 CV, 25% ethyl acetate for 10 CV, then 30% ethyl acetate for 5 CV. The product started to elute at 25 CV.

TLC (80/20 hexanes/ethyl acetate): R_f = 0.20 (visualized by UV)
^{1}H NMR (400 MHz, $CDCl_3$) δ 8.61 (br s, 1H), 7.78 (d, *J* = 11.8 Hz, 1H), 7.41 (dd, *J* = 7.6, 1.7 Hz, 1H), 7.39 – 7.35 (m, 1H), 7.27 – 7.23 (m, 1H), 7.15 (td, *J* = 7.4, 1.2 Hz, 1H), 6.23 (d, *J* = 11.8 Hz, 1H), 4.77 (br s, 1H).
^{13}C{^{1}H} NMR (101 MHz, Acetone-d_6) δ 153.6, 149.5, 130.1, 129.6, 125.4, 122.8, 119.1.
^{11}B NMR (128 MHz, Acetone-d_6) δ 27.6.

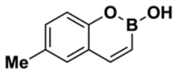

2b

6-methyl-2H-benzo[e][1,2]oxaborinin-2-ol (2b): Compound **2b** was obtained as a white solid from **1b** (32.0 mg, 0.200 mmol). General Procedure A: 29.8 mg of **2b**, 0.186 mmol, 93%; General Procedure B: 8.31 mg of **2b**, 0.0519 mmol, 26%.

Column condition: 0% ethyl acetate for 5 CV, 10% ethyl acetate for 10 CV, 20% ethyl acetate for 10 CV, 25% ethyl acetate for 10 CV, then 30% ethyl acetate for 5 CV. The product started to elute at 25 CV.

TLC (80/20 hexanes/ethyl acetate): R_f = 0.40 (visualized by UV)
^{1}H NMR (400 MHz, Acetone-d_6) δ 7.76 (d, *J* = 11.8 Hz, 1H), 7.27 (d, *J* = 2.0 Hz, 1H), 7.20 (dd, *J* = 8.3, 2.2 Hz, 1H), 7.09 (d, *J* = 8.3 Hz, 1H), 6.15 (d, *J* = 11.8 Hz, 1H), 2.33 (s, 3H).
^{13}C{^{1}H} NMR (101 MHz, CD_2Cl_2) δ 150.8, 149.7, 132.1, 130.7, 129.1, 124.6, 118.3, 20.8.
^{11}B NMR (193 MHz, CD_2Cl_2) δ 27.7.
IR (FT-ATR, cm^{-1}, neat) ν_{max} 3333 (br), 2957 (m), 2922 (m), 1599 (vs), 1588 (vs), 1444 (s), 1327 (vs), 1275 (vs), 1241 (vs).
HRMS (ESI) calcd. for $[C_9H_9BO_2–H]^–$: 159.0623, found 159.0621.

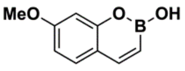

2c

7-methoxy-2H-benzo[e][1,2]oxaborinin-2-ol (2c): Compound **2c** was obtained as a white solid from **1c** (35.2 mg, 0.200 mmol). General Procedure A: 29.4 mg of **2c**, 0.167 mmol, 84%; General Procedure B: 3.55 mg of **2c**, 0.0202 mmol, 10%.

Column condition: 0% ethyl acetate for 5 CV, 10% ethyl acetate for 10 CV, 20% ethyl acetate for 10 CV, 25% ethyl acetate for 10 CV, then 30% ethyl acetate for 5 CV. The product started to elute at 25 CV.

TLC (80/20 hexanes/ethyl acetate): $R_f = 0.40$ (visualized by UV)
^{1}H NMR (400 MHz, Acetone-d_6) δ 7.74 (br s overlapping with d, J = 11.9 Hz, 1H), 7.40 – 7.35 (m, 1H), 6.77 – 6.71 (m, 2H), 5.98 (d, J = 11.8 Hz, 1H), 3.84 (s, 3H).
^{13}C{^{1}H} NMR (101 MHz, Acetone-d_6) δ 162.0, 155.1, 149.3, 130.3, 119.0, 110.2, 103.6, 55.8.
^{11}B NMR (193 MHz, Acetone-d_6) δ 27.8
IR (FT-ATR, cm^{-1}, neat) ν_{max} 3402 (br), 3232 (m), 2959 (m), 2930 (m), 1619 (vs), 1599 (vs), 1505 (s), 1439 9(s), 1328 (s), 1265 (vs), 1219 (vs).
HRMS (ESI) calcd. for $[C_9H_9BO_3–H]^-$: 175.0571, found 175.0607.

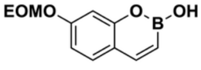

2d

7-(ethoxymethoxy)-2H-benzo[e][1,2]oxaborinin-2-ol (2d): Compound **2d** was obtained as a white solid from **1d** (44.0 mg, 0.200 mmol). General Procedure A: 30.0 mg of **2d**, 0.135 mmol, 68%; General Procedure B: 2.29 mg of **2d**, 0.0104 mmol, 10%.

Column condition: 0% ethyl acetate for 5 CV, 10% ethyl acetate for 10 CV, 20% ethyl acetate for 10 CV, 25% ethyl acetate for 10 CV, then 30% ethyl acetate for 5 CV. The product started to elute at 25 CV.

TLC (80/20 hexanes/ethyl acetate): $R_f = 0.20$ (visualized by UV)
^{1}H NMR (400 MHz, Acetone-d_6) δ 7.82 – 7.75 (br s overlapped with d, J = 11.8 Hz, 2H), 7.39 (d, J = 8.5 Hz, 1H), 6.89 (d, J = 2.6 Hz, 1H), 6.84 (dd, J = 8.5, 2.5 Hz, 1H), 6.00 (d, J = 11.8 Hz, 1H), 5.29 (s, 2H), 3.72 (q, J = 7.1 Hz, 2H), 1.17 (t, J = 7.1 Hz, 3H).
^{13}C{^{1}H} NMR (101 MHz, CD_2Cl_2) δ 159.0, 153.9, 149.4, 129.7, 119.4, 111.5, 105.9, 93.5, 64.8, 15.3.
^{11}B NMR (128 MHz, CD_2Cl_2) δ 27.8
IR (FT-ATR, cm^{-1}, neat) ν_{max} 3365 (br), 3225 (m), 2975 (s), 2924 9s), 2855 (m), 1619 (vs), 1600 (vs), 1502 (s), 1330 (vs).
HRMS (ESI) calcd. for $[C_{11}H_{13}BO_4–H]^-$: 219.0834, found 219.0843.

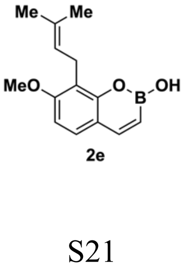

2e

7-methoxy-8-(3-methylbut-2-en-1-yl)-2H-benzo[e][1,2]oxaborinin-2-ol (2e): Compound **2e** was obtained as a colorless solid from **1e** (48.8 mg, 0.200 mmol). General Procedure A: 19.8 mg of **2e**, 0.0811 mmol, 41%; General Procedure B: 13.2 mg of **2e**, 0.0542 mmol, 27%.

Column condition: 0% ethyl acetate for 5 CV, 5% ethyl acetate for 10 CV, 10% ethyl acetate for 10 CV, 15% ethyl acetate for 10 CV, then 20% ethyl acetate for 5 CV. The product started to elute at 15 CV.

TLC (80/20 hexanes/ethyl acetate): R_f = 0.25 (visualized by UV)
^{1}H NMR (400 MHz, Acetone-d_6) δ 7.77 – 7.70 (br s overlapped with d, *J* = 11.5 Hz, 2H), 7.29 (d, *J* = 8.5 Hz, 1H), 6.83 (d, *J* = 8.6 Hz, 1H), 5.97 (d, *J* = 11.8 Hz, 1H), 5.24 (apparent tp, *J* = 7.4, 1.5 Hz, 1H), 3.88 (s, 3H), 3.51 (d, *J* = 7.3 Hz, 2H), 1.80 (d, *J* = 1.3 Hz, 3H), 1.62 (d, *J* = 1.4 Hz, 3H).
^{13}C{^{1}H} NMR (101 MHz, Acetone-d_6) δ 159.3, 152.1, 149.9, 131.5, 127.6, 123.6, 119.4, 119.2, 106.1, 56.2, 25.9, 22.8, 18.0.
^{11}B NMR (128 MHz, Acetone-d_6) δ 27.9
IR (FT-ATR, cm^{-1}, neat) ν_{max} 3452 (br), 2924 (vs), 2854 (s), 1605 (s), 1554 (s), 1501 (s), 1450 (s), 1376 (s), 1306 (vs).
HRMS (ESI) calcd. for $[C_{14}H_{17}BO_3+H]^+$: 243.1197, found 243.1265.

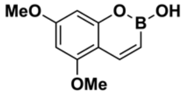

2f

5,7-dimethoxy-4-methyl-2H-benzo[e][1,2]oxaborinin-2-ol (2f): Compound **2f** was obtained as a colorless solid from **1f** (41.2 mg, 0.200 mmol). General Procedure A: 15.7 mg of **2f**, 0.0762 mmol, 38%. General Procedure B: 19.3 mg of **2f**, 0.0937 mmol, 47%.

Column condition: 0% ethyl acetate for 5 CV, 10% ethyl acetate for 10 CV, 20% ethyl acetate for 10 CV, 25% ethyl acetate for 10 CV, then 30% ethyl acetate for 5 CV. The product started to elute at 20 CV.

TLC (80/20 hexanes/ethyl acetate): R_f = 0.10 (visualized by UV)
^{1}H NMR (400 MHz, Acetone-d_6) δ 8.02 (d, *J* = 12.1 Hz, 1H), 7.68 (br s, 1H), 6.37 (d, *J* = 2.2 Hz, 1H), 6.34 (d, *J* = 2.3 Hz, 1H), 5.90 (d, *J* = 12.2 Hz, 1H), 3.89 (s, 3H), 3.84 (s, 3H).
^{13}C{^{1}H} NMR (101 MHz, Acetone-d_6) δ 162.6, 158.1, 155.8, 143.4, 109.2, 95.9, 93.6, 56.2, 55.8.
^{11}B NMR (193 MHz, Acetone-d_6) δ 27.9
IR (FT-ATR, cm^{-1}, neat) ν_{max} 3377 (br), 2940 (m), 2840 (m), 1600 (vs), 1411 (s), 1323 (s), 1204 (s), 1140 (s).
HRMS (ESI) calcd. for $[C_{10}H_{11}BO_4–H]^-$: 205.0678, found 205.0688.

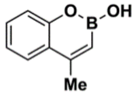

2g

4-methyl-2H-benzo[e][1,2]oxaborinin-2-ol (2g): Compound **2g** was obtained as a white solid from **1h** (32.0 mg, 0.200 mmol). General Procedure A: The amount of product is too low to be isolated. General Procedure B: 19.5 mg of **2g**, 0.122 mmol, 61%.

Column condition: 0% ethyl acetate for 5 CV, 10% ethyl acetate for 10 CV, 15% ethyl acetate for 10 CV, 20% ethyl acetate for 10 CV, then 25% ethyl acetate for 5 CV. The product started to elute at 20 CV.

TLC (80/20 hexanes/ethyl acetate): $R_f = 0.20$ (visualized by UV).
^{1}H NMR (600 MHz, Acetone-d_6) δ 7.68 (s, 1H), 7.64 (dd, *J* = 7.9, 2.3 Hz, 1H), 7.39 (tt, *J* = 7.2, 1.5 Hz, 1H), 7.21 (dd, *J* = 8.2, 1.3 Hz, 1H), 7.16 (tt, *J* = 7.2, 1.3 Hz, 1H), 6.00 (s, 1H), 2.41 (s, 3H).
^{13}C{^{1}H} NMR (101 MHz, Acetone-d_6) δ 156.1, 153.8, 130.1, 126.1, 125.9, 122.7, 119.4, 22.2.
^{11}B NMR (128 MHz, Acetone-d_6) δ 27.6.
IR (FT-ATR, cm^{-1}, neat) 3205 (br s), 2972 (w), 1717 (w), 1606 (s), 1558 (m), 1483 (w), 1445 (w), 1387 (w), 1371 (w), 1329 (s), 1276 (w), 1242 (w), 1202 (w).
HRMS (ESI) calcd. for $[C_9H_9BO_2+H]^+$: 161.0768, found 161.0765.

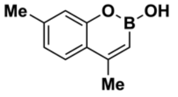

2h

4,7-dimethyl-2H-benzo[e][1,2]oxaborinin-2-ol (2h): Compound **2g** was obtained as a white solid from **1g** (32.0 mg, 0.200 mmol). General Procedure A: 11.1 mg of **2g**, 0.0638 mmol, 32%. General Procedure B: 19.2 mg of **2g**, 0.110 mmol, 55%.

Column condition: 0% ethyl acetate for 5 CV, 10% ethyl acetate for 10 CV, 15% ethyl acetate for 10 CV, 20% ethyl acetate for 10 CV, then 25% ethyl acetate for 5 CV. The product started to elute at 20 CV.

TLC (80/20 hexanes/ethyl acetate): $R_f = 0.20$ (visualized by UV).
^{1}H NMR (400 MHz, Acetone-d_6) δ 7.63 (s, 1H), 7.50 (d, *J* = 8.0 Hz, 1H), 7.03 (d, *J* = 1.7 Hz, 1H), 6.98 (dd, *J* = 8.0, 1.7 Hz, 1H), 5.91 (s, 1H), 2.37 (2 x s, 6H).
^{13}C{^{1}H} NMR (101 MHz, Acetone-d_6) δ 156.0, 153.8, 140.4, 125.9, 123.7, 123.4, 119.7, 22.1, 21.2.
^{11}B NMR (128 MHz, Acetone-d_6) δ 27.6.
IR (FT-ATR, cm^{-1}, neat) 3204 (br s), 2922 (w), 1603 (m), 1547 (w), 1506 (w), 1326 (s), 1185 (w).
HRMS (ESI) calcd. for $[C_{10}H_{11}BO_2+H]^+$: 175.0925, found 175.0927.

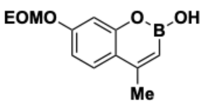

2i

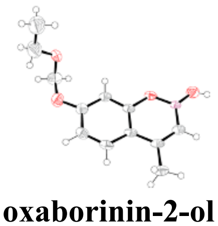

7-(ethoxymethoxy)-4-methyl-2H-benzo[e][1,2]oxaborinin-2-ol (2i): Compound **2i** was obtained from **1i** (46.8 mg, 0.200 mmol). General Procedure A: The amount of product is too low to be isolated. General Procedure B: 27.3 mg of **2i**, 0.117 mmol, 58%.

Column condition: 0% ethyl acetate for 5 CV, 10% ethyl acetate for 10 CV, 20% ethyl acetate for 10 CV, 25% ethyl acetate for 10 CV, then 30% ethyl acetate for 5 CV. The product started to elute at 25 CV.

TLC (80/20 hexanes/ethyl acetate): R_f = 0.20 (visualized by UV)
^{1}H NMR (400 MHz, Acetone-d_6) δ 7.59 (br s, 1H), 7.55 (d, J = 8.6 Hz, 1H), 6.88 (d, J = 2.6 Hz, 1H), 6.86 (dd, J = 8.6, 2.5 Hz, 1H), 5.84 (q, J = 1.4 Hz, 1H), 5.30 (s, 2H), 3.72 (q, J = 7.1 Hz, 2H), 2.37 (dd, J = 1.3, 0.6 Hz, 3H), 1.17 (t, J = 7.1 Hz, 3H).
^{13}C{^{1}H} NMR (101 MHz, Acetone-d_6) δ 159.5, 156.0, 155.0, 126.9, 120.3, 111.5, 106.4, 93.8, 64.9, 22.2, 15.5.
^{11}B NMR (193 MHz, Acetone-d_6) δ 27.8.
IR (FT-ATR, cm^{-1}, neat) ν_{max} 3380 (br), 3240 (s), 2977 (s), 2919 (s), 1605 (vs), 1551 (s), 1501 (s), 1388 (s), 1335 (vs), 1277 (s).
HRMS (ESI) calcd. for $[C_{12}H_{15}BO_4\text{–}H]^-$: 233.0990, found 233.0976.
Crystallography. A 1-dram vial was charged with **2i** (20 mg), and a mixture of ethyl acetate/hexanes (20/80, 1.0 mL). The mixture was stirred until **2i** was completely dissolved. The stir bar was removed and the solution was let sit at room temperature (open cap). Crystals appeared after 48 h. A colorless plate specimen of the crystals, approximate dimensions 0.700 mm × 0.220 mm × 0.190 mm was used for the X-ray crystallographic analysis. Crystal suitable for X--ray structure determination was selected under the polarizing microscope, covered with Paratone oil and mounted on a goniometer head using Mitegen cryoloop. Experiment was performed at the low temperature. QUINN software was used to calculate optimal data collection strategy. Data were collected till resolution of 0.81 Å and were truncated with XPREP till actual observed resolution. The X-ray intensity data were measured on a Bruker D8 VENTURE diffractometer system equipped with a microfocus sealed tube (Cu Kα, λ = 1.54178 Å) and a multilayer mirror monochromator. Additional details are provided in Table S9 and are available free of charge from the Cambridge Crystallographic Data Center, CCDC 2211749.

Table S8. Crystal and structural data for product 2i

**Chemical formula**	$C_{12}H_{15}BO_4$	
**Formula weight**	234.05 g/mol	
**Temperature**	143(2) K	
**Wavelength**	1.54178 Å	
**Crystal size**	0.700 × 0.220 × 0.190 mm	
**Crystal habit**	colorless plate	
**Crystal system**	triclinic	
**Space group**	P –1	
**Unit cell dimensions**	a = 6.8119(3) Å	α = 76.094(3)°
	b = 7.3837(3) Å	β = 76.182(3)°
	c = 12.3435(5) Å	γ = 76.106(3)°
**Volume**	574.10(4) Å^3	
**Z**	2	
**Density (calculated)**	1.354 g/cm^3	
**Absorption coefficient**	0.817 mm^{-1}	
**F(000)**	248	
**Diffractometer**	Bruker D8 VENTURE diffractometer	

**Radiation source**	sealed tube microfocus sealed tube (Cu Kα, λ = 1.54178 Å)
**Theta range for data collection**	3.76 to 63.69°
**Index ranges**	-7<=h<=6, -8<=k<=8, -14<=l<=14
**Reflections collected**	6369
**Independent reflections**	1853 [R(int) = 0.0467]
**Coverage of independent reflections**	100.0%
**Absorption correction**	Multi-Scan
**Max. and min. transmission**	0.860 and 0.570
**Structure solution technique**	direct methods
**Structure solution program**	SHELXT 2018/2 (Sheldrick, 2018)
**Refinement method**	Full-matrix least-squares on F2
**Refinement program**	SHELXL-2018/3 (Sheldrick, 2018)
**Function minimized**	Σ w(Fo2 - Fc2)2
**Data / restraints / parameters**	1853 / 150 / 199
**Goodness-of-fit on F2**	0.922
**Δ/σmax**	0.002
**Final R indices**	1583 data; I>2σ(I) R1 = 0.0599, wR2 = 0.1777
	all data R1 = 0.0667, wR2 = 0.1879
**Weighting scheme**	$w=1/[\sigma^2(F_o^2)+(0.0420P)^2+0.0200P]$ where $P=(F_o^2+2F_c^2)/3$
**Largest diff. peak and hole**	0.506 and -0.295 eÅ^{-3}
**R.M.S. deviation from mean**	0.000 eÅ^{-3}

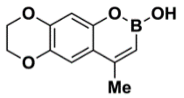

2j

4-methyl-7,8-dihydro-2H-[1,4]dioxino[2',3':4,5]benzo[1,2-e][1,2]oxaborinin-2-ol (2j): Compound **2j** was obtained as a colorless solid from **1j** (43.6 mg, 0.200 mmol). General Procedure A: The amount of product is too low to be isolated. General Procedure B: 31.0 mg of **2j**, 0.142 mmol, 71%.

Column condition: 0% ethyl acetate for 5 CV, 10% ethyl acetate for 10 CV, 20% ethyl acetate for 10 CV, 25% ethyl acetate for 10 CV, then 30% ethyl acetate for 5 CV. The product started to elute at 25 CV.

TLC (80/20 hexanes/ethyl acetate): R_f = 0.15 (visualized by UV)
^{1}H NMR (400 MHz, Acetone-d_6) δ 7.03 (s, 1H), 6.65 (s, 1H), 5.83 (s, 1H), 4.32 – 4.28 (m, 2H), 4.28 – 4.24 (m, 2H), 2.32 (s, 3H).
^{13}C{^{1}H} NMR (101 MHz, Acetone-d_6) δ 155.7, 148.6, 145.7, 139.7, 119.8, 113.2, 107.0, 65.6, 65.0, 22.3.
^{11}B NMR (128 MHz, Acetone-d_6) δ 27.7

IR (FT-ATR, cm^{-1}, neat) ν_{max} 3339 (br), 2974 (s), 2927 (s), 2875 (s), 1607 (s), 1554 (vs), 1500 (vs), 1341 (vs), 1302 (vs).
HRMS (ESI) calcd. for $[C_{11}H_{11}BO_4–H]^-$: 217.0677, found 217.0678.

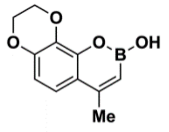

2k

4-methyl-8,9-dihydro-2H-[1,4]dioxino[2',3':3,4]benzo[1,2-e][1,2]oxaborinin-2-ol (2k): Compound **2k** was obtained from **1k** (31.0 mg, 0.200 mmol, 71%). General Procedure A: The amount of product is too low to be isolated. General Procedure B: 31.0 mg of **2k**, 0.142 mmol, 71%.

Column condition: 0% ethyl acetate for 5 CV, 20% ethyl acetate for 10 CV, 30% ethyl acetate for 10 CV, 35% ethyl acetate for 10 CV, then 40% ethyl acetate for 5 CV. The product started to elute at 35 CV.

TLC (50/50 hexanes/ethyl acetate): $R_f = 0.20$ (visualized by UV)
^{1}H NMR (400 MHz, Acetone-d$_6$) δ 7.60 (br s, 1H), 7.09 (d, J = 8.8 Hz, 1H), 6.66 (d, J = 8.8 Hz, 1H), 5.83 (q, J = 1.3 Hz, 1H), 4.33 – 4.31 (m, 4H), 2.35 (d, J = 1.2 Hz, 3H).
^{13}C{^{1}H} NMR (101 MHz, Acetone-d$_6$) δ 156.3, 145.5, 143.6, 134.3, 120.1, 117.3, 111.3, 65.3, 64.9, 22.4.
^{11}B NMR (193 MHz, Acetone-d$_6$) δ 27.7.
IR (FT-ATR, cm^{-1}, neat) ν_{max} 3413 (br), 2980 (m), 2930 (m), 2878 (m), 1604 (vs), 1563 (vs), 1499 (s), 1330 (vs), 1204 (vs).
HRMS (ESI) calcd. for $[C_{11}H_{11}BO_4+H]^+$: 219.0824, found 219.0885.

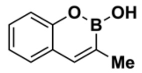

2l

3-methyl-2H-benzo[e][1,2]oxaborinin-2-ol (2l): Compound **2l** was obtained as a light yellow solid from **1l** (32.0 mg, 0.200 mmol). General Procedure A: 1.67 mg of **2l**, 0.0104 mmol, 5%; General Procedure B: 9.26 mg of **2l**, 0.0579 mmol, 29%. NMR data match previously reported values.[1]

Column condition: 0% ethyl acetate for 5 CV, 10% ethyl acetate for 10 CV, 15% ethyl acetate for 10 CV, 20% ethyl acetate for 10 CV, then 25% ethyl acetate for 5 CV. The product started to elute at 20 CV.

TLC (80/20 hexanes/ethyl acetate): $R_f = 0.20$ (visualized by UV).
^{1}H NMR (600 MHz, Acetone-d$_6$) δ 7.78 (s, 1H), 7.48 (s, 1H), 7.40 (dd, J = 7.6, 1.6 Hz, 1H), 7.29 (t, J = 7.7 Hz, 1H), 7.15 (d, J = 8.1 Hz, 1H), 7.10 (t, J = 7.7 Hz, 1H), 2.07 (s, 3H).
^{13}C{^{1}H} NMR (101 MHz, Acetone-d$_6$) δ 152.9, 144.4, 128.8, 128.7, 125.8, 122.8, 118.6, 19.0.
^{11}B NMR (128 MHz, Acetone-d$_6$) δ 27.6.

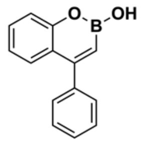

2m

4-phenyl-2*H*-benzo[*e*][1,2]oxaborinin-2-ol (2m): Compound **2m** was obtained as a light yellow solid from **1m** (44.5 mg, 0.200 mmol). General Procedure A: 21.9 mg of **2m**, 0.0986 mmol, 49%. General Procedure B: 29.3 mg of **2m**, 0.132 mmol, 66%.

Column condition: 0% ethyl acetate for 5 CV, 10% ethyl acetate for 5 CV, 15% ethyl acetate for 5 CV, 20% ethyl acetate for 10 CV, then 30% ethyl acetate for 5 CV. The product started to elute at 30 CV.

TLC (80/20 hexanes/ethyl acetate): $R_f = 0.35$ (visualized by UV)
^{1}H NMR (400 MHz, Acetone-d$_6$): δ 7.99 (s, 1H), 7.55 – 7.36 (m, 6H), 7.31 (ddd, J = 11.8, 8.1, 1.5 Hz, 2H), 7.08 (ddd, J = 8.2, 7.3, 1.3 Hz, 1H), 6.07 (s, 1H).
^{13}C{^{1}H} NMR (101 MHz, Acetone-d$_6$) δ 160.8, 154.1, 141.8, 130.4, 129.3 (2C), 129.2 (2C), 128.8, 128.2, 124.8, 122.6, 119.7.
^{11}B NMR (128 MHz, Acetone) δ 27.3.
IR (FT-ATR, cm^{-1}, neat) 3333 (br m), 3058 (w), 1595 (s), 1549 (s), 1491 (w), 1480 (w), 1445 (w), 1377 (w), 1342 (s), 1277 (m), 1241 (w), 1210 (w).
HRMS (ESI) calcd. for $[C_{14}H_{11}BO_2+H]^+$: 223.0925, found 223.0930.

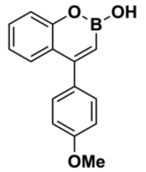

2n

4-(4-methoxyphenyl)-2*H*-benzo[*e*][1,2]oxaborinin-2-ol (2n): Compound **2n** was obtained as a light yellow solid from **1n** (50.5 mg, 0.200 mmol). General Procedure A: 35.7 mg of **2n**, 0.142 mmol, 71%. General Procedure B: 46.0 mg of **2n**, 0.183 mmol, 92%.

Column condition: 0% ethyl acetate for 5 CV, 10% ethyl acetate for 5 CV, 20% ethyl acetate for 10 CV, then 30% ethyl acetate for 10 CV. The product started to elute at 30 CV.

TLC (80/20 hexanes/ethyl acetate): $R_f = 0.23$ (visualized by UV)
^{1}H NMR (400 MHz, Acetone-d$_6$): δ 7.93 (s, 1H), 7.46 – 7.38 (m, 2H), 7.37 – 7.32 (m, 2H), 7.28 (d, J = 8.1 Hz, 1H), 7.13 – 7.01 (m, 3H), 6.04 (s, 1H), 3.87 (s, 3H).
^{13}C{^{1}H} NMR (101 MHz, Acetone-d$_6$) δ 159.7, 159.6, 153.3, 133.1, 129.6 (2C), 129.4, 127.4, 124.1, 121.7, 118.8, 113.8 (2C), 54.8.
^{11}B NMR (128 MHz, Acetone) δ 27.4.
IR (FT-ATR, cm^{-1}, neat) 3377 (br m), 3036 (w), 2932 (w), 2837 (w), 1693 (w), 1597 (s), 1549 (m), 1506 (s), 1480 (w), 1456 (w), 1379 (w), 1344 (m), 1276 (w), 1245 (s), 1211 (w).
HRMS (ESI) calcd. for $[C_{15}H_{13}BO_3+H]^+$: 253.1031, found 253.1032.

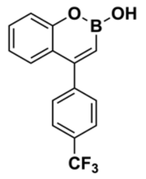

2o

4-(4-(trifluoromethyl)phenyl)-2*H*-benzo[*e*][1,2]oxaborinin-2-ol (2o): Compound **2o** was obtained as a white solid from **1o** (58.1 mg, 0.200 mmol). General Procedure A: 16.1 mg of **2o**, 0.0555 mmol, 28%. General Procedure B: 40.0 mg of **2o**, 0.141 mmol, 70%.

Column condition: 0% ethyl acetate for 5 CV, 10% ethyl acetate for 5 CV, 20% ethyl acetate for 10 CV, then 30% ethyl acetate for 10 CV. The product started to elute at 30 CV.

TLC (80/20 hexanes/ethyl acetate): $R_f = 0.28$ (visualized by UV)
^{1}H NMR (400 MHz, Acetone-d_6): δ 8.11 (s, 1H), 7.86 (d, *J* = 8.0 Hz, 2H), 7.64 (d, *J* = 8.0 Hz, 2H), 7.45 (ddd, *J* = 8.6, 7.1, 1.7 Hz, 1H), 7.31 (dd, *J* = 8.2, 1.2 Hz, 1H), 7.25 (dd, *J* = 7.9, 1.6 Hz, 1H), 7.11 (ddd, *J* = 8.1, 7.2, 1.3 Hz, 1H), 6.12 (s, 1H).
^{13}C{^{1}H} NMR (101 MHz, Acetone-d_6) δ 159.2, 154.1, 145.8, 130.7, 130.4 (q, *J* = 32.2 Hz), 130.1 (2C), 128.0, 126.3 (q, *J* = 3.9 Hz, 2C), 125.4 (q, *J* = 271.3 Hz), 124.2, 122.8, 119.8.
^{11}B NMR (128 MHz, Acetone) δ 27.7.
IR (FT-ATR, cm^{-1}, neat) 3302 (br s), 2922 (w), 1598 (m), 1550 (s), 1515 (w), 1482 (w), 1457 (w), 1414 (w), 1373 (w), 1328 (s), 1277 (w), 1209 (w)
HRMS (ESI) calcd. for $[C_{15}H_{10}BF_3O_2\text{-}H]^-$: 289.0653, found 289.0740.

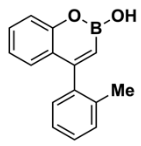

2p

4-(*o*-tolyl)-2*H*-benzo[*e*][1,2]oxaborinin-2-ol (2p): Compound **2p** was obtained as a light yellow solid from **1p** (47.3 mg, 0.200 mmol). General Procedure A: 20.4 mg of **2o**, 0.0864 mmol, 43%. General Procedure B: 32.1 mg of **2p**, 0.136 mmol, 68%.

Column condition: 0% ethyl acetate for 5 CV, 10% ethyl acetate for 5 CV, 15% ethyl acetate for 5 CV, 20% ethyl acetate for 10 CV, then 30% ethyl acetate for 5 CV. The product started to elute at 30 CV.

TLC (80/20 hexanes/ethyl acetate): $R_f = 0.35$ (visualized by UV)
^{1}H NMR (400 MHz, Acetone-d_6): δ 8.00 (s, 1H), 7.40 (ddd, *J* = 8.6, 7.2, 1.7 Hz, 1H), 7.36 – 7.26 (m, 4H), 7.15 (dt, *J* = 7.3, 1.2 Hz, 1H), 7.04 (ddd, *J* = 8.1, 7.3, 1.3 Hz, 1H), 6.91 (dd, *J* = 7.9, 1.7 Hz, 1H), 5.99 (s, 1H), 2.09 (s, 3H).
^{13}C{^{1}H} NMR (101 MHz, Acetone-d_6) δ 160.8, 153.8, 141.4, 135.7, 130.8, 130.4, 129.3, 128.8, 128.0, 126.8, 125.1, 122.8, 119.6, 19.8.
^{11}B NMR (128 MHz, Acetone-d_6) δ 27.5.
IR (FT-ATR, cm^{-1}, neat) 3323 (br m), 3061 (w), 2924 (w), 1607 (w), 1596 (s), 1552 (m), 1481 (w), 1455 (m), 1379 (w), 1340 (s), 1276 (m), 1241 (w), 1216 (w).
HRMS (ESI) calcd. for $[C_{15}H_{13}BO_2\text{+}H]^+$: 237.1081, found 237.1085.

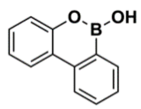

2q

6H-dibenzo[c,e][1,2]oxaborinin-6-ol (2q): Compound **2q** was obtained as a colorless solid from **1q** (39.2 mg, 0.200 mmol). General Procedure A: 8.59 mg of **2q**, 0.0438 mmol, 22%. General Procedure B: 21.1 mg of **2q**, 0.107 mmol, 54%. NMR data match previously reported values.[9]

Column condition: 0% ethyl acetate for 5 CV, 10% ethyl acetate for 10 CV, 20% ethyl acetate for 5 CV, 30% ethyl acetate for 15 CV, then 40% ethyl acetate for 5 CV. The product started to elute at 30 CV.

TLC (80/20 hexanes/ethyl acetate): R_f = 0.25 (visualized by UV)
^{1}H NMR (600 MHz, $CDCl_3$) δ 8.16 (d, *J* = 8.2 Hz, 1H), 8.13 (dd, *J* = 8.0, 1.5 Hz, 1H), 8.07 (dd, *J* = 7.4, 1.6 Hz, 1H), 7.71 (td, *J* = 7.6, 1.6 Hz, 1H), 7.47 (td, *J* = 7.3, 1.1 Hz, 1H), 7.37 (ddd, *J* = 8.4, 7.4, 1.6 Hz, 1H), 7.30-7.19 (m, 2H), 4.77 (s, 1H).
^{13}C{^{1}H} NMR (101 MHz, $CDCl_3$) δ 151.2, 140.5, 133.5, 132.7, 129.1, 127.4, 123.7, 123.1, 122.8, 121.8, 119.7.
^{11}B NMR (128 MHz, $CDCl_3$) δ 28.3.

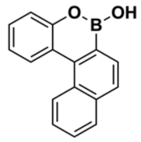

2r

6H-naphtho[2,1-c]chromen-6-one (2r): Compound **2n** was obtained as a colorless solid from **1r** (39.2 mg, 0.200 mmol). General Procedure A: 15.7 mg of **2r**, 0.0640 mmol, 32%. General Procedure B: 27.3 mg of **2r**, 0.111 mmol, 55%.

Column condition: 0% ethyl acetate for 5 CV, 10% ethyl acetate for 10 CV, 20% ethyl acetate for 10 CV, 30% ethyl acetate for 10 CV, then 40% ethyl acetate for 5 CV. The product started to elute at 30 CV.

TLC (80/20 hexanes/ethyl acetate): R_f = 0.15 (visualized by UV)
^{1}H NMR (600 MHz, Acetone-d_6) δ 8.88 – 8.83 (m, 1H), 8.49 (dd, *J* = 8.2, 2.7 Hz, 1H), 8.36 (s, 1H), 8.11 (d, *J* = 8.1 Hz, 1H), 8.08 – 8.03 (m, 1H), 7.96 (dd, *J* = 8.1, 2.8 Hz, 1H), 7.70 – 7.63 (m, 2H), 7.45 (tt, *J* = 7.6, 1.7 Hz, 1H), 7.37 (dd, *J* = 8.1, 1.4 Hz, 1H), 7.29 (td, *J* = 8.0, 2.4 Hz, 1H).
^{13}C{^{1}H} NMR (101 MHz, Acetone-d_6) δ 153.1, 140.5, 137.7, 130.0, 129.9, 129.8, 129.5, 129.0, 128.5, 128.0, 127.4, 127.2, 124.2, 122.7, 120.3.
^{11}B NMR (193 MHz, Acetone-d_6) δ 28.3.
IR (FT-ATR, cm^{-1}, neat) 3275 (br s), 2923 (w), 1914 (w), 1616 (w), 1594 (m), 1548 (m), 1483 (w), 1467 (m), 1388 (m), 1328 (w), 1308 (s), 1287 (w).
HRMS (ESI) calcd. for $[C_{16}H_{11}BO_2\text{-}H]^-$: 245.0779, found 245.0783.

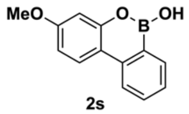

2s

3-methoxy-6H-dibenzo[c,e][1,2]oxaborinin-6-ol (2s): Compound **2s** was obtained as a colorless solid from **1s** (39.2 mg, 0.200 mmol). General Procedure A: 3.32 mg of **2s**, 0.0147 mmol, 7%. General Procedure B: 19.8 mg of **2s**, 0.0877 mmol, 44%.

Column condition: 0% ethyl acetate for 5 CV, 5% ethyl acetate for 10 CV, 10% ethyl acetate for 10 CV, 15% ethyl acetate for 10 CV, then 20% ethyl acetate for 5 CV. The product started to elute at 15 CV.

TLC (80/20 hexanes/ethyl acetate): $R_f = 0.25$ (visualized by UV).
^{1}H NMR (400 MHz, Acetone-d_6) δ 8.21 (s, 1H), 8.16 (d, J = 8.1 Hz, 1H), 8.12 (d, J = 8.8 Hz, 1H), 8.09 (dd, J = 7.4, 1.5 Hz, 1H), 7.68 (ddd, J = 8.3, 7.2, 1.5 Hz, 1H), 7.40 (td, J = 7.3, 1.0 Hz, 1H), 6.82 (dd, J = 8.8, 2.7 Hz, 1H), 6.78 (d, J = 2.6 Hz, 1H), 3.86 (s, 3H).
^{13}C{^{1}H} NMR (101 MHz, Acetone-d_6) δ 161.6, 153.8, 141.5, 134.3, 133.2, 126.9, 125.5, 121.9, 116.9, 110.5, 104.6, 55.8.
^{11}B NMR (128 MHz, Acetone-d_6) δ 28.1.
IR (FT-ATR, cm^{-1}, neat) 3463 (s), 3084 (w), 3000 (w), 2919 (m), 2838 (w), 1729 (w), 1622 (w), 1606 (s), 1580 (w), 1556 (w), 1509 (w), 1483 (m), 1451 (m), 1438 (w), 1412 (w), 1380 (s), 1344 (w), 1319 (w), 1294 (m), 1255 (w).
HRMS (ESI) calcd. for $[C_{13}H_{11}BO_3+H]^+$: 227.0874, found 227.0877.

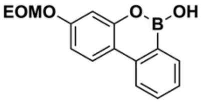

2t

3-(methoxymethoxy)-6H-dibenzo[c,e][1,2]oxaborinin-6-ol (2t): Compound **2t** was obtained as a colorless solid from **1t** (51.2 mg, 0.200 mmol) General Procedure A: 13.5 mg of **2t**, 0.0501 mmol, 25%. General Procedure B: 26.5 mg of **2t**, 0.0981 mmol, 49%.

Column condition: 0% ethyl acetate for 5 CV, 10% ethyl acetate for 10 CV, 15% ethyl acetate for 10 CV, 20% ethyl acetate for 10 CV, then 30% ethyl acetate for 5 CV. The product started to elute at 20 CV.

TLC (80/20 hexanes/ethyl acetate): $R_f = 0.25$ (visualized by UV)
^{1}H NMR (600 MHz, Acetone-d_6) δ 8.08 (s, 1H), 8.04 (t, J = 6.6 Hz, 1H), 8.00 (t, J = 7.1 Hz, 1H), 7.96 (d, J = 7.4 Hz, 1H), 7.55 (tt, J = 7.3, 1.7 Hz, 1H), 7.28 (t, J = 7.3 Hz, 1H), 6.80 – 6.75 (m, 2H), 5.17 (s, 2H), 3.59 (q, J = 7.1 Hz, 2H), 1.05 (t, J = 7.1 Hz, 3H).
^{13}C{^{1}H} NMR (101 MHz, Acetone-d_6) δ 159.3, 153.5, 141.4, 134.3, 133.2, 127.1, 125.4, 122.0, 117.8, 112.1, 107.2, 93.9, 64.9, 15.5.
^{11}B NMR (128 MHz, Acetone-d_6) δ 28.0.
IR (FT-ATR, cm^{-1}, neat) 3374 (s), 2982 (w), 2937 (w), 2883 (w), 1711 (w), 1606 (s), 1581 (w), 1557 (w), 1510 (w), 1484 (m), 1448 (m), 1419 (w), 1384 (s), 1318 (w), 1291 (s), 1256 (m).
HRMS (ESI) calcd. for $[C_{15}H_{15}BO_4+H]^+$: 271.1136, found 271.1137.

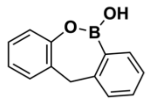

2u

dibenzo[c,f][1,2]oxaborepin-6(11H)-ol (2u): Compound **2u** was obtained as a colorless solid from **1u** (42.0 mg, 0.200 mmol) General Procedure A: 29.4 mg of **2u**, 0.140 mmol, 70%. General Procedure B: 23.8 mg of **2u**, 0.113 mmol, 57%.

Column condition: 0% ethyl acetate for 5 CV, 10% ethyl acetate for 10 CV, 15% ethyl acetate for 10 CV, 20% ethyl acetate for 10 CV, then 25% ethyl acetate for 5 CV. The product started to elute at 20 CV. The UV signal of the product is very low.

TLC (80/20 hexanes/ethyl acetate): R_f = 0.25 (visualized by UV)
^{1}H NMR (400 MHz, CD_2Cl_2) δ 7.71 (d, *J* = 7.5 Hz, 1H), 7.39 (d, *J* = 7.3 Hz, 1H), 7.30 – 7.20 (m, 3H), 7.16 (t, *J* = 7.7 Hz, 1H), 7.07 – 6.97 (m, 2H), 4.91 (br s, 1H), 3.91 (s, 2H).
^{13}C{^{1}H} NMR (101 MHz, CD_2Cl_2) δ 152.3, 147.9, 135.0, 132.1, 131.5, 129.6, 127.1, 126.5, 123.9, 120.5, 40.1.
^{11}B NMR (128 MHz, CD_2Cl_2) δ 29.3
IR (FT-ATR, cm^{-1}, neat) ν_{max} 3389 (br), 3066 (m), 2952 (m), 2926 (w), 2901 (w), 1600 (m), 1488 (s), 1443 (s), 1310 (vs).
HRMS (ESI) calcd. for $[C_{13}H_{11}BO_2{-}H]^-$: 209.0779, found 209.0806.

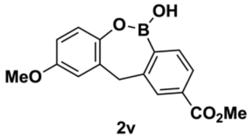

2v

methyl 6-hydroxy-2-methoxy-6,11-dihydrodibenzo[c,f][1,2]oxaborepine-9-carboxylate (2v): Compound **2v** was obtained as a colorless solid from **1v** (59.7 mg, 0.200 mmol) General Procedure A: 22.1 mg of **2v**, 0.0741 mmol, 33%. General Procedure B: The amount of product is too low to be isolated. The product started to elute at 40 CV.

Column condition: 0% ethyl acetate for 5 CV, 20% ethyl acetate for 10 CV, 30% ethyl acetate for 10 CV, 35% ethyl acetate for 10 CV, then 40% ethyl acetate for 10 CV. The product started to elute at 35 CV. The UV signal of the product is very low.

TLC (80/20 hexanes/ethyl acetate): R_f = 0.20 (visualized by UV)
^{1}H NMR (600 MHz, CD_2Cl_2) δ 7.91 (s, 1H), 7.87 (d, *J* = 7.6 Hz, 1H), 7.79 (d, *J* = 7.4 Hz, 1H), 6.97 (d, *J* = 8.8 Hz, 1H), 6.78 (d, *J* = 3.2 Hz, 1H), 6.69 (dd, *J* = 8.7, 3.2 Hz, 1H), 5.02 (s, 1H), 3.93 (s, 2H), 3.90 (s, 3H), 3.74 (s, 3H).
^{13}C{^{1}H} NMR (101 MHz, CD_2Cl_2) δ 167.1, 156.0, 147.8, 145.8, 135.2, 133.1, 131.6, 127.7, 127.2, 121.0, 114.5, 113.2, 55.9, 52.5, 40.1.
^{11}B NMR (128 MHz, Acetone-d_6) δ 29.2.
IR (FT-ATR, cm^{-1}, neat) ν_{max} 3398 (br), 2923 (s), 1724 (vs), 1609 (s), 1497 (vs), 1410 (vs), 1294 (vs).
HRMS (ESI) calcd. for $[C_{16}H_{15}BO_5{-}H]^-$: 297.0940, found 297.0948.

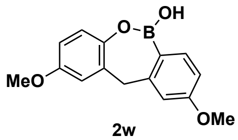

2w

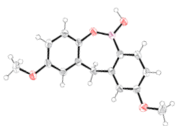

2,9-dimethoxydibenzo[c,f][1,2]oxaborepin-6(11H)-ol (2w): Compound **2w** was obtained as a white solid from **1w** (54.1 mg, 0.200 mmol). General Procedure A: 16.8 mg of **2w**, 0.0623 mmol, 31%. General Procedure B: 16.1 mg of **2w**, 0.0595 mmol, 30%.

Column condition: 0% ethyl acetate for 5 CV, 10% ethyl acetate for 10 CV, 20% ethyl acetate for 10 CV, 25% ethyl acetate for 10 CV, then 30% ethyl acetate for 10 CV. The product started to elute at 30 CV.

TLC (80/20 hexanes/ethyl acetate): R_f = 0.15 (visualized by UV)
^{1}H NMR (600 MHz, $CDCl_3$) δ 7.65 (d, *J* = 8.1 Hz, 1H), 6.95 (d, *J* = 8.8 Hz, 1H), 6.81 (d, *J* = 2.9 Hz, 1H), 6.77 (m, 2H), 6.69 (dd, *J* = 8.7, 3.2 Hz, 1H), 4.83 (br s, 1H), 3.84 (s, 2H), 3.82 (s, 3H), 3.75 (s, 3H).
^{13}C{^{1}H} NMR (101 MHz, $CDCl_3$) δ 162.8, 155.8, 149.6, 146.3, 136.9, 131.9, 121.0, 114.6, 113.1, 112.9, 111.6, 55.9, 55.5, 40.5.
^{11}B NMR (193 MHz, $CDCl_3$) δ 29.1.
IR (FT-ATR, cm^{-1}, neat) ν_{max} 3411 (br), 2957 (s), 2933 (s), 2914 (s), 2837 (s), 1603 (vs), 1496 (vs), 1239 (vs).
HRMS (ESI) calcd. for $[C_{15}H_{15}BO_4–H]^-$: 269.0990, found 269.0990.
Crystallography. A 1-dram vial was charged with **2w** (20 mg), and a mixture of dichloromethane/hexanes (20/80, 1.0 mL). The mixture was stirred until **2w** was completely dissolved. The stir bar was removed and the solution was let sit at room temperature. Crystals appeared after 72 h. A colorless plate specimen of the crystals, approximate dimensions 0.480 mm × 0.160 mm × 0.050 mm was used for the X-ray crystallographic analysis. Crystal suitable for X--ray structure determination was selected under the polarizing microscope, covered with Paratone oil and mounted on a goniometer head using Mitegen cryoloop. Experiment was performed at low temperature. QUINN software was used to calculate optimal data collection strategy. Data were collected till resolution of 0.81 Å and were truncated with XPREP till actual observed resolution. The X-ray intensity data were measured on a Bruker D8 VENTURE diffractometer system equipped with a microfocus sealed tube (Cu Kα, λ = 1.54178 Å) and a multilayer mirror monochromator. Additional details are provided in Table S10 and are available free of charge from the Cambridge Crystallographic Data Center, CCDC 2211750.

Table S9. Crystal and structural data for product 2w

**Chemical formula**	$C_{15}H_{15}BO_4$
**Formula weight**	270.08 g/mol
**Temperature**	173(2) K
**Wavelength**	1.54178 Å
**Crystal size**	0.480 × 0.160 × 0.050 mm
**Crystal habit**	colorless plate
**Crystal system**	triclinic

**Space group**	P –1	
**Unit cell dimensions**	a = 4.2390(2) Å	α = 74.345(2)°
	b = 11.1314(4) Å	β = 85.286(3)°
	c = 14.6147(6) Å	γ = 85.346(3)°
**Volume**	660.53(5) $Å^3$	
**Z**	2	
**Density (calculated)**	1.358 g/cm^3	
**Absorption coefficient**	0.792 mm^{-1}	
**F(000)**	284	
**Diffractometer**	Bruker D8 VENTURE diffractometer	
**Radiation source**	sealed tube microfocus sealed tube (Cu Kα, λ = 1.54178 Å)	
**Theta range for data collection**	3.15 to 63.60°	
**Index ranges**	-4<=h<=4, -12<=k<=12, -16<=l<=16	
**Reflections collected**	2130	
**Independent reflections**	2130 [R(int) = 0.1353]	
**Coverage of independent reflections**	100.0%	
**Absorption correction**	Multi-Scan	
**Max. and min. transmission**	0.960 and 0.500	
**Structure solution technique**	direct methods	
**Structure solution program**	SHELXT 2018/2 (Sheldrick, 2018)	
**Refinement method**	Full-matrix least-squares on F2	
**Refinement program**	SHELXL-2018/3 (Sheldrick, 2018)	
**Function minimized**	Σ w(Fo2 - Fc2)2	
**Data / restraints / parameters**	2130 / 0 / 185	
**Goodness-of-fit on F2**	1.391	
**Δ/σmax**	0.000	
**Final R indices**	1703 data; I>2σ(I)	R1 = 0.0599, wR2 = 0.1879
	all data	R1 = 0.0667, wR2 = 0.8888
**Weighting scheme**	$w=1/[\sigma^2(F_o^2)+(0.0420P)^2+0.0200P]$ where $P=(F_o^2+2F_c^2)/3$	
**Largest diff. peak and hole**	0.324 and -0.308 $eÅ^{-3}$	
**R.M.S. deviation from mean**	0.000 $eÅ^{-3}$	

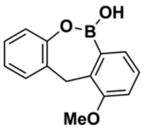

2x

10-methoxydibenzo[c,f][1,2]oxaborepin-6(11H)-ol (2x): Compound **2x** (20.0 mg, 0.0833 mmol, 42%) was obtained as a white solid from **1x** (48.1 mg, 0.200 mmol). General Procedure A: 2.57 mg of **2x**, 0.0107 mmol, 5%. General Procedure B: 20.0 mg of **2x**, 0.0835 mmol, 42%.

Column condition: 0% ethyl acetate for 5 CV, 10% ethyl acetate for 10 CV, 15% ethyl acetate for 10 CV, 20% ethyl acetate for 10 CV, then 25% ethyl acetate for 5 CV. The product started to elute at 20 CV.

TLC (80/20 hexanes/ethyl acetate): R_f = 0.20 (visualized by UV)
^{1}H NMR (400 MHz, CD_2Cl_2) δ 7.30 – 7.24 (m, 2H), 7.23 – 7.12 (m, 2H), 7.06 – 6.96 (m, 3H), 4.99 (br s, 1H), 4.00 (s, 2H), 3.86 (s, 3H).
^{13}C{^{1}H} NMR (101 MHz, CD_2Cl_2) δ 155.5, 152.8, 135.9, 131.8, 129.8, 128.0, 127.5, 126.5, 123.8, 120.3, 114.3, 56.2, 30.0.
^{11}B NMR (193 MHz, CD_2Cl_2) δ 29.3.
IR (FT-ATR, cm^{-1}, neat) ν_{max} 3420 (vs), 3000 (m), 2950 (m), 2832 (m), 1574 (m), 1453 (vs), 1368 (vs), 1311 (vs), 1255 (vs).
HRMS (ESI) calcd. for $[C_{14}H_{13}BO_3–H]^–$: 239.0884, found 239.0904.

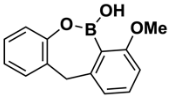

2y

7-methoxydibenzo[c,f][1,2]oxaborepin-6(11H)-ol (2y): Compound **2y** was obtained as a white solid from **1y** (48.1 mg, 0.200 mmol). General Procedure A: 38.0 mg of **2y**, 0.158 mmol, 79%. General Procedure B: 21.0 mg of **2y**, 0.0875 mmol, 44%.

Column condition: 0% ethyl acetate for 5 CV, 10% ethyl acetate for 10 CV, 15% ethyl acetate for 10 CV, 20% ethyl acetate for 10 CV, then 25% ethyl acetate for 5 CV. The product started to elute at 20 CV.

TLC (80/20 hexanes/ethyl acetate): R_f = 0.20 (visualized by UV)
^{1}H NMR (400 MHz, CD_2Cl_2) δ 7.35 (dd, *J* = 8.4, 7.5 Hz, 1H), 7.21 (dd, *J* = 7.5, 1.8 Hz, 1H), 7.17 (dt, *J* = 7.5, 1.8 Hz, 1H), 7.08 (dd, *J* = 8.0, 1.3 Hz, 1H), 6.99 (td, *J* = 7.4, 1.4 Hz, 1H), 6.92 (dd, *J* = 7.5, 0.9 Hz, 1H), 6.80 (d, *J* = 8.4 Hz, 1H), 6.37 (br s, 1H), 3.84 (2 overlapping s, 5H).
^{13}C{^{1}H} NMR (101 MHz, CD_2Cl_2) δ 164.7, 152.3, 149.8, 133.2, 131.4, 129.1, 128.2, 123.6, 120.8, 120.5, 108.8, 56.0, 39.9.
^{11}B NMR (193 MHz, CD_2Cl_2) δ 29.2.
IR (FT-ATR, cm^{-1}, neat) ν_{max} 3538 (br), 2945 (m), 2839 (m), 1597 (vs), 1571 (vs), 1465 (vs), 1318 (vs), 1244 (vs).
HRMS (ESI) calcd. for $[C_{14}H_{13}BO_3–H]^–$: 239.0884, found 239.0901.

5.3. Unsuccessful substrates

During the course of our studies, several coumarin derivatives failed to undergo efficient transformation to the desired benzoxaborins. These substrates are listed here for reference.

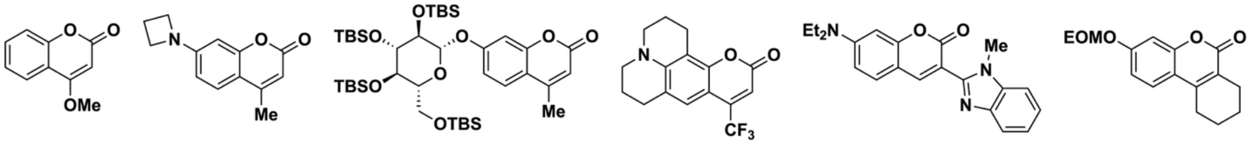

6. Experimental procedures for applications of C-to-B swap

6.1. Synthesis of dibenzo[b,f]oxepine (4) from coumarin 1a

Large scale reaction of coumarin 1a: A 250-mL Schlenk bomb was charged with a stir bar and coumarin **1a** (292 mg, 2.00 mmol). The vessel was brought inside a glovebox, then $Ni(cod)_2$ (0.110 g, 0.40 mmol, 20 mol%), P^nBu_3 (0.162 g, 0.80 mmol, 40 mol%), B_2eg_2 (0.340 g, 2.40 mmol, 1.2 equiv), and xylene (10.0 mL) were added. The vessel was sealed, brought outside, and placed in an oil bath preheated at 170 °C. The mixture was stirred (700 rpm) for 20 h at 170 °C. After cooling to room temperature, the mixture was filtered, and the filtrate was concentrated under vacuum. The crude product was chromatographed by Combiflash using a 12-g Gold column and ethyl acetate/hexanes mixture as the eluent (0% ethyl acetate for 5 CV, 10% ethyl acetate for 20 CV, 20% ethyl acetate for 30 CV). The product started to elute at 25 CV. The combined product containing fractions were concentrated by rotary evaporator. The residue was dried under vacuum to give **2a** (173 mg, 1.19 mmol, 59%) as a white solid. NMR data match the reported values as described in section 5.2.

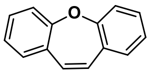

4

dibenzo[b,f]oxepine (4): A 2-dram vial was charged with a stir bar and **2a** (14.6 mg, 0.100 mmol). The vial was brought inside a glovebox then $Pd(OAc)_2$ (2.2 mg, 0.010 mmol, 10 mol%), BrettPhos (8.1 mg, 0.015 mmol, 15 mol%), 1,2-dibromobenzene (30 µL, 0.200 mmol, 2.0 equiv), CsF (60 mg, 0.400 mmol, 4.0 equiv), and toluene (1.0 mL) were added. The vial was sealed and brought outside and placed in an aluminum heating block preheated at 100 °C. The mixture was stirred (600 rpm) at 100 °C for 48 h. After cooling to room temperature, the mixture was loaded directly onto a silica gel column (1 × 20 cm). The column was eluted with hexanes (20 mL) and 10/90 DCM/hexanes (100 mL). The product containing fractions were combined and concentrated by rotary evaporator. The residue was dried under vacuum to give **4** (19.4 mg, 0.100 mmol, >99%) as a white solid. NMR data match previously reported values.[10]

TLC (80/20 hexanes/DCM): $R_f = 0.50$ (visualized by UV)
^{1}H NMR (400 MHz, $CDCl_3$) 7.30 (dt, J = 7.7, 1.7 Hz, 2H), 7.18 (dd, J = 9.8, 7.9 Hz, 4H), 7.12 (t, J = 7.4 Hz, 2H), 6.72 (s, 2H).
^{13}C{^{1}H} NMR (151 MHz, $CDCl_3$) δ 157.5, 130.7, 130.2, 130.0, 129.5, 125.0, 121.5.

6.2. Synthesis of heteroarenes 6 and 7 from phenanthrene 5

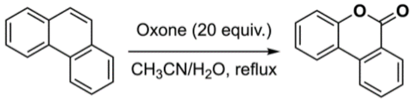

5 1q

6H-benzo[c]chromen-6-one (1q): A 100-mL round bottom flask was charged with a stir bar, phenanthrene **5** (0.356 g, 2.00 mmol), Oxone (24.4 g, 40 mmol, 20 equiv), CH_3CN (20 mL), and water (20 mL). The flask was equipped with a reflux condenser and was placed in an oil bath preheated at 120 °C. The mixture was stirred (700 rpm) and heated at 120 °C for 72 h. After cooling to room temperature, the mixture was decanted into a separatory funnel. The remaining white solid was rinsed with ethyl acetate (10 × 5 mL). The ethyl acetate rinse was added into the separatory funnel. The organic phase was collected and the aqueous phase was extracted with more ethyl acetate (2 × 10 mL). The combined organic fractions were concentrated by rotary evaporator. The residue was loaded onto a Combiflash sample cartridge packed with silica gel (5 × 5 cm). The sample was chromatographed by Combiflash using a 12-g RediSep Gold column and hexanes/ethyl acetate mixture as the eluent (0% ethyl acetate for 7 CV then 20% ethyl acetate for 20 CV). The product started to elute at 13 CV. The combined product containing fractions were concentrated by rotary evaporator. The residue was dried under vacuum to give **1q** (0.242 g, 1.24 mmol, 62%) as a white solid. NMR data match previously reported values.[6,11]

TLC (80/20 hexanes/ethyl acetate): $R_f = 0.30$ (visualized by UV)
^{1}H NMR (400 MHz, $CDCl_3$) δ 8.42 (dd, J = 8.0, 1.5 Hz, 1H), 8.15 (d, J = 8.1 Hz, 1H), 8.08 (dd, J = 8.0, 1.5 Hz, 1H), 7.84 (ddd, J = 8.3, 7.3, 1.4 Hz, 1H), 7.60 (ddd, J = 8.2, 7.3, 1.1 Hz, 1H), 7.50 (ddd, J = 8.5, 7.1, 1.6 Hz, 1H), 7.38 – 7.31 (m, 2H).
^{13}C{^{1}H} NMR (101 MHz, $CDCl_3$) δ 161.4, 151.4, 135.0, 134.9, 130.7, 130.6, 129.0, 124.7, 122.9, 121.8, 121.4, 118.2, 117.9.

Large scale reaction of 1q: A 100-mL Schlenk bomb was charged with a stir bar and benzocoumarin **1q** (235 mg, 1.20 mmol). The vessel was brought inside a glovebox then $Ni(cod)_2$ (66.0 mg, 0.24 mmol, 20 mol%), dcype (101 mg, 0.24 mmol, 20 mol%), B_2eg_2 (204 mg, 1.44 mmol, 1.2 equiv), and xylene (6 mL) were added. The vessel was sealed, brought outside, and placed in an oil bath preheated at 170 °C. The mixture was stirred (700 rpm) for 40 h at 170 °C. After cooling to room temperature, the mixture was filtered, and the filtrate was concentrated under vacuum. The residue was loaded onto a Combiflash sample cartridge packed with silica gel (5 × 10 cm). The sample was chromatographed by Combiflash using 12-g Gold column and ethyl acetate/hexanes mixture as the eluent (0% ethyl acetate for 5 CV, 10% ethyl acetate for 20 CV, 20% ethyl acetate for 30 CV). The product started to elute at 20 CV. The combined product containing fractions were concentrated by rotary evaporator. The residue was dried under vacuum to give **2q** (110 mg, 0.648 mmol, 47%) as a white solid. NMR data match the reported values as described in section 5.2.

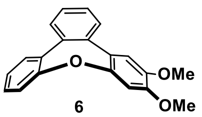

6

6,7-dimethoxytribenzo[b,d,f]oxepine (6): A 2-dram vial was charged with a stir bar and **2q** (20.0 mg, 0.100 mmol). The vial was brought inside a glovebox then $Pd(OAc)_2$ (2.2 mg, 0.010 mmol,

10 mol%), BrettPhos (8.1 mg, 0.015 mmol, 15 mol%), 4,5-dibromoveratrole (60 mg, 0.200 mmol, 2.0 equiv), CsF (60 mg, 0.400 mmol, 4.0 equiv), and toluene (1.0 mL) were added. The vial was sealed and brought outside and placed in an aluminum heating block preheated at 100 °C. The mixture was stirred (600 rpm) at 100 °C for 48 h. After cooling to room temperature, the mixture was loaded directly onto a Combiflash sample cartridge packed with silica gel (5 × 5 cm). The sample was chromatographed by Combiflash using a 12-g Gold column and hexanes/ethyl acetate mixture as the eluent (0% ethyl acetate for 10 CV then 10% ethyl acetate for another 30 CV). The product started to elute at 20 CV. The combined product containing fractions were concentrated by rotary evaporator. The residue was dried under vacuum to give **6** (42.0 mg, 0.088 mmol, 88%) as a white solid.

TLC (80/20 hexanes/ethyl acetate): R_f = 0.30 (visualized by UV)
^{1}H NMR (600 MHz, CD_2Cl_2) δ 7.62 (m, 2H), 7.57 (dd, *J* = 7.7, 1.7 Hz, 1H), 7.47 (m, 2H), 7.36 (ddd, *J* = 8.1, 7.3, 1.7 Hz, 1H), 7.30 (dd, *J* = 8.1, 1.3 Hz, 1H), 7.26 (dt, *J* = 7.5, 1.4 Hz, 1H), 7.03 (s, 1H), 6.88 (s, 1H), 3.88 (s, 3H), 3.85 (s, 3H).
^{13}C{^{1}H} NMR (101 MHz, CD_2Cl_2) δ 160.8, 154.1, 150.6, 147.3, 136.9, 136.7, 133.2, 130.1, 129.7 (2 × s), 129.0, 128.4, 127.9, 125.9, 124.0, 120.9, 112.3, 104.9, 56.7, 56.4.
IR (FT-ATR, cm^{-1}, neat) ν_{max} 2929 (s), 2849 (m), 1612 (m), 1512 (vs), 1494 (vs), 1430 (vs), 1200 (vs).
HRMS (ESI) calcd. for $[C_{20}H_{16}O_3+H]^-$: 305.1173, found 305.1176.

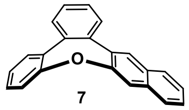

7

dibenzo[b,d]naphtho[2,3-f]oxepine (7): A 2-dram vial was charged with a stir bar and **2l** (20.0 mg, 0.100 mmol). The vial was brought inside a glovebox then $Pd(OAc)_2$ (2.2 mg, 0.010 mmol, 10 mol%), BrettPhos (8.1 mg, 0.015 mmol, 15 mol%), 2,3-dibromonaphthalene (57 mg, 0.200 mmol, 2.0 equiv), CsF (60 mg, 0.400 mmol, 4.0 equiv), and toluene (1.0 mL) were added. The vial was sealed and brought outside and placed in an aluminum heating block preheated at 100 °C. The mixture was stirred (600 rpm) at 100 °C for 48 h. After cooling to room temperature, the mixture was loaded directly onto a silica gel column (1 × 20 cm). The column was eluted with hexanes (20 mL) and 10/90 DCM/hexanes (100 mL). The product containing fractions were combined and concentrated by rotary evaporator. The residue was dried under vacuum to give **7** (24.0 mg, 0.082 mmol, 82%) as a white solid.
TLC (80/20 hexanes/DCM): R_f = 0.50 (visualized by UV)
^{1}H NMR (600 MHz, CD_2Cl_2) δ 8.05 (s, 1H), 7.89 (d, *J* = 8.1 Hz, 1H), 7.84 (d, *J* = 8.1 Hz, 1H), 7.79 (dt, *J* = 4.5, 2.3 Hz, 1H), 7.75 (s, 1H), 7.67 (dt, *J* = 4.6, 3.0 Hz, 1H), 7.60 (dd, *J* = 7.8, 1.6 Hz, 1H), 7.57 – 7.53 (m, 2H), 7.52 – 7.43 (m, 2H), 7.43 – 7.34 (m, 2H), 7.27 (t, *J* = 7.4 Hz, 1H).
^{13}C{^{1}H} NMR (101 MHz, CD_2Cl_2) δ 159.6, 158.9, 137.0, 136.8, 134.2, 133.4, 133.2, 131.9, 130.2, 130.1, 129.9, 129.7, 129.6, 128.9, 128.7, 128.3, 127.3, 127.0, 126.1, 125.9, 121.3, 117.3.
IR (FT-ATR, cm^{-1}, neat) ν_{max} 3055 (m), 2924 (s), 2853 (m), 1424 (s), 1263 (s), 1203 (s).
HRMS (ESI) calcd. for $C_{22}H_{14}O$: 294.1045, found 294.0679.

7. References

1. Saito, H.; Otsuka, S.; Nogi, K; Yorimitsu, H. *J. Am. Chem. Soc.*, **2016**, *138*, 15315-15318.
2. Xu, Y.-Z.; Tian, J.-W.; Sha, F.; Li, Q.; Wu, X.-Y. *J. Org. Chem.*, **2021**, *86*, 6765-6779.
3. Bulut, M.; Erk, C. *Dyes Pigm.*, **1996**, *30*, 99-104.
4. Melliou, E.; Magiatis, P.; Mitaku, S.; Skaltsounis, A.-L.; Chinou, E.; Chinou, I. *J. Nat. Prod.*, **2005**, *68*, 78-82.
5. Tang, Z.-Y.; Hu, Q-S. *Adv. Synth. Catal.* **2004**, *346*, 1635–1637.
6. Luu, Q. H.; Li, J. *Chem. Sci.*, **2022**, *13*, 1095-1100.
7. Bringmann, G.; Breuning, M.; Henschel, P.; Hinrichs, J. *Org. Synth.*, **2002**, *79*, 72-83.
8. Eck, M.; Würtemberger-Pietsch, S.; Eichhorn, A.; Berthel, J. H.; Bertermann, R.; Paul, U. S. et al. *Dalton Trans.*, **2017**, *46*, 3661-3680
9. Sumida, Y.; Harada, R.; Kato-Sumida, T.; Johmoto, K.; Uekusa, H. Hosoya, T. *Org. Lett.*, **2014**, *16*, 6240-6243.
10. Kotani, R.; Liu, L.; Kumar, P.; Kuramochi, H.; Tahara, T.; Liu, P.; Osuka, A.; Karadakov, P. B.; Saito, S. *J. Am. Chem. Soc.*, **2020**, *142*, 14985-14992.
11. Zhou, Q. J.; Worm, K.; Dolle, R. E. *J. Org. Chem.*, **2004**, *69*, 5147-5149.

8. NMR spectra of new compounds

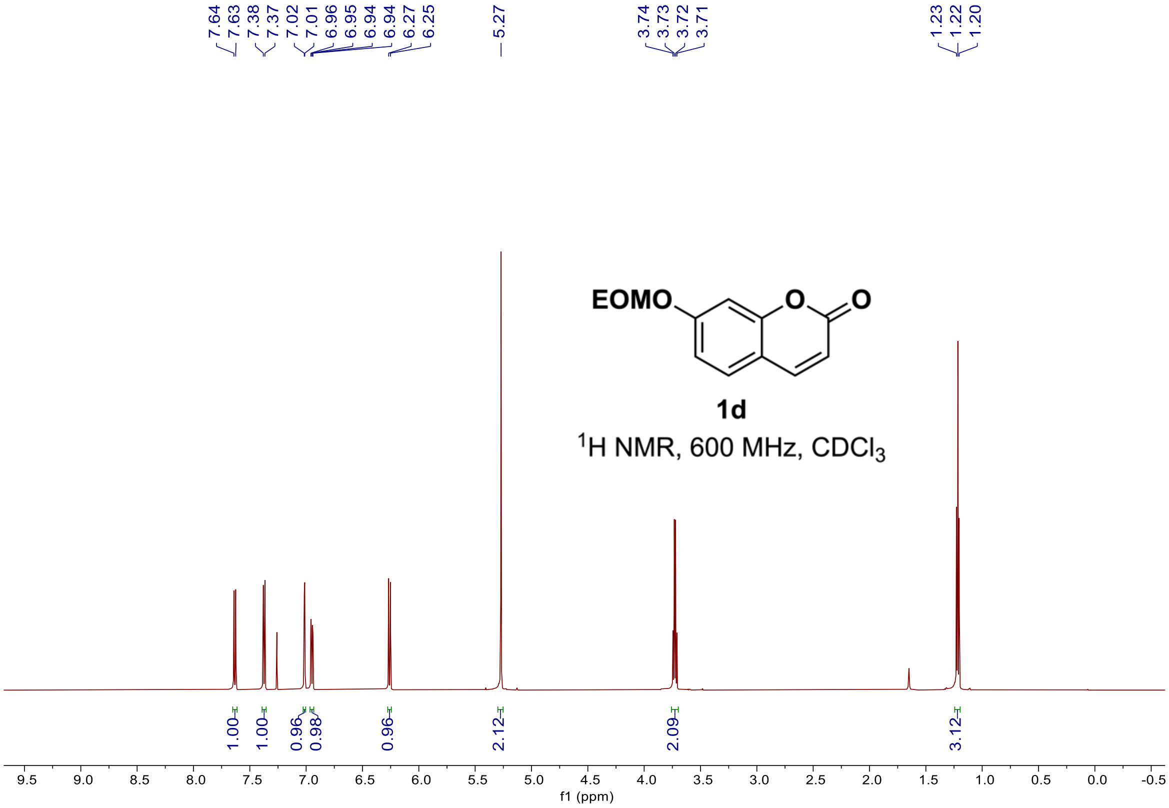

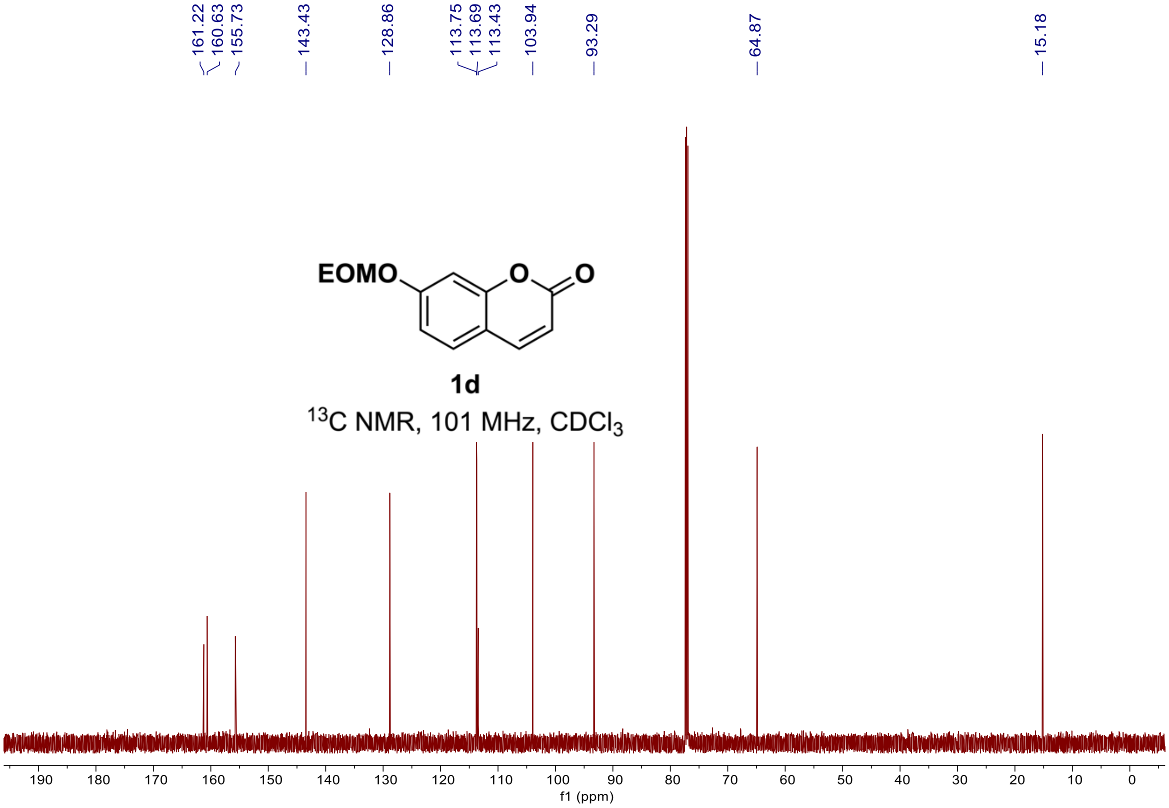

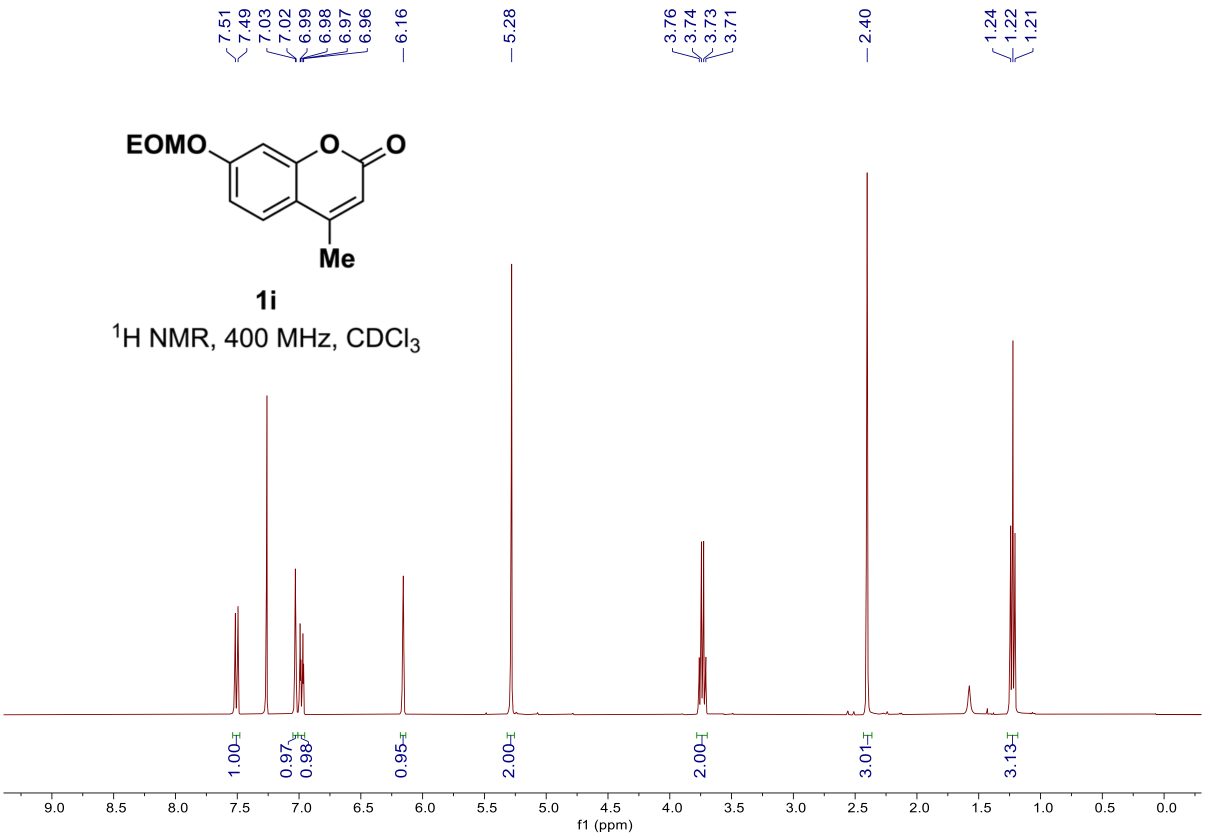
7.51
7.49
7.03
7.02
6.99
6.98
6.97
6.96
6.16
5.28
3.76
3.74
3.73
3.71
2.40
1.24
1.22
1.21
EOMO
O
O
Me
1i
1H NMR, 400 MHz, CDCl3
1.00
0.97
0.98
0.95
2.00
2.00
3.01
3.13
9.0 8.5 8.0 7.5 7.0 6.5 6.0 5.5 5.0 4.5 4.0 3.5 3.0 2.5 2.0 1.5 1.0 0.5 0.0
f1 (ppm)

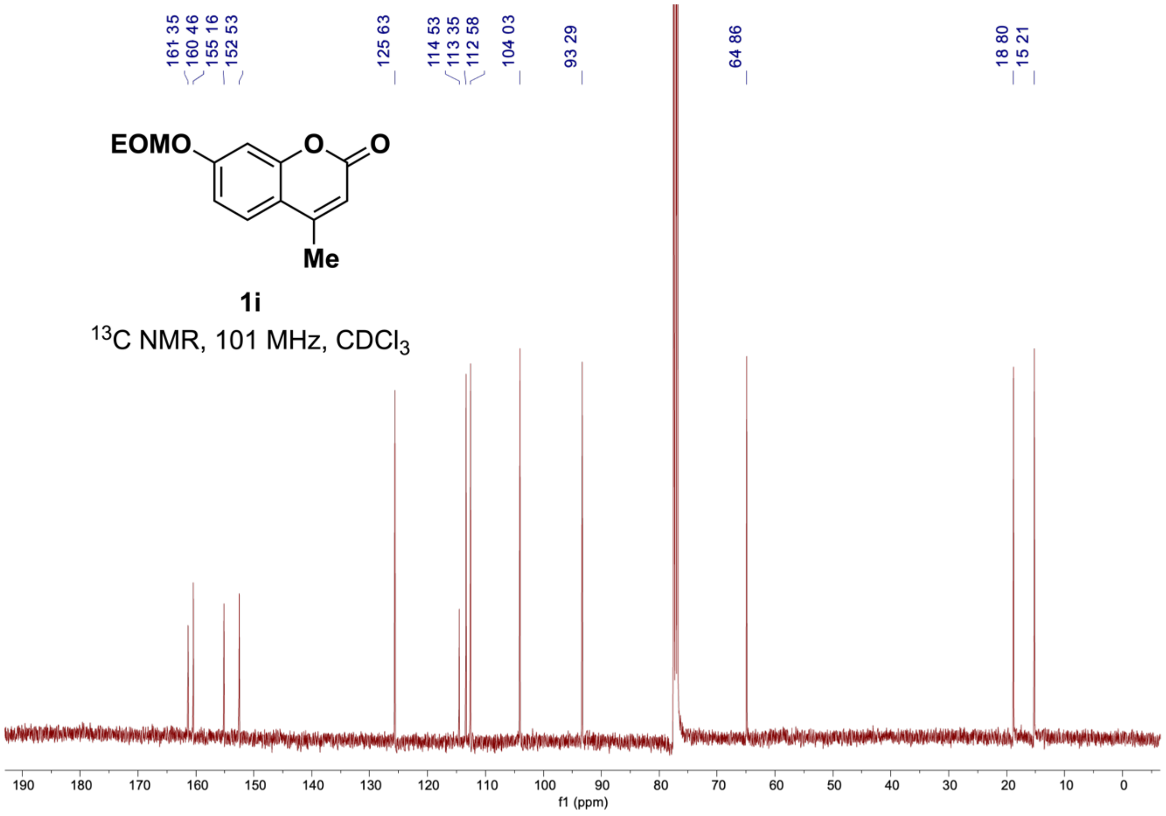
161 35
160 46
155 16
152 53
125 63
114 53
113 35
112 58
104 03
93 29
64 86
18 80
15 21
EOMO
O
O
Me
1i
13C NMR, 101 MHz, CDCl3
190 180 170 160 150 140 130 120 110 100 90 80 70 60 50 40 30 20 10 0
f1 (ppm)

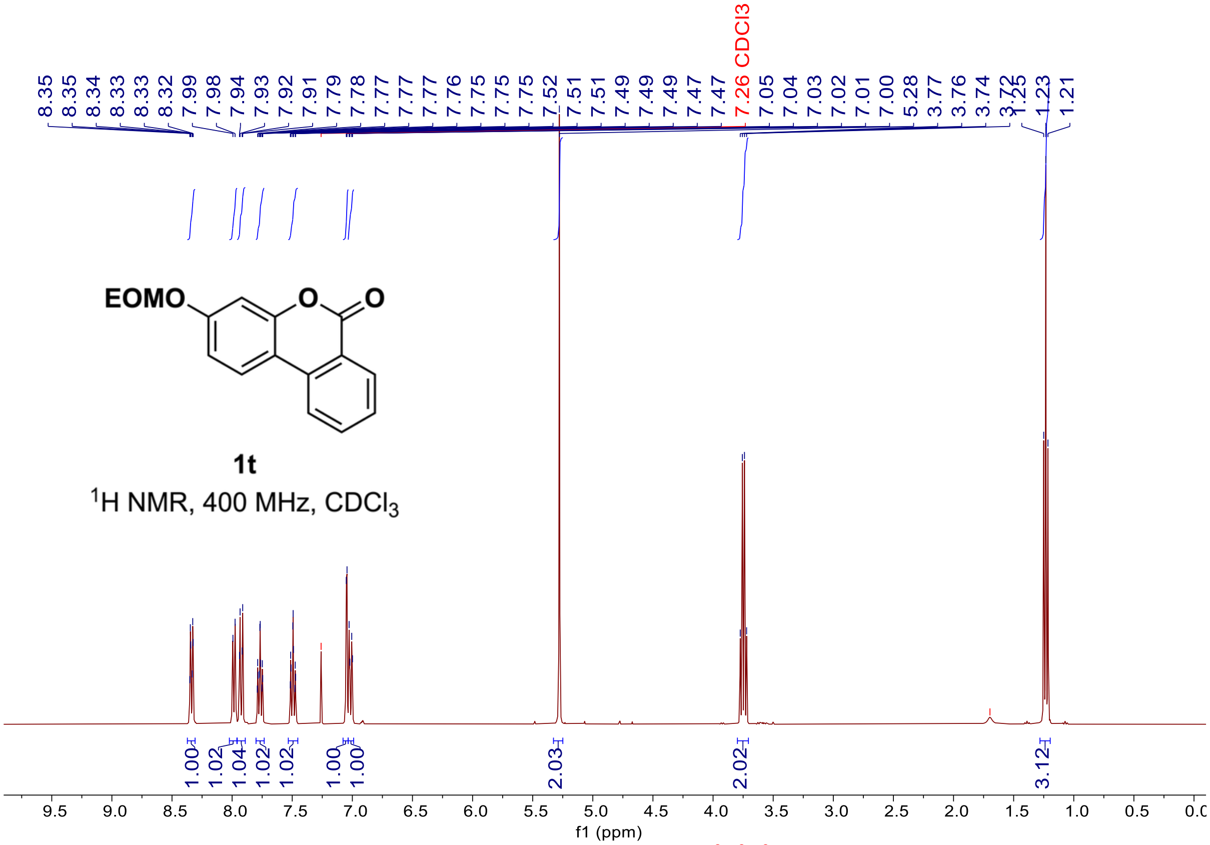
8.35 8.35 8.34 8.33 8.33 8.32 7.99 7.98 7.94 7.93 7.92 7.91 7.79 7.78 7.77 7.77 7.77 7.76 7.75 7.75 7.75 7.52 7.51 7.51 7.49 7.49 7.49 7.47 7.47 7.26 CDCl3 7.05 7.04 7.03 7.02 7.01 7.00 5.28 3.77 3.76 3.74 3.72 1.25 1.23 1.21
EOMO
O
O
1t
^{1}H NMR, 400 MHz, $CDCl_3$
1.00 1.02 1.04 1.02 1.02 1.00 1.00 2.03 2.02 3.12
9.5 9.0 8.5 8.0 7.5 7.0 6.5 6.0 5.5 5.0 4.5 4.0 3.5 3.0 2.5 2.0 1.5 1.0 0.5
f1 (ppm)

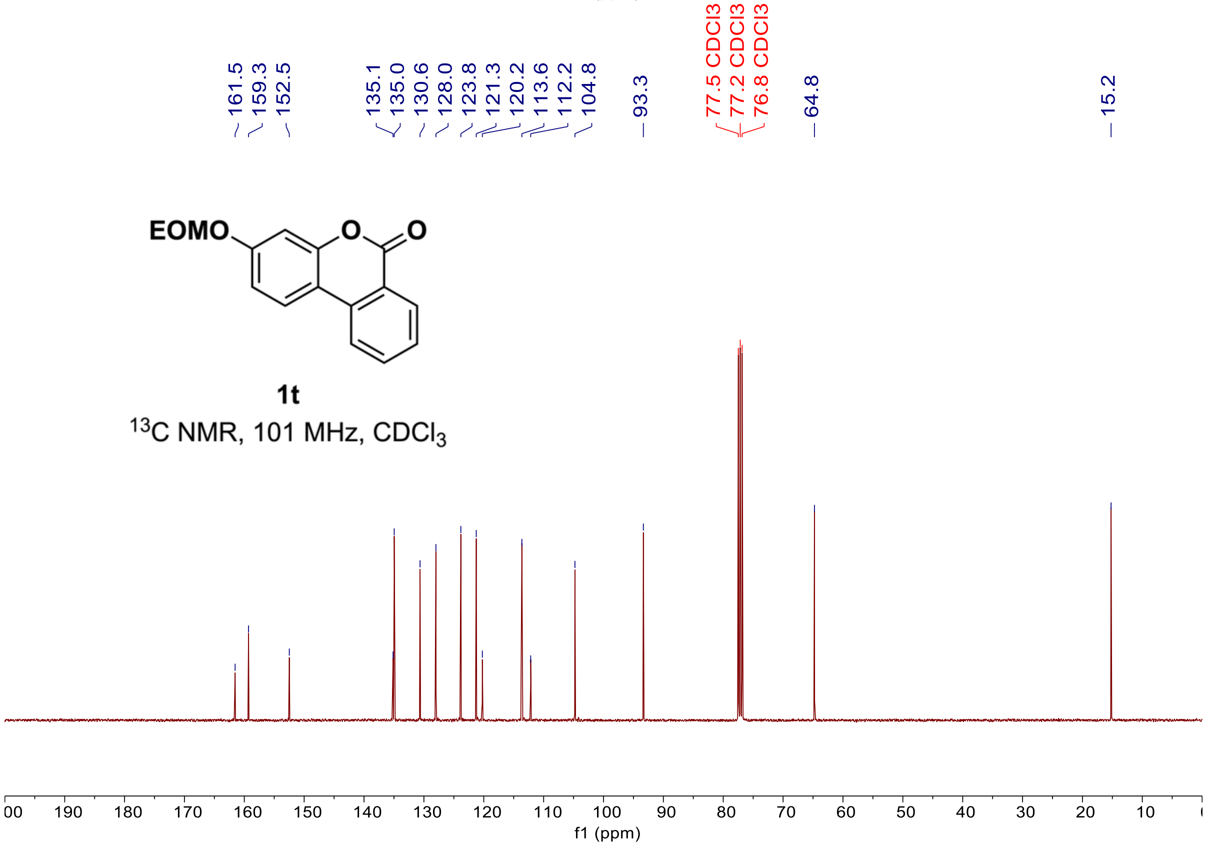
161.5 159.3 152.5 135.1 135.0 130.6 128.0 123.8 121.3 120.2 113.6 112.2 104.8 93.3 77.5 CDCl3 77.2 CDCl3 76.8 CDCl3 64.8 15.2
EOMO
O
O
1t
^{13}C NMR, 101 MHz, $CDCl_3$
190 180 170 160 150 140 130 120 110 100 90 80 70 60 50 40 30 20 10
f1 (ppm)

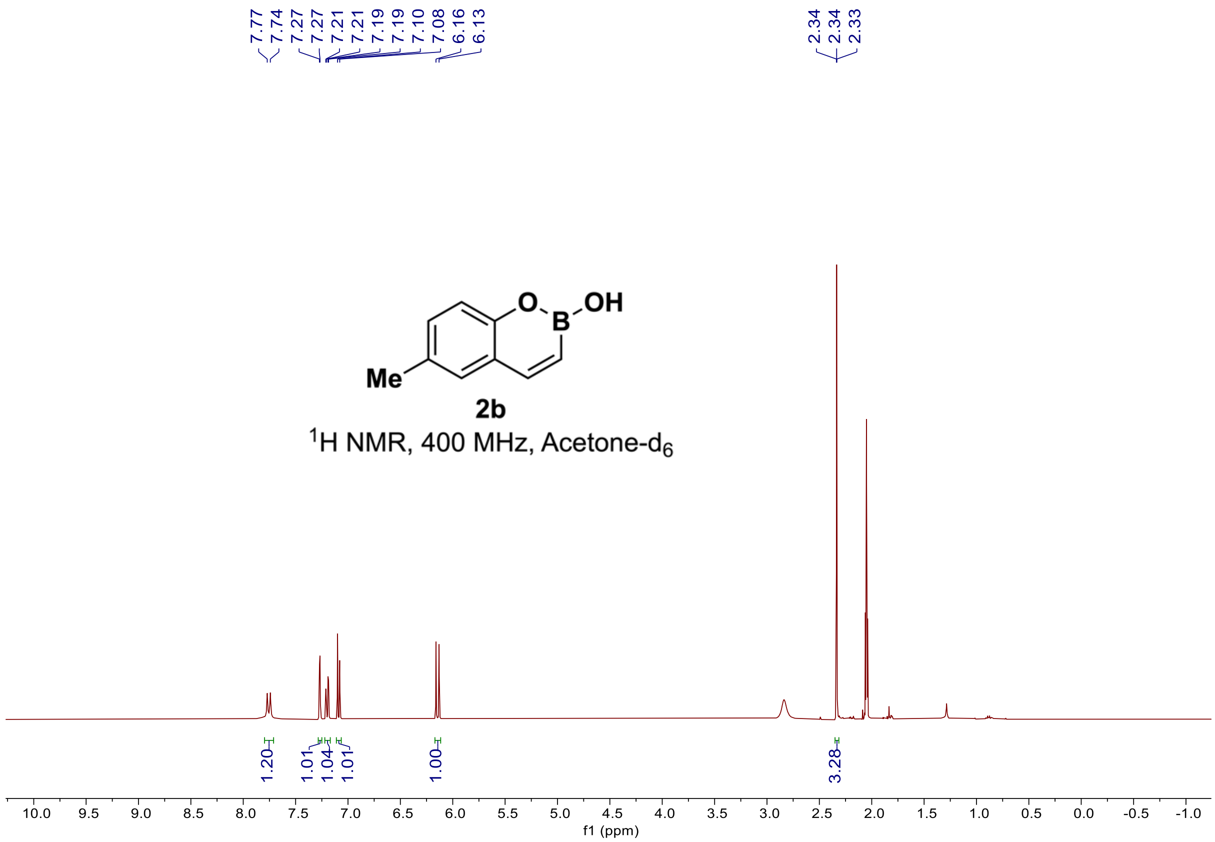
7.77
7.74
7.27
7.27
7.21
7.21
7.19
7.19
7.10
7.08
6.16
6.13
2.34
2.34
2.33
O
B
OH
Me
2b
^{1}H NMR, 400 MHz, Acetone-d_6
1.20
1.01
1.04
1.01
1.00
3.28
10.0 9.5 9.0 8.5 8.0 7.5 7.0 6.5 6.0 5.5 5.0 4.5 4.0 3.5 3.0 2.5 2.0 1.5 1.0 0.5 0.0 -0.5 -1.0
f1 (ppm)

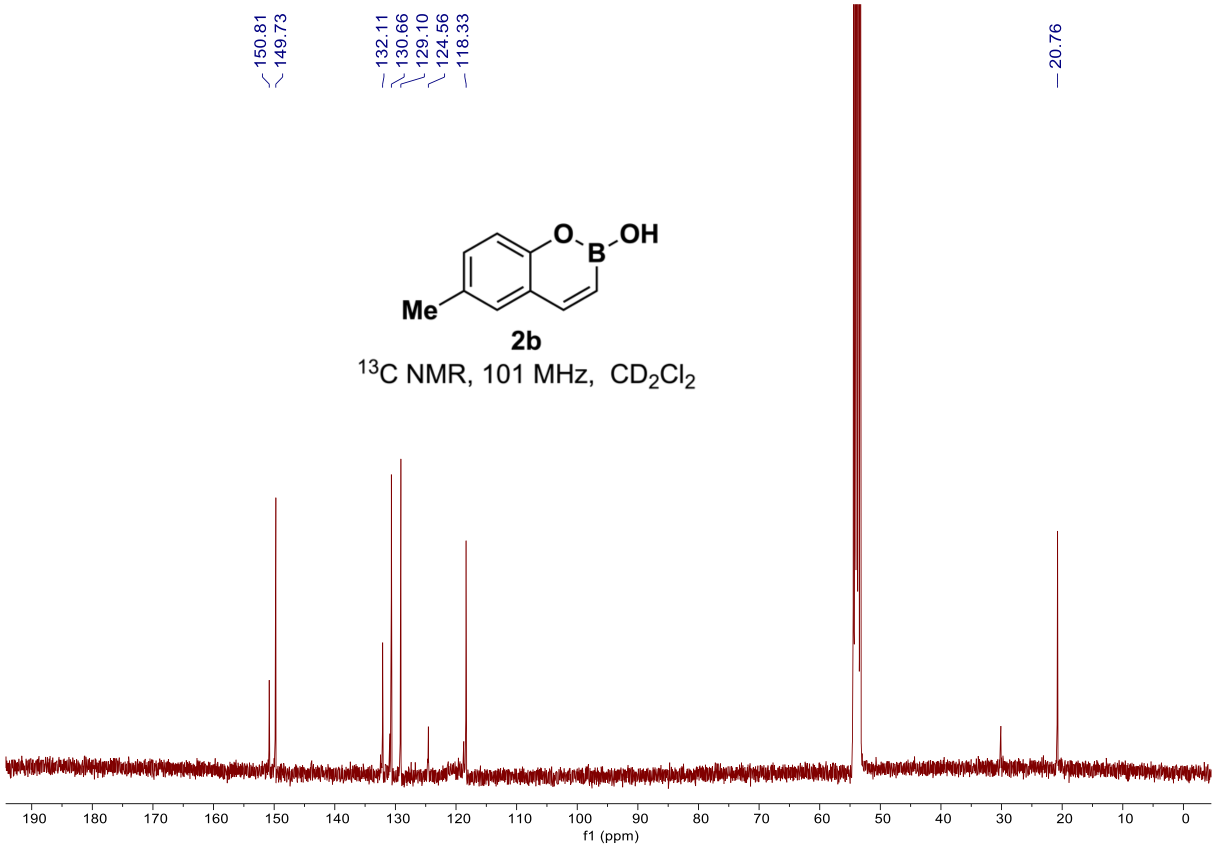
150.81
149.73
132.11
130.66
129.10
124.56
118.33
20.76
O
B
OH
Me
2b
^{13}C NMR, 101 MHz, CD_2Cl_2
190 180 170 160 150 140 130 120 110 100 90 80 70 60 50 40 30 20 10 0
f1 (ppm)

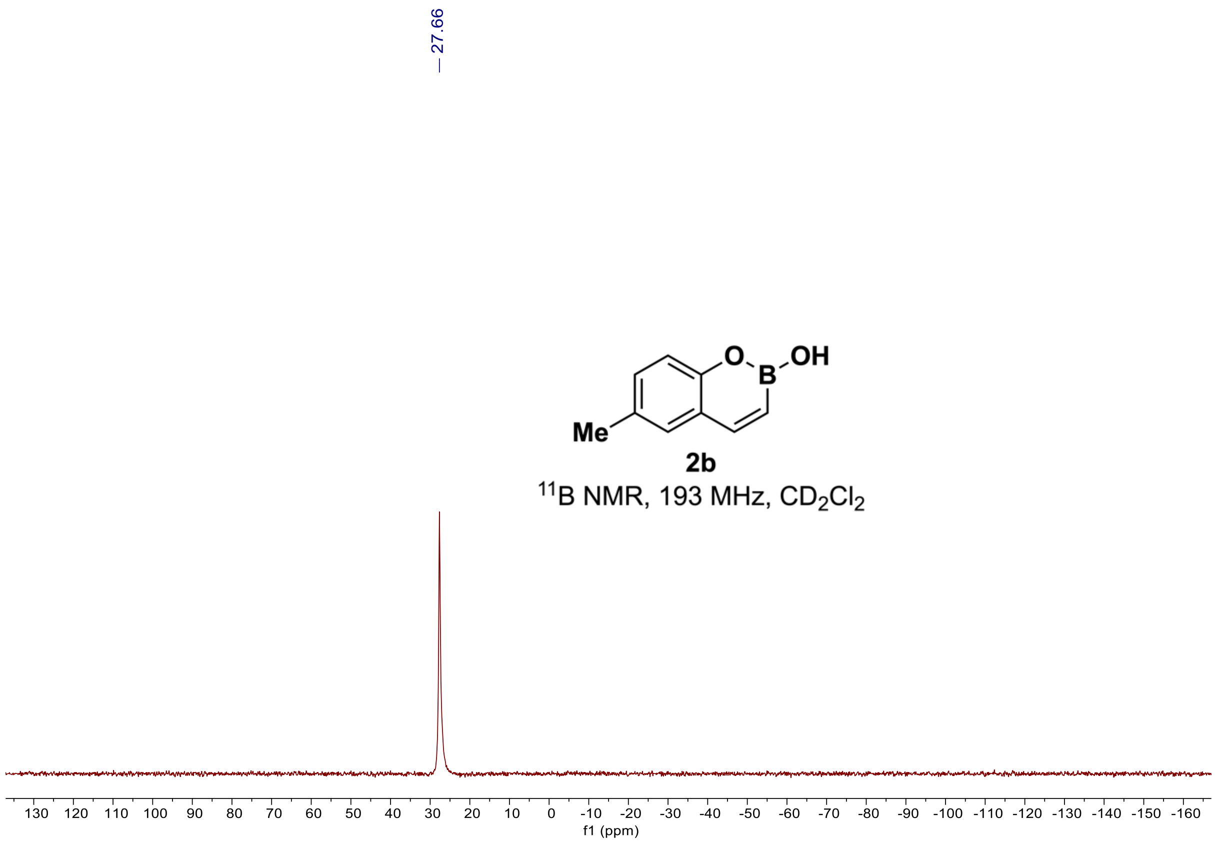
27.66
OH
O
B
Me
2b
11B NMR, 193 MHz, CD2Cl2
130 120 110 100 90 80 70 60 50 40 30 20 10 0 -10 -20 -30 -40 -50 -60 -70 -80 -90 -100 -110 -120 -130 -140 -150 -160
f1 (ppm)

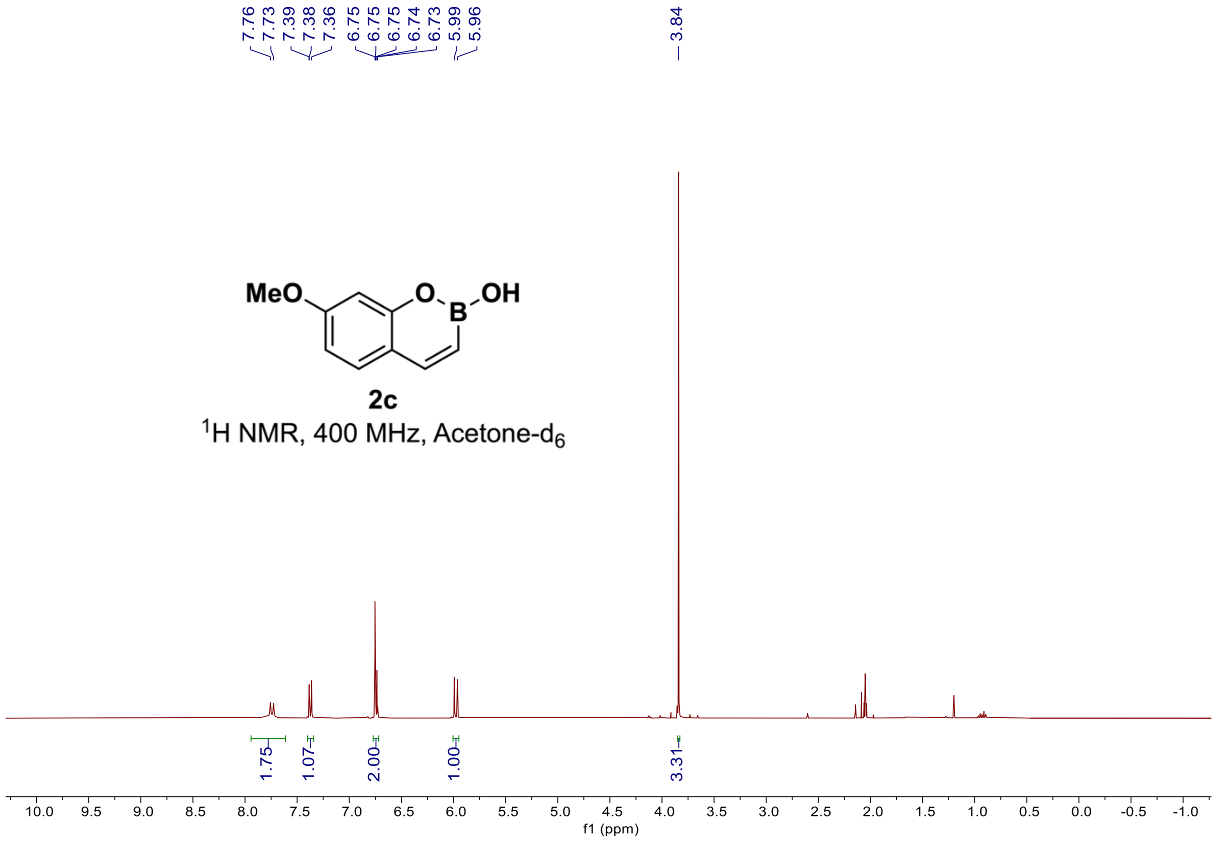
7.76
7.73
7.39
7.38
7.36
6.75
6.75
6.75
6.74
6.73
5.99
5.96
3.84
MeO
O
B
OH
2c
1H NMR, 400 MHz, Acetone-d6
1.75
1.07
2.00
1.00
3.31
10.0 9.5 9.0 8.5 8.0 7.5 7.0 6.5 6.0 5.5 5.0 4.5 4.0 3.5 3.0 2.5 2.0 1.5 1.0 0.5 0.0 -0.5 -1.0
f1 (ppm)

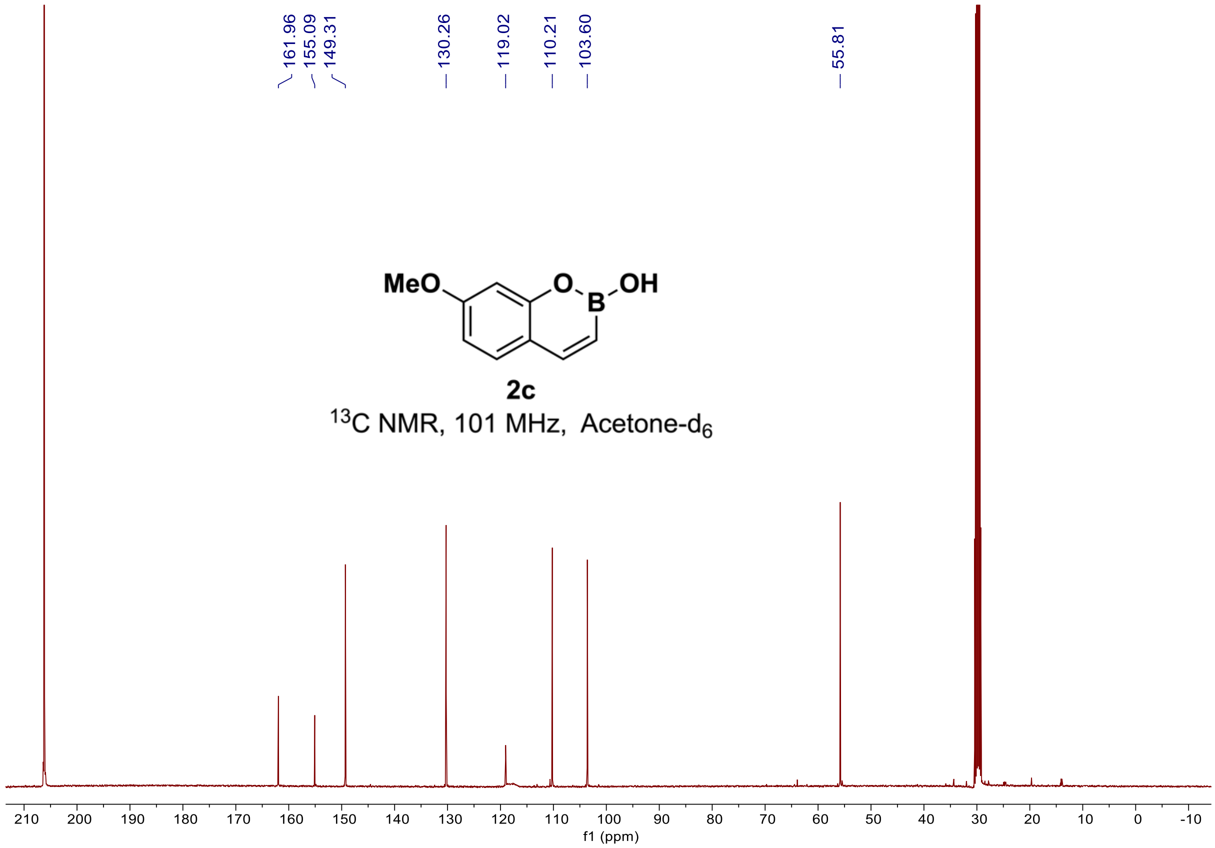
161.96
155.09
149.31
130.26
119.02
110.21
103.60
55.81
MeO
O
B
OH
2c
^{13}C NMR, 101 MHz, Acetone-d_6
210 200 190 180 170 160 150 140 130 120 110 100 90 80 70 60 50 40 30 20 10 0 -10
f1 (ppm)

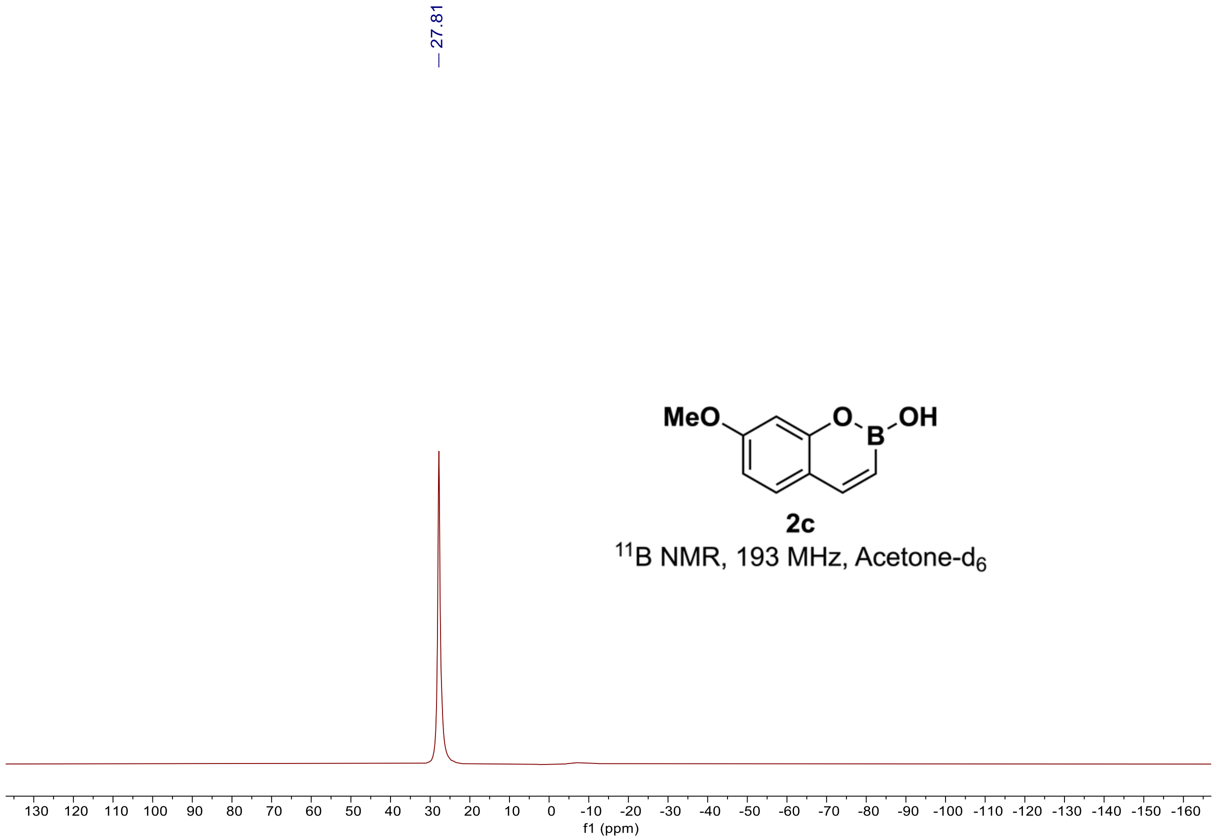
27.81
MeO
O
B
OH
2c
^{11}B NMR, 193 MHz, Acetone-d_6
130 120 110 100 90 80 70 60 50 40 30 20 10 0 -10 -20 -30 -40 -50 -60 -70 -80 -90 -100 -110 -120 -130 -140 -150 -160
f1 (ppm)

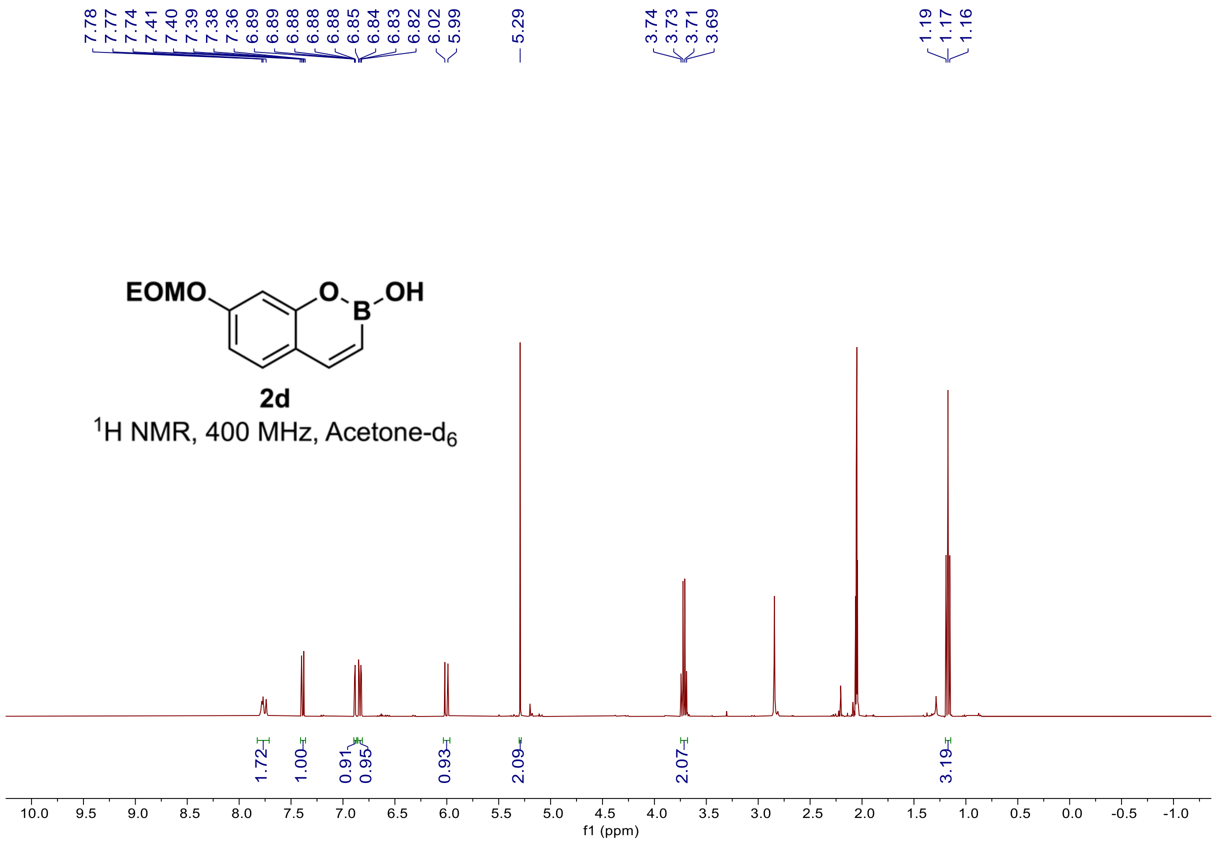
7.78 7.77 7.74 7.41 7.40 7.39 7.38 7.36 6.89 6.89 6.88 6.88 6.85 6.84 6.83 6.82 6.02 5.99
5.29
3.74 3.73 3.71 3.69
1.19 1.17 1.16
EOMO
O
B
OH
2d
1H NMR, 400 MHz, Acetone-d6
1.72 1.00 0.91 0.95 0.93 2.09 2.07 3.19
10.0 9.5 9.0 8.5 8.0 7.5 7.0 6.5 6.0 5.5 5.0 4.5 4.0 3.5 3.0 2.5 2.0 1.5 1.0 0.5 0.0 -0.5 -1.0
f1 (ppm)

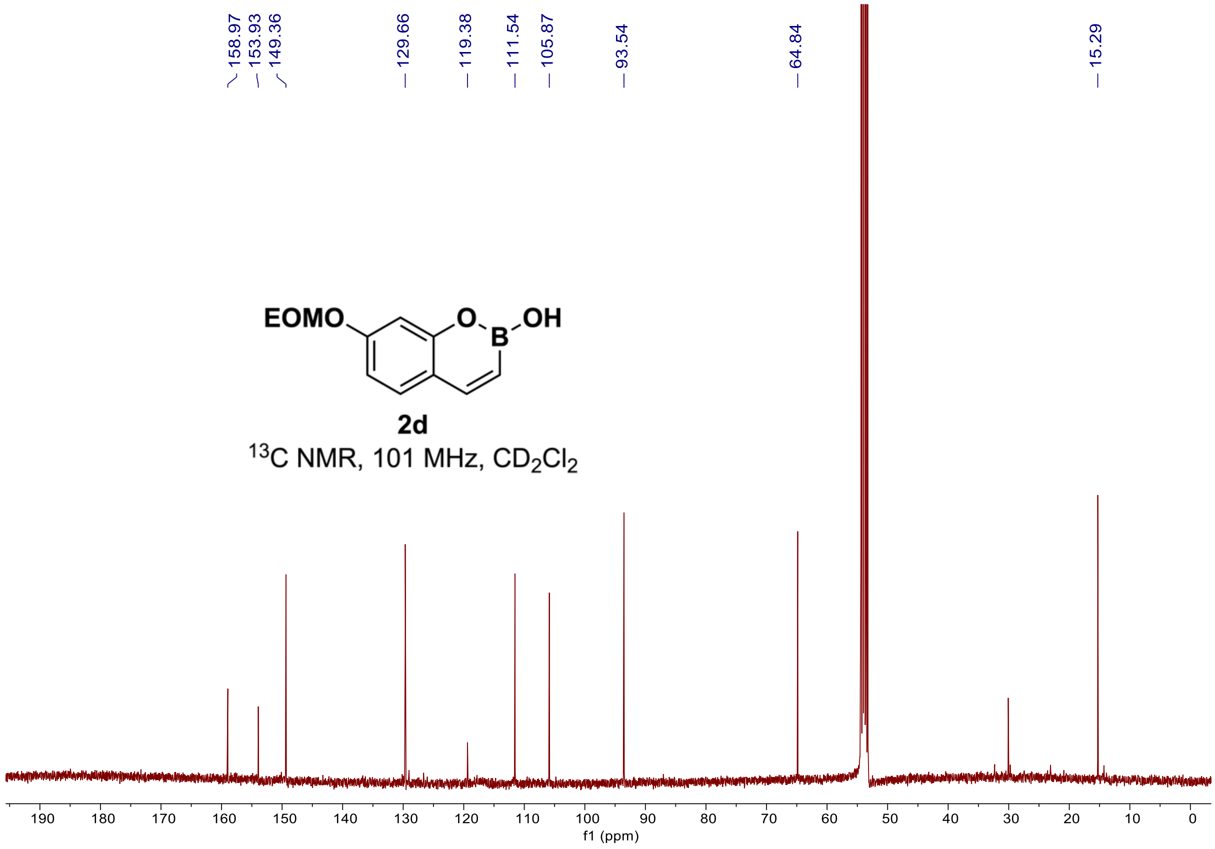
158.97 153.93 149.36
129.66
119.38
111.54
105.87
93.54
64.84
15.29
EOMO
O
B
OH
2d
13C NMR, 101 MHz, CD2Cl2
190 180 170 160 150 140 130 120 110 100 90 80 70 60 50 40 30 20 10 0
f1 (ppm)

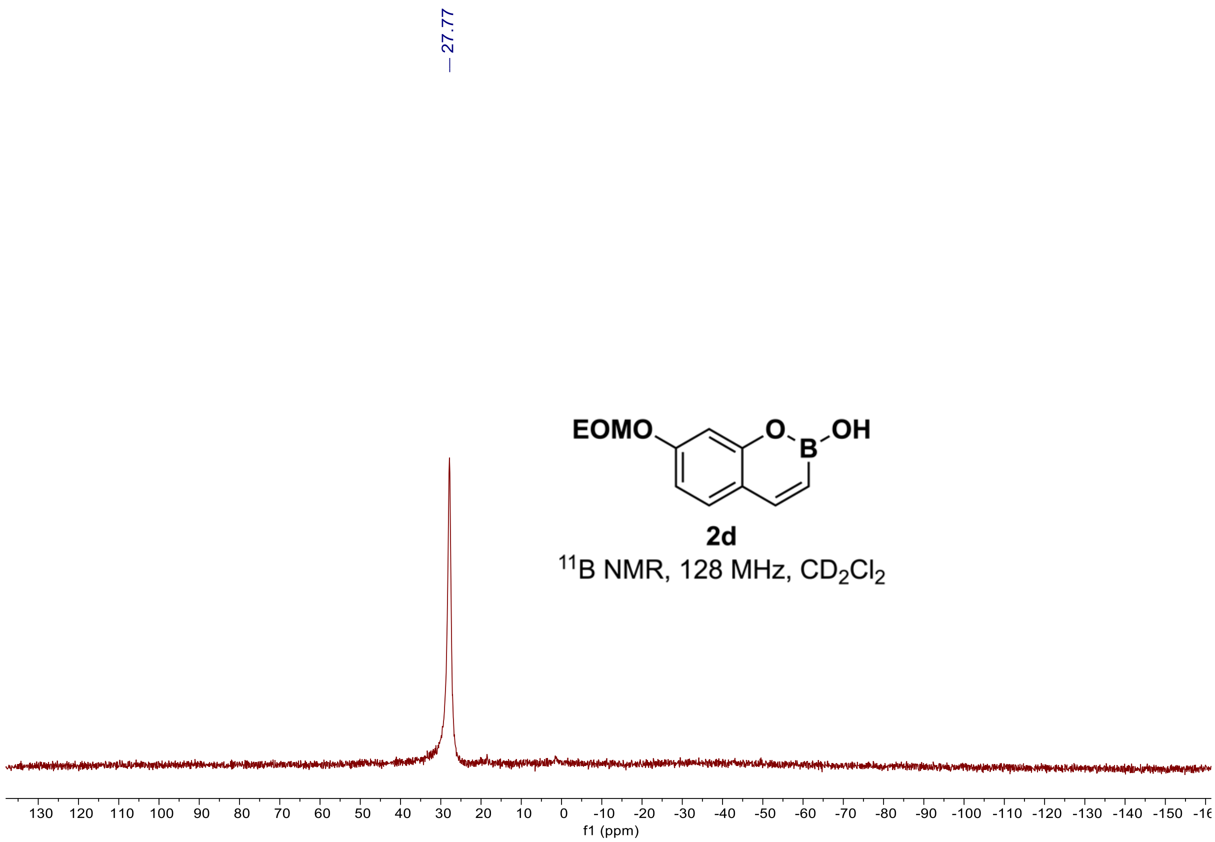
EOMO
O
B
OH
2d
^{11}B NMR, 128 MHz, CD_2Cl_2
27.77
f1 (ppm)

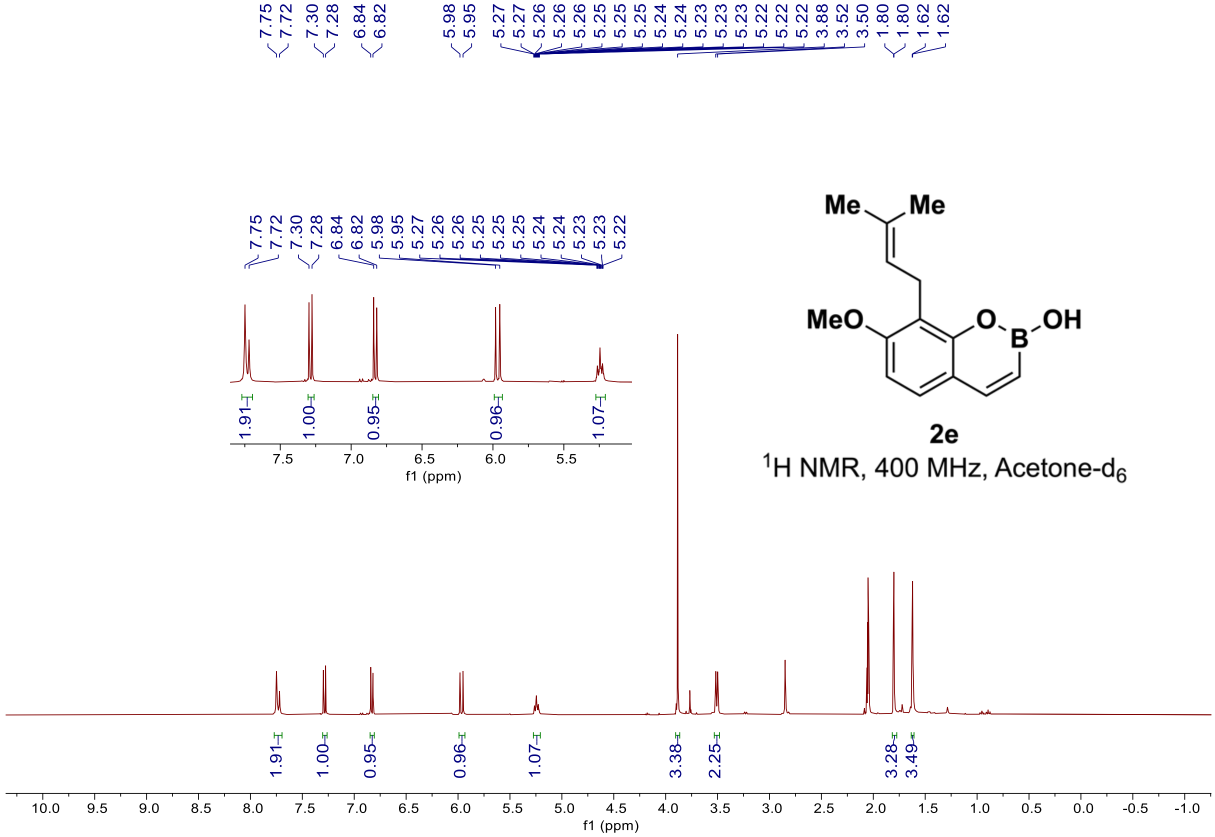
Me
Me
MeO
O
B
OH
2e
^{1}H NMR, 400 MHz, Acetone-d_6
f1 (ppm)

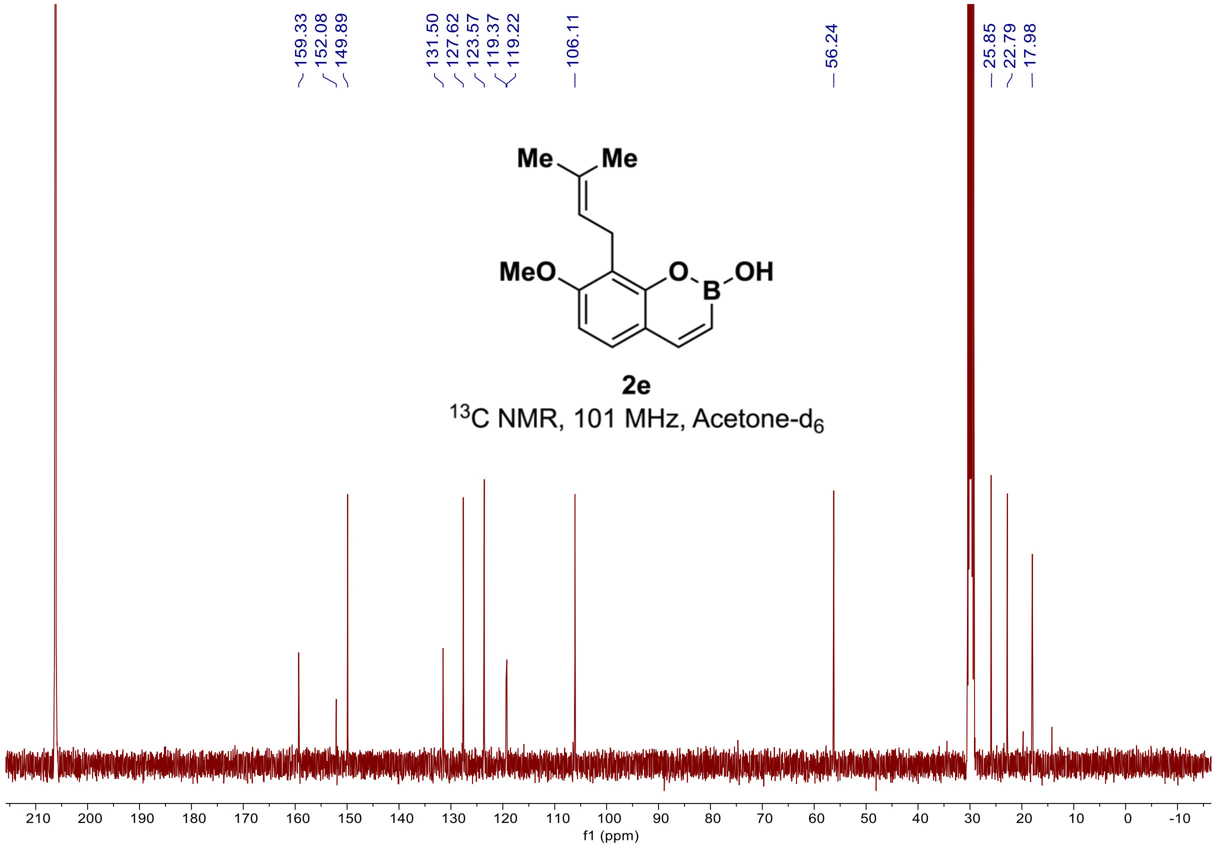
159.33
152.08
149.89
131.50
127.62
123.57
119.37
119.22
106.11
56.24
25.85
22.79
17.98
Me
Me
MeO
O
B
OH
2e
^{13}C NMR, 101 MHz, Acetone-d_6
f1 (ppm)

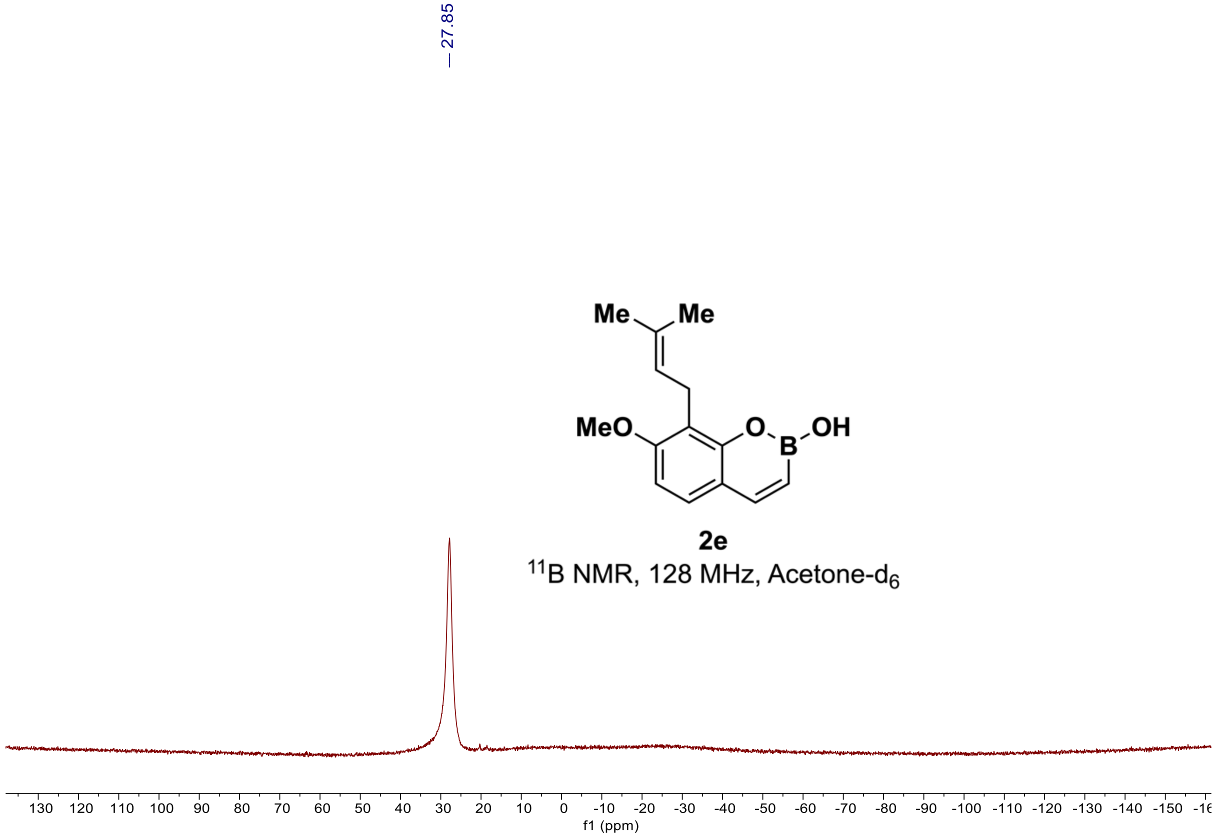
27.85
Me
Me
MeO
O
B
OH
2e
^{11}B NMR, 128 MHz, Acetone-d_6
f1 (ppm)

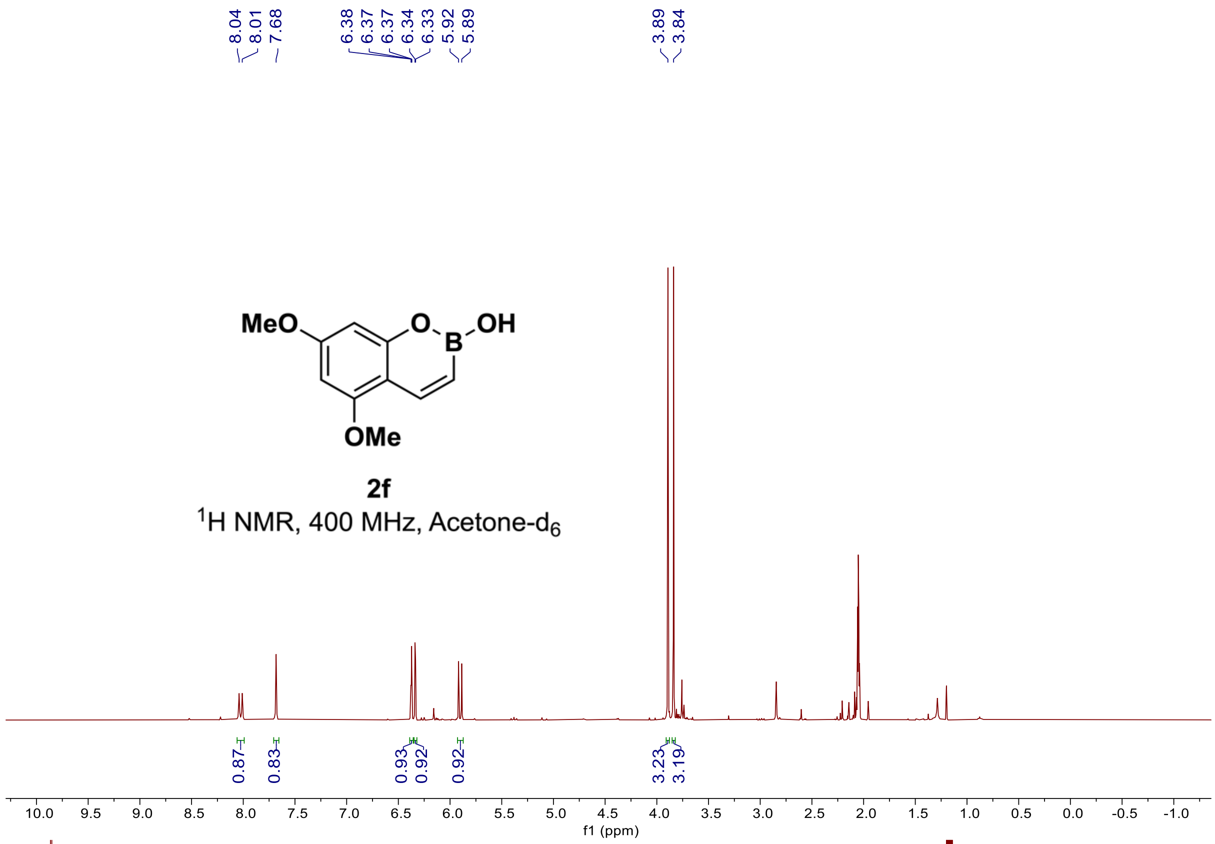
8.04
8.01
7.68
6.38
6.37
6.37
6.34
6.33
5.92
5.89
3.89
3.84
MeO
O
B
OH
OMe
2f
1H NMR, 400 MHz, Acetone-d6
0.87
0.83
0.93
0.92
0.92
3.23
3.19
f1 (ppm)

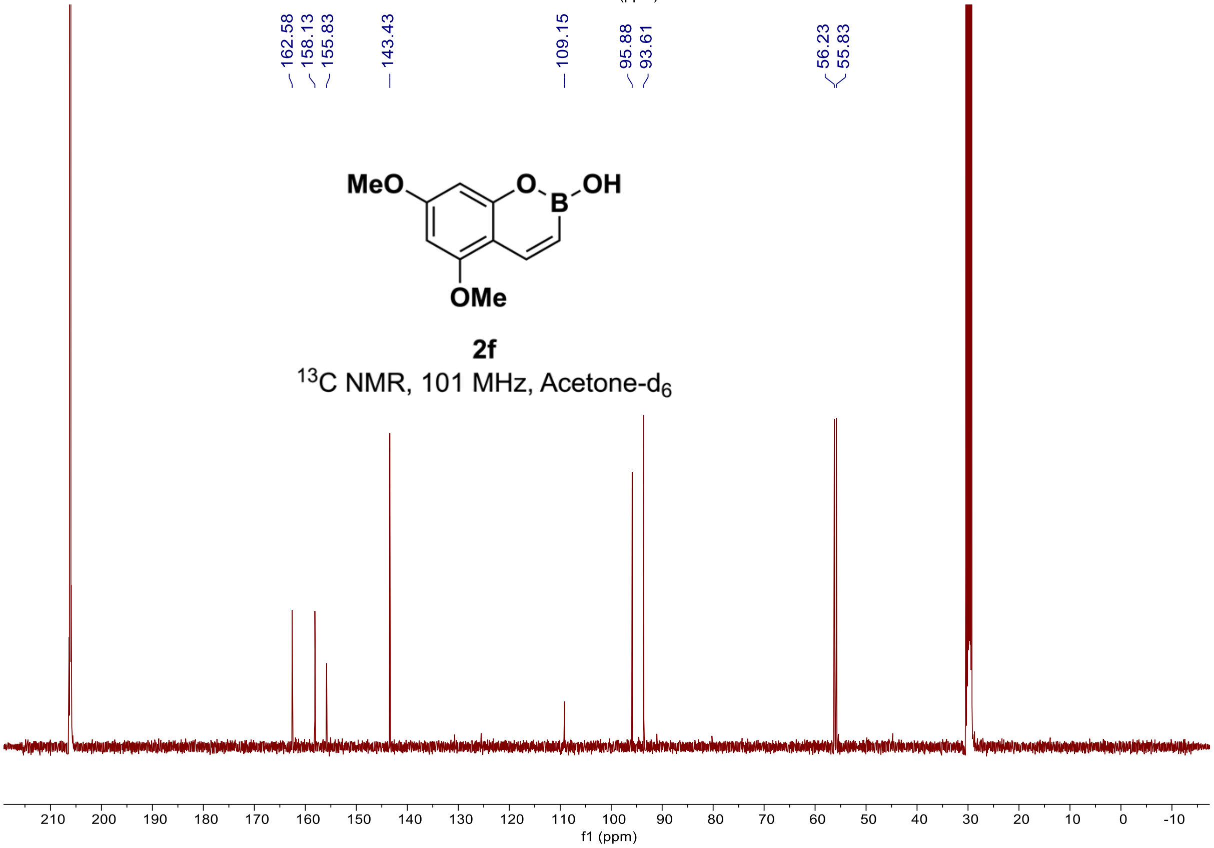
162.58
158.13
155.83
143.43
109.15
95.88
93.61
56.23
55.83
MeO
O
B
OH
OMe
2f
13C NMR, 101 MHz, Acetone-d6
f1 (ppm)

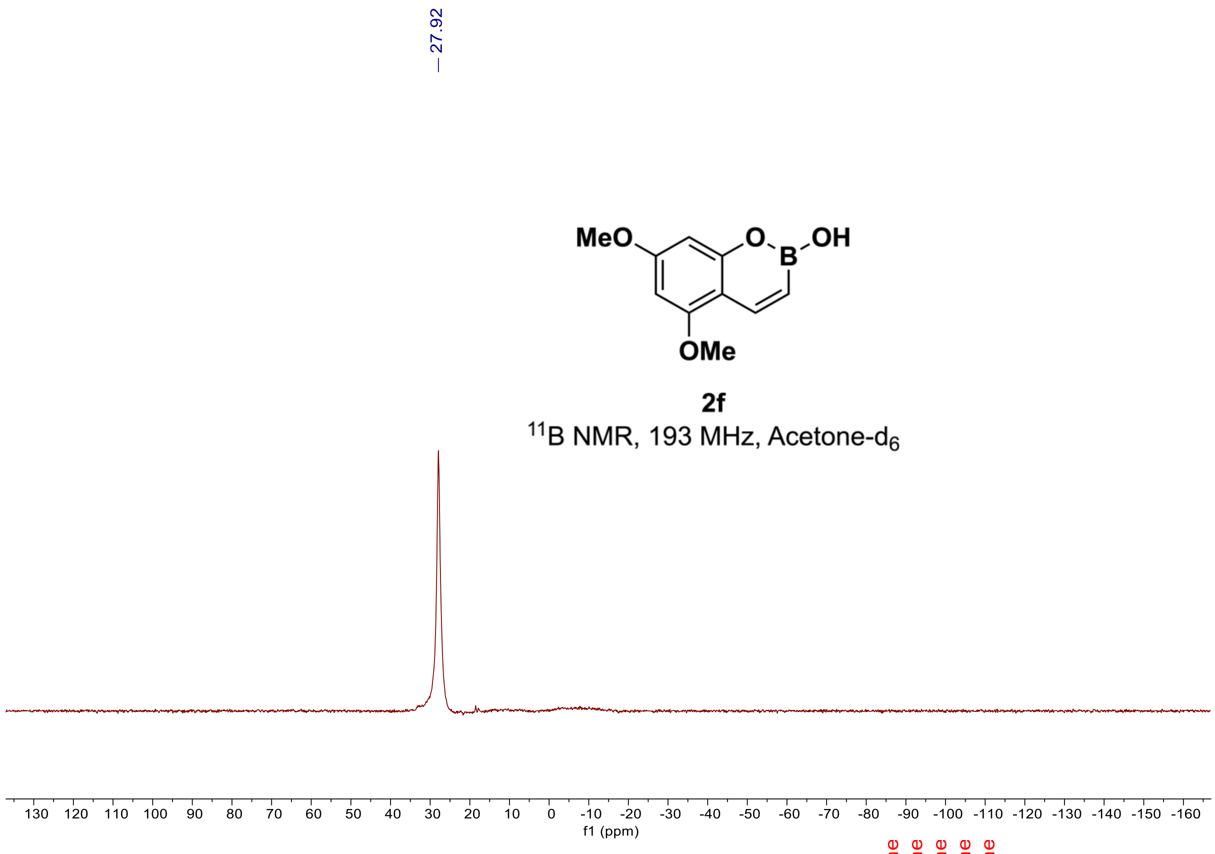
27.92
MeO
O
B
OH
OMe
2f
11B NMR, 193 MHz, Acetone-d6
130 120 110 100 90 80 70 60 50 40 30 20 10 0 -10 -20 -30 -40 -50 -60 -70 -80 -90 -100 -110 -120 -130 -140 -150 -160
f1 (ppm)

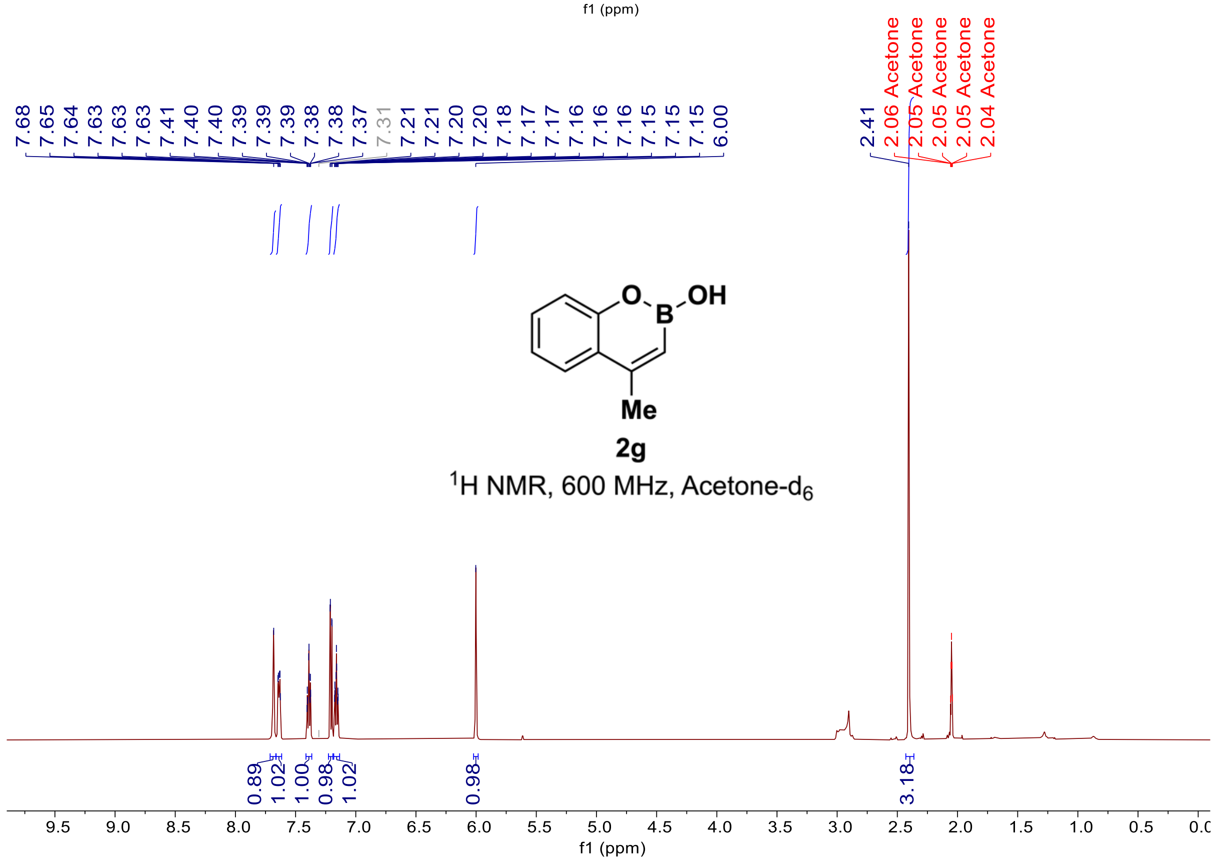
7.68 7.65 7.64 7.63 7.63 7.63 7.41 7.40 7.39 7.39 7.39 7.38 7.38 7.37 7.31 7.21 7.21 7.20 7.20 7.18 7.17 7.17 7.16 7.16 7.15 7.15 7.15 6.00
2.41
2.06 Acetone
2.05 Acetone
2.05 Acetone
2.05 Acetone
2.04 Acetone
O
B
OH
Me
2g
1H NMR, 600 MHz, Acetone-d6
0.89 1.02 1.00 0.98 1.02 0.98 3.18
9.5 9.0 8.5 8.0 7.5 7.0 6.5 6.0 5.5 5.0 4.5 4.0 3.5 3.0 2.5 2.0 1.5 1.0 0.5
f1 (ppm)

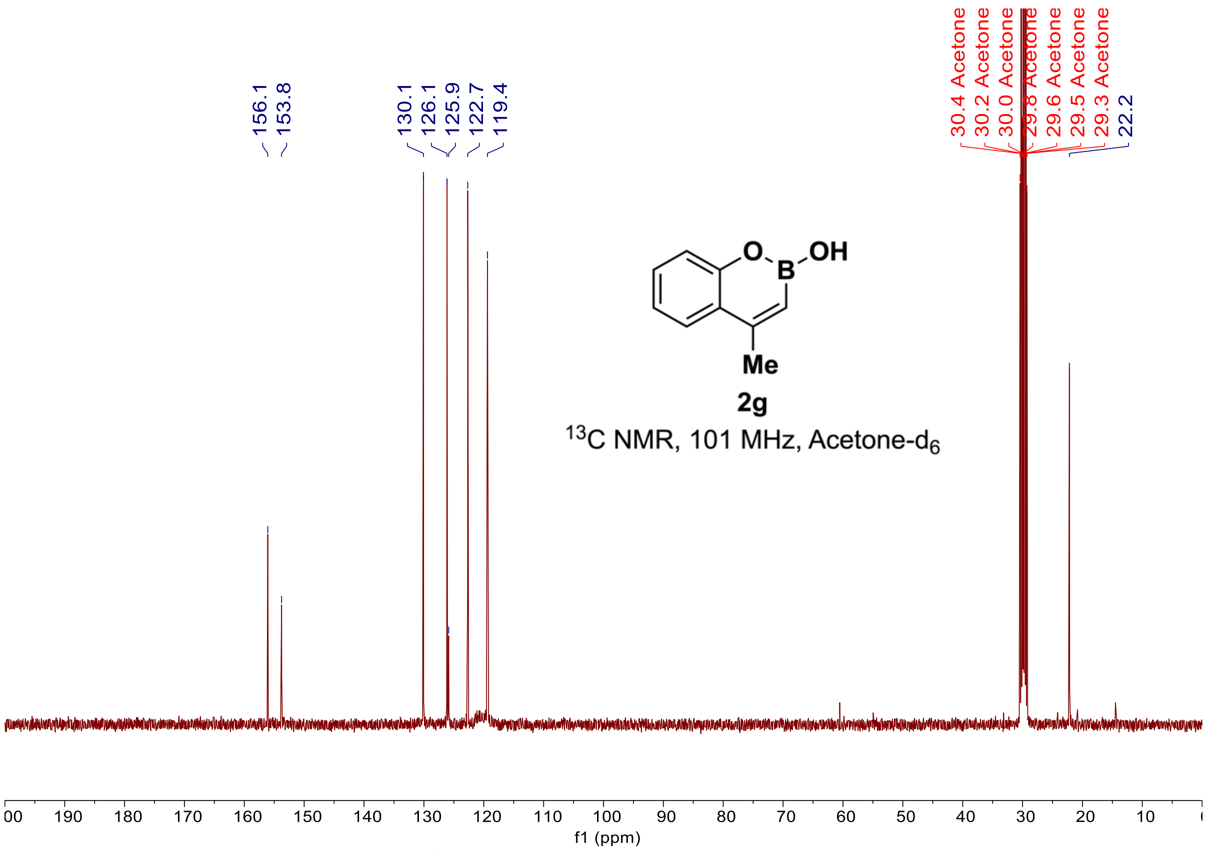
156.1
153.8
130.1
126.1
125.9
122.7
119.4
30.4 Acetone
30.2 Acetone
30.0 Acetone
29.8 Acetone
29.6 Acetone
29.5 Acetone
29.3 Acetone
22.2
O
B
OH
Me
2g
13C NMR, 101 MHz, Acetone-d6
f1 (ppm)

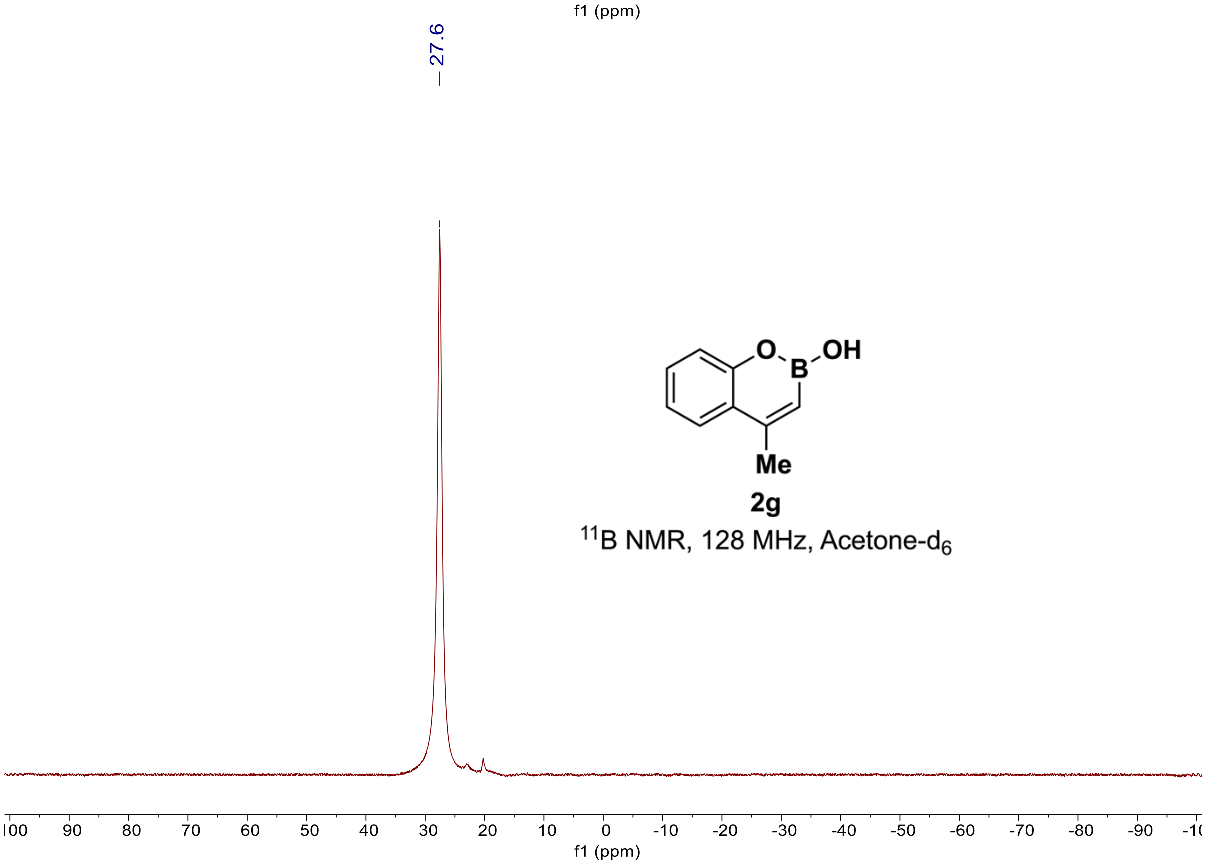
27.6
O
B
OH
Me
2g
11B NMR, 128 MHz, Acetone-d6
f1 (ppm)

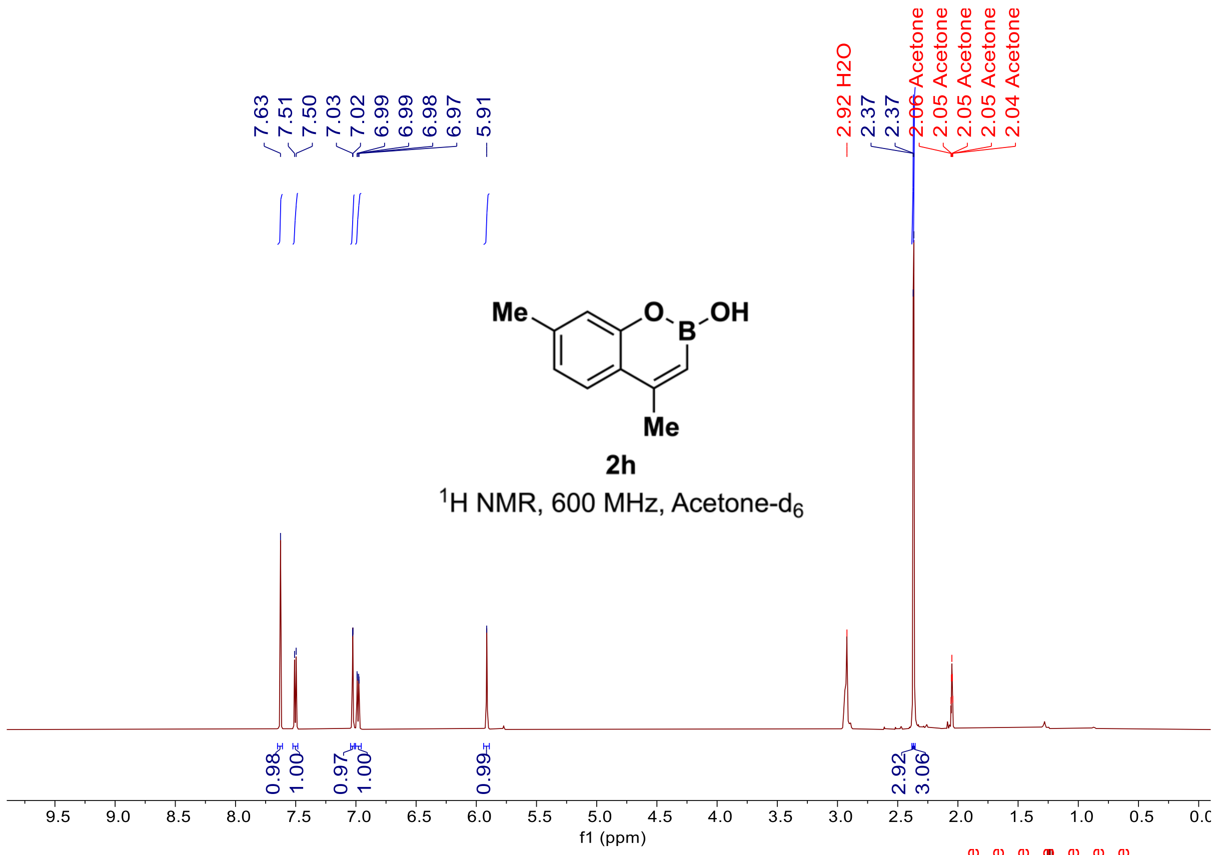
7.63
7.51
7.50
7.03
7.02
6.99
6.99
6.98
6.97
5.91
2.92 H2O
2.37
2.37
2.06 Acetone
2.05 Acetone
2.05 Acetone
2.05 Acetone
2.04 Acetone
Me
O
B
OH
Me
2h
1H NMR, 600 MHz, Acetone-d6
0.98
1.00
0.97
1.00
0.99
2.92
3.06
9.5
9.0
8.5
8.0
7.5
7.0
6.5
6.0
5.5
5.0
4.5
4.0
3.5
3.0
2.5
2.0
1.5
1.0
0.5
f1 (ppm)

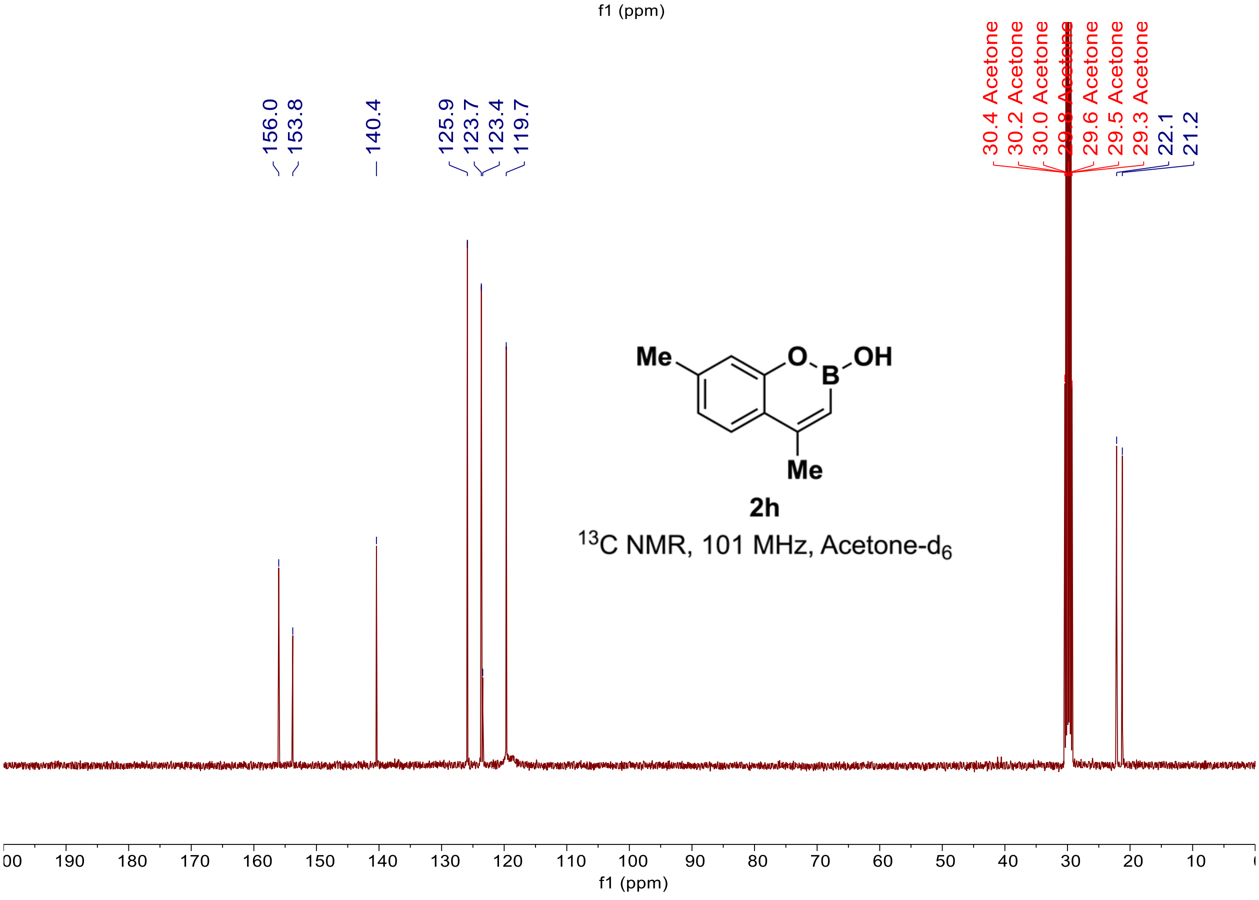
156.0
153.8
140.4
125.9
123.7
123.4
119.7
30.4 Acetone
30.2 Acetone
30.0 Acetone
29.6 Acetone
29.5 Acetone
29.3 Acetone
22.1
21.2
Me
O
B
OH
Me
2h
13C NMR, 101 MHz, Acetone-d6
190
180
170
160
150
140
130
120
110
100
90
80
70
60
50
40
30
20
10
f1 (ppm)

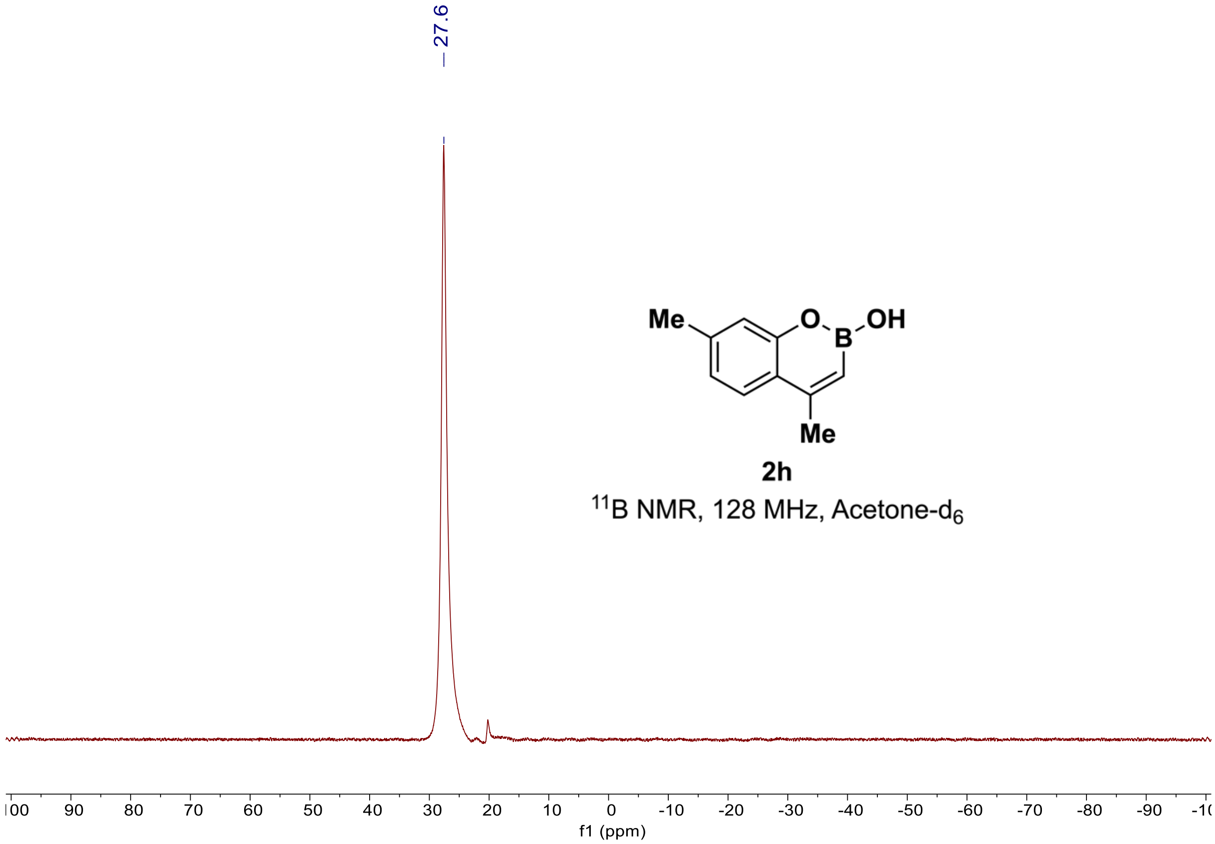
27.6
Me
O
B
OH
Me
2h
^{11}B NMR, 128 MHz, Acetone-d$_6$
100 90 80 70 60 50 40 30 20 10 0 -10 -20 -30 -40 -50 -60 -70 -80 -90 -100
f1 (ppm)

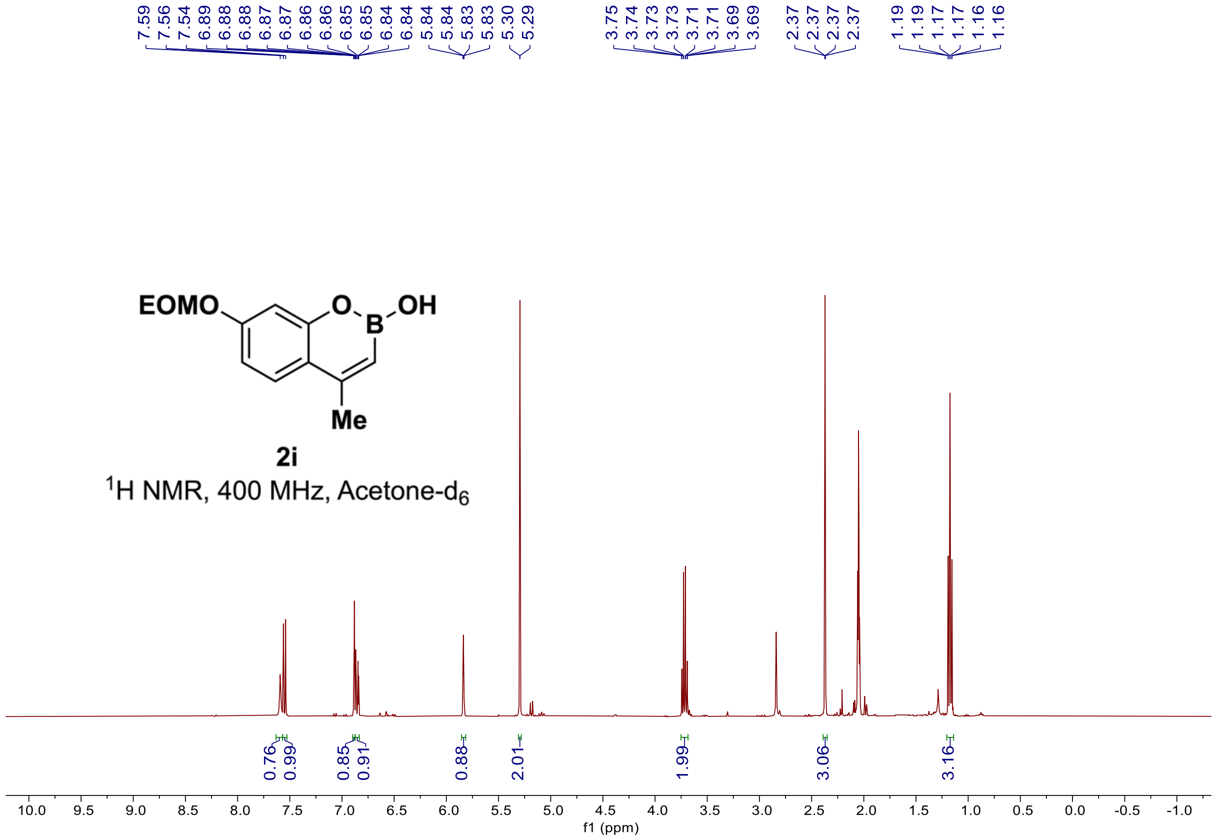
7.59 7.56 7.54 6.89 6.88 6.88 6.87 6.87 6.86 6.86 6.85 6.85 6.84 6.84 5.84 5.84 5.83 5.83 5.30 5.29 3.75 3.74 3.73 3.73 3.71 3.71 3.69 3.69 2.37 2.37 2.37 2.37 1.19 1.19 1.17 1.17 1.16 1.16
EOMO
O
B
OH
Me
2i
^{1}H NMR, 400 MHz, Acetone-d$_6$
0.76 0.99 0.85 0.91 0.88 2.01 1.99 3.06 3.16
10.0 9.5 9.0 8.5 8.0 7.5 7.0 6.5 6.0 5.5 5.0 4.5 4.0 3.5 3.0 2.5 2.0 1.5 1.0 0.5 0.0 -0.5 -1.0
f1 (ppm)

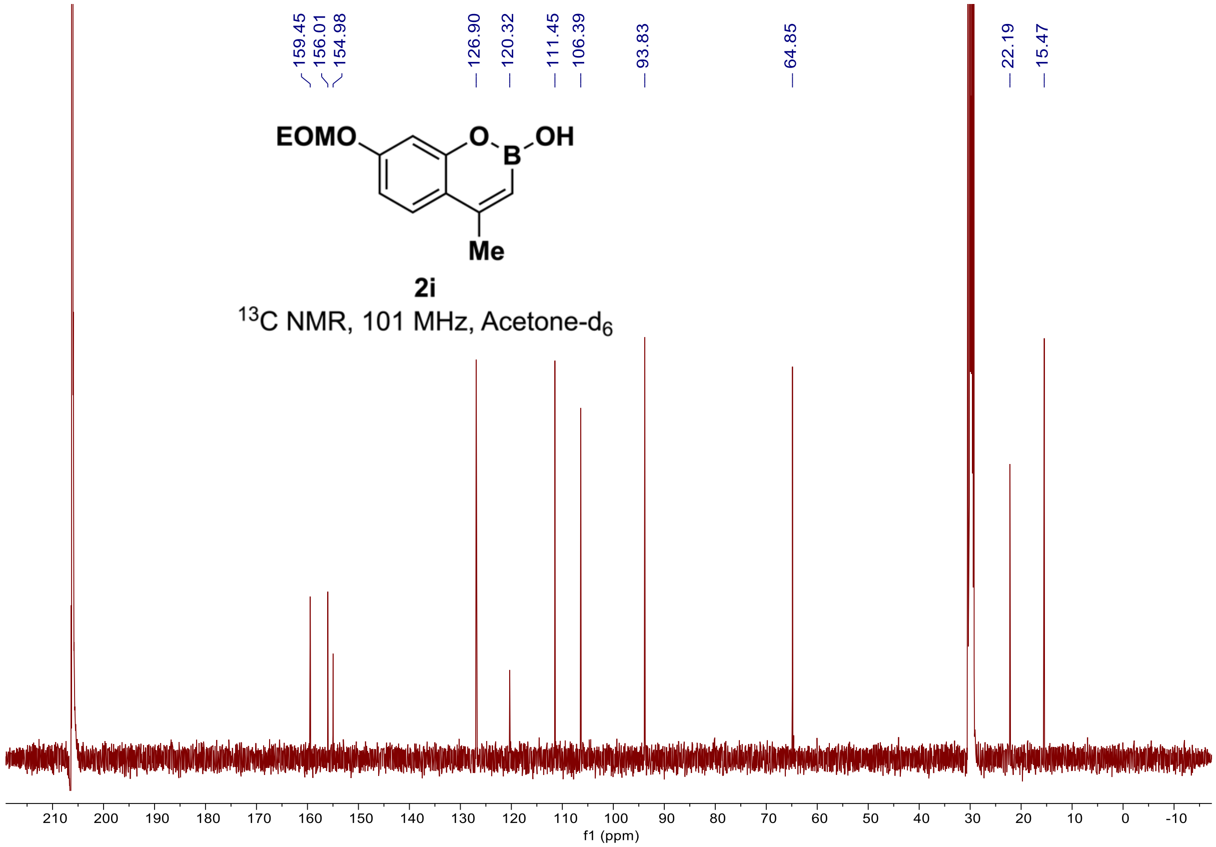
159.45
156.01
154.98
126.90
120.32
111.45
106.39
93.83
64.85
22.19
15.47
EOMO
O
B
OH
Me
2i
^{13}C NMR, 101 MHz, Acetone-d$_6$
210 200 190 180 170 160 150 140 130 120 110 100 90 80 70 60 50 40 30 20 10 0 -10
f1 (ppm)

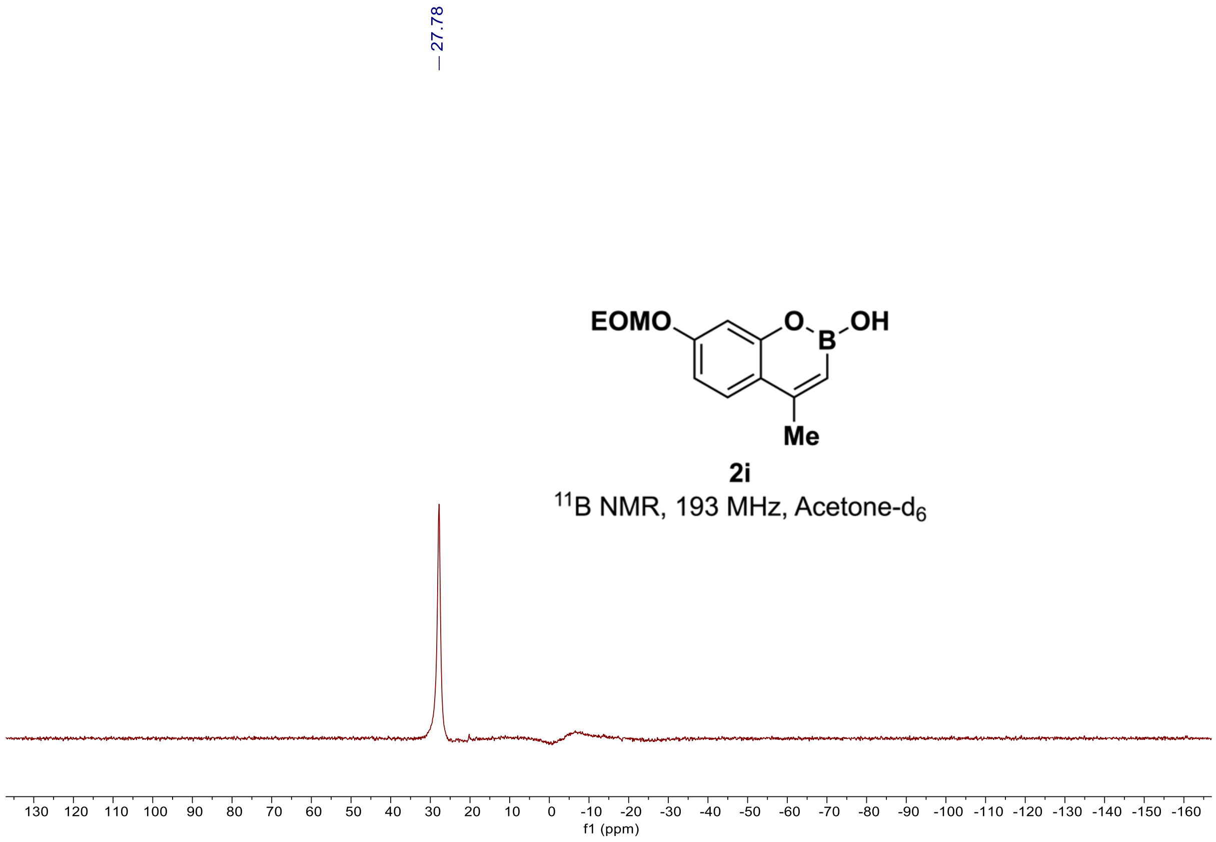
27.78
EOMO
O
B
OH
Me
2i
^{11}B NMR, 193 MHz, Acetone-d$_6$
130 120 110 100 90 80 70 60 50 40 30 20 10 0 -10 -20 -30 -40 -50 -60 -70 -80 -90 -100 -110 -120 -130 -140 -150 -160
f1 (ppm)

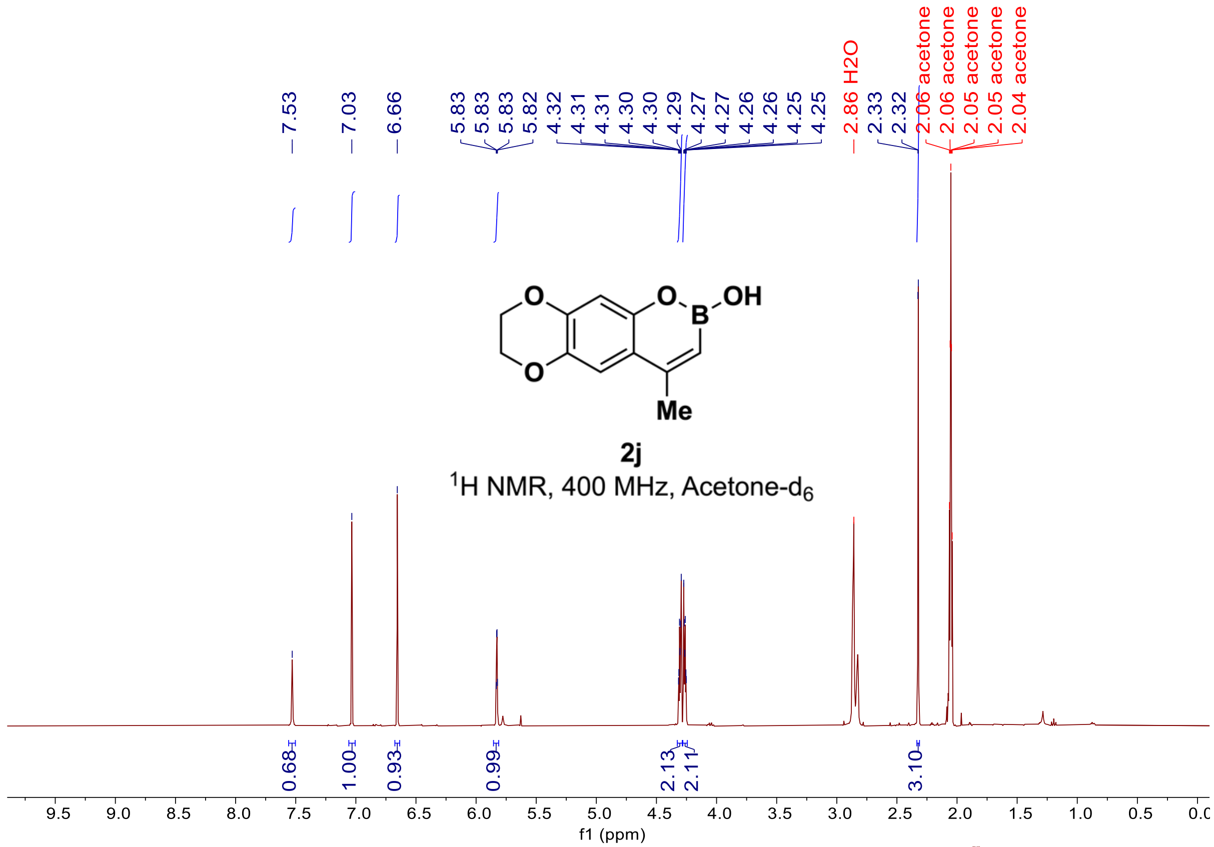
7.53
7.03
6.66
5.83
5.83
5.83
5.82
4.32
4.31
4.31
4.30
4.30
4.29
4.27
4.27
4.26
4.26
4.25
4.25
2.86 H2O
2.33
2.32
2.06 acetone
2.06 acetone
2.05 acetone
2.05 acetone
2.04 acetone
OH
B
O
O
O
Me
2j
^{1}H NMR, 400 MHz, Acetone-d_6
0.68
1.00
0.93
0.99
2.13
2.11
3.10
f1 (ppm)

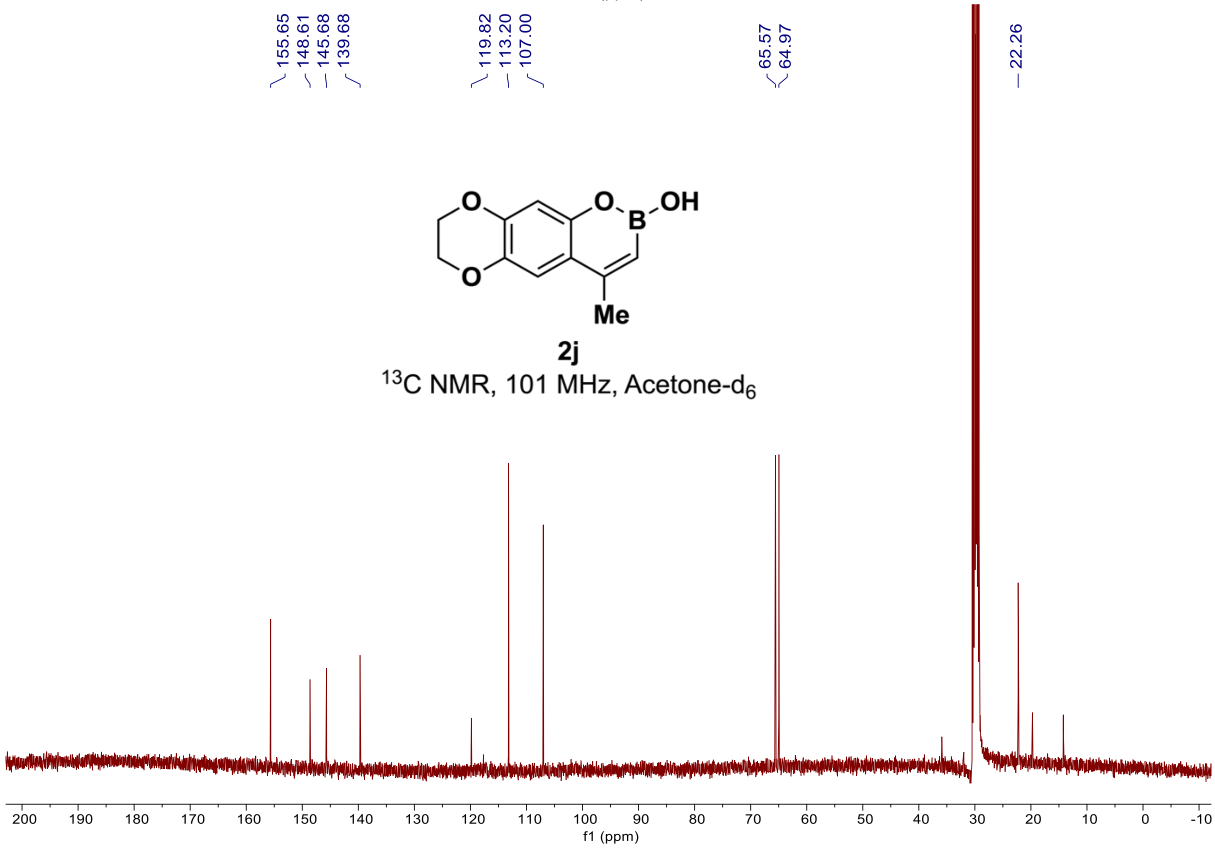
155.65
148.61
145.68
139.68
119.82
113.20
107.00
65.57
64.97
22.26
OH
B
O
O
O
Me
2j
^{13}C NMR, 101 MHz, Acetone-d_6
f1 (ppm)

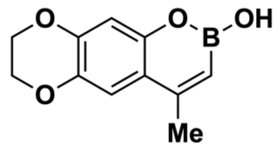

2j

^{11}B NMR, 128 MHz, Acetone-d$_6$

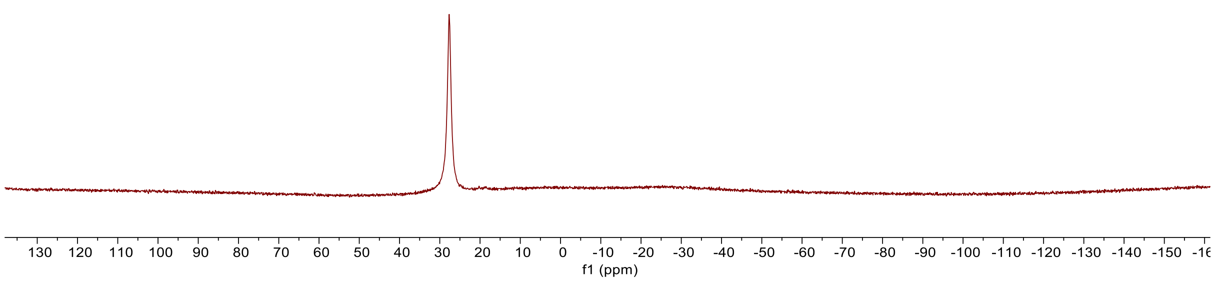

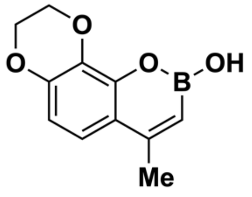

2k

^{1}H NMR, 400 MHz, Acetone-d$_6$

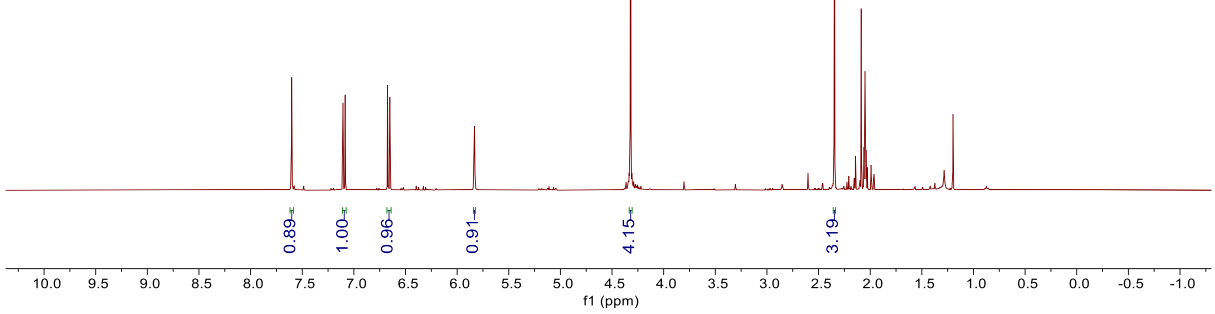

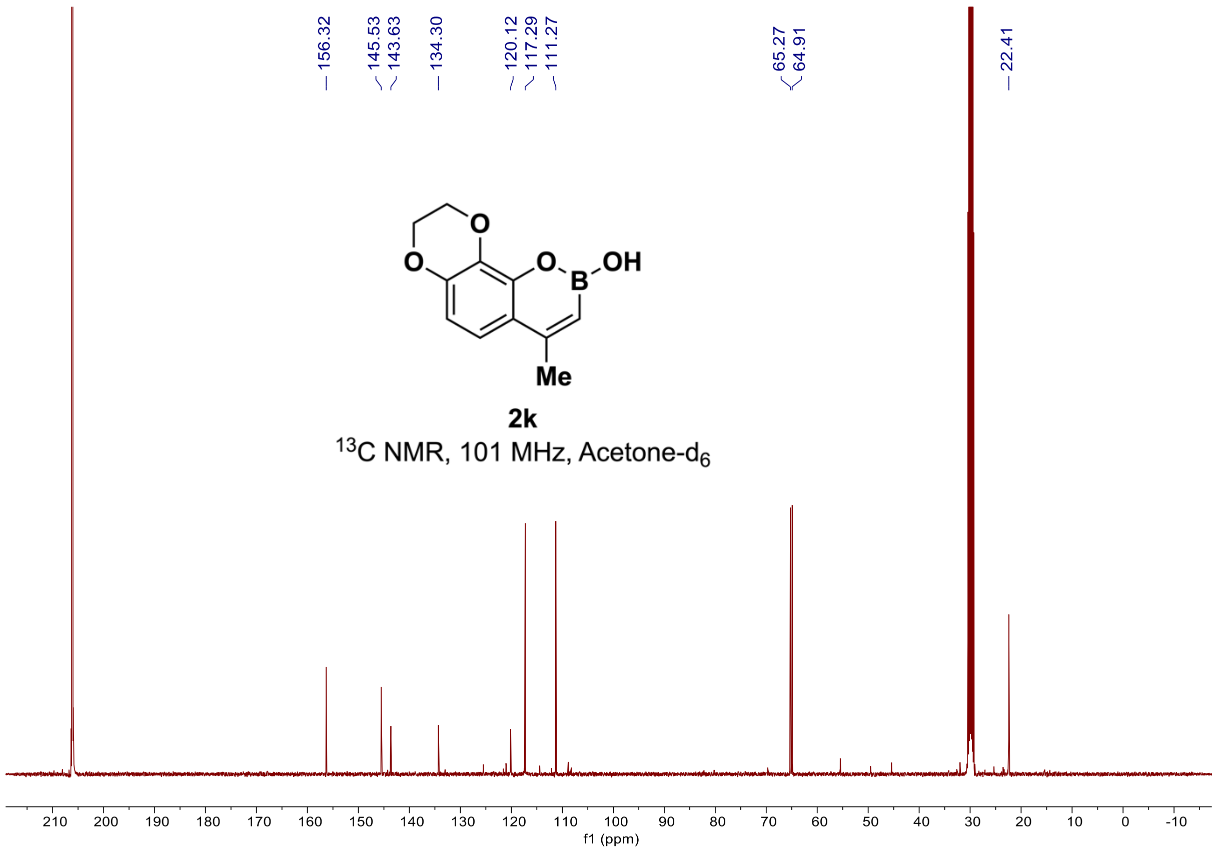

156.32
145.53
143.63
134.30
120.12
117.29
111.27
65.27
64.91
22.41
O
O
O
B
OH
Me
2k
^{13}C NMR, 101 MHz, Acetone-d$_6$
210 200 190 180 170 160 150 140 130 120 110 100 90 80 70 60 50 40 30 20 10 0 -10
f1 (ppm)

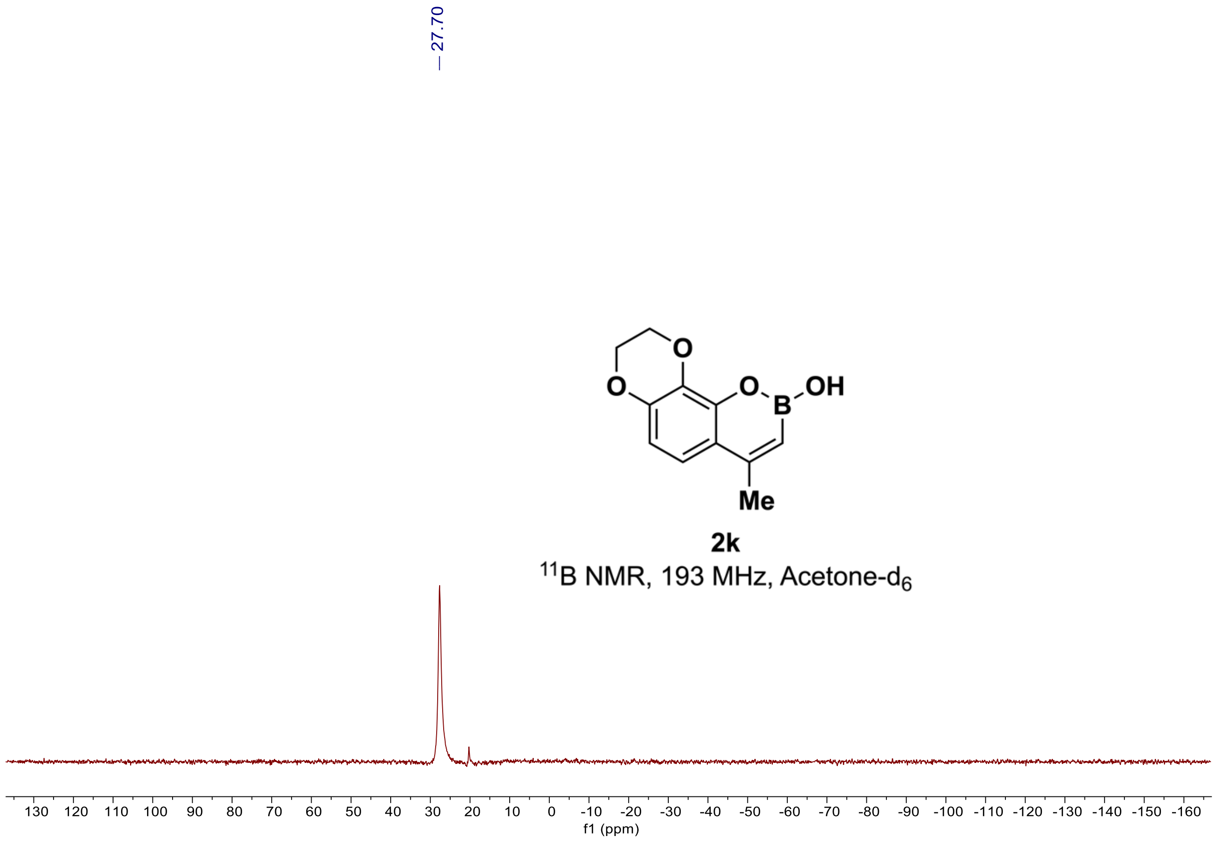

27.70
O
O
O
B
OH
Me
2k
^{11}B NMR, 193 MHz, Acetone-d$_6$
130 120 110 100 90 80 70 60 50 40 30 20 10 0 -10 -20 -30 -40 -50 -60 -70 -80 -90 -100 -110 -120 -130 -140 -150 -160
f1 (ppm)

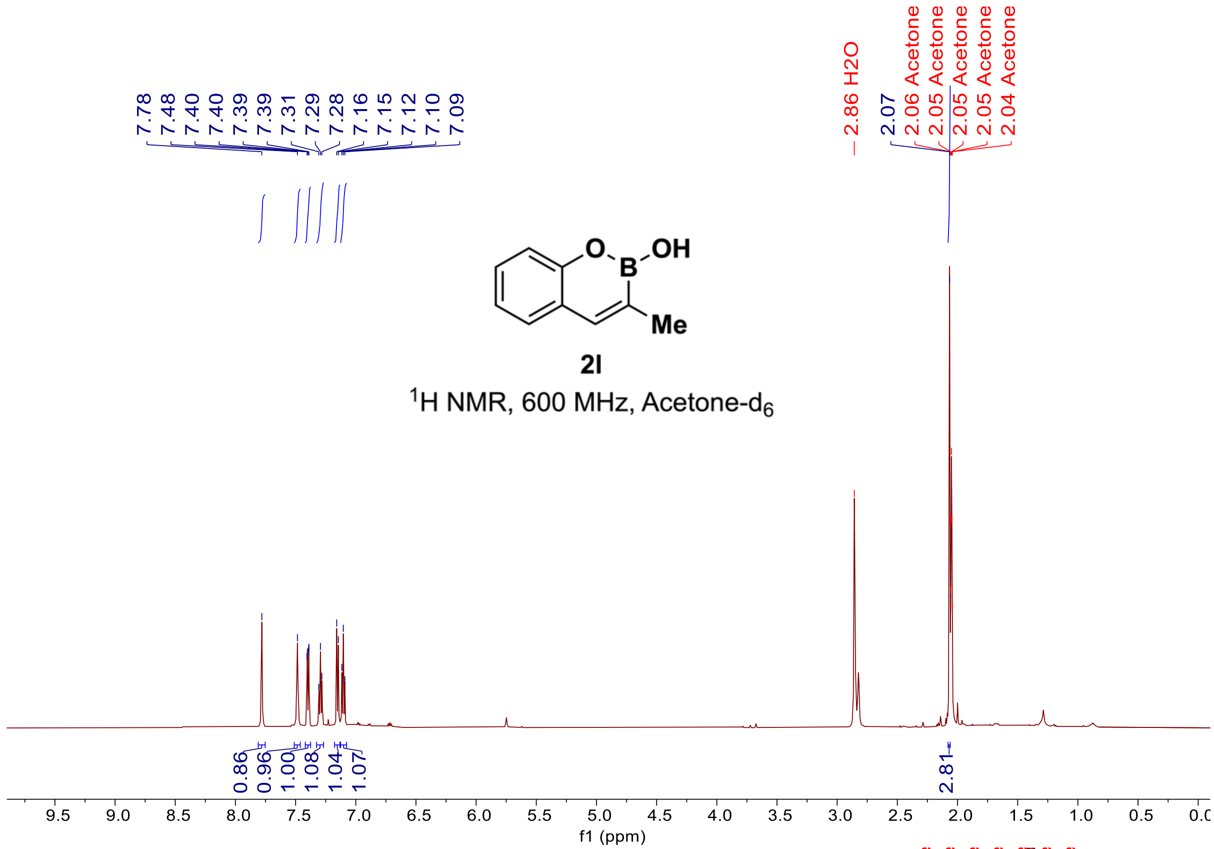
7.78
7.48
7.40
7.40
7.39
7.39
7.31
7.29
7.28
7.16
7.15
7.12
7.10
7.09
2.86 H2O
2.07
2.06 Acetone
2.05 Acetone
2.05 Acetone
2.05 Acetone
2.04 Acetone
O
B
OH
Me
2l
1H NMR, 600 MHz, Acetone-d6
0.86
0.96
1.00
1.08
1.04
1.07
2.81
f1 (ppm)

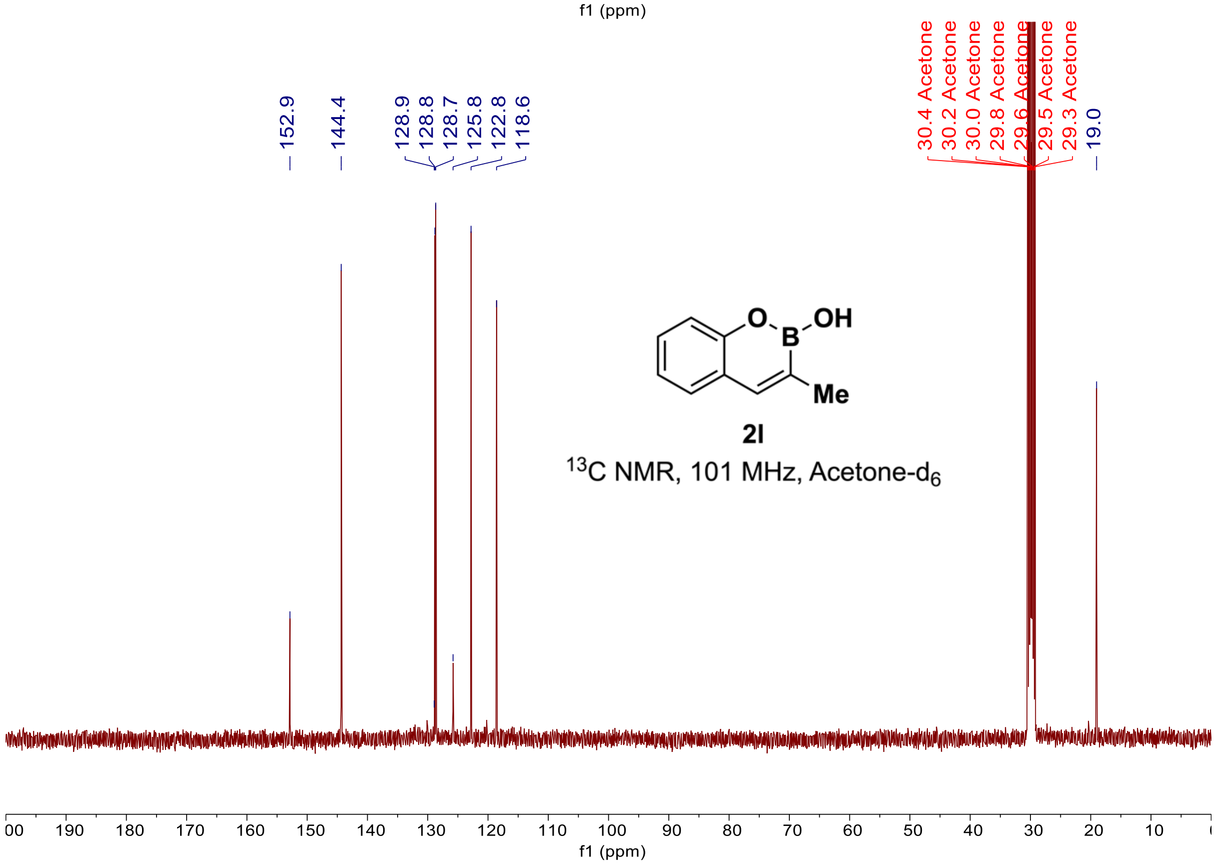
152.9
144.4
128.9
128.8
128.7
125.8
122.8
118.6
30.4 Acetone
30.2 Acetone
30.0 Acetone
29.8 Acetone
29.6 Acetone
29.5 Acetone
29.3 Acetone
19.0
O
B
OH
Me
2l
13C NMR, 101 MHz, Acetone-d6
f1 (ppm)

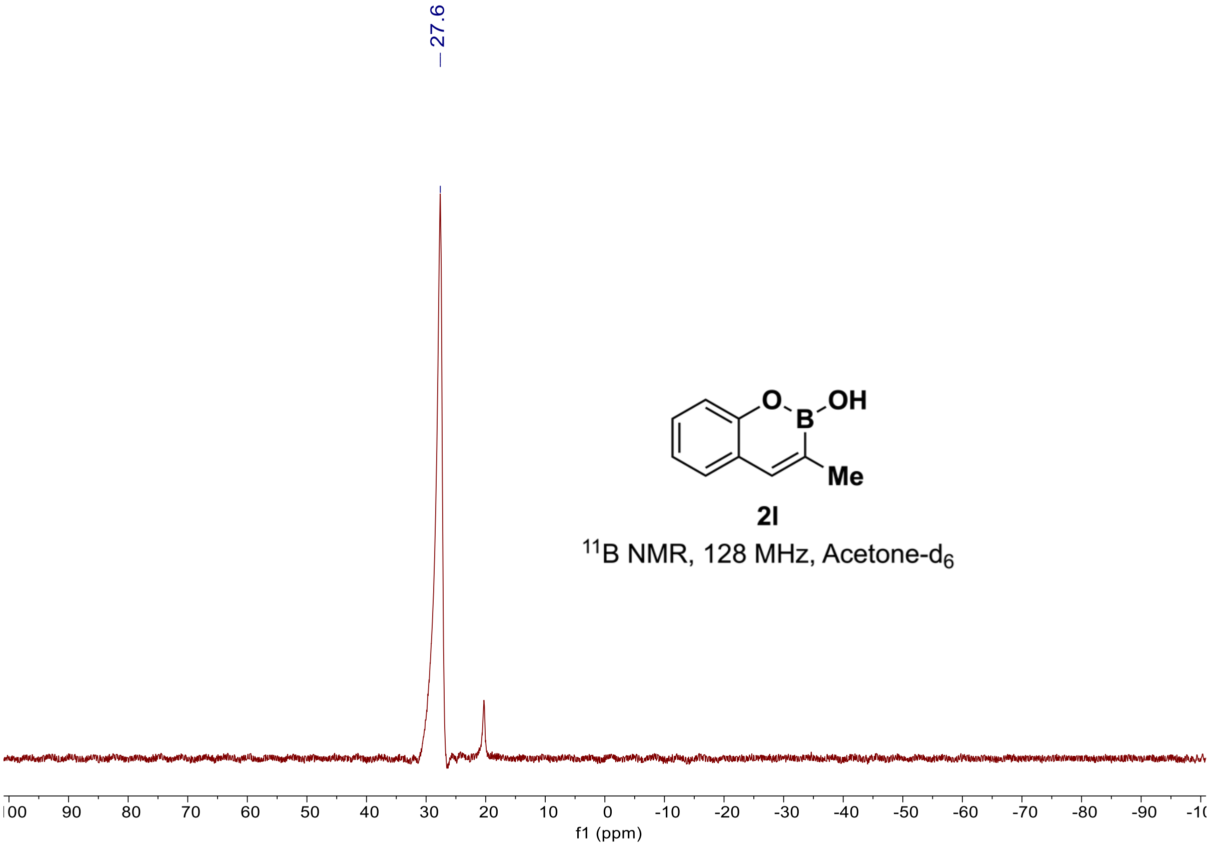

2l

^{11}B NMR, 128 MHz, Acetone-d_6

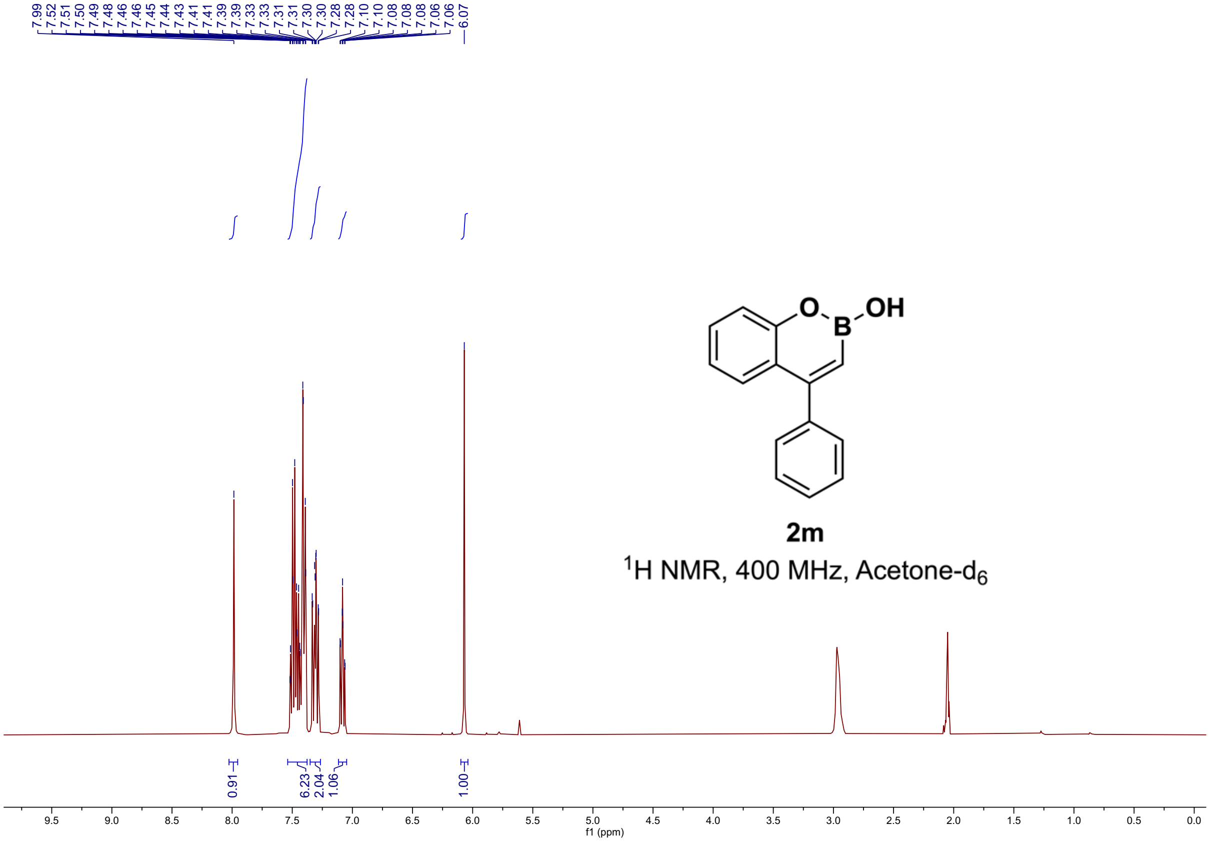

2m

^{1}H NMR, 400 MHz, Acetone-d_6

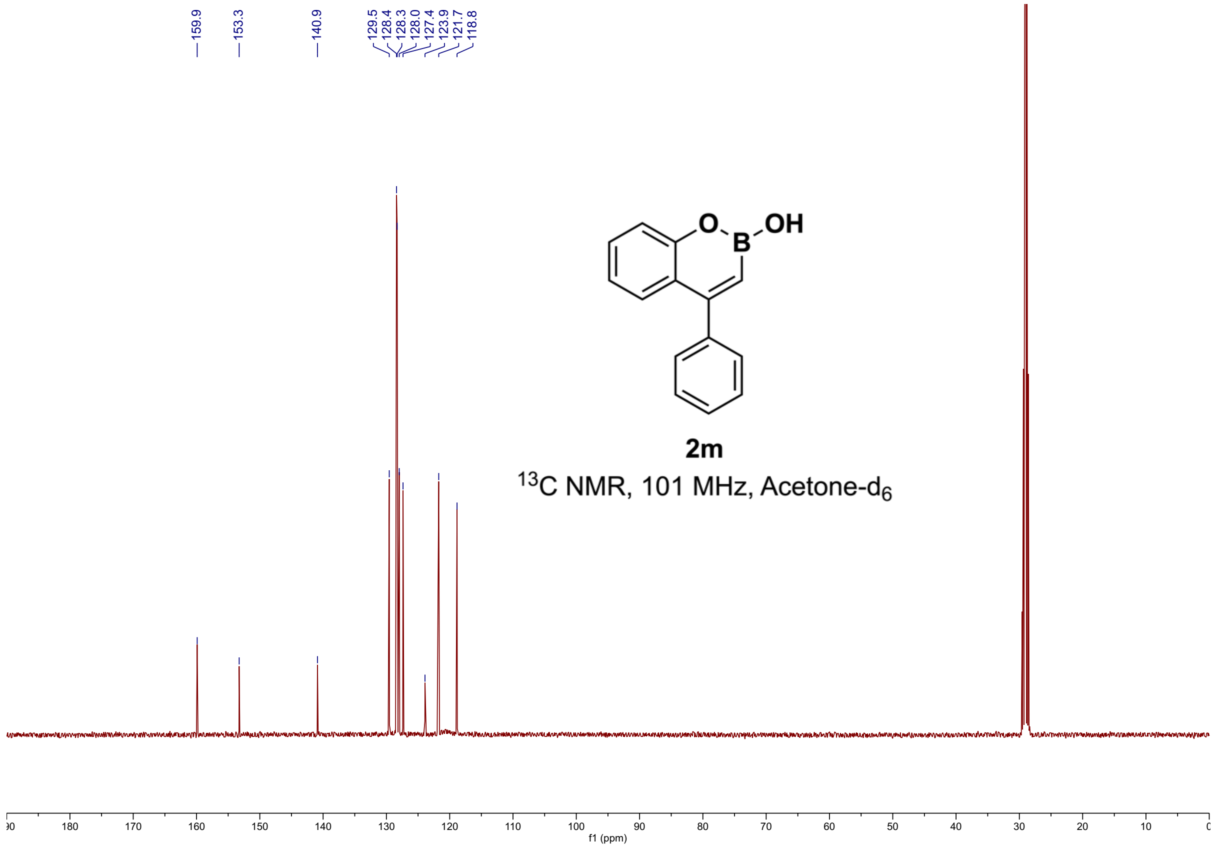
159.9
153.3
140.9
129.5
128.4
128.3
128.0
127.4
123.9
121.7
118.8
2m
^{13}C NMR, 101 MHz, Acetone-d_6
f1 (ppm)

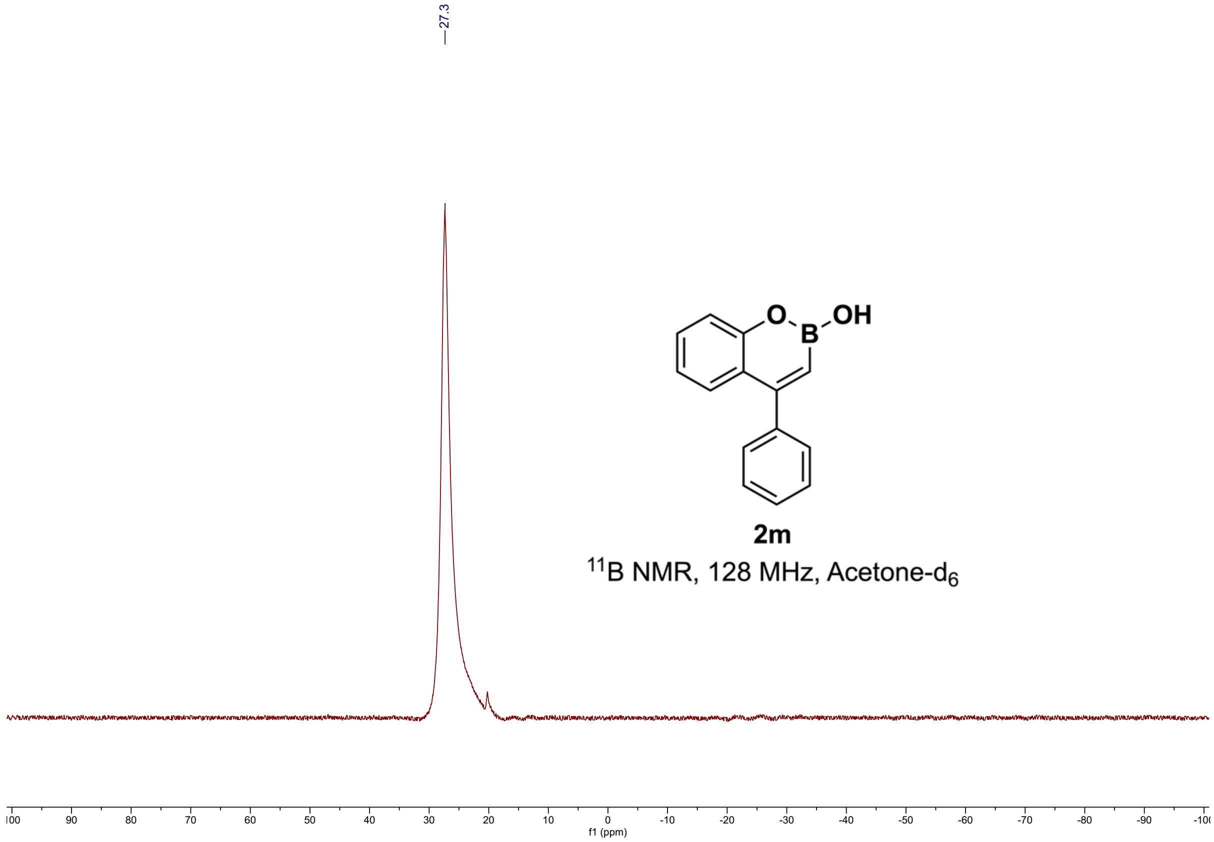
27.3
2m
^{11}B NMR, 128 MHz, Acetone-d_6
f1 (ppm)

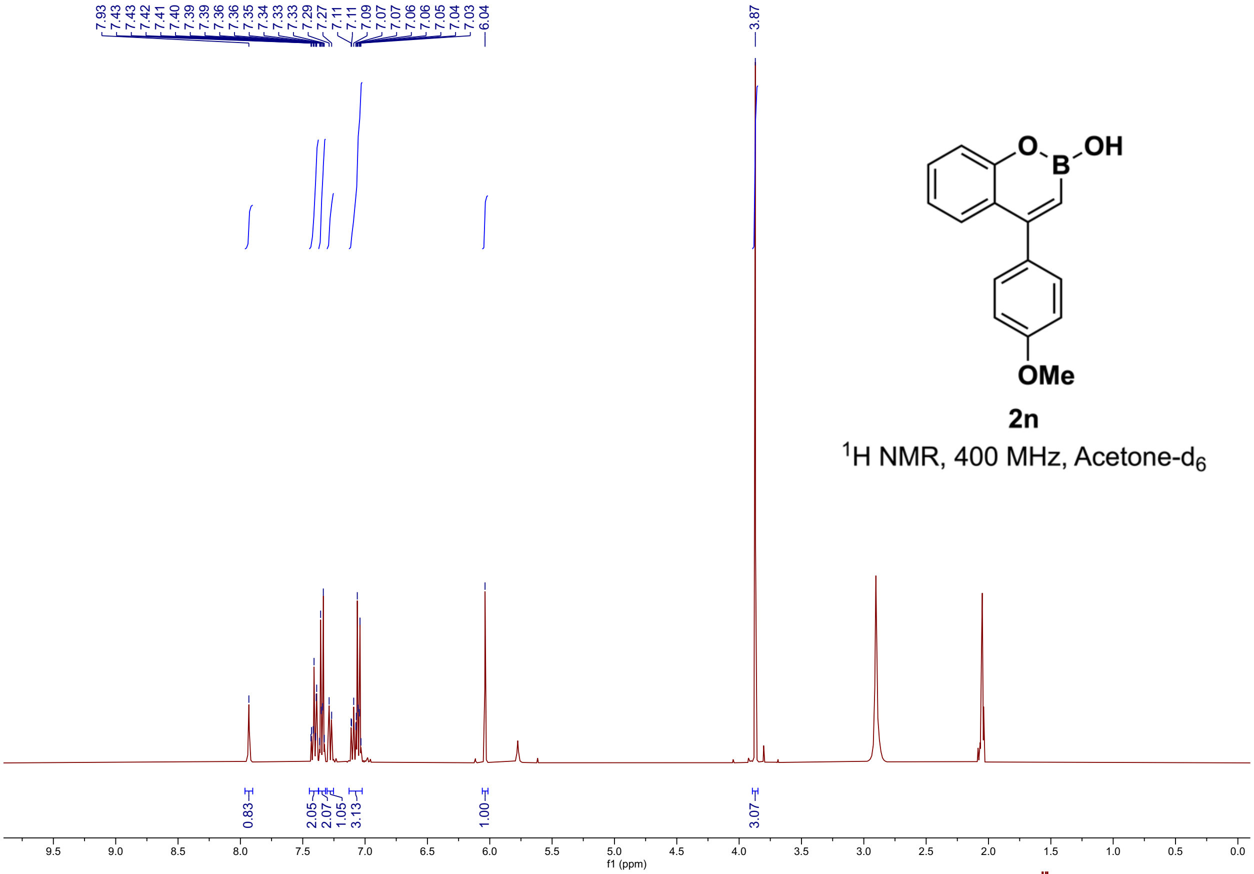
7.93 7.43 7.43 7.42 7.41 7.40 7.39 7.39 7.36 7.36 7.35 7.34 7.33 7.33 7.29 7.27 7.11 7.11 7.09 7.07 7.07 7.06 7.06 7.05 7.04 7.03
6.04
3.87
O
B
OH
OMe
2n
1H NMR, 400 MHz, Acetone-d6
0.83
2.05
2.07
1.05
3.13
1.00
3.07
9.5 9.0 8.5 8.0 7.5 7.0 6.5 6.0 5.5 5.0 4.5 4.0 3.5 3.0 2.5 2.0 1.5 1.0 0.5 0.0
f1 (ppm)

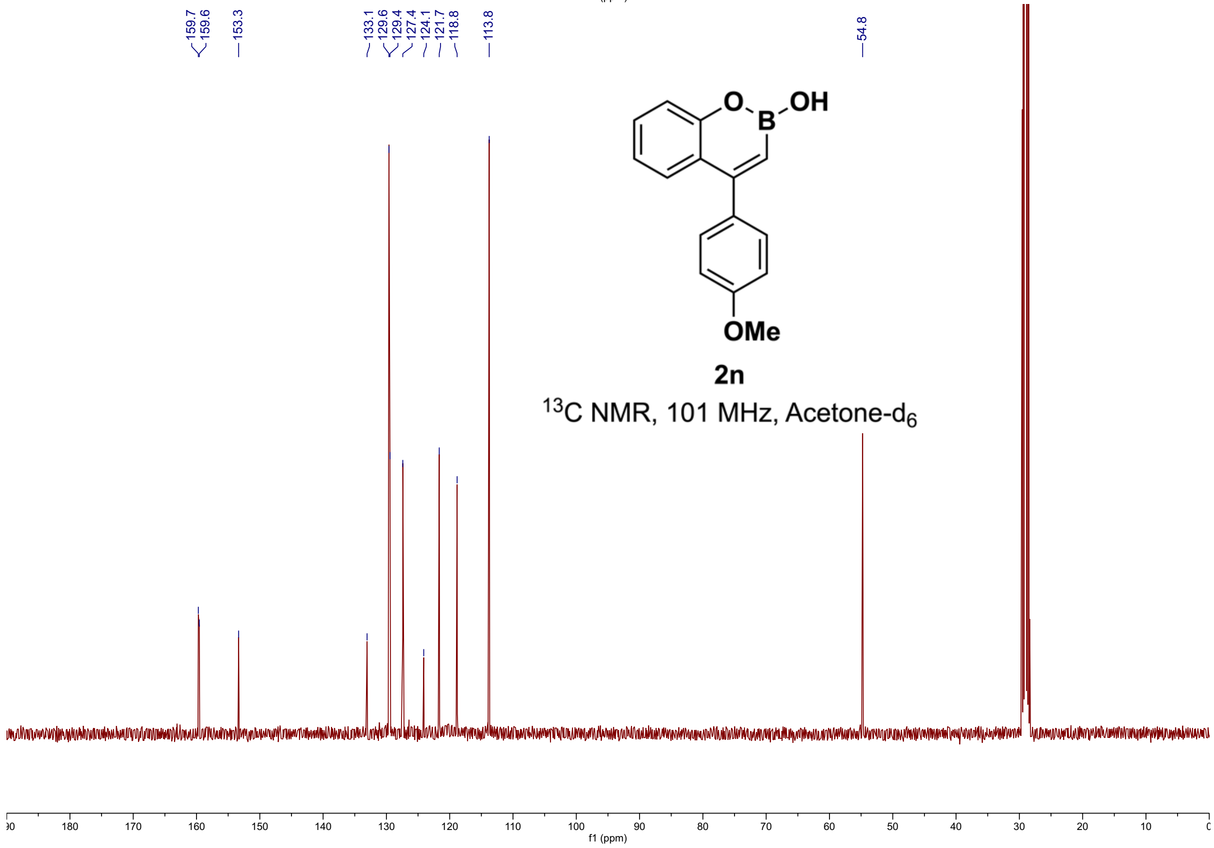
159.7 159.6
153.3
133.1 129.6 129.4 127.4 124.1 121.7 118.8
113.8
54.8
O
B
OH
OMe
2n
13C NMR, 101 MHz, Acetone-d6
180 170 160 150 140 130 120 110 100 90 80 70 60 50 40 30 20 10
f1 (ppm)

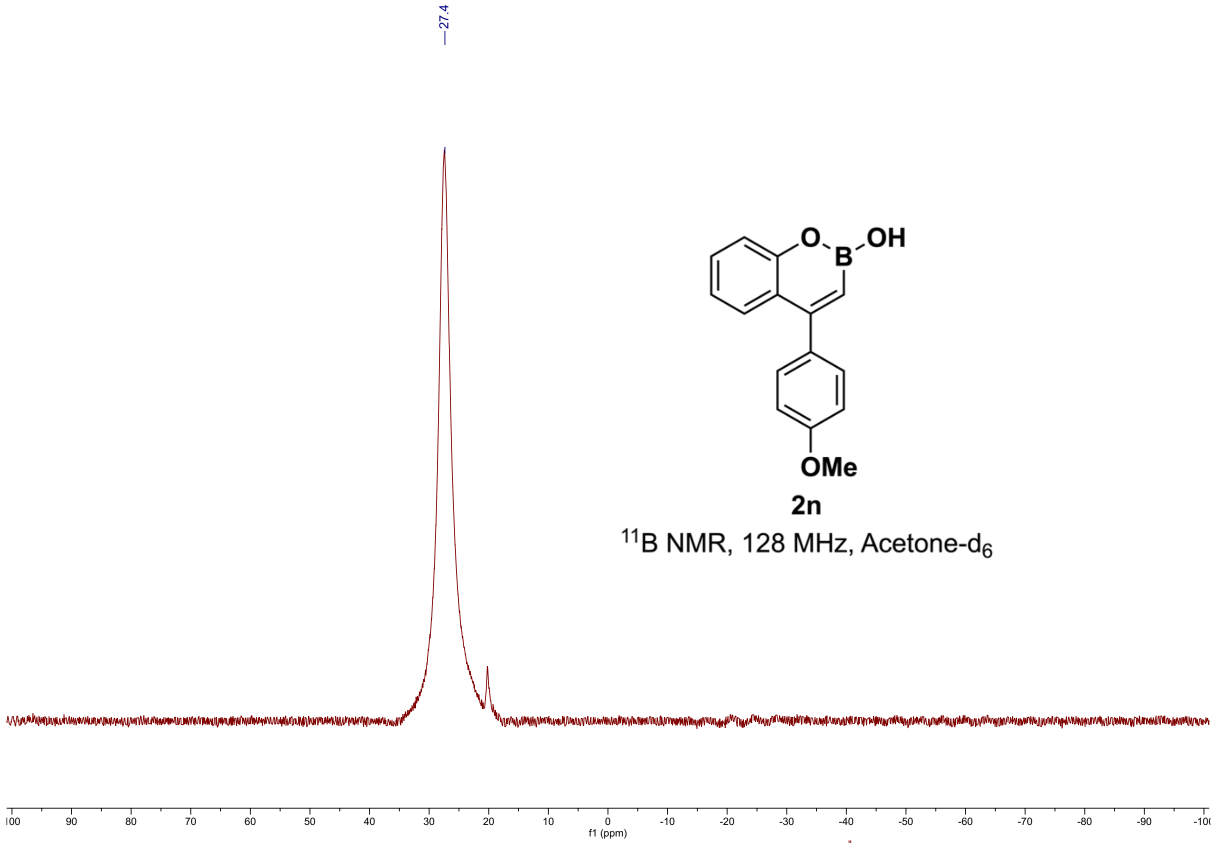
27.4
OH
O
B
OMe
2n
^{11}B NMR, 128 MHz, Acetone-d$_6$
f1 (ppm)

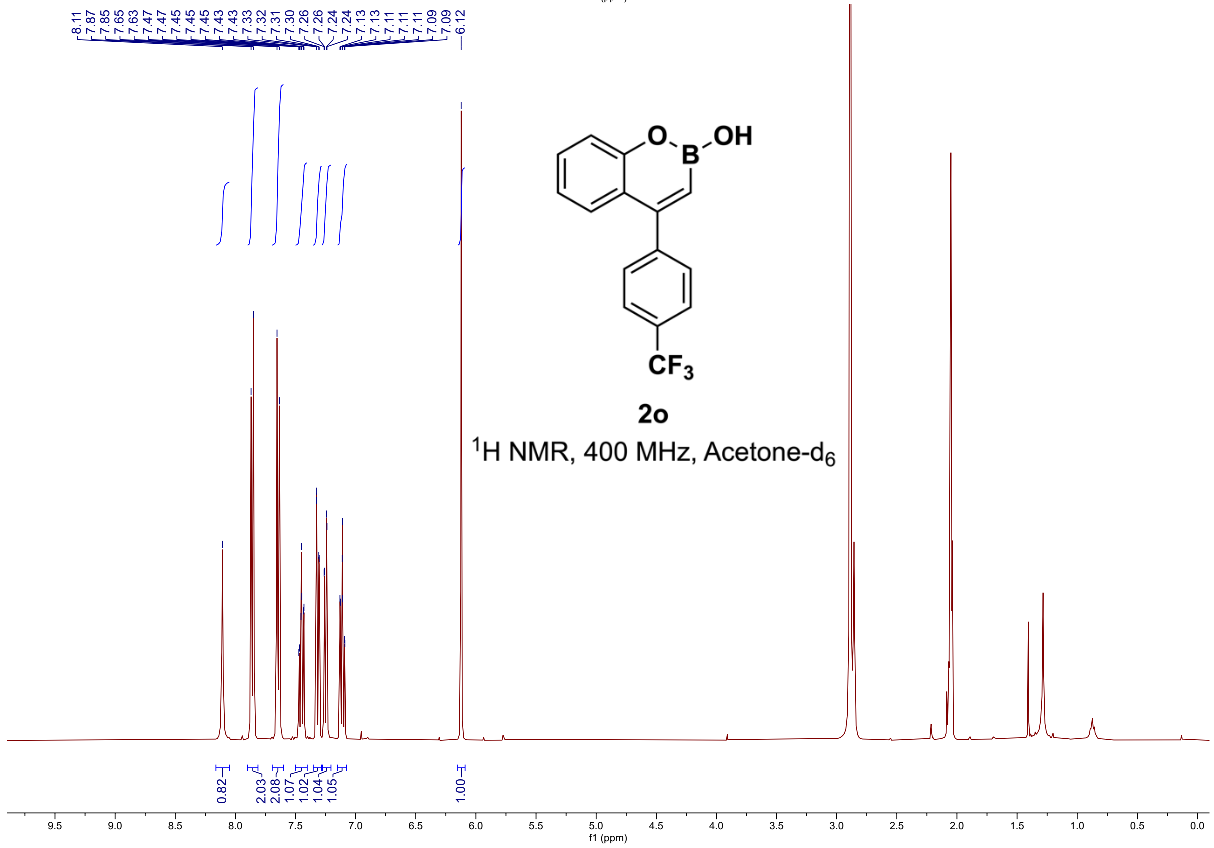
8.11 7.87 7.85 7.65 7.63 7.47 7.47 7.45 7.45 7.45 7.43 7.43 7.33 7.32 7.31 7.30 7.26 7.26 7.24 7.24 7.13 7.13 7.11 7.11 7.11 7.09 6.12
OH
O
B
CF$_3$
2o
^{1}H NMR, 400 MHz, Acetone-d$_6$
0.82 2.03 2.08 1.07 1.02 1.04 1.05 1.00
f1 (ppm)

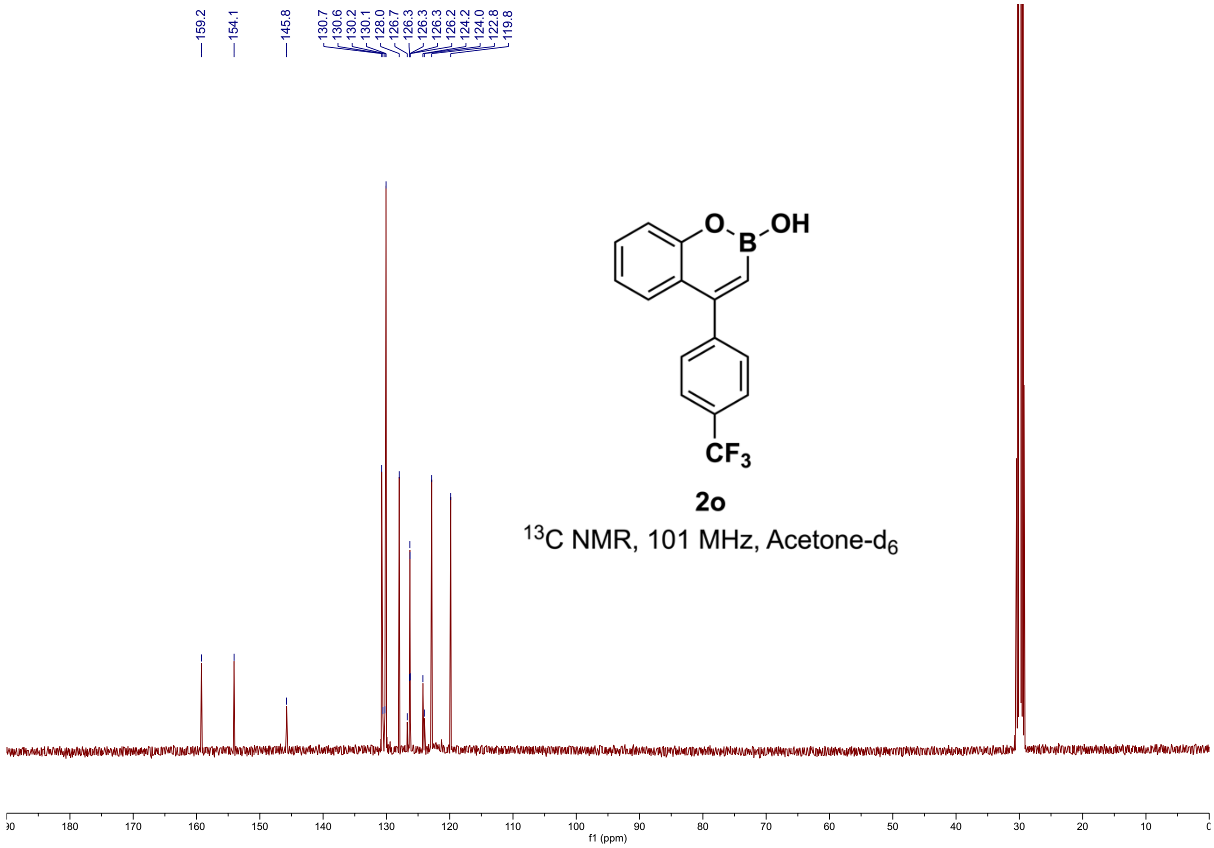
159.2
154.1
145.8
130.7
130.6
130.2
130.1
128.0
126.7
126.3
126.3
126.3
126.2
124.2
124.0
122.8
119.8
OH
O
B
CF3
2o
13C NMR, 101 MHz, Acetone-d6
f1 (ppm)

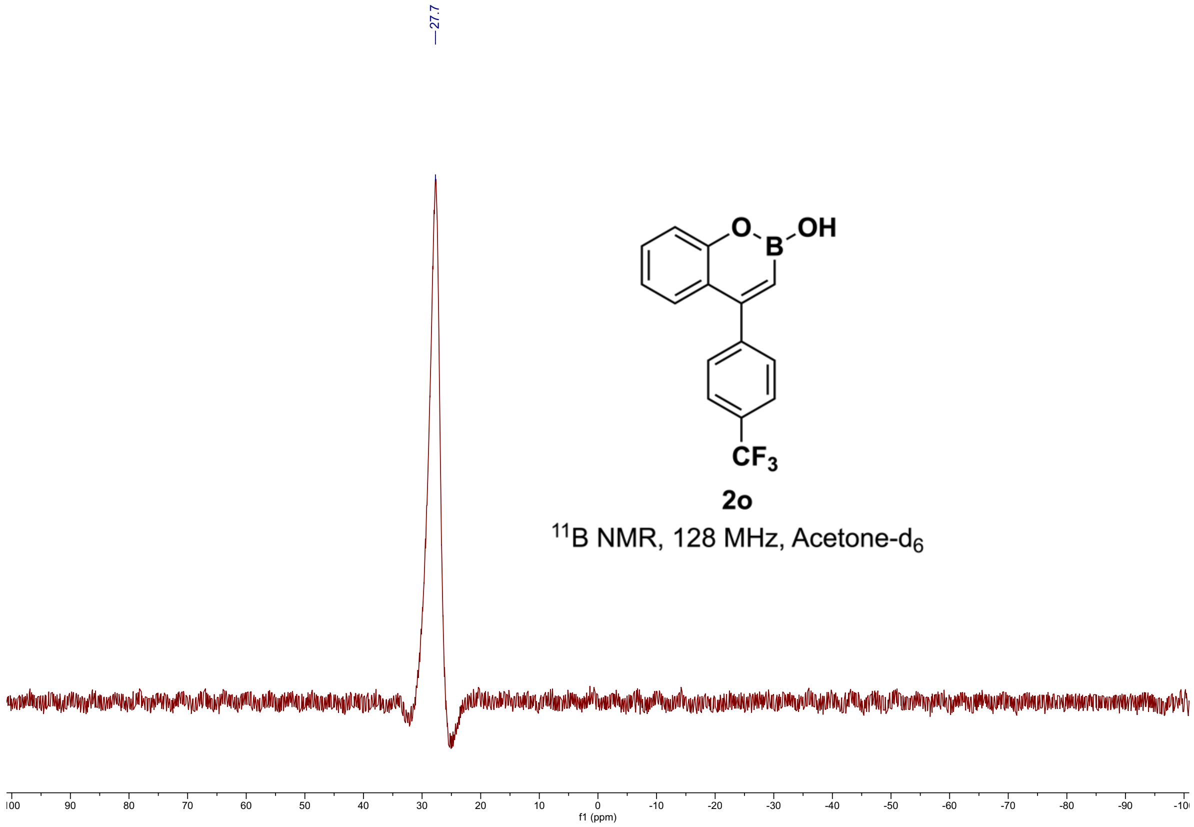
27.7
OH
O
B
CF3
2o
11B NMR, 128 MHz, Acetone-d6
f1 (ppm)

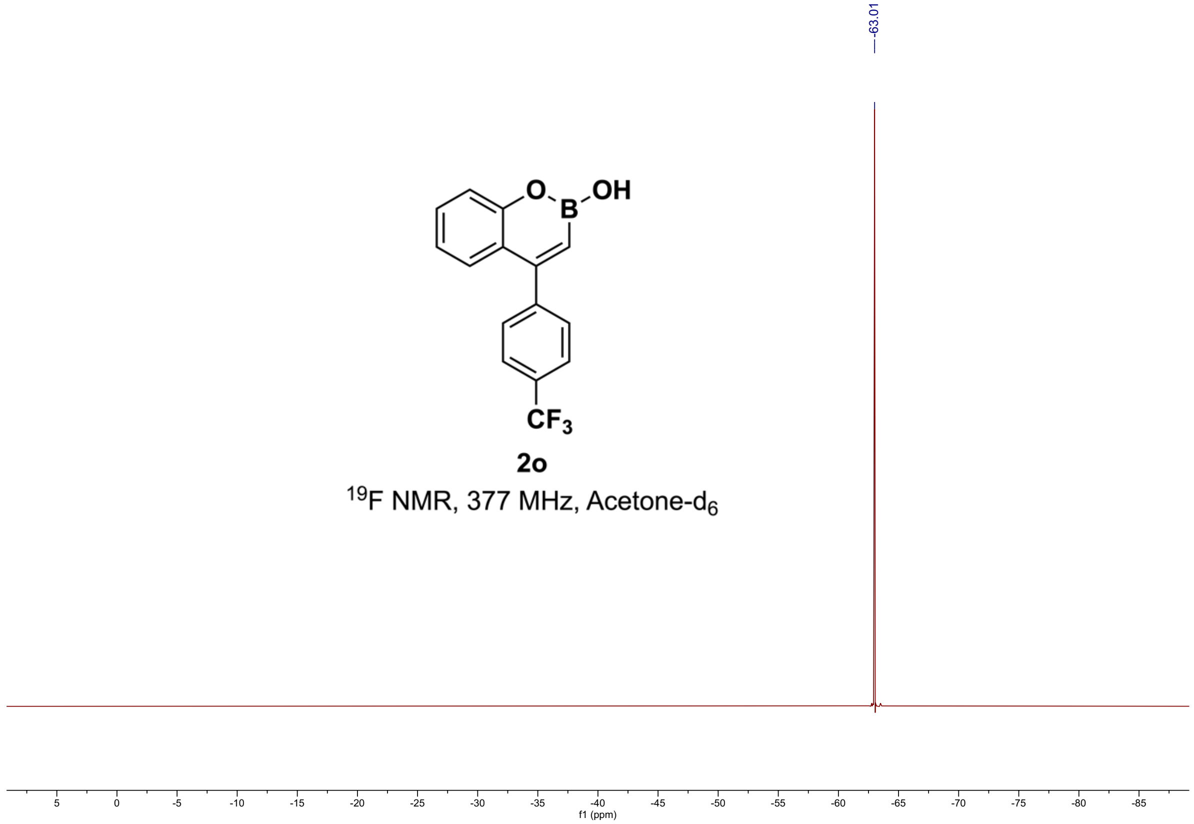
-63.01
OH
B
O
CF3
2o
19F NMR, 377 MHz, Acetone-d6
f1 (ppm)

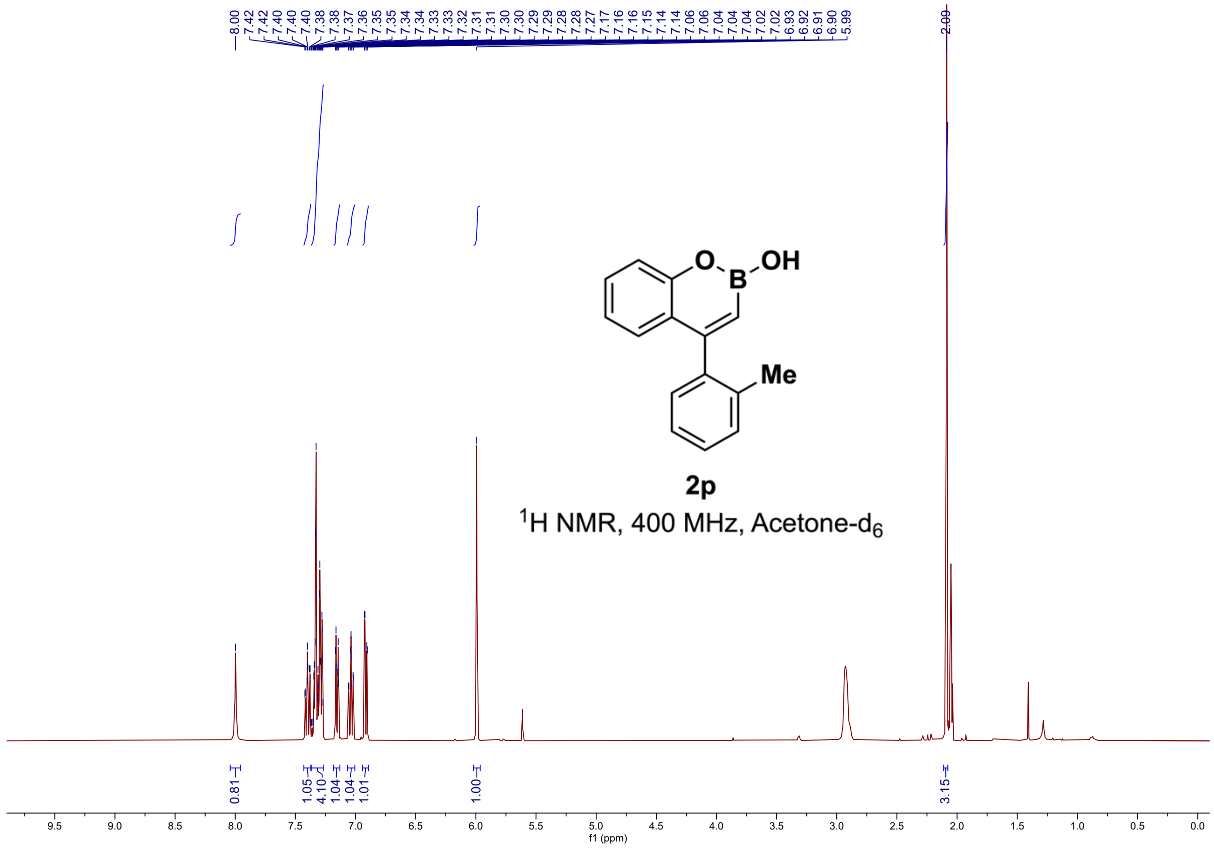
8.00
7.42
7.42
7.40
7.40
7.40
7.38
7.38
7.37
7.36
7.35
7.35
7.34
7.34
7.33
7.33
7.32
7.31
7.31
7.30
7.29
7.29
7.28
7.28
7.27
7.17
7.16
7.15
7.14
7.14
7.06
7.04
7.04
7.04
7.02
7.02
6.93
6.92
6.91
6.90
5.99
2.09
OH
B
O
Me
2p
1H NMR, 400 MHz, Acetone-d6
0.81
1.05
4.10
1.04
1.04
1.01
1.00
3.15
f1 (ppm)

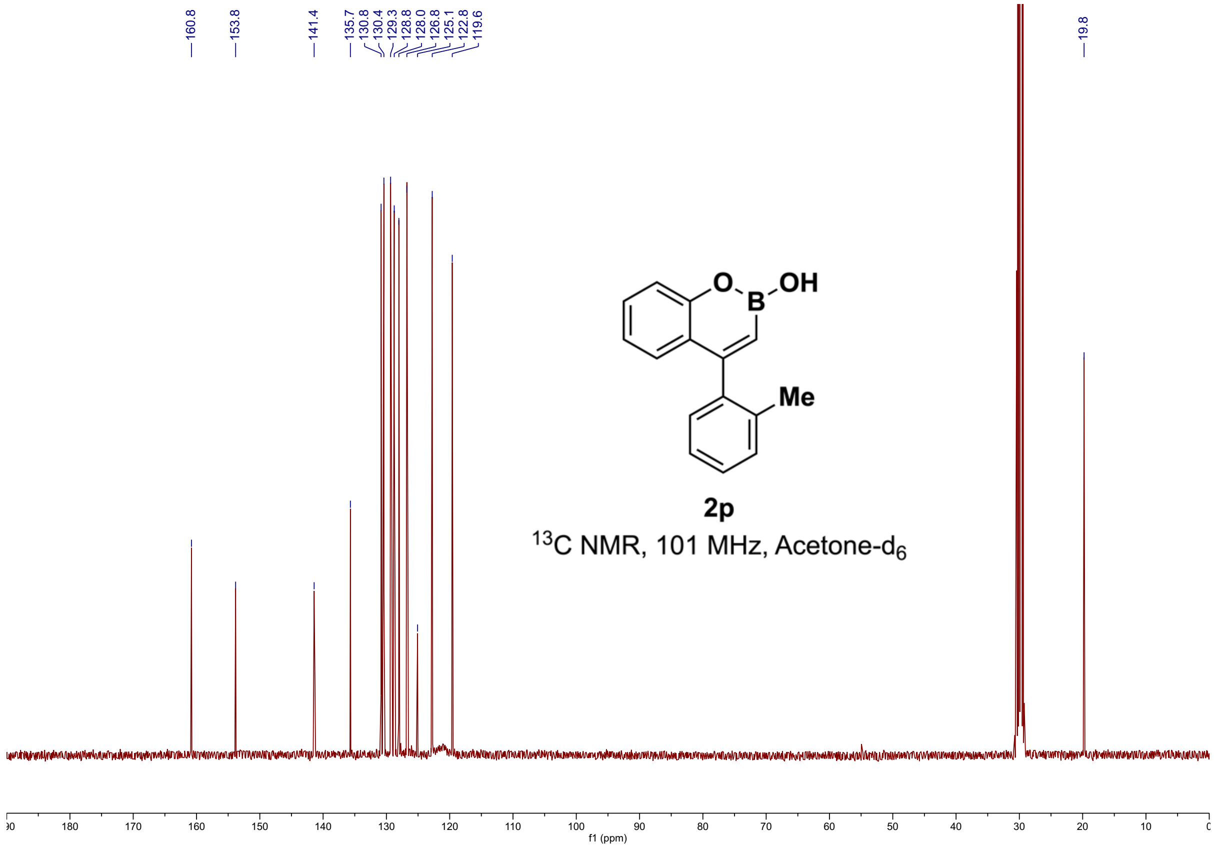
160.8
153.8
141.4
135.7
130.8
130.4
129.3
128.8
128.0
126.8
125.1
122.8
119.6
19.8
2p
^{13}C NMR, 101 MHz, Acetone-d_6
f1 (ppm)

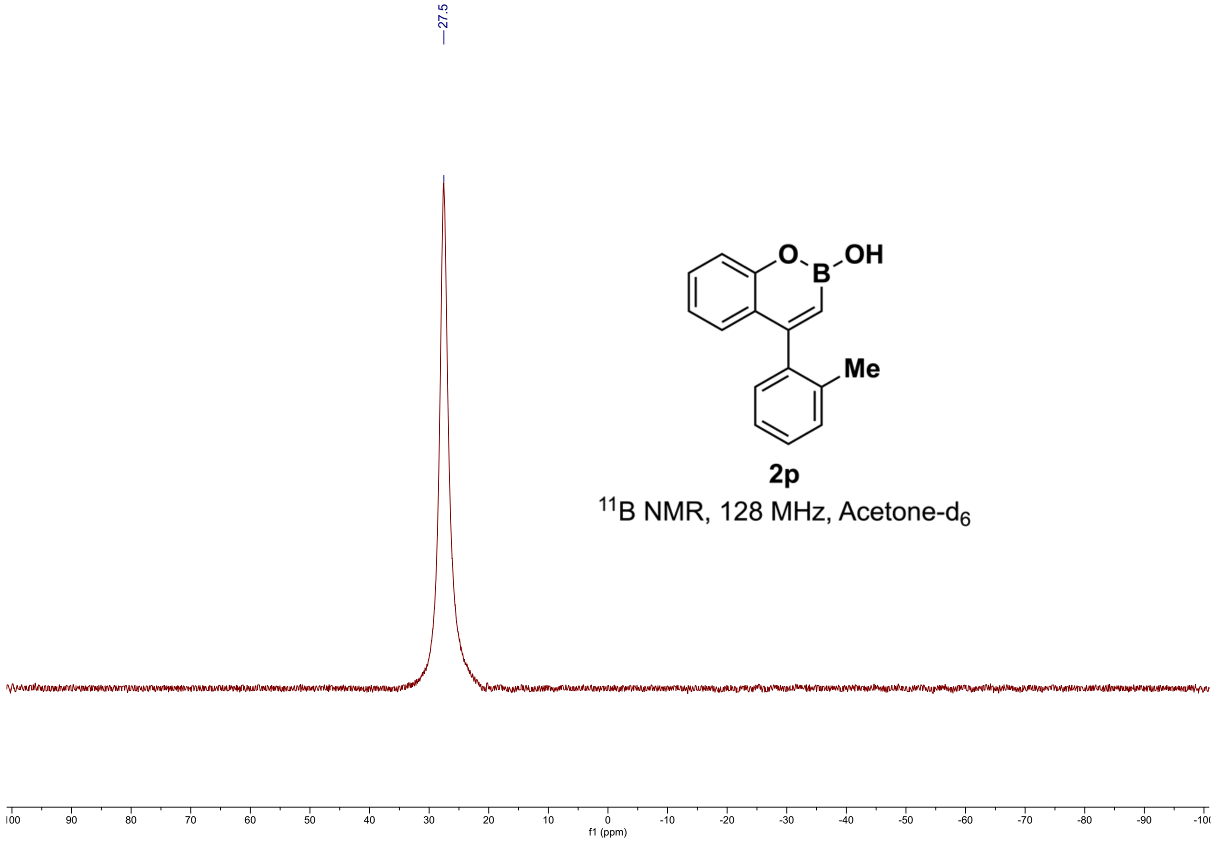
27.5
2p
^{11}B NMR, 128 MHz, Acetone-d_6
f1 (ppm)

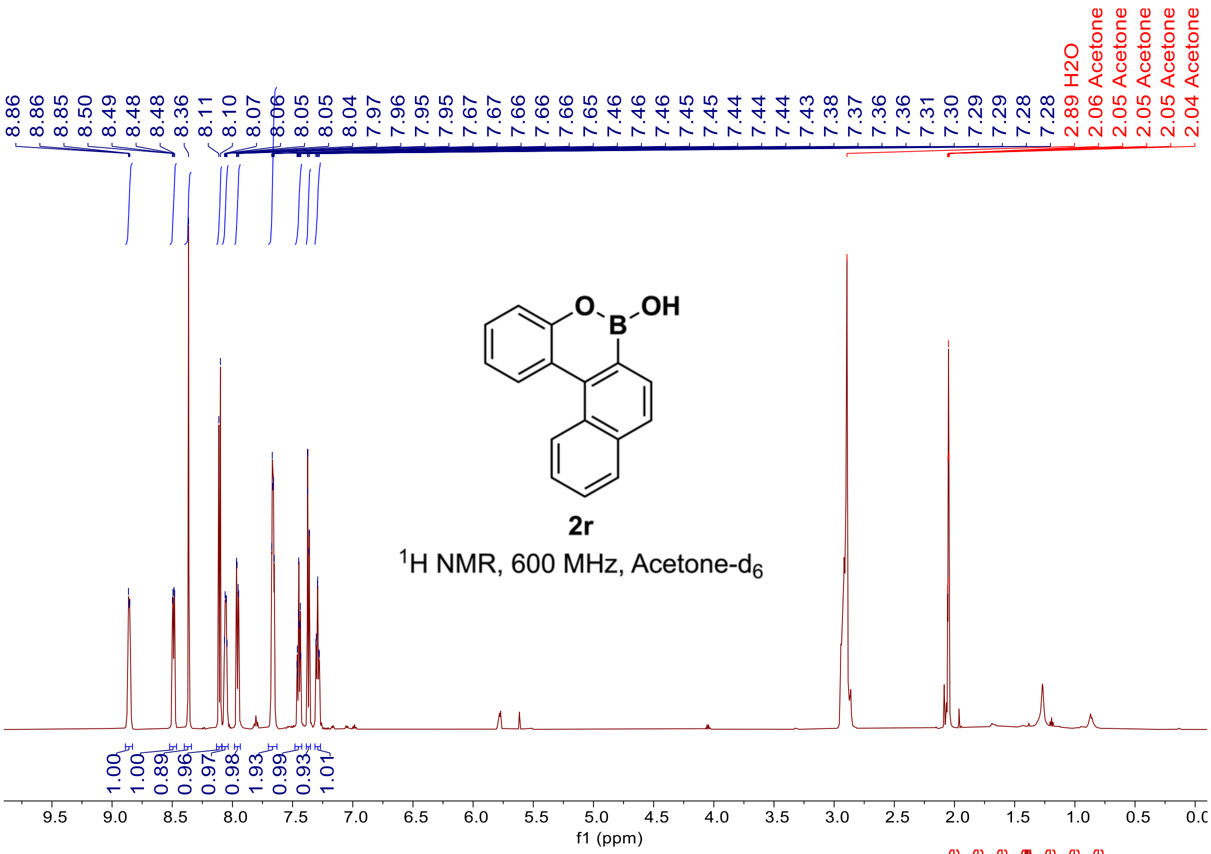
8.86 8.86 8.85 8.50 8.49 8.48 8.48 8.36 8.11 8.10 8.07 8.06 8.05 8.05 8.04 7.97 7.96 7.95 7.95 7.67 7.67 7.66 7.66 7.65 7.46 7.46 7.46 7.45 7.45 7.44 7.44 7.44 7.43 7.38 7.37 7.36 7.36 7.31 7.30 7.29 7.29 7.28 7.28
2.89 H2O
2.06 Acetone
2.05 Acetone
2.05 Acetone
2.05 Acetone
2.04 Acetone
O
B
OH
2r
1H NMR, 600 MHz, Acetone-d6
1.00 1.00 0.89 0.96 0.97 0.98 1.93 0.99 0.93 1.01
f1 (ppm)

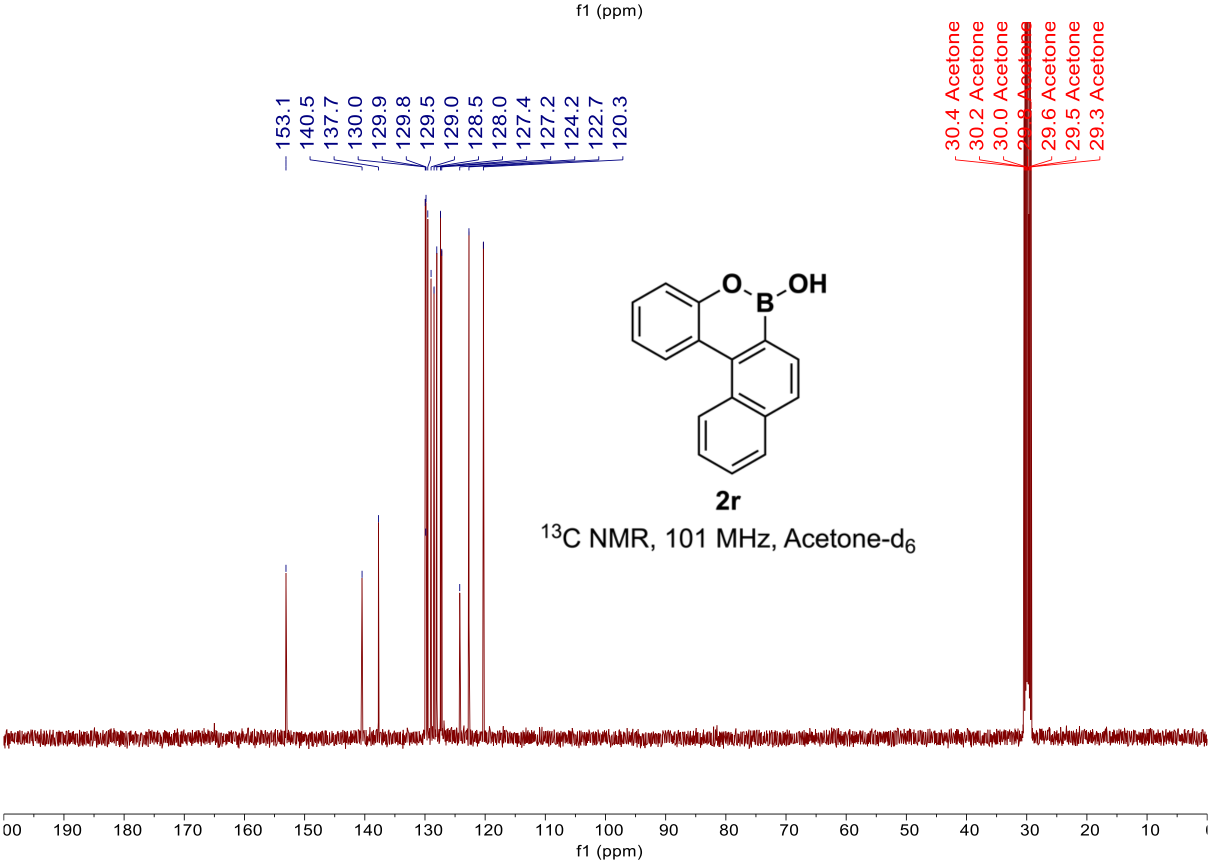
153.1 140.5 137.7 130.0 129.9 129.8 129.5 129.0 128.5 128.0 127.4 127.2 124.2 122.7 120.3
30.4 Acetone
30.2 Acetone
30.0 Acetone
29.8 Acetone
29.6 Acetone
29.5 Acetone
29.3 Acetone
O
B
OH
2r
13C NMR, 101 MHz, Acetone-d6
f1 (ppm)

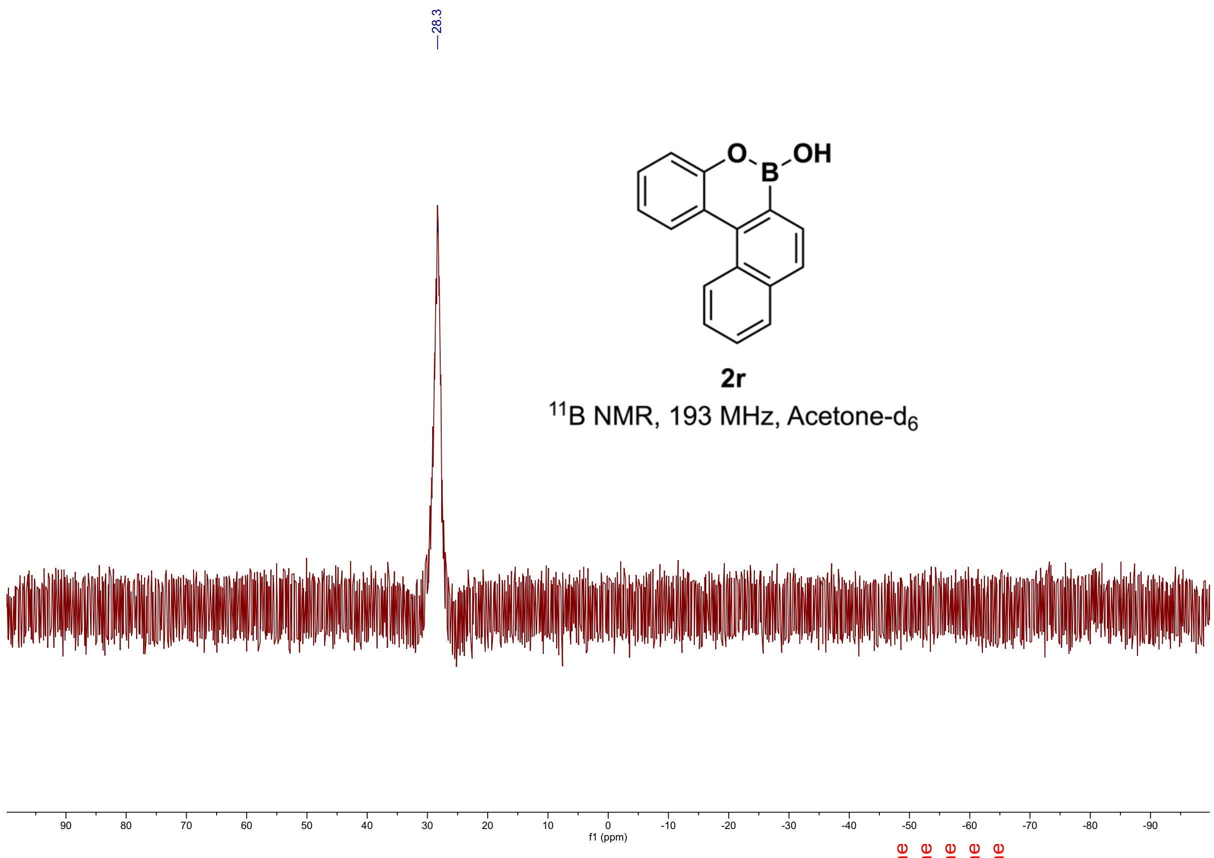

28.3
OH
O
B
2r
^{11}B NMR, 193 MHz, Acetone-d$_6$
f1 (ppm)

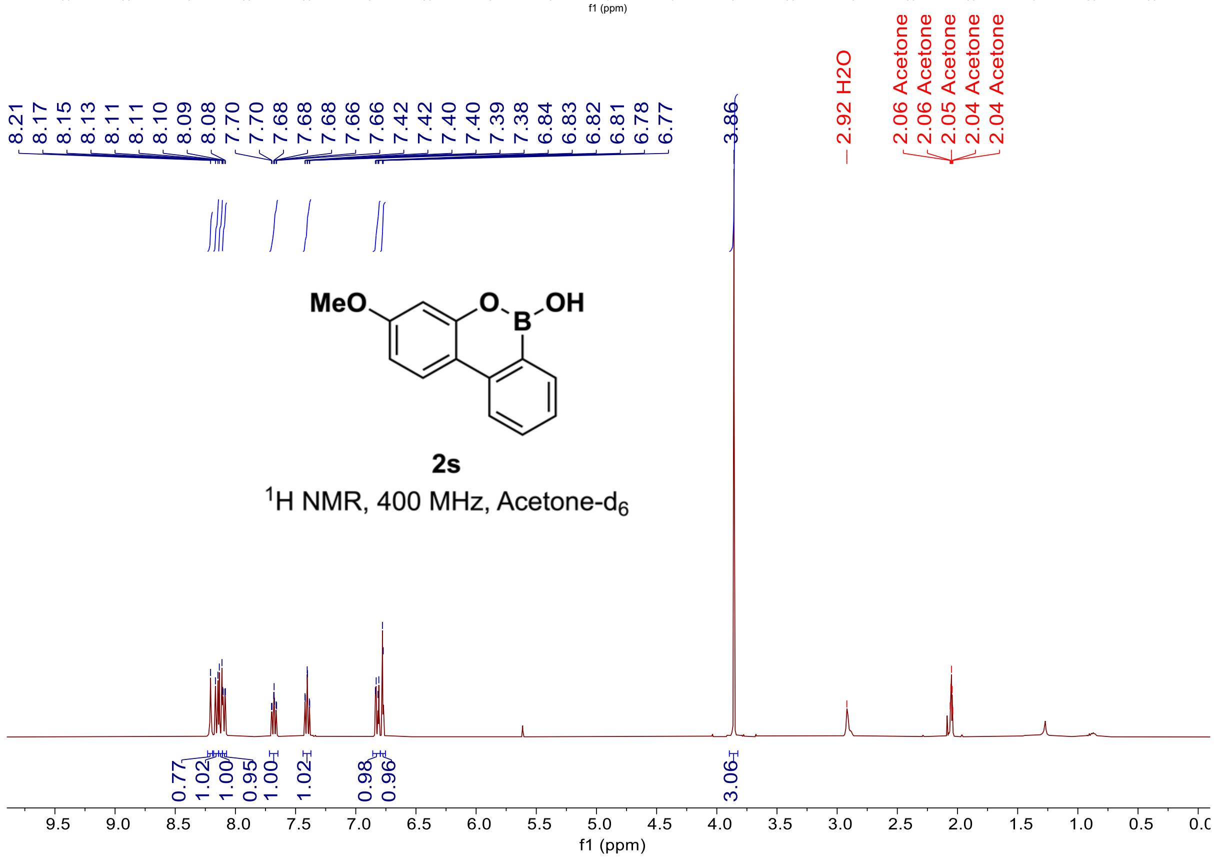

8.21 8.17 8.15 8.13 8.11 8.11 8.10 8.09 8.08 7.70 7.70 7.68 7.68 7.68 7.66 7.66 7.42 7.42 7.40 7.40 7.39 7.38 6.84 6.83 6.82 6.81 6.78 6.77
3.86
2.92 H2O
2.06 Acetone
2.06 Acetone
2.05 Acetone
2.04 Acetone
2.04 Acetone
MeO
O
B
OH
2s
^{1}H NMR, 400 MHz, Acetone-d$_6$
0.77 1.02 1.00 0.95 1.00 1.02 0.98 0.96 3.06
f1 (ppm)

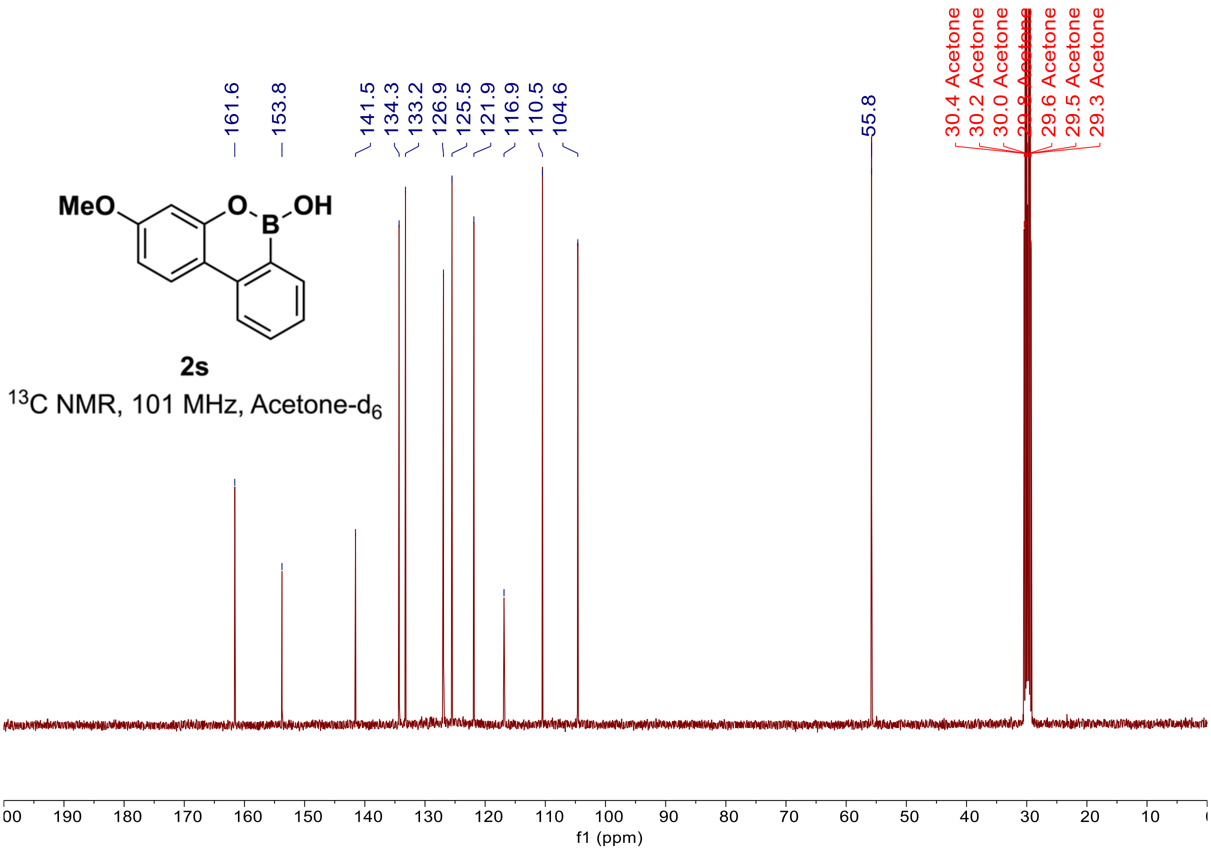

161.6
153.8
141.5
134.3
133.2
126.9
125.5
121.9
116.9
110.5
104.6
55.8
30.4 Acetone
30.2 Acetone
30.0 Acetone
29.8 Acetone
29.6 Acetone
29.5 Acetone
29.3 Acetone
MeO
O
B
OH
2s
13C NMR, 101 MHz, Acetone-d6
f1 (ppm)

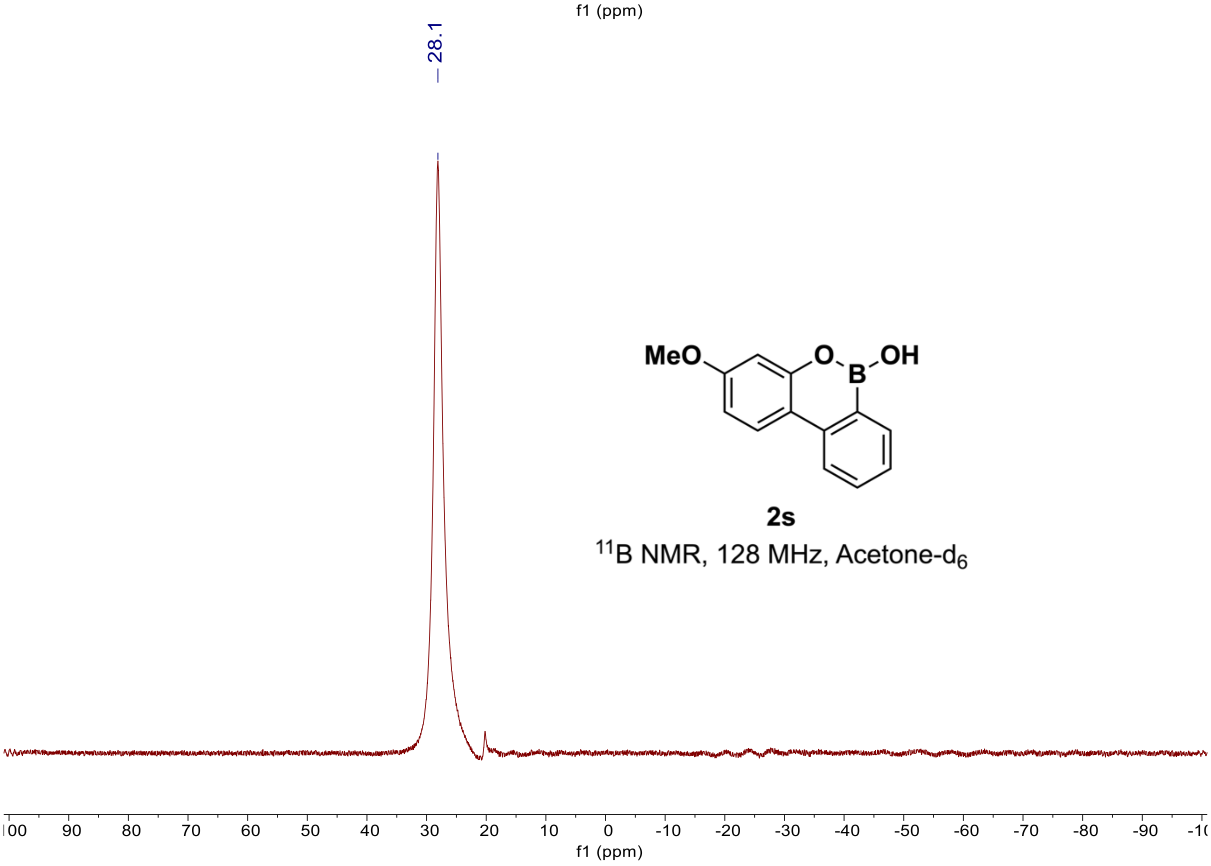

28.1
MeO
O
B
OH
2s
11B NMR, 128 MHz, Acetone-d6
f1 (ppm)

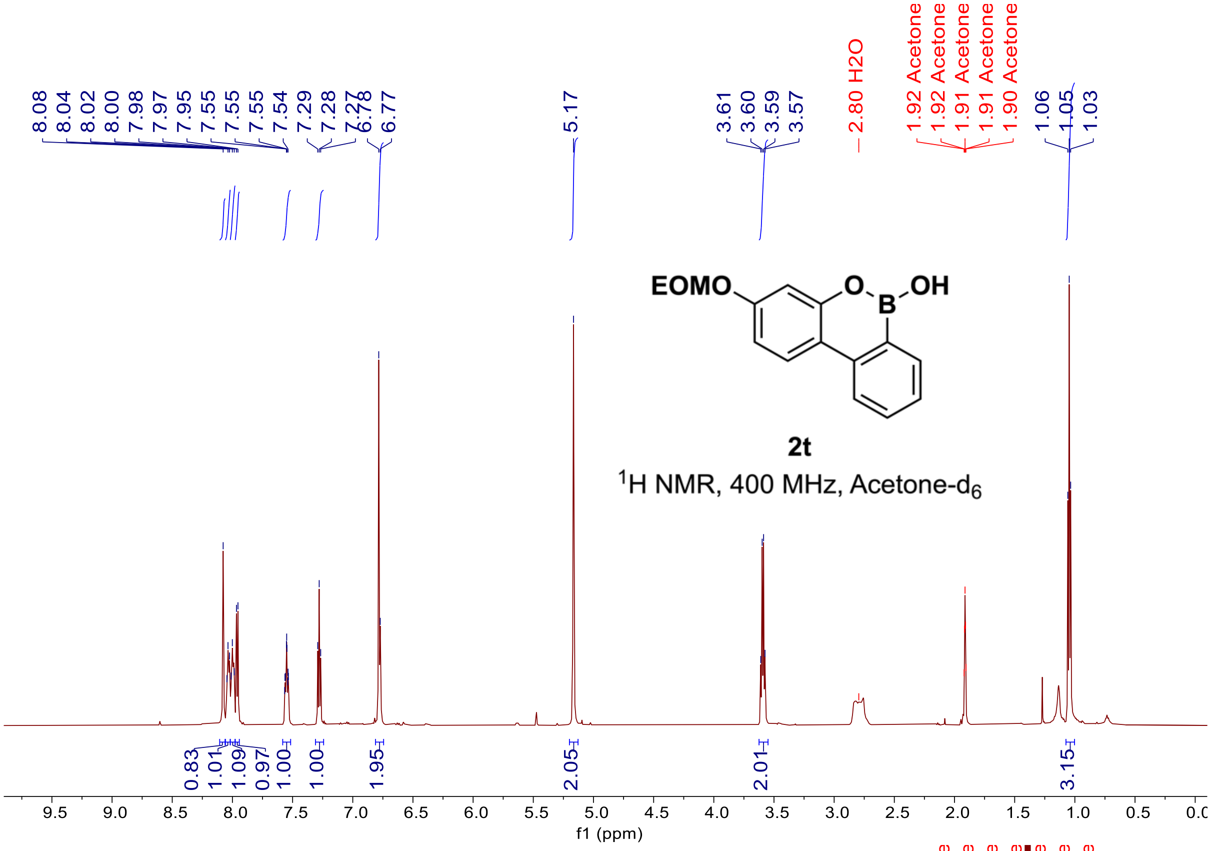
EOMO
O
B
OH
2t
1H NMR, 400 MHz, Acetone-d6
8.08 8.04 8.02 8.00 7.98 7.97 7.95 7.55 7.55 7.55 7.54 7.29 7.28 7.27 6.78 6.77
5.17
3.61 3.60 3.59 3.57
2.80 H2O
1.92 Acetone 1.92 Acetone 1.91 Acetone 1.91 Acetone 1.90 Acetone
1.06 1.05 1.03
0.83 1.01 1.09 0.97 1.00 1.00 1.95 2.05 2.01 3.15
9.5 9.0 8.5 8.0 7.5 7.0 6.5 6.0 5.5 5.0 4.5 4.0 3.5 3.0 2.5 2.0 1.5 1.0 0.5 0.0
f1 (ppm)

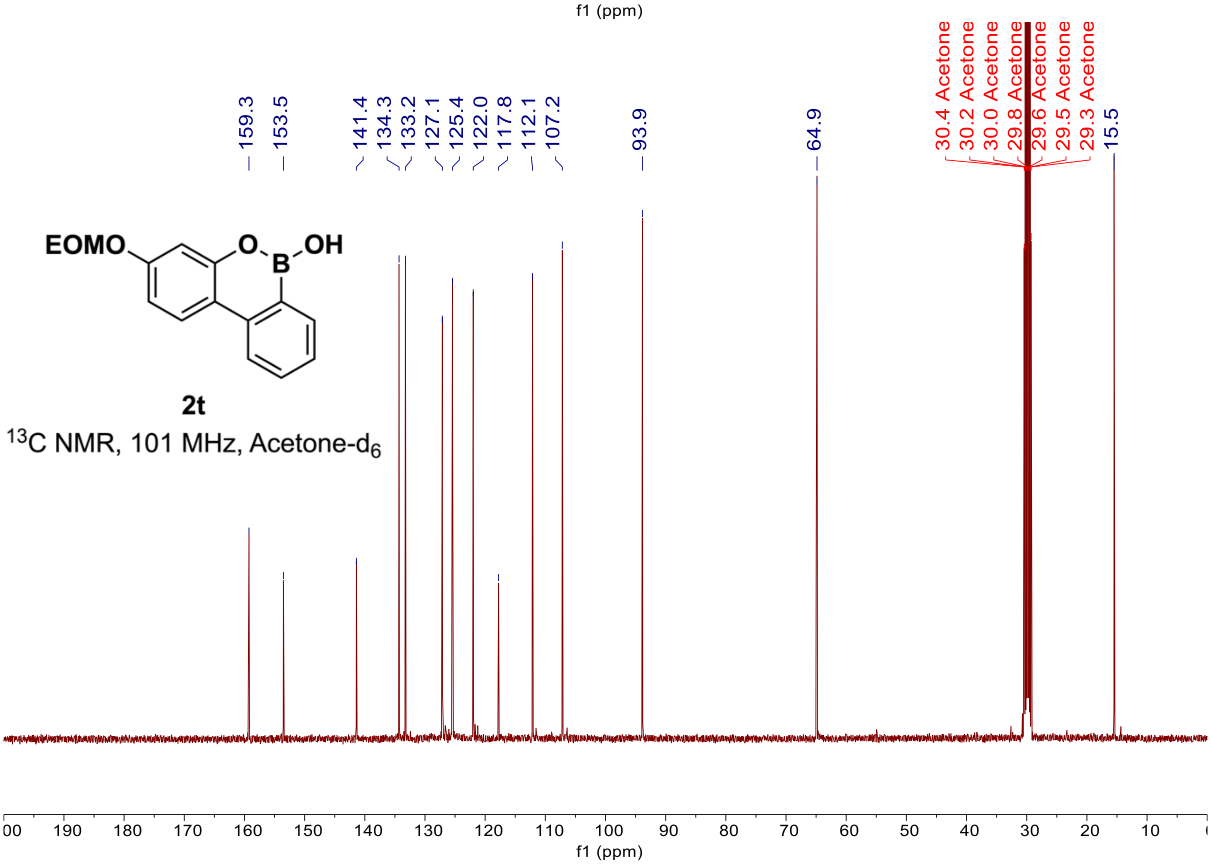
159.3
153.5
141.4 134.3 133.2 127.1 125.4 122.0 117.8 112.1 107.2
93.9
64.9
30.4 Acetone 30.2 Acetone 30.0 Acetone 29.8 Acetone 29.6 Acetone 29.5 Acetone 29.3 Acetone
15.5
EOMO
O
B
OH
2t
13C NMR, 101 MHz, Acetone-d6
190 180 170 160 150 140 130 120 110 100 90 80 70 60 50 40 30 20 10
f1 (ppm)

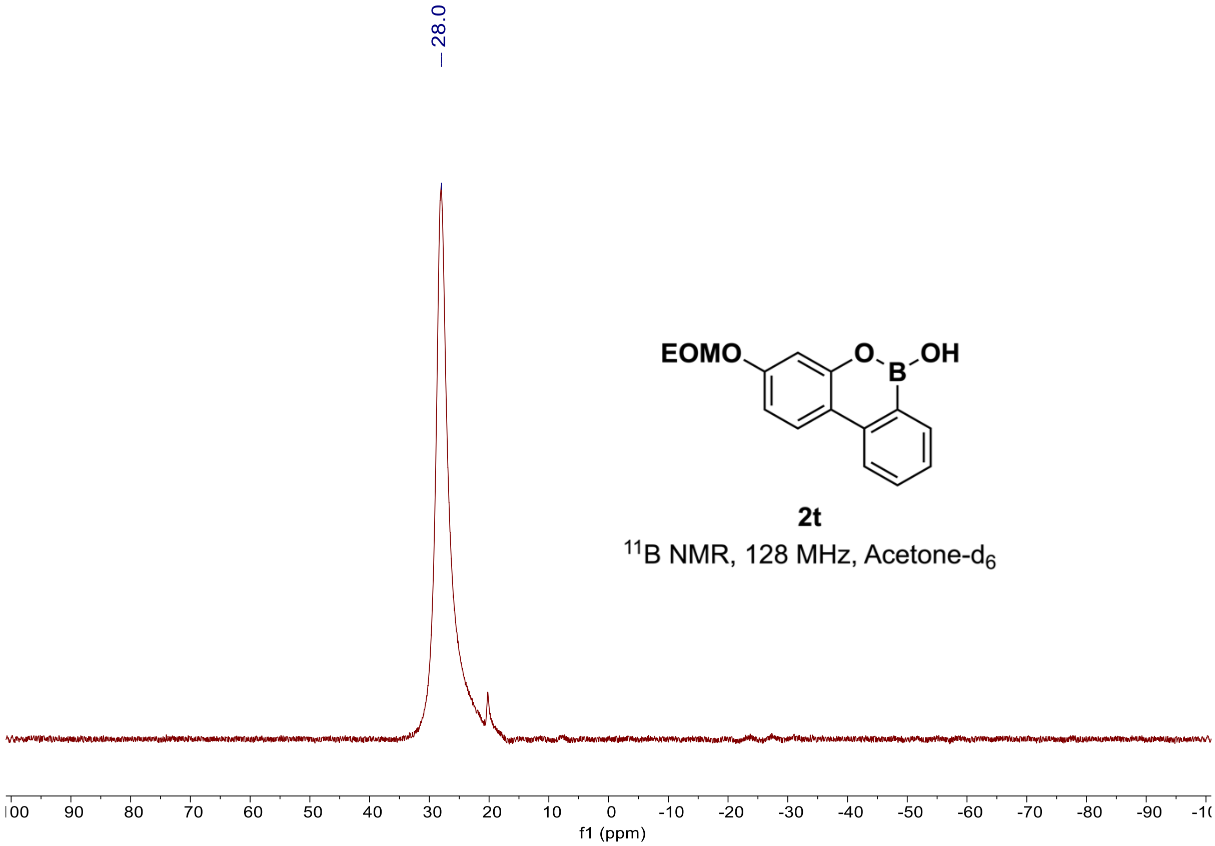
28.0
EOMO
O
B
OH
2t
11B NMR, 128 MHz, Acetone-d6
100 90 80 70 60 50 40 30 20 10 0 -10 -20 -30 -40 -50 -60 -70 -80 -90 -100
f1 (ppm)

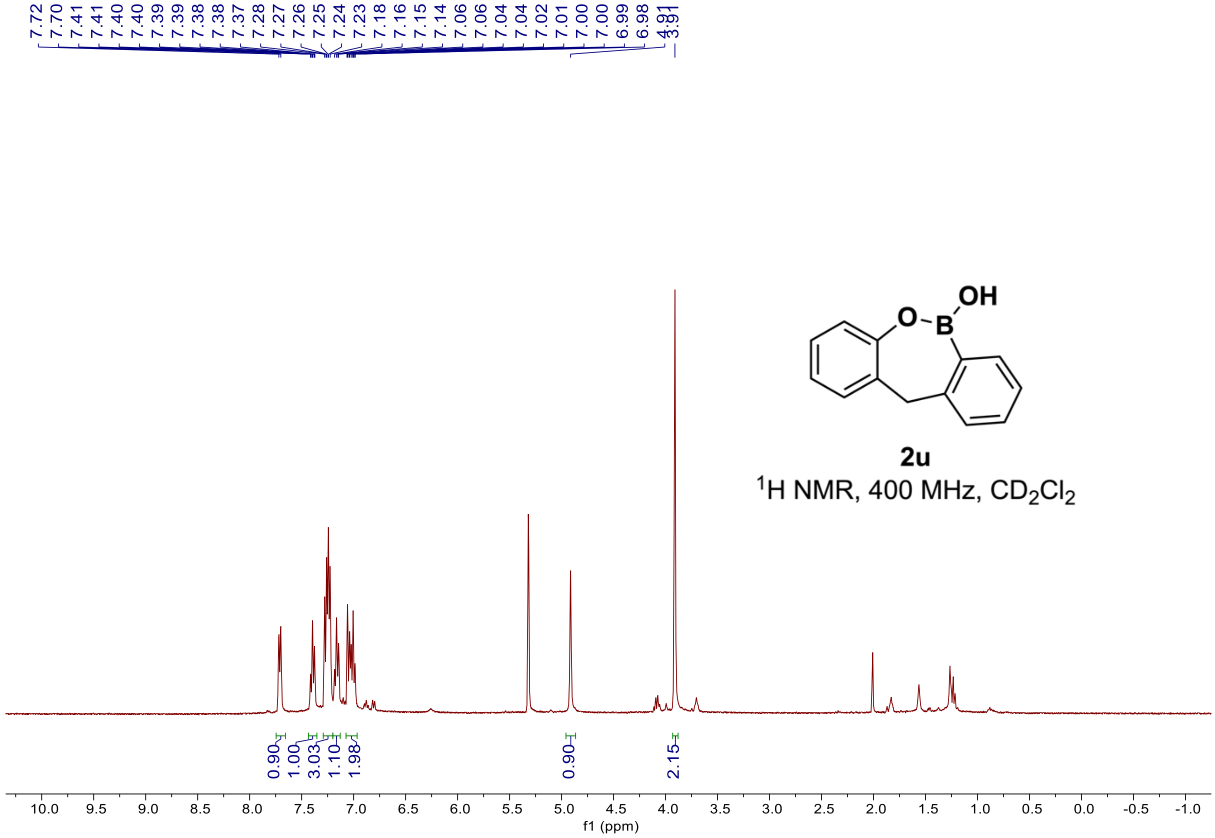
7.72 7.70 7.41 7.41 7.40 7.40 7.39 7.39 7.38 7.38 7.37 7.28 7.27 7.26 7.25 7.24 7.23 7.18 7.16 7.15 7.14 7.06 7.06 7.04 7.04 7.02 7.01 7.00 7.00 6.99 6.98 4.91 3.91
OH
O
B
2u
1H NMR, 400 MHz, CD2Cl2
0.90 1.00 3.03 1.10 1.98 0.90 2.15
10.0 9.5 9.0 8.5 8.0 7.5 7.0 6.5 6.0 5.5 5.0 4.5 4.0 3.5 3.0 2.5 2.0 1.5 1.0 0.5 0.0 -0.5 -1.0
f1 (ppm)

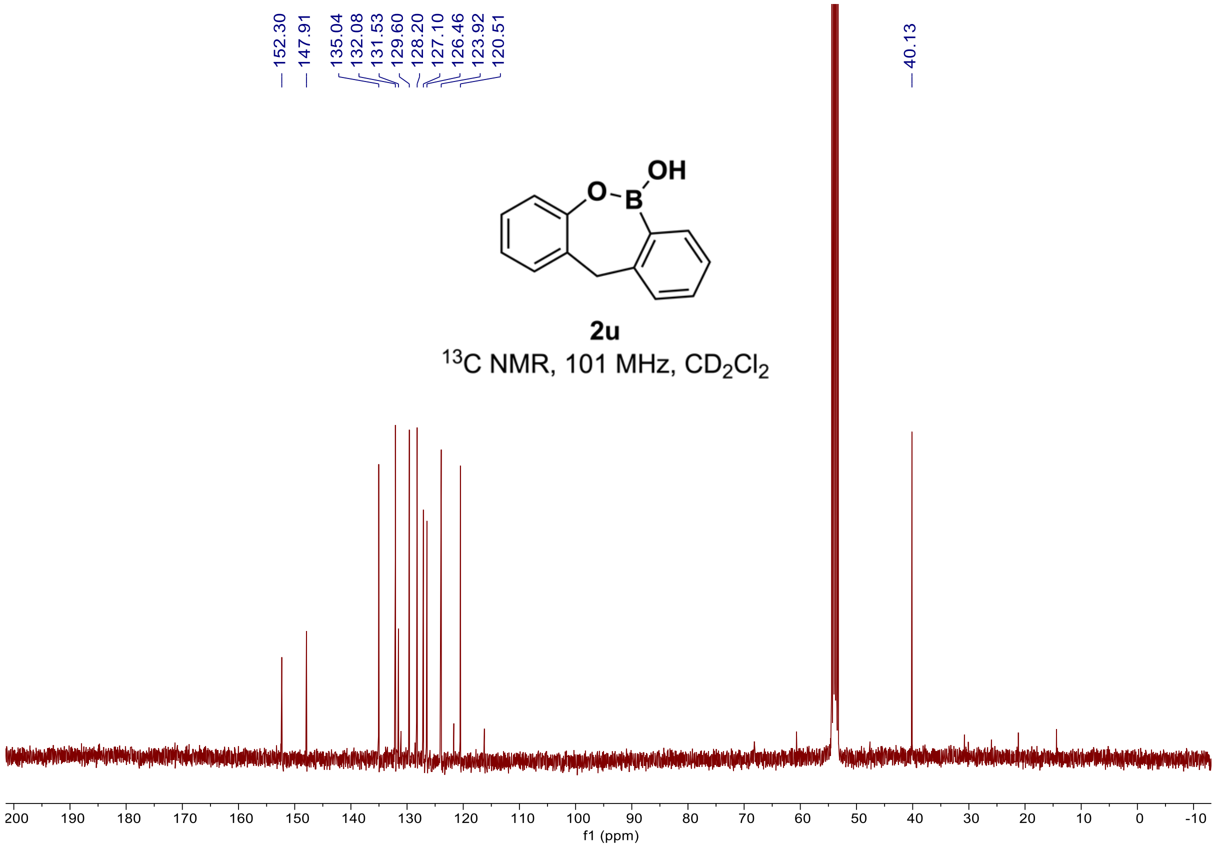
152.30
147.91
135.04
132.08
131.53
129.60
128.20
127.10
126.46
123.92
120.51
40.13
OH
O
B
2u
^{13}C NMR, 101 MHz, CD_2Cl_2
200 190 180 170 160 150 140 130 120 110 100 90 80 70 60 50 40 30 20 10 0 -10
f1 (ppm)

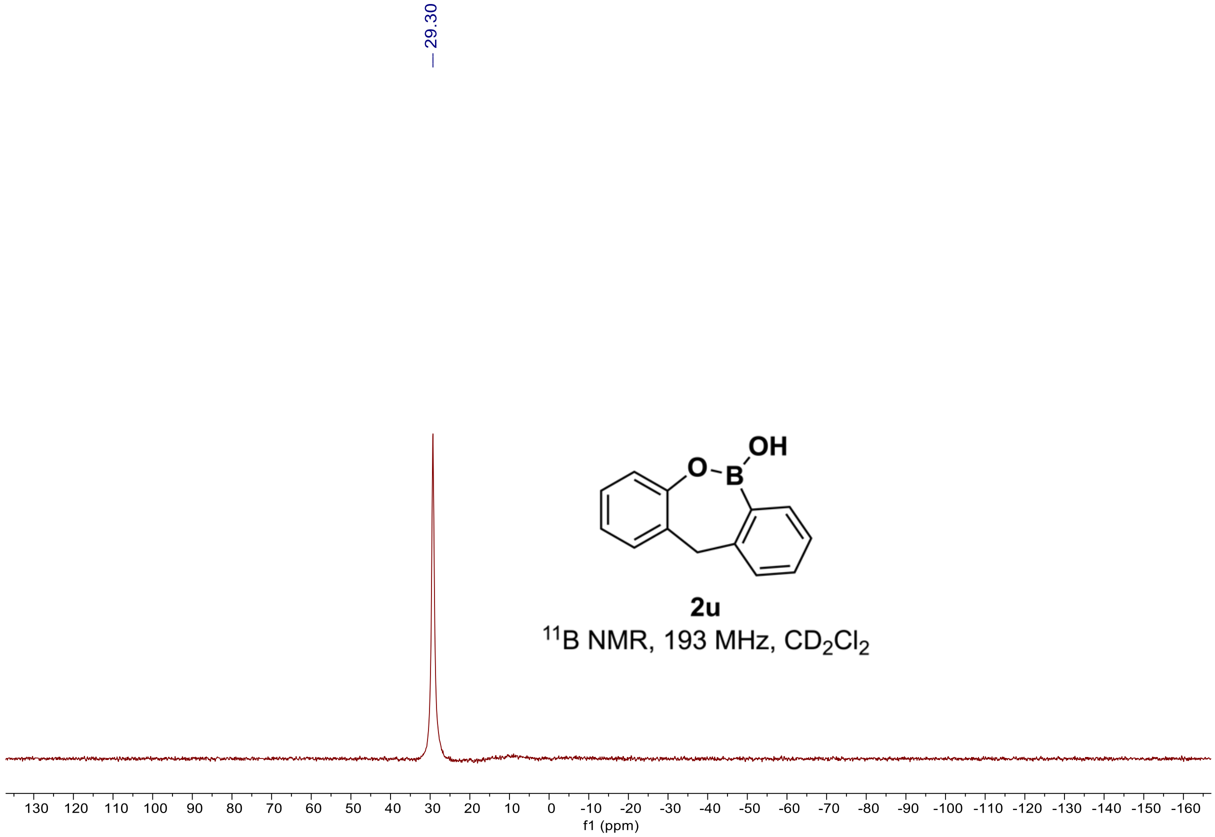
29.30
OH
O
B
2u
^{11}B NMR, 193 MHz, CD_2Cl_2
130 120 110 100 90 80 70 60 50 40 30 20 10 0 -10 -20 -30 -40 -50 -60 -70 -80 -90 -100 -110 -120 -130 -140 -150 -160
f1 (ppm)

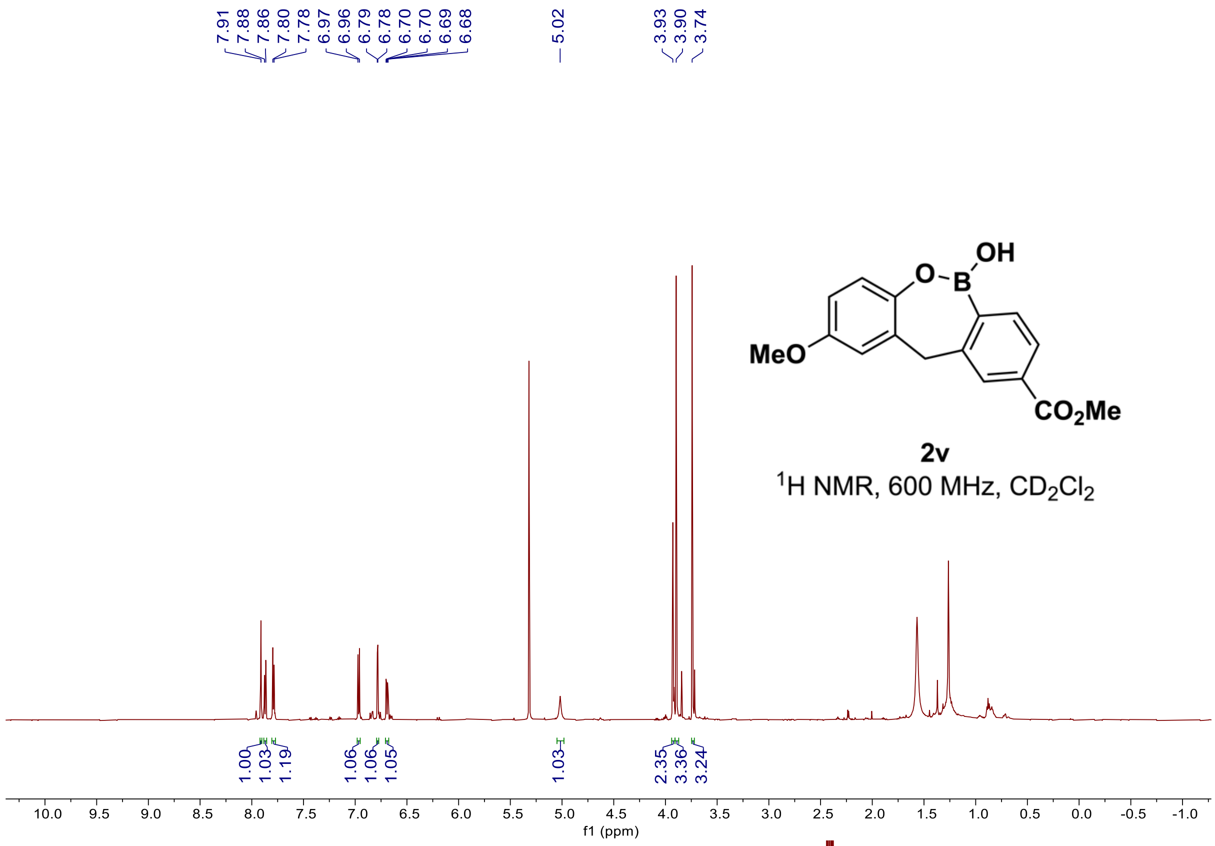

7.91
7.88
7.86
7.80
7.78
6.97
6.96
6.79
6.78
6.70
6.70
6.69
6.68
5.02
3.93
3.90
3.74
OH
O
B
MeO
CO2Me
2v
1H NMR, 600 MHz, CD2Cl2
1.00
1.03
1.19
1.06
1.06
1.05
1.03
2.35
3.36
3.24
10.0 9.5 9.0 8.5 8.0 7.5 7.0 6.5 6.0 5.5 5.0 4.5 4.0 3.5 3.0 2.5 2.0 1.5 1.0 0.5 0.0 -0.5 -1.0
f1 (ppm)

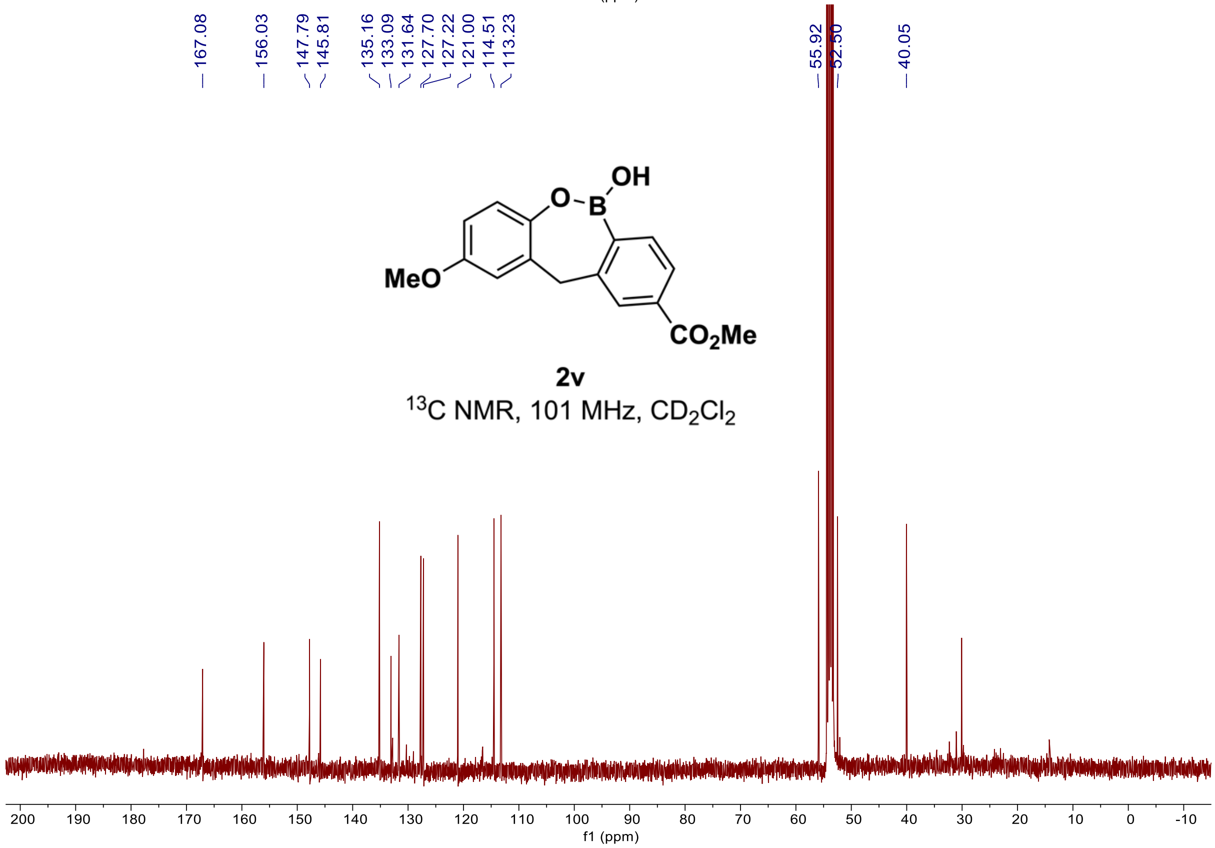

167.08
156.03
147.79
145.81
135.16
133.09
131.64
127.70
127.22
121.00
114.51
113.23
55.92
52.50
40.05
OH
O
B
MeO
CO2Me
2v
13C NMR, 101 MHz, CD2Cl2
200 190 180 170 160 150 140 130 120 110 100 90 80 70 60 50 40 30 20 10 0 -10
f1 (ppm)

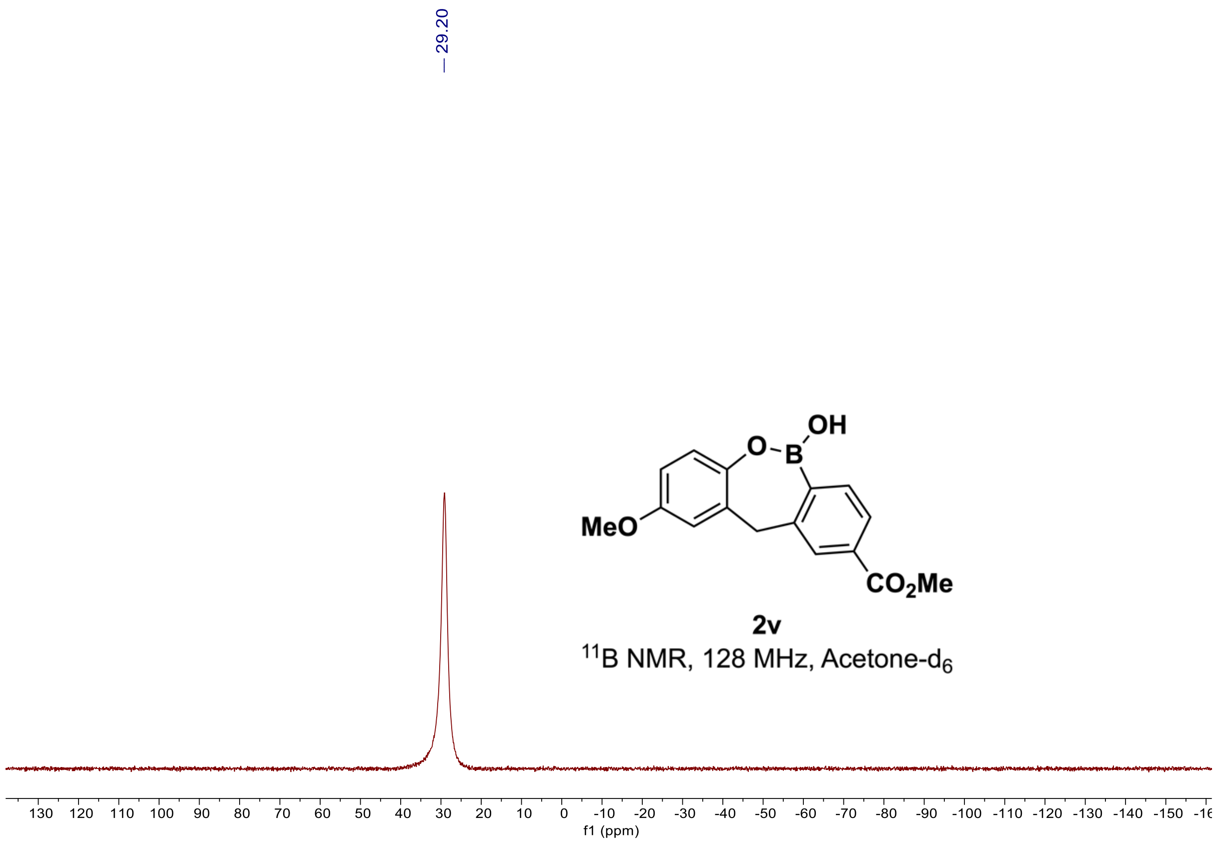

29.20
OH
O
B
MeO
CO_2Me
2v
^{11}B NMR, 128 MHz, Acetone-d_6
f1 (ppm)

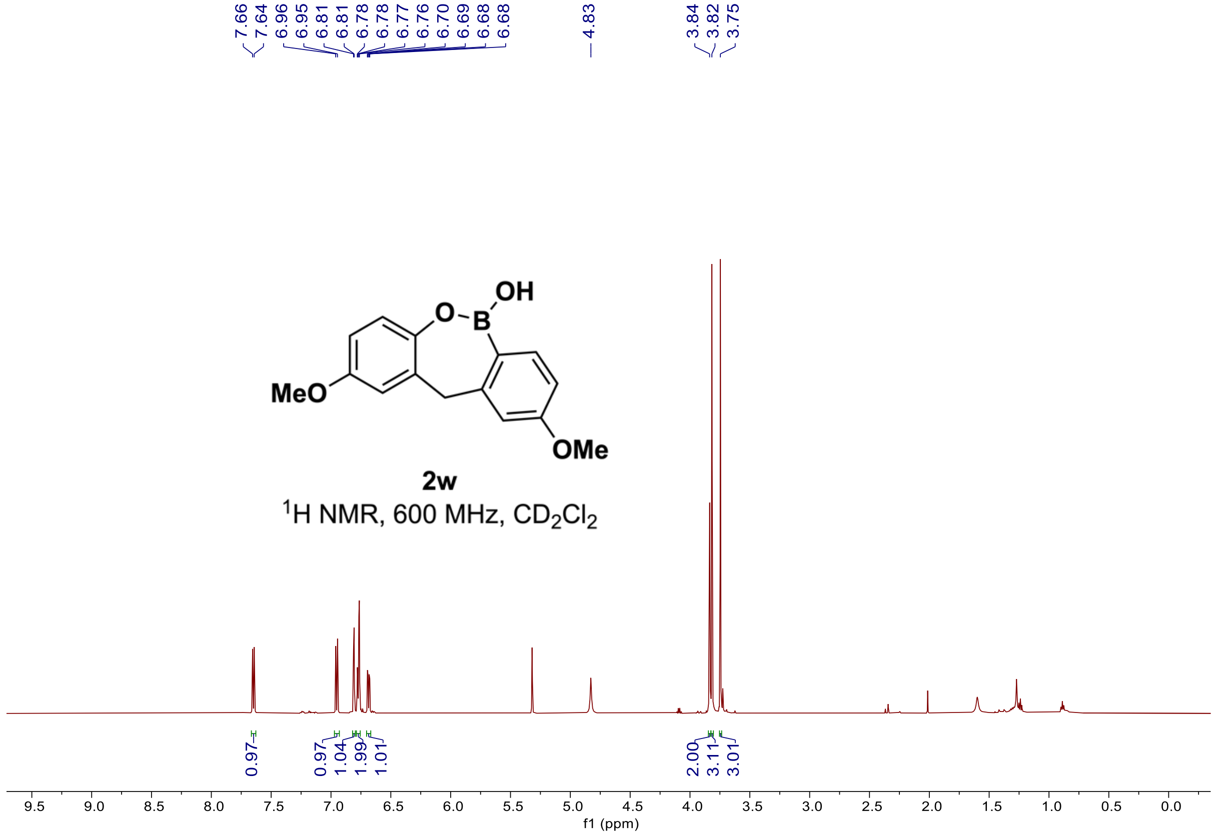

7.66
7.64
6.96
6.95
6.81
6.81
6.78
6.78
6.77
6.76
6.70
6.69
6.68
6.68
4.83
3.84
3.82
3.75
OH
O
B
MeO
OMe
2w
^{1}H NMR, 600 MHz, CD_2Cl_2
0.97
0.97
1.04
1.99
1.01
2.00
3.11
3.01
f1 (ppm)

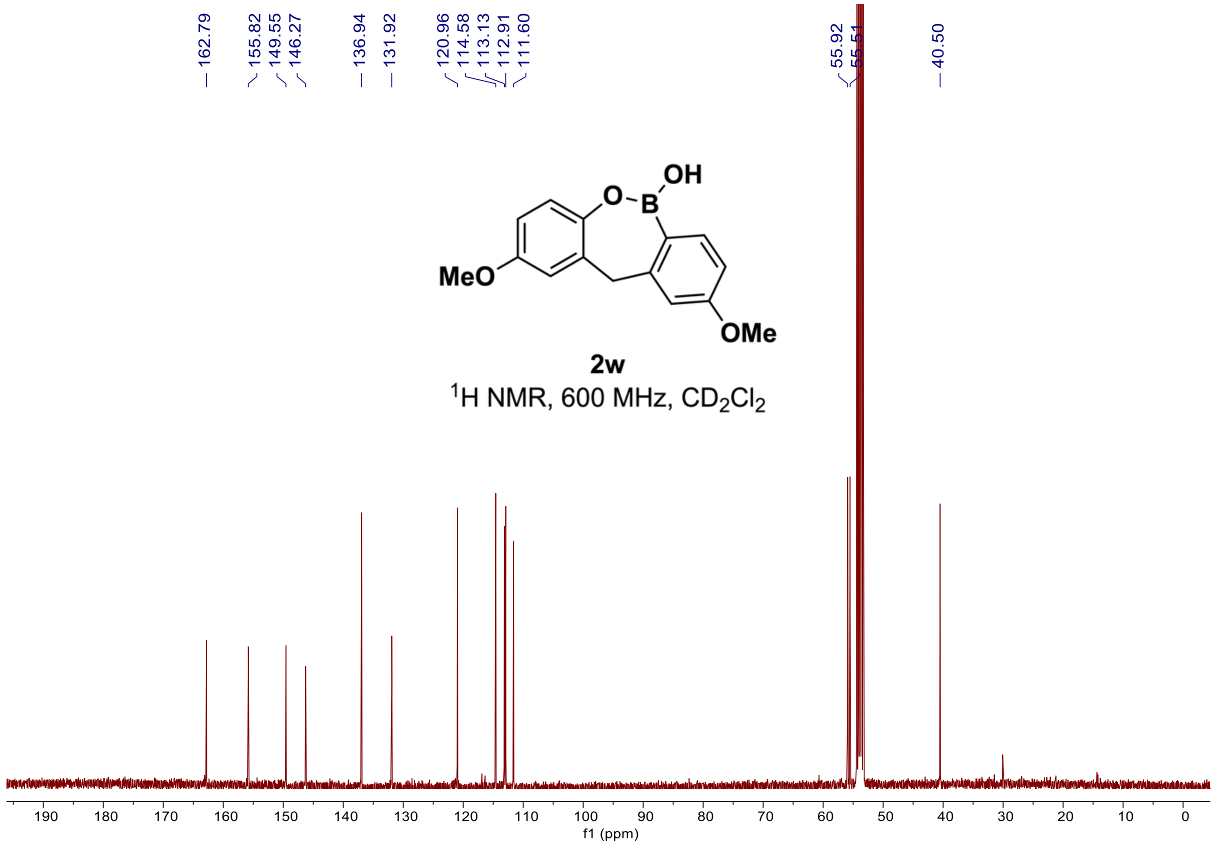
162.79
155.82
149.55
146.27
136.94
131.92
120.96
114.58
113.13
112.91
111.60
55.92
55.51
40.50
OH
O
B
MeO
OMe
2w
^{1}H NMR, 600 MHz, CD_2Cl_2
f1 (ppm)

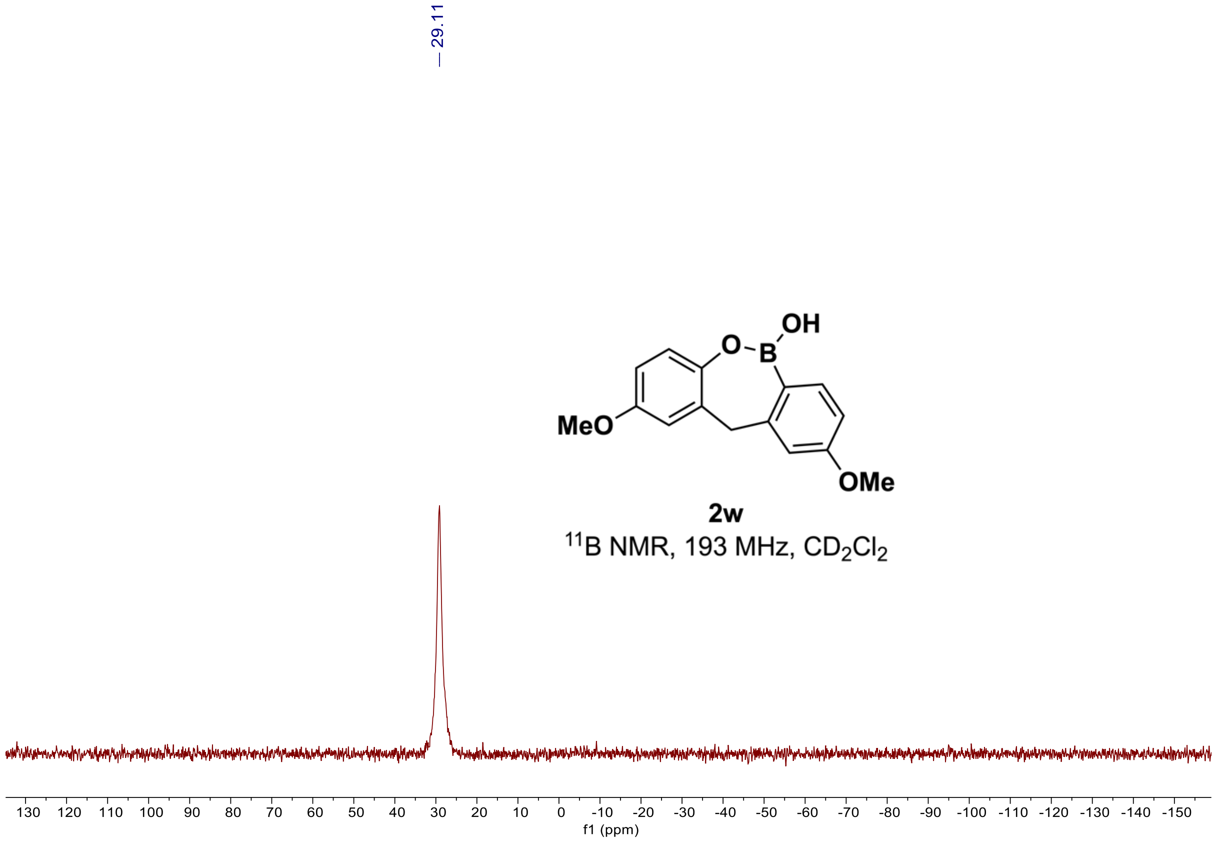
29.11
OH
O
B
MeO
OMe
2w
^{11}B NMR, 193 MHz, CD_2Cl_2
f1 (ppm)

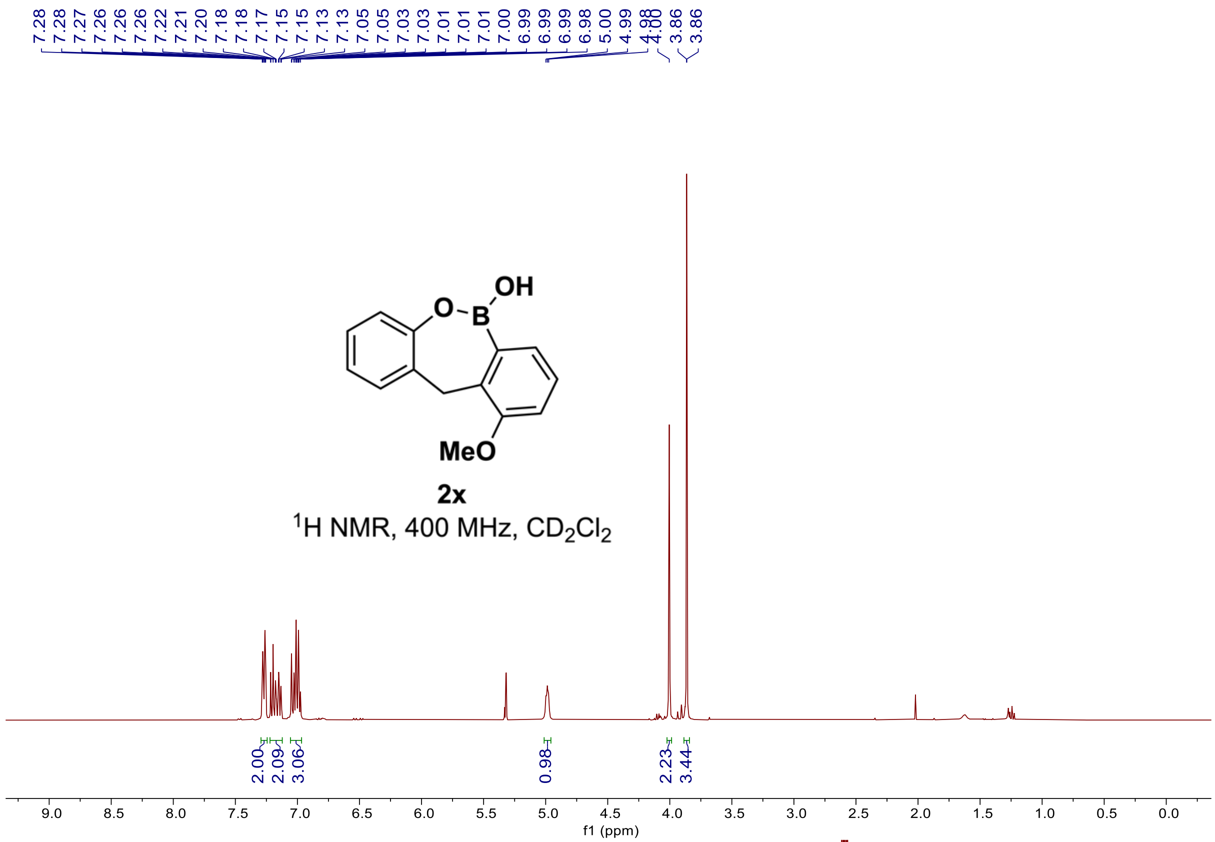
7.28 7.28 7.27 7.26 7.26 7.26 7.22 7.21 7.20 7.18 7.18 7.17 7.15 7.15 7.13 7.13 7.05 7.05 7.03 7.03 7.01 7.01 7.01 7.00 6.99 6.99 6.98 5.00 4.99 4.98 4.00 3.86 3.86
OH
O
B
MeO
2x
1H NMR, 400 MHz, CD2Cl2
2.00 2.09 3.06 0.98 2.23 3.44
9.0 8.5 8.0 7.5 7.0 6.5 6.0 5.5 5.0 4.5 4.0 3.5 3.0 2.5 2.0 1.5 1.0 0.5 0.0
f1 (ppm)

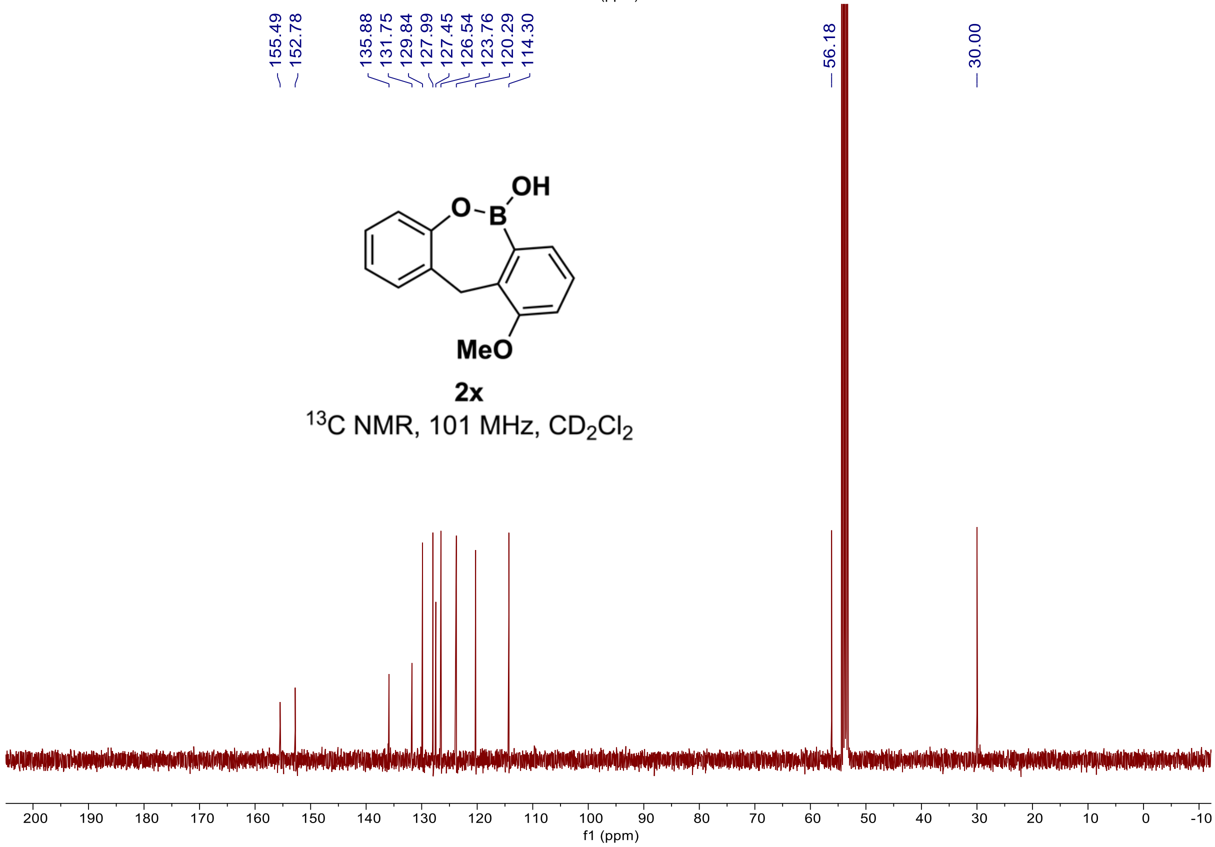
155.49 152.78 135.88 131.75 129.84 127.99 127.45 126.54 123.76 120.29 114.30 56.18 30.00
OH
O
B
MeO
2x
13C NMR, 101 MHz, CD2Cl2
200 190 180 170 160 150 140 130 120 110 100 90 80 70 60 50 40 30 20 10 0 -10
f1 (ppm)

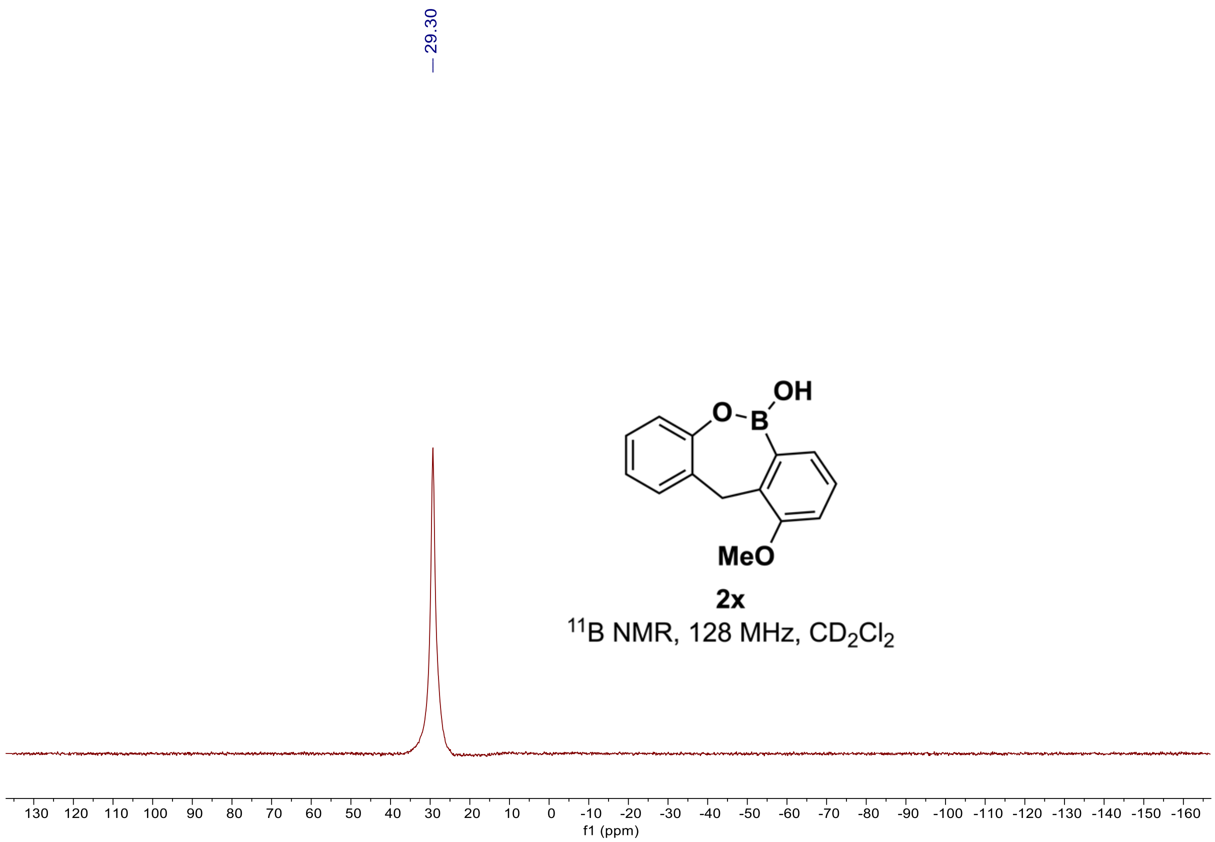
29.30
OH
O
B
MeO
2x
^{11}B NMR, 128 MHz, CD_2Cl_2
130 120 110 100 90 80 70 60 50 40 30 20 10 0 -10 -20 -30 -40 -50 -60 -70 -80 -90 -100 -110 -120 -130 -140 -150 -160
f1 (ppm)

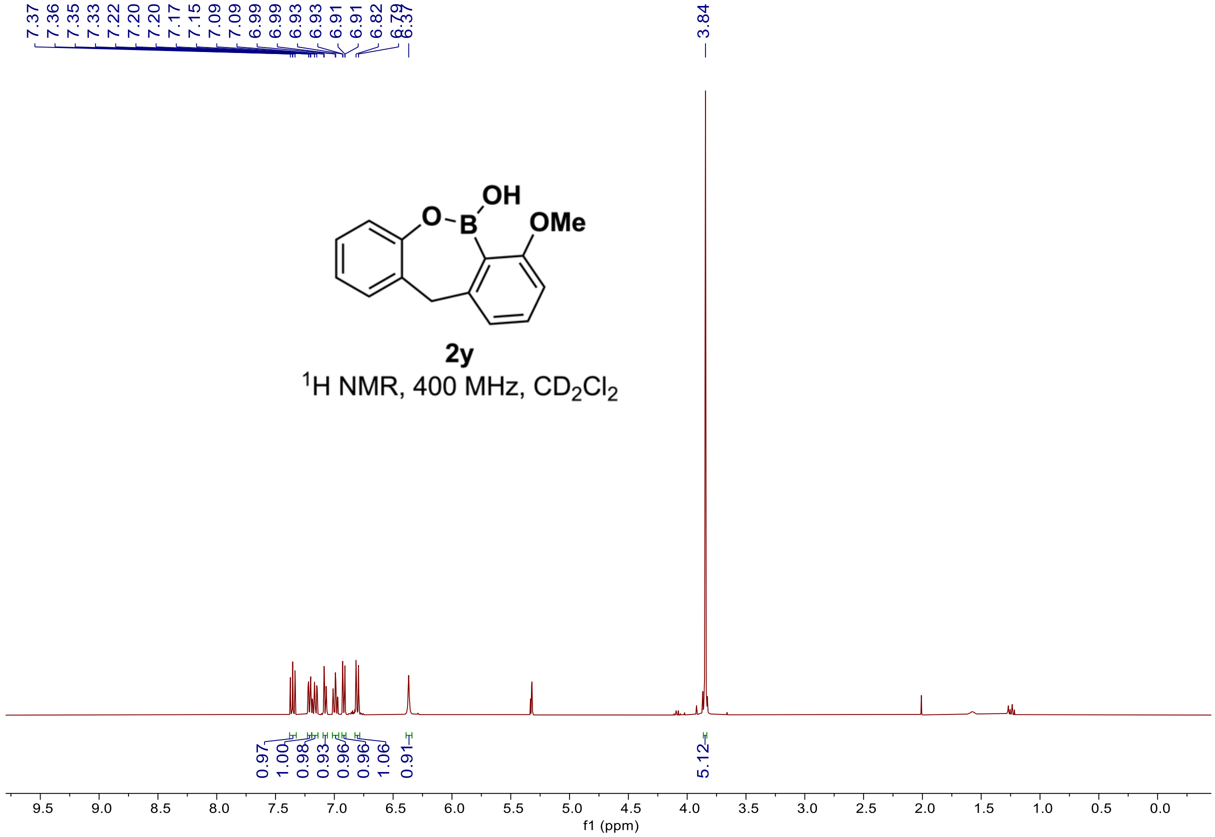
7.37 7.36 7.35 7.33 7.22 7.20 7.20 7.17 7.15 7.09 7.09 6.99 6.99 6.93 6.93 6.91 6.91 6.82 6.79 6.37
3.84
OH
O
B
OMe
2y
^{1}H NMR, 400 MHz, CD_2Cl_2
0.97 1.00 0.98 0.93 0.96 0.96 1.06 0.91
5.12
9.5 9.0 8.5 8.0 7.5 7.0 6.5 6.0 5.5 5.0 4.5 4.0 3.5 3.0 2.5 2.0 1.5 1.0 0.5 0.0
f1 (ppm)

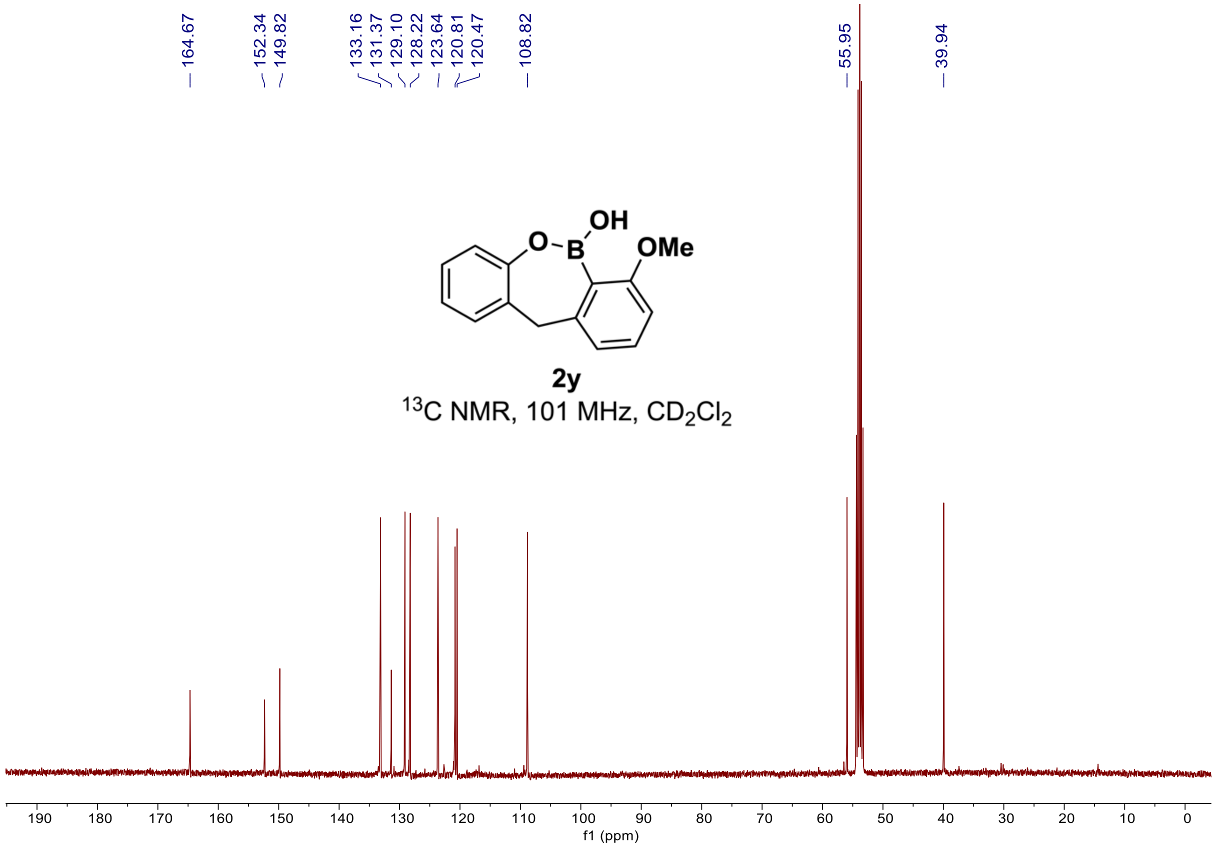
164.67
152.34
149.82
133.16
131.37
129.10
128.22
123.64
120.81
120.47
108.82
55.95
39.94
OH
O
B
OMe
2y
^{13}C NMR, 101 MHz, CD_2Cl_2
190 180 170 160 150 140 130 120 110 100 90 80 70 60 50 40 30 20 10 0
f1 (ppm)

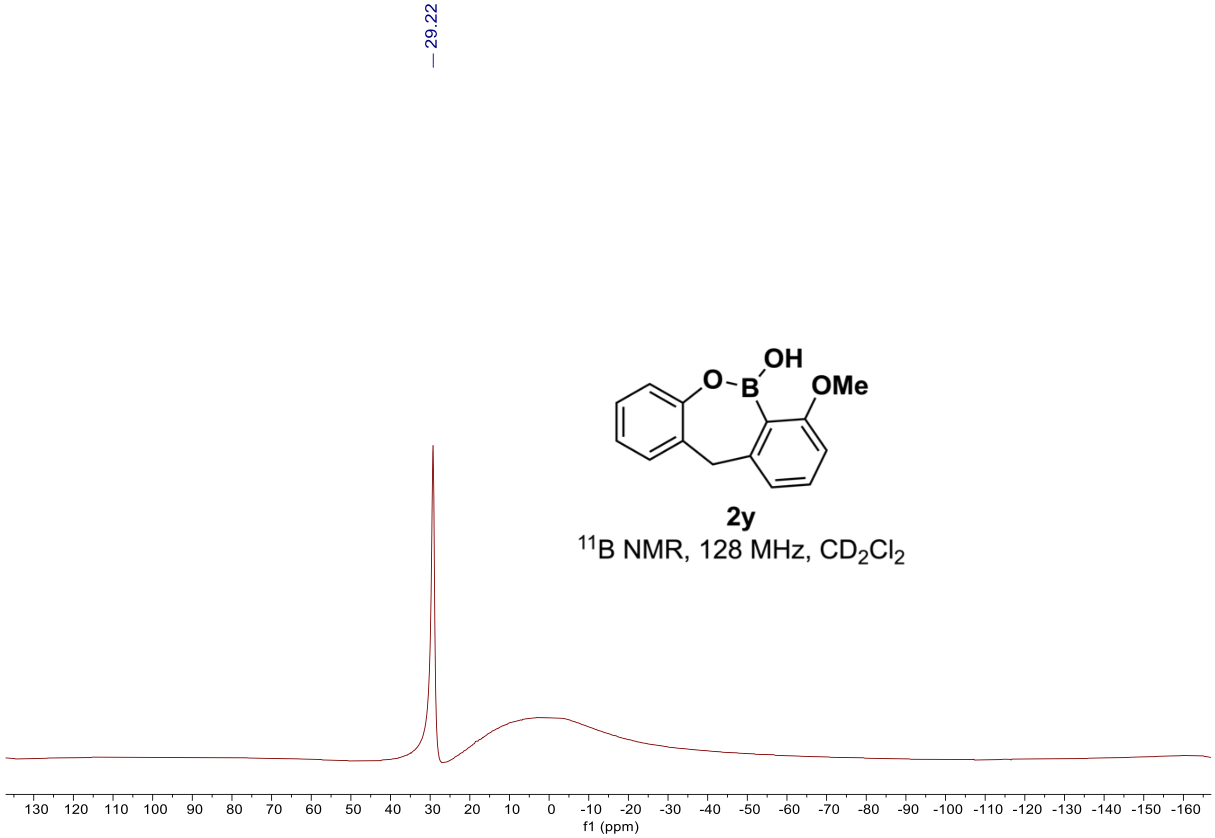
29.22
OH
O
B
OMe
2y
^{11}B NMR, 128 MHz, CD_2Cl_2
130 120 110 100 90 80 70 60 50 40 30 20 10 0 -10 -20 -30 -40 -50 -60 -70 -80 -90 -100 -110 -120 -130 -140 -150 -160
f1 (ppm)

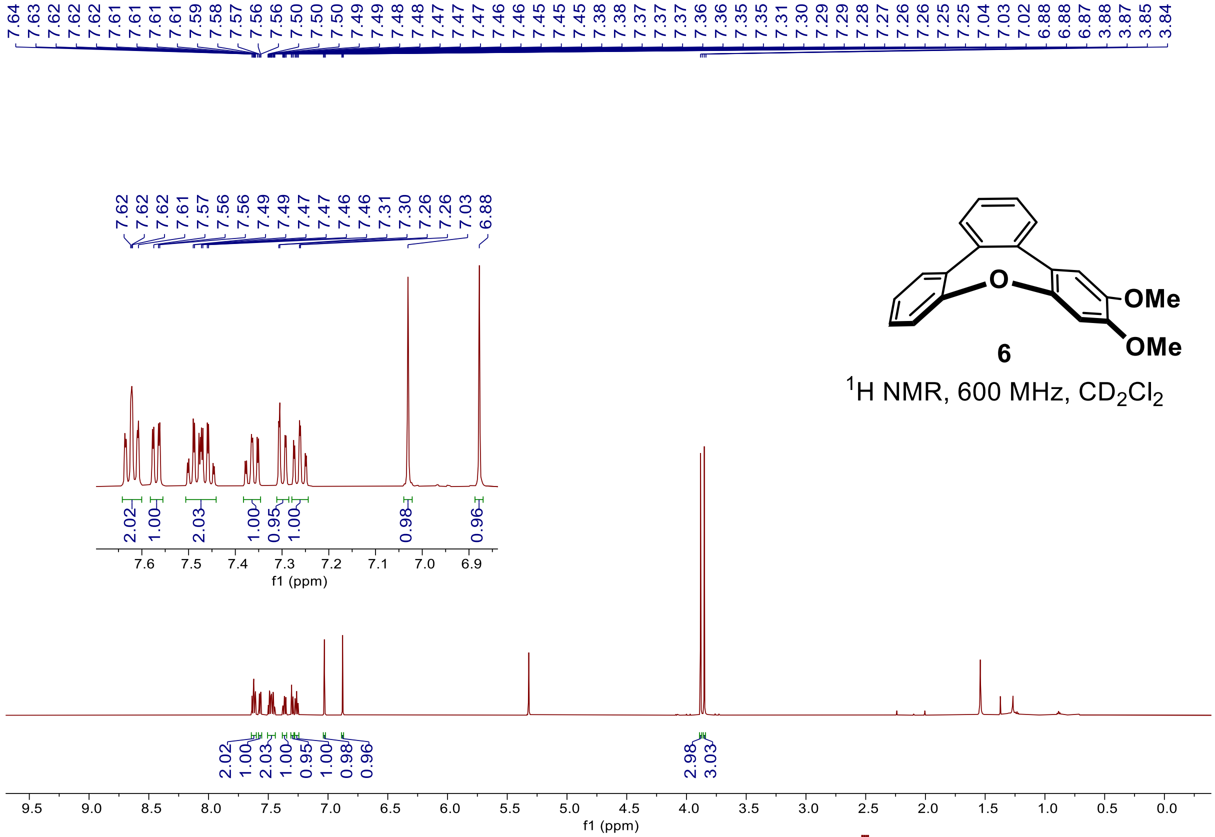
6
^{1}H NMR, 600 MHz, CD_2Cl_2
f1 (ppm)

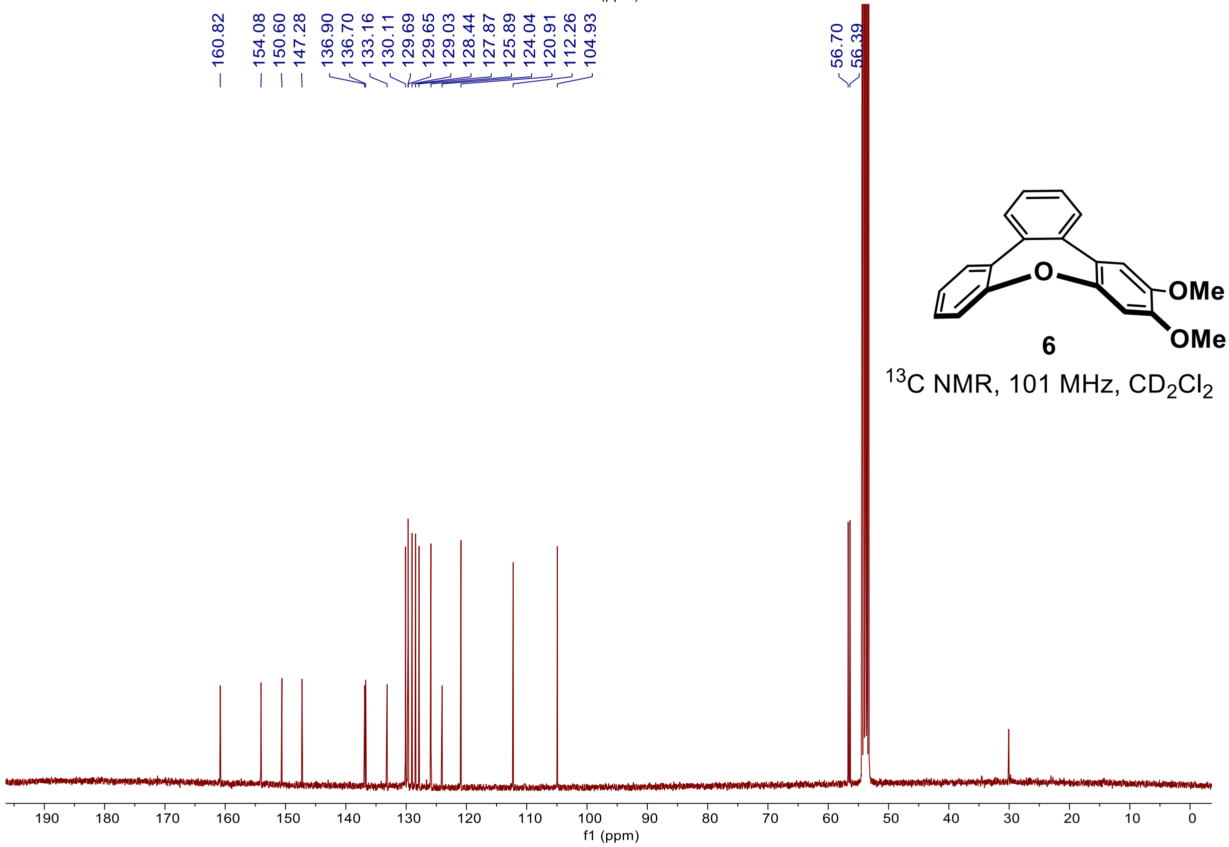
6
^{13}C NMR, 101 MHz, CD_2Cl_2
f1 (ppm)

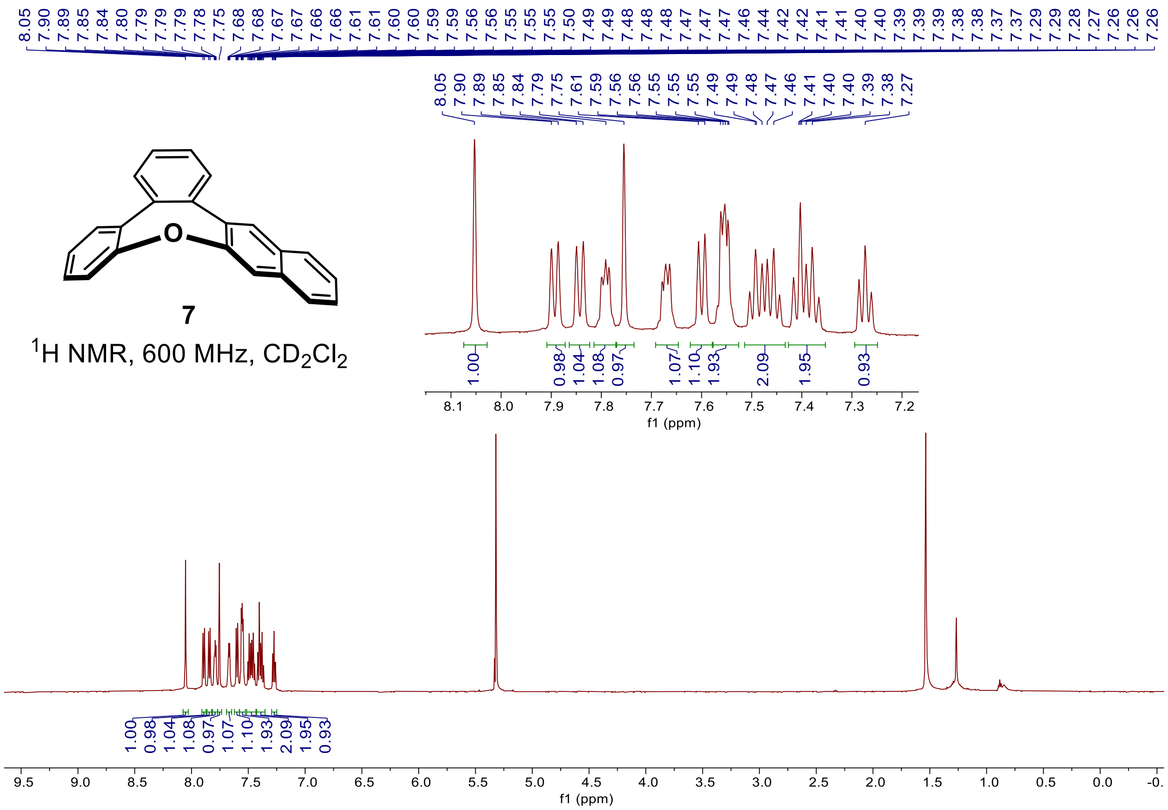
8.05 7.90 7.89 7.85 7.84 7.80 7.79 7.79 7.79 7.78 7.75 7.68 7.68 7.67 7.66 7.66 7.61 7.61 7.60 7.60 7.59 7.59 7.56 7.56 7.55 7.55 7.55 7.50 7.49 7.49 7.48 7.48 7.47 7.47 7.47 7.46 7.44 7.42 7.42 7.41 7.41 7.40 7.40 7.39 7.39 7.39 7.38 7.38 7.37 7.37 7.29 7.29 7.28 7.27 7.26 7.26 7.26
8.05 7.90 7.89 7.85 7.84 7.79 7.75 7.61 7.59 7.56 7.56 7.55 7.55 7.49 7.49 7.48 7.47 7.46 7.41 7.40 7.40 7.39 7.38 7.27
O
7
^{1}H NMR, 600 MHz, CD_2Cl_2
1.00 0.98 1.04 1.08 0.97 1.07 1.10 1.93 2.09 1.95 0.93
f1 (ppm)

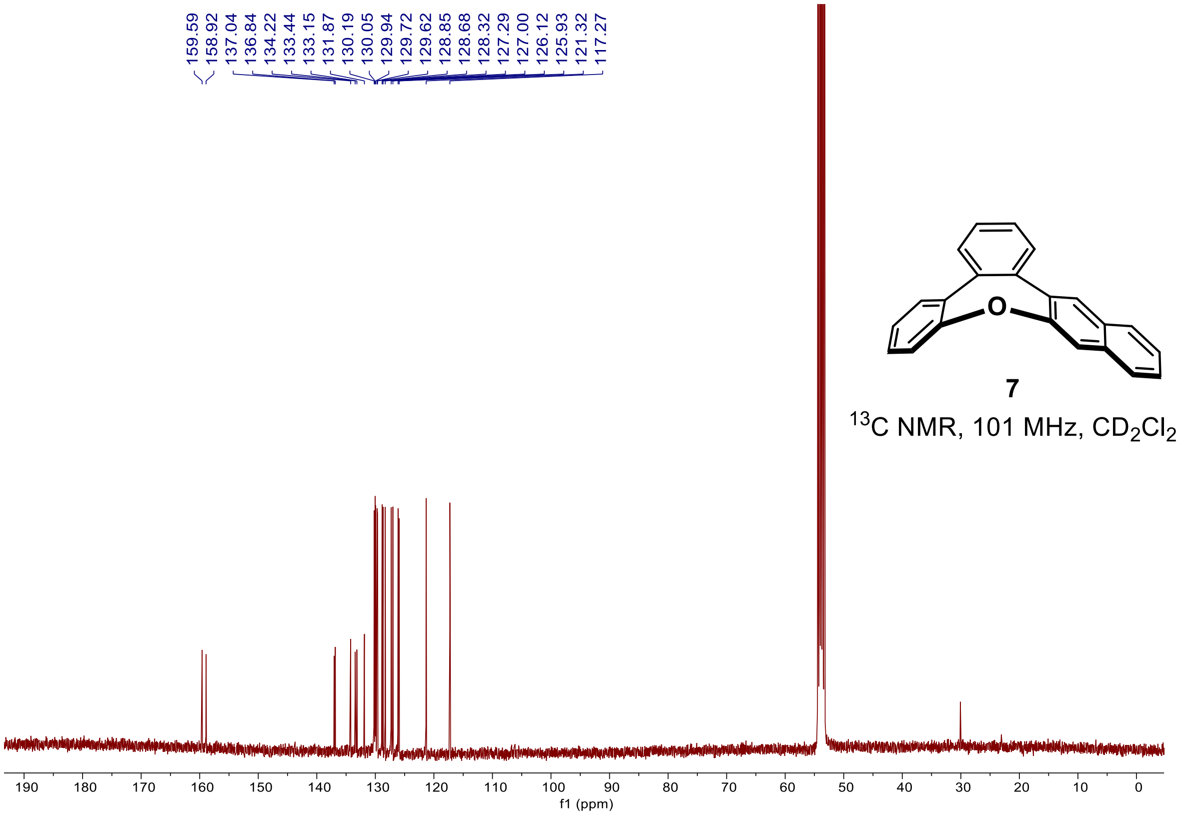
159.59 158.92 137.04 136.84 134.22 133.44 133.15 131.87 130.19 130.05 129.94 129.72 129.62 128.85 128.68 128.32 127.29 127.00 126.12 125.93 121.32 117.27
O
7
^{13}C NMR, 101 MHz, CD_2Cl_2
f1 (ppm)
